# Synthesis and In Vitro Activity of Hypofuran B and Analogs Against *Plasmodium Falciparum* and *Trypanosoma Cruzi*

**DOI:** 10.1002/cmdc.202500719

**Published:** 2025-11-30

**Authors:** Cristiane Aparecida Franco, Jodieh Oliveira Santana Varejão, Isabela Penna Ceravolo, Victória Miranda Machado, Antoniana Ursine Krettli, Daniela de Melo Resende, Silvane Maria Fonseca Murta, Felipe Terra Martins, Eduardo Jorge Pilau, Vinícius Ribeiro Montes, Markus Kohlhoff, Eduardo V. V. Varejão

**Affiliations:** ^1^ Laboratory of Natural Product Chemistry and Organic Synthesis Department of Chemistry Universidade Federal de Viçosa Avenida PH Rolfs s/n Viçosa 36570–900 Brazil; ^2^ Laboratory of Immunopathology Fundação Oswaldo Cruz (FIOCRUZ) René Rachou Institute (IRR) Avenida Augusto de Lima, 1715 Belo Horizonte Minas Gerais 30190–002 Brazil; ^3^ Laboratory of Functional Genomics of Parasites Fundação Oswaldo Cruz (FIOCRUZ) René Rachou Institute (IRR) Avenida Augusto de Lima, 1715 Belo Horizonte Minas Gerais 30190–002 Brazil; ^4^ Institute of Chemistry Universidade Federal de Goiás Avenida Esperança, sn, Samambaia Goiânia 690–900 Brazil; ^5^ Laboratory of Biomolecules and Mass Spectrometry Universidade Estadual de Maringá Paraná 87020–900 Brazil; ^6^ Laboratory of Natural Products Chemistry Fundação Oswaldo Cruz (FIOCRUZ) Avenida Augusto de Lima, 1715 Belo Horizonte Minas Gerais 30190–002 Brazil

**Keywords:** 5‐HMF, chagas disease, furfural, malaria, neglected tropical diseases

## Abstract

Herein, the synthesis and biological evaluation of hypofuran B and a series of analogs against *Trypanosoma cruzi* and *Plasmodium falciparum* is described. The compounds are obtained through crossed aldol condensation between phenylacetaldehyde and furfural derivatives, using reaction conditions optimized according to the aromatic substituents. Yields ranged from 20% to 83%, with *E*/*Z* ratios between 89:11 and 98:2. Three compounds are isolated as single crystals suitable for X‐ray diffraction, and their crystal structures are determined. The most active analogs showed IC_50_ values of 5.35–10.35 µg mL^−1^ and are further evaluated for cytotoxicity in L929 cells. For *P. falciparum*, a clear structure–activity–toxicity relationship is observed. The most promising compound displayed a CC_50_ value above 400 µg mL^−1^, indicating lower cytotoxicity than chloroquine. In silico predictions also supported favorable drug‐like profiles. Overall, the moderate antiparasitic activity, low cytotoxicity, and consistent structure–activity trends highlight hypofuran B and related drynaran derivatives as promising antimalarial leads.

## Graphical abstract

Hypofuran B, drynaran, and a series of analogs are obtined by crossed aldol condensation from furfural dereivatives. Compunds are tested for in vitro activity agaisnt *Trypanosoma cruzi* and *Plasmodium falciparum*. Best results are obtained Against *P. falciparum*, with IC_50_ values ranging from 5,35 to 10,35 µg mL^−1^. A clear structure activity relationship is observed. The most promising compounds showed low cytotoxicity on L929 human cell line (CC_50_ > 400 µg mL^−1^) and suitable in silico drug‐like parameters according to the Lipinski's rule of 5.

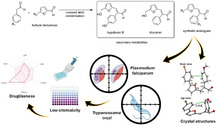

## 1 Introduction

Chagas disease and malaria are parasitic infections that continue to impose substantial burdens on global public health.^[^ ^1^ ^]^ Chagas disease, first described by the Brazilian physician Carlos Chagas, is caused by *Trypanosoma cruzi* and primarily transmitted by hematophagous triatomine insects (“kissing bugs”). Transmission occurs when parasite‐containing feces enter the host's bloodstream through skin lesions created during feeding.^[^ ^2^ ^]^ The disease remains endemic in Latin America, with an estimated 6–8 million people infected and around 75 million at risk. In addition to vector‐borne transmission, infection may occur through contaminated food, congenital exposure, blood transfusion, organ transplantation, or reactivation in immunocompromised individuals. Increasing migration from endemic regions has led to a growing number of cases in nonendemic countries, including those in Europe, North America, Japan, and Australia.^[^ ^3^ ^]^

Chagas disease is curable if treated early, during the acute phase; however, this stage is often asymptomatic or presents with mild, nonspecific fever, making diagnosis difficult. In the chronic phase, *T. cruzi* persists mainly in cardiac and digestive muscle tissues. One to three decades after infection, up to one‐third of patients develop cardiac disorders and ≈10% develop digestive or mixed forms, which may progress to arrhythmias, heart failure, or sudden death.^[^ ^4^ ^]^ Since 2005, the World Health Organization (WHO) has classified Chagas disease among the Neglected Tropical Diseases (NTDs), a group that disproportionately affects impoverished populations in developing regions. These diseases receive limited attention and funding due to low commercial interest, weak political prioritization, and competition with conditions of higher mortality.^[^ ^5^ ^]^ Currently, only two *N*‐heterocyclic drugs, benznidazole and nifurtimox, are approved for Chagas treatment. Both are effective when administered during the acute phase, but prolonged therapy, variable efficacy, and adverse effects limit their use. Cure rates in the chronic phase range from 5% to 20% after long‐term follow‐up, and drug resistance among *T. cruzi* strains remains a challenge.^[^ ^6^, ^7^, ^8^ ^]^ Consequently, the development of safer and more effective treatments remains an urgent priority.

Like Chagas disease, malaria is caused by protozoan parasites and continues to represent a major global health problem, particularly in tropical and subtropical regions. It is transmitted through the bite of infected female *Anopheles* mosquitoes carrying *Plasmodium* species, mainly *P. falciparum* and *P. knowlesi*. The WHO estimated 247 million malaria cases and more than 600,000 deaths worldwide, with 94% of fatalities occurring in Africa.^[^ ^9^, ^10^, ^11^, ^12^ ^]^ In 2023, global cases reached ≈263 million, 97% of which were reported in African countries.

Current antimalarial therapies rely mainly on chloroquine or artemisinin in combination with partner drugs (artemisinin‐based combination therapy, ACT). However, the growing resistance to both chloroquine and artemisinin threatens control efforts and underscores the urgent need for new antimalarial agents.^[^ ^12^ ^,^ ^13^ ^]^

In pursuit of novel scaffolds for anti‐*T. cruzi* and antiplasmodial agents, our research group has established collaborations among several Brazilian teams specializing in natural product synthesis and tropical parasitology. This partnership has enabled the evaluation of multiple compound classes as potential leads for Chagas and malaria therapies.

**Figure 1:**
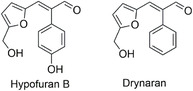
Chemical structures of hypofuran B and drynaran.

In the present study, we report, for the first time, the synthesis of the natural compound hypofuran B and a series of analogs, together with their in vitro evaluation against *P. falciparum* and *T. cruzi*. Hypofuran B [(*E*)‐3‐(5‐(hydroxymethyl)furan‐2‐yl)‐2‐(4‐hydroxyphenyl)acrylaldehyde] is a phenolic compound isolated from the marine‐derived fungus *Hypocrea koningii* PF04—the teleomorphic (sexual) stage of the *Trichoderma* genus, well known for producing bioactive metabolites of medicinal interest.^[^ ^14^ ^]^ Hypofuran B was originally isolated in a yield of only 3 mg and exhibited no significant antibacterial activity against *Staphylococcus aureus* or *Escherichia coli*. Structurally, it is closely related to drynaran, a natural compound isolated from the rhizomes of *Drynaria bonii* H. Christ, a fern used in traditional Asian medicine. The sole structural difference between the two is the presence of a hydroxyl group on the aromatic ring of hypofuran B (**Figure** **1**). Like hypofuran B, drynaran was isolated in minute quantities (9 mg) and exhibited modest antibacterial and antioxidant activity.^[^ ^15^ ^,^ ^16^ ^]^

Herein, we report the first synthesis of hypofuran B along with a series of methoxylated analogs and two new drynaran derivatives. Some of these compounds were obtained as single crystals suitable for X‐ray diffraction, and their crystal structures are described. All synthesized compounds, together with previously reported analogs, were evaluated for their in vitro activity against *T. cruzi* and *P. falciparum*, as well as for cytotoxicity. Finally, in silico drug‐likeness parameters were calculated for the most active molecules.

## 2 Results and Discussion

### 2.1 Synthesis

**Scheme 1:**
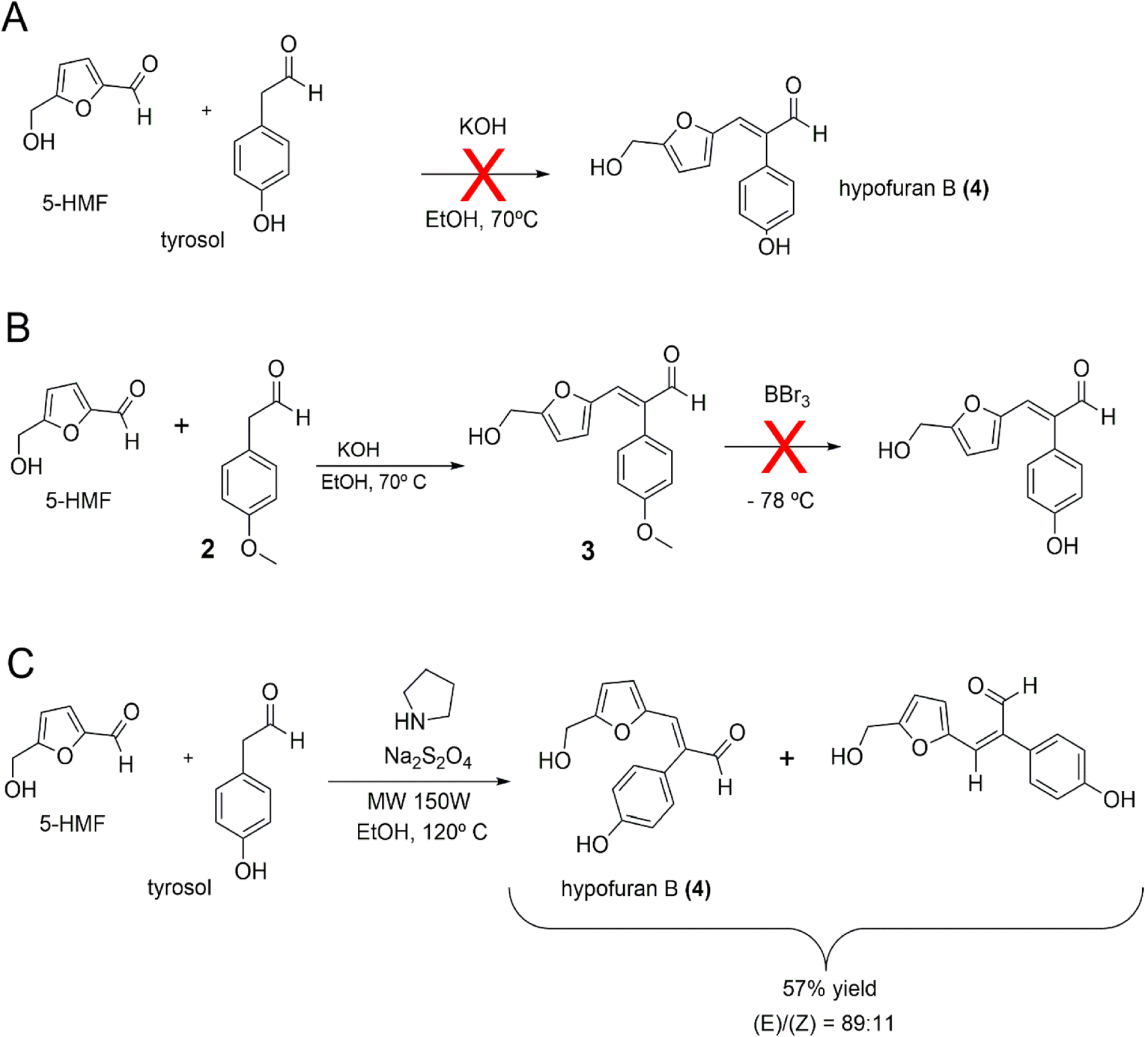
Synthetic routes attempted for the synthesis of hypofuran B.

Drynaran and its derivatives (compounds **16–26**) were synthesized previously.^[^ ^17^ ^]^ Structurally, hypofuran B (**3**) differs from drynaran (**16**) by the presence of a phenolic hydroxyl group. Our initial attempt to prepare hypofuran B (**3**) followed the same base‐catalyzed crossed aldol condensation used for drynaran and its analogs,^[^ ^17^ ^]^ involving 5‐hydroxymethyl‐2‐furfural (5‐HMF) and 2‐(4‐hydroxyphenyl)acetaldehyde with KOH as the catalyst (**Scheme** **1**A). However, this reaction failed owing to deprotonation of the phenolic hydroxyl group, which led to precipitation of the corresponding phenolate.

To circumvent this issue, 2‐(4‐hydroxyphenyl)acetaldehyde was methylated to yield 2‐(4‐methoxyphenyl)acetaldehyde (**1**), thereby protecting the hydroxyl group during condensation (Scheme 1B).^[^ ^18^ ^]^ This afforded the 4‐methoxyphenyl analog of hypofuran B (**2**) in 33% yield. Subsequent treatment of compound (2) with BBr_3_ did not produce hypofuran B (**3**) but rather a complex mixture of by‐products, most likely due to decomposition of the 5‐hydroxymethyl‐2‐furfural moiety under the acidic conditions generated by in situ HBr formation from BBr_3_.^[^ ^19^, ^20^, ^21^ ^]^

**Figure 2:**
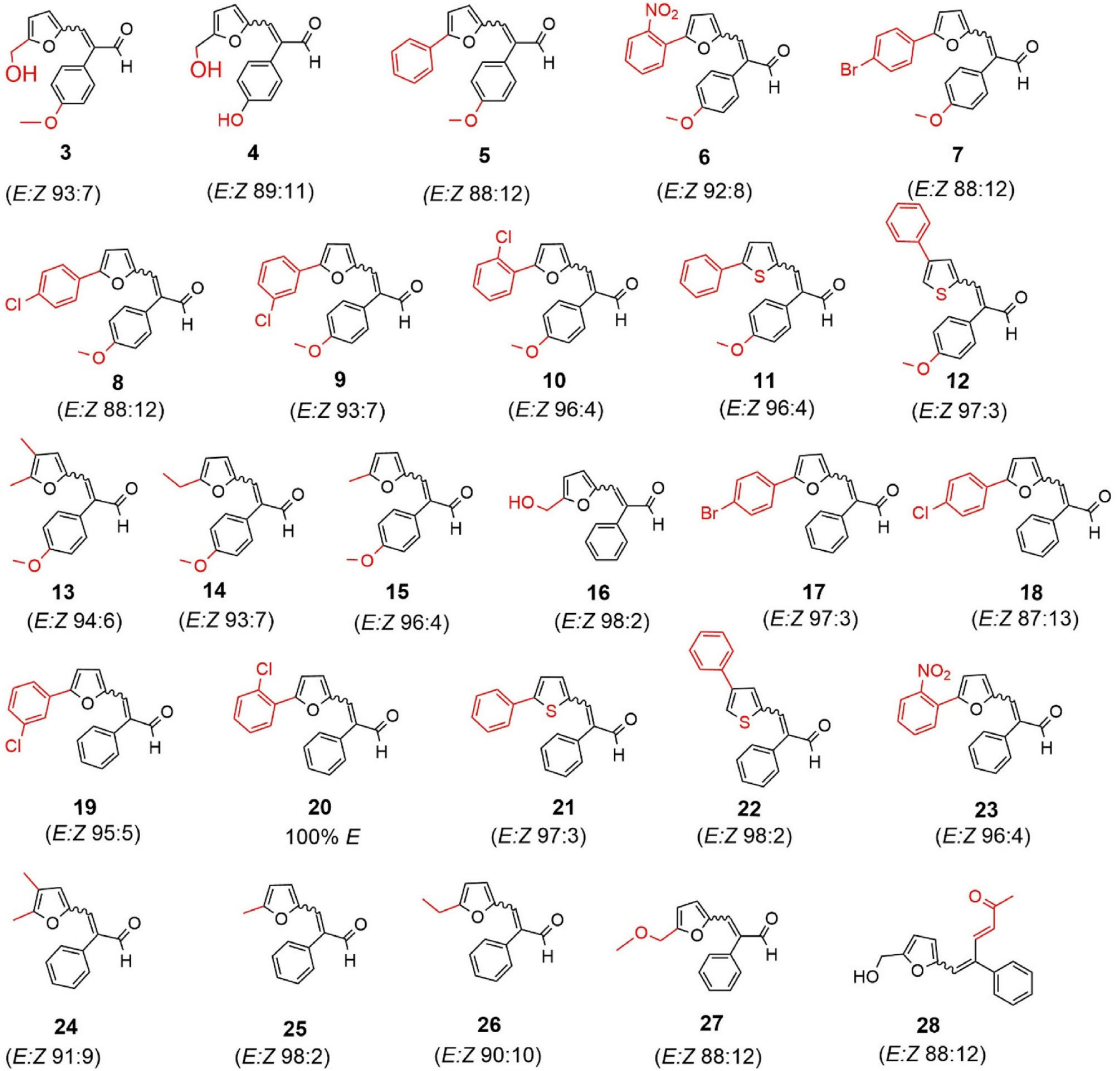
Chemical structures and diastereomeric ratios of the synthesized compounds (**3–15**, **27**, and **28**), together with drynaran (**16**) and its analogues (**17–26**).

An alternative approach was therefore pursued, employing a methodology described by Limnios and Kokotos,^[^ ^22^ ^]^ which enables crossed aldol condensations of aldehydes under microwave irradiation using pyrrolidine as a catalyst (Scheme 1C). We slightly modified this procedure by adding sodium dithionite (Na_2_S_2_O_4_), known to minimize polymerization of 5‐HMF at elevated temperatures^[^ ^19^ ^]^. This method afforded hypofuran B (3) in 57% yield with an *E*/*Z* ratio of 89:11. All other compounds synthesized in this study were obtained following the same procedure as for compound (3) from (2), as shown in Scheme 1B. Their structures and corresponding diastereomeric ratios are displayed in **Figure** **2**.

The EI–MS spectrum of hypofuran B (**3**) showed a molecular ion peak at *m/z* 244 and a base peak at *m/z* 213, consistent with *β*‐cleavage adjacent to the hydroxyl group. HRMS confirmed the molecular formula with a [M + Na^+^] peak at *m/z* 267.0623.

In the ^1^H NMR spectrum, a singlet at *δ* 4.33 (2H) corresponded to the methylene protons H‐6. Signals at *δ* 6.06 and 6.16 were assigned to the furan protons H‐4 and H‐3, respectively, with COSY cross‐peaks confirming their mutual coupling. Aromatic protons H‐9 and H‐9′ appeared as a doublet at *δ* 6.74, shielded by the neighboring hydroxyl group, and showed COSY correlation with H‐8 and H‐8′ at *δ* 6.89. The vinylic proton H‐3′ displayed a NOESY correlation with the aldehydic proton H‐1′ at *δ* 9.49, confirming the predominance of the (E)‐isomer. Integration of H‐1′ signals corresponding to both isomers established an E/Z ratio of 89:11.

**Figure 3:**
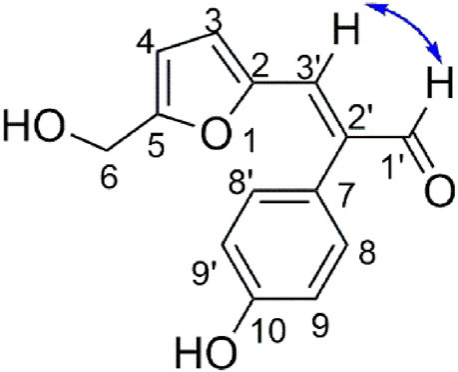
NOESY correlations observed for hypofuran B.

In the ^13^C NMR spectrum (**Figure** **3**), C‐6 resonated at *δ* 56.50. The furan carbons C‐3 and C‐4 were assigned to *δ* 109.81 and 116.71, while aromatic carbons appeared at *δ* 115.30 and 130.58. The vinylic carbon C‐3′ and aldehydic carbon C‐1′ resonated at *δ* 135.46 and 192.38, respectively.

For the 4‐methoxyphenyl analog of hypofuran B (compound **4**), HRMS displayed a [M + H^+^] peak at *m/z* 259.0955, in excellent agreement with the calculated value. In the NMR spectra, the methyl group signals appeared at *δ* 3.87 (^1^H) and *δ* 55.41 (^13^C). The spectroscopic data for all other synthesized compounds were consistent with the proposed structures.In each case, the NOESY correlation between H‐3′ and H‐1′ confirmed the configuration of the major diastereomer, while integration of H‐3′ signals provided the *E*/*Z* ratios. All compounds were predominantly obtained as the (E)‐isomers, with ratios ranging from 89:11 to 98:2, consistent with earlier findings for drynaran (16) and its analogs (17–26).

**Scheme 2:**
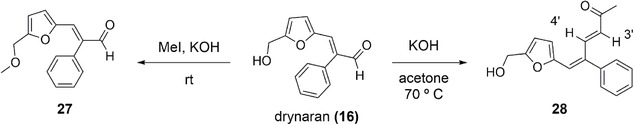
Synthesis novel drynaran derivatives.

Additionally, two new derivatives (27 and 28) were synthesized from drynaran (**Scheme** **2**). Compound (27) was obtained by methylation of the aliphatic hydroxyl group, whereas compound (28) was prepared via aldol condensation between drynaran and acetone, yielding the product in 77% yield. In both cases, the (*E*)‐isomer predominated. Spectroscopic characterization (MS and NMR) confirmed the proposed structures. For compound (28), a coupling constant (*J*) of 15 Hz between vicinal olefinic protons H‐3′ and H‐4′ clearly indicated an (*E*)‐configuration for the *β*,*γ*‐double bond.

### 2.2 Crystallography

**Figure 4:**
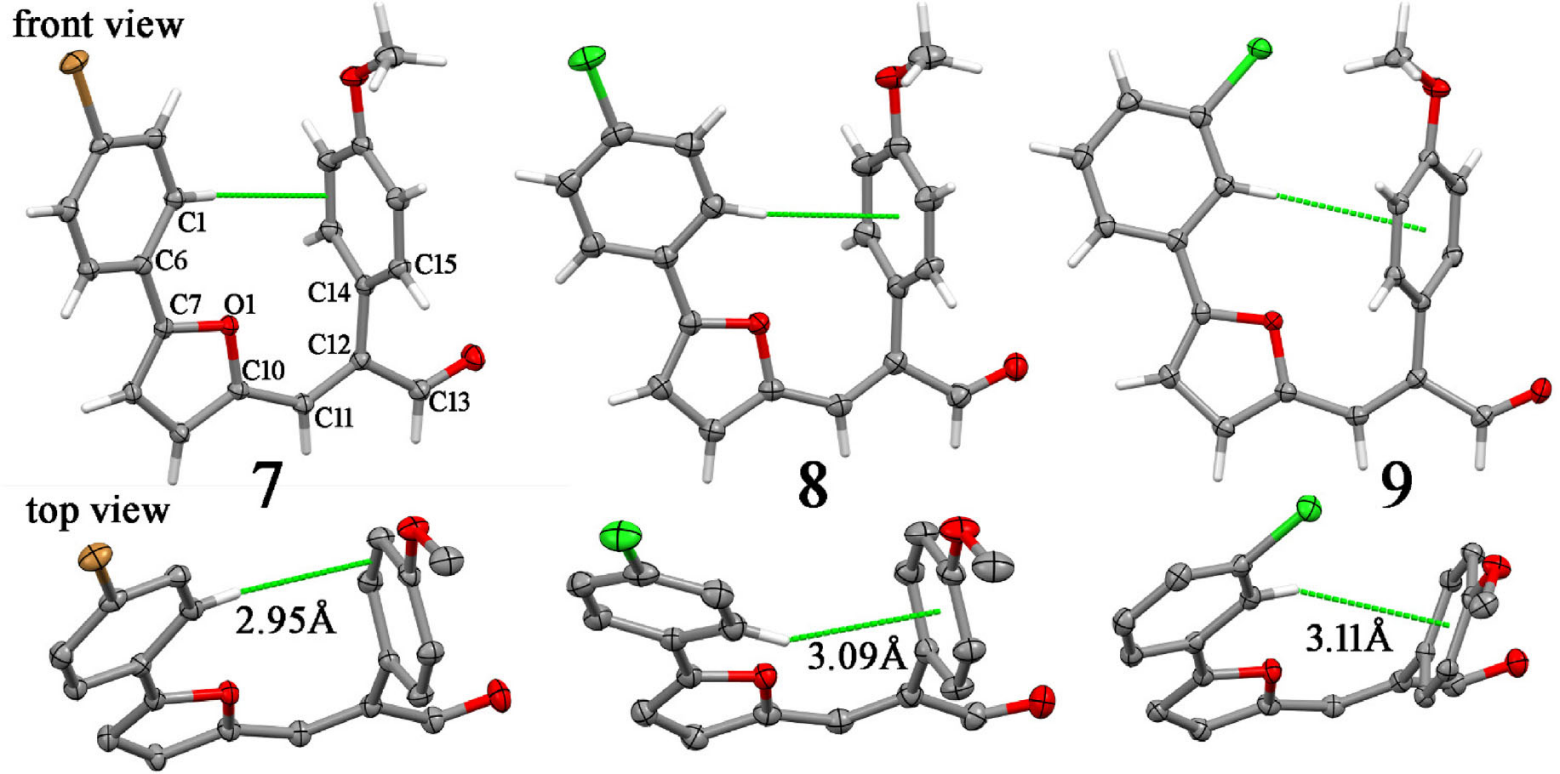
A 50% probability ellipsoid drawing for nonhydrogen atoms present in the asymmetric units of **7**–**9** (hydrogens are depicted as sticks, and only that H atom involved in the CH…p intramolecular interaction is not hidden in the top view panel). Green dashed lines depict these noncovalent bonds, and H…Cg distance is shown, where Cg is the centroid over the two bonded carbons arriving the green line in **7**, or the six carbons enclosing the methoxylated phenyl ring in **8** and **9**). Some atoms were labeled in **7** to follow the torsions chosen to describe the conformational features os the three compounds.

The three compounds were elucidated with one molecule in the asymetric unit belonging to the centrosymetric monoclinic space groups *P*2_1_/*n* (**7** and **9**) or *P*2_1_/*c* (**8**) (**Figure** **4**). Despite of their distinct halogen substitution patterns, the three compounds have similar molecular backbones. Their halogen substituted phenyl and furan rings show high coplanarity, with slight torsion around the C—C bond between them. For instance, the O1‐C7‐C6‐C1 angle measures 19.0(2)°, −18.9(2)° and 11.8(2)° in **7**, **8,** and **9**, respectively. And this coplanarity fetaure is extended toward the open chain, with the furan ring having even higher coplanarity with the neighboring chain ending into aldehyde (the O1‐C10‐C11‐C12 angle measures −1.0(3)°, −1.2(2)° and 3.9(2)° in in **7**, **8,** and **9**, respectively). The methoxy group is also coplanar to its bonded phenyl ring, but, in contrary, the whole methoxylated phenyl moiety is almost perpendicular to the open chain, which can be described by the C13‐C12‐C14‐C15 torsion around the CC bond linking these molecular motifs (−84.4(2)°, −77.1(2)° and −117.7(2)° in **7**, **8,** and **9**, respectively). This conformational pattern gives rise to U‐shaped molecules present with an intramolcular CH…p interaction engaging the CH group at *para*‐position of the halogen substituted phenyl ring and the p‐electronic cloud of the methoxylated phenyl ring. If the shortest distance between the interacted H atom and a centroid calculated over the bonds between phenyl carbons or the six ones is considered (Figure 4), it is possible to state that this CH…p interaction is localized on a specific C=C bond in **7** while it is delocalized over the phenyl ring in **8** and **9**.

### 2.3 In Vitro Activity Against *T. cruzi* and *P. Falciparum*

In the initial screening, all compounds inhibited at least 70% of *Trypanosoma cruzi* growth when tested at 100 µg mL^−1^ and were therefore subjected to further assays to determine their half‐maximal inhibitory concentrations (IC_50_)^[^ ^23^, ^24^ ^]^ (**Table** **1**). Compounds exhibiting IC_50_ values equal to or lower than 30 µg mL^−1^ were subsequently evaluated for cytotoxicity against the L929 cell line, a rapidly proliferating murine fibroblast line widely employed to assess the cytotoxic potential of drug candidates. Their half‐maximal cytotoxic concentrations (CC_50_) and selectivity indices (SI = CC_50_/IC_50_) are also presented in Table 1.

**Table 1:** Half‐maximal inhibitory concentrations (IC_50_) against *Trypanosoma cruzi* and the chloroquine‐resistant *plasmodium falciparum* W2 clone, half‐maximal cytotoxic concentrations (CC_50_) against L929 cells, and calculated selectivity indices (SI).

Compound	CC_50_ b)[µM]	*T. cruzi*		*P. falciparum*	
		IC_50_ a) [µM]	SIc)	IC_50_ [µM]	SI
**3**	–	≥406	–	≥123	–
**4**	418	322	–	30.4	13.8
**5**	482	272	–	22.7	21.3
**6**	–	174	–	74.1	–
**7**	42.1	33.2	1.27	130	–
**8**	53.7	45.2	1.19	147	–
**9**	–	286	–	147	–
**10**	–	≥293	–	89.2	–
**11**	≥1,240	136	≥9.11	32.1	≥38.6
**12**	53.8	116	–	21.6	2.50
**13**	387	387	–	20.7	18.7
**14**	149	283	–	54.9	2.69
**15**	131	174	–	58.5	2.24
**16**	–	≥434	–	≥217	–
**17**	–	≥281	–	111	–
**18**	–	101	–	≥135	–
**19**	91.2	56.9	1.60	74.8	1.22
**20**	25.7	53.9	0.477	≥135	≤0.191
**21**	–	≥342	–	≥171	–
**22**	58.8	30.1	1.95	≥171	≥0.344
**23**	30.5	37.0	0.824	125	–
**24**	159	167	–	25.4	6.21
**25**	–	16	–	150	–
**26**	–	140	–	≥219	–
**27**	–	249	–	≥200	–
**28**	1,523	133	–	27.8	5.62
**benznidazole**	9,149	14.6	627	–	–
**chloroquine**	113	–	–	0.344	328

a) concentration that induces 50% mortality in parasites;

b) concentration that induces 50% mortality in L929 cell line;

c) selectivity index = CC_50_/IC_50_.

For the antiplasmodial assays, the compounds were tested against the *Plasmodium falciparum* clone W2‐chloroquine‐resistant.^[^ ^25^ ^]^ Compounds displaying IC_50_ values below 20 µg mL^−1^ were further assessed for cytotoxicity.^[^ ^24^ ^]^ IC_50_ and CC_50_ values expressed in molar concentrations are reported in Table 1.

### 2.4 Anti‐*Trypanosoma cruzi* Activity

The two natural compounds used as templates for the synthesis of the analogs, hypofuran B (**4**) and drynaran (**16**), were inactive against both protozoan species. Only a few compounds exhibited activity against *Trypanosoma cruzi*, and no clear structure–activity relationship could be established.

Among the compounds bearing a methoxy group at the para position of the benzene ring attached to the carbonyl‐containing B ring—considered the methoxylated derivatives of hypofuran B (**4–15**)—those carrying a halogen atom at the para position of the second aromatic ring directly bonded to the furan moiety (compounds **7** and **8**) displayed higher activity. This halogen effect, however, was not observed in compounds lacking the 4‐methoxyphenyl group (**17**–**25**), classified as drynaran derivatives.

The most active compound overall was compound **22**, featuring a thiophene ring (with a sulfur atom replacing the furan oxygen) substituted by a phenyl group at the 4‐position. In contrast, compound **21**—its constitutional isomer bearing the phenyl ring at the 5‐position—was inactive, underscoring the critical influence of the phenyl group's position on biological activity.

Comparison between compounds **12** and **22**, both containing a phenyl group at the 4‐position of the thiophene ring, revealed that the presence of a para‐methoxy substituent on the benzene ring in compound **12** led to an approximately four‐fold reduction in anti‐*Trypanosoma* activity relative to compound **22**, which lacks aromatic substituents. Nonetheless, it is important to note that the IC_50_ values of the active compounds against *T. cruzi* were very close to their CC_50_ values against L929 cells, resulting in low selectivity indices. This clearly indicates that the observed in vitro antiparasitic activity of these compounds is largely attributable to their intrinsic cytotoxicity rather than genuine trypanocidal selectivity.

### 2.5 Anti‐*Plasmodium falciparum* Activity

Regarding anti‐*Plasmodium* activity, comparison between compounds **3** (hypofuran B) and **4** revealed that substituting the hydroxyl group with a methoxy group markedly enhanced biological activity. The introduction of a second benzene ring directly attached to the furan ring in compound 5 further increased activity while reducing cytotoxicity compared with compound **4**.

In contrast, incorporation of electron‐withdrawing substituents at any position on this benzene ring (compounds **6**–**10**) diminished activity. When compound **5** was compared with compound **11**, replacement of the oxygen atom by sulfur (formation of a thiophene ring) slightly improved activity and greatly reduced cytotoxicity (CC_50_ >> 1,240 µM). A further comparison between compounds **11** and **12** indicated that the position of the benzene ring on the thiophene moiety substantially affected cytotoxic behavior.

In compound **28**, the presence of a side chain containing an *α*,*β*‐unsaturated carbonyl group produced a pronounced enhancement in activity relative to drynaran (**16**), suggesting that greater conjugation within the molecular system favorably influences anti‐*Plasmodium* properties.

Despite these observations, the IC_50_ values of the most active analogs remained considerably higher than those of benznidazole and chloroquine—the standard drugs used as positive controls for *T. cruzi* and *P. falciparum*, respectively. Notably, compound **11**, although exhibiting intermediate IC_50_ values against *P. falciparum*, displayed exceptionally low cytotoxicity—lower even than that of chloroquine.

Collectively, these findings provide a useful basis for the rational design and synthesis of new derivatives within this chemical class, aimed at developing more potent and selective antimalarial and anti‐Chagas agents.

### 2.6 Predicted Drug‐Related Physicochemical Parameters

The biological activity of a drug candidate depends not only on its intrinsic potency but also on its solubility, distribution, metabolism, and excretion (ADME) properties. These pharmacokinetic characteristics are empirically correlated with a set of physicochemical descriptors commonly summarized as Lipinski's Rule of Five.^[^ ^26^ ^]^ Such drug‐likeness criteria provide valuable predictors of whether molecules under early‐stage development possess a physicochemical profile conducive to oral bioavailability and drug‐like behavior. According to this rule, compounds that violate two or more criteria are more likely to exhibit unfavorable pharmacokinetic properties and are therefore considered poor drug candidates.^[^ ^26^ ^,^ ^27^ ^]^

To evaluate the drug‐likeness of the synthesized compounds, the following parameters were calculated in accordance with Lipinski's Rule of Five: octan‐1‐ol/water partition coefficient (LogP), number of hydrogen‐bond acceptors, number of hydrogen‐bond donors, number of rotatable bonds, molecular weight, and topological polar surface area (TPSA). The results are provided in the Supporting Infomation (Table S2). None of the compounds violated any of Lipinski's parameters, suggesting that this class of molecules may display favorable pharmacokinetic properties and good potential for further development.

## 3 Conclusion

In this study, we report for the first time the synthesis of the natural phenolic compound hypofuran B, together with a series of methoxyphenyl analogs. The compounds were prepared via base‐catalyzed cross‐aldol condensation between phenylacetaldehyde and furfural derivatives, with reaction conditions tailored according to the presence or absence of a phenolic hydroxyl group in the starting materials. Unlike the other compounds, the use of a strong base (KOH) in the synthesis of hypofuran B resulted in neutralization of the phenolic hydroxyl group, preventing the desired transformation. Successful synthesis was achieved using pyrrolidine under microwave irradiation. The reactions afforded products in yields ranging from 20% to 83%, with excellent *E*/*Z* diastereomeric ratios—from 89:11 for hypofuran B to 98:2 for the methoxylated derivatives.

The compounds, along with the natural product drynaran and several previously reported analogs, were evaluated in vitro for activity against Plasmodium falciparum and Trypanosoma cruzi, the causative agents of malaria and Chagas disease, respectively. A clear structure–activity relationship emerged, particularly for the antiplasmodial assays. Both natural products (hypofuran B and drynaran) were inactive. However, substitution of the hydroxyl group with a methoxy group on the aromatic ring, replacement of the furan ring by a thiophene ring, and variation in the position of a phenyl substituent on the five‐membered ring markedly influenced antiparasitic activity. Compounds bearing a phenyl group at the C‐4 position of the thiophene ring were the most promising, showing the lowest IC_50_ values and reduced cytotoxicity (higher CC_50_ values) in L929 cells.

Although the IC_50_ values of the most active compounds remained higher than those of benznidazole and chloroquine, used as positive controls, some compounds displayed lower cytotoxicity than chloroquine. Moreover, none of the compounds violated Lipinski's Rule of Five, suggesting favorable pharmacokinetic properties. Overall, the observed antiparasitic and cytotoxicity profiles, well‐defined structure–activity relationships, and promising drug‐likeness parameters support further investigation of hypofuran B and drynaran derivatives as potential antimalarial candidates.

## Conflict of Interest

The authors declare no conflict of interest.

## Crystallographic Data

Deposition Number 480202 (for compound 7), 2480201 (for compound 8), 2480203 (for compound 9) contain the supplementary crystallographic data for this paper. These data are provided free of charge by the joint Cambridge Crystallographic Data Centre and Fachinformationszentrum Karlsruhe Access Structures service.

## Supporting information

Supplementary Material

## Data Availability

The data that support the findings of this study are available in the supplementary material of this article.
