# Supplementary material for: Synthesis and In Vitro Activity of Hypofuran B and Analogs Against Plasmodium Falciparum and Trypanosoma Cruzi
Source: ChemMedChem. 2025 Nov 30;21(1):e202500719. doi: 10.1002/cmdc.202500719 (PMC12811995; doi:10.1002/cmdc.202500719)
Supplement: Supplementary file 1 — Supplementary Material [file CMDC-21-e202500719-s001.pdf]

Supporting Information

Synthesis and *In Vitro* Activity of Hypofuran B and Analogues Against *Plasmodium falciparum* and *Trypanosoma cruzi*

Cristiane Aparecida Franco[a], Jodieh Oliveira Santana Varejão[a], Isabela Penna Ceravolo[b], Victória Miranda Machado[b], Antoniana Ursine Krettli,[b] Daniela de Melo Resende[c], Silvane Maria Fonseca Murta[c], Felipe Terra Martins[d], Eduardo Jorge Pilau[e], Vinícius Ribeiro Montes[e], Markus Kohlhoff[f], Eduardo V V Varejão*[a]

[a] Cristiane Aparecida Franco, Jodieh Oliveira Santana Varejão, Eduardo V V Varejão
Laboratory of Natural Product Chemistry and Organic Synthesis
Department of Chemistry, Universidade Federal de Viçosa
Av. PH Rolfs, s/n, Viçosa, Brazil, 36570-900.
E-mail: eduardo.varejao@ufv.br
[b] Isabela Pena Ceravolo, Victória Miranda Machado, Antoniana Ursine Kretti
Laboratory of Immunopathology
René Rachou Institute (IRR), Fundação Oswaldo Cruz (FIOCRUZ)
Av. Augusto de Lima, 1715, Belo Horizonte, Minas Gerais, Brazil, 30190-002.
[c] Daniela de Melo Resende, Silvane Maria Fonseca Murta
Laboratory of Functional Genomics of Parasites
René Rachou Institute (IRR), Fundação Oswaldo Cruz (FIOCRUZ)
Av. Augusto de Lima, 1715, Belo Horizonte, Minas Gerais, Brazil, 30190-002.
[d] Felipe Terra Martins, Aluno do Felipe
Institute of Chemistry
Universidade Federal de Goiás
Av Esperança, sn, Samambaia, Goiânia, Brazil, 74.690-900.
[e] Eduardo Jorge Pilau, Vinícius Ribeiro Montes (UEL)
Laboratory of Biomolecules and Mass Spectrometry
Universidade Estadual de Maringá, Paraná, Brazil, 87020-900.
[f] Laboratory of Bioactive Natural Product Chemistry, René Rachou Institute (IRR), Fundação Oswaldo Cruz (FIOCRUZ), Av. Augusto de Lima, 1715, Belo Horizonte, Minas Gerais, Brazil, 30190-002.

1. Material And Methods

1.1. General Chemical Procedures

All reagents were purchased from commercially available suppliers and used without any further purification. All solvents used were of analytical grade and were distilled before use. Analytical thin-layer chromatography (TLC) analysis was conducted on aluminum-packed pre-coated silica gel 60 G F254TLC plates (Macherey-Nagel, 0.25 mm, Düren, Germany). Column chromatography was performed using silica gel 60 (230-400 Mesh) as stationary phase and hexane/ethyl acetate mixtures as mobile phases. Gas chromatography-mass spectrometry (GC-MS) analysis was carried outin a Shimadzu GCMS-QP5050A apparatus (Shimadzu Europe, Duisburg, Germany) equipped with capillary column RTx5 (30 m x 0.25 mm, 0.25 µm) by electron impact method (70 eV). Melting temperature (M.p.) ranges were determined in Microchemical MQAPF-302 apparatus (MicroquímicaEquipamentos, Palhoça, Santa Catarina, Brazil). ^{1}H and ^{13}C Nuclear Magnetic Resonance (NMR) experiments were recorded on a Varian Mercury 500 instrument (Varian, Palo Alto, California, USA) at 500-300 MHz and 126-75 MHz, respectively, using $CDCl_3$, acetone-d6 or DMSO-d6 as the solvents, and their chemical shifts were used as reference. Coupling constants (*J*) are given in Hertz. High-resolution mass spectrometry (HRMS) measurements were conducted using electrospray ionization (Bruker Daltonics Corporation, Q-TOF geometry Impact II). Reactions that occur at MW as a heating source were carried out in a sealed 10 mL Pyrex glass tube using a CEM Discovery microwave reactor, with temperature monitoring performed via an internal probe.

1.2. Synthetic Procedures

Synthesis of 2-(4-methoxyphenyl)etanol (1)

A mixture of 3 mmol of tyrosol, 12 mmol of potassium carbonate (K_2CO_3), and 10 mL of anhydrous acetone was transferred to a round-bottom flask under a nitrogen atmosphere and stirred magnetically. Subsequently, 12 mmol of methyl iodide was added, and the reaction mixture was stirred for 24 hours at 50 °C. After completion (monitored by TLC), the reaction mixture was filtered and the solvent was removed under reduced pressure. The residue was dissolved in 15 mL of water and extracted with dichloromethane (3 × 20 mL). The combined organic layers were concentrated to afford the crude product, whose purity was confirmed by GC-MS analysis, making further purification steps unnecessary.[25]
Yellow oil, 92% yield. MS (EI, 70 eV), *m/z* (%): 121 (100%), 152 (17%).

Synthesis of 2-(4-Methoxyphenyl)acetaldehyde (2)

Compound (2) was obtained from (1) via Swern oxidation. To perform the reaction, a solution of 2 mmol of oxalyl chloride in dichloromethane (DCM) was transferred to a round-bottom flask under a nitrogen atmosphere. The system was cooled to −78 °C using a liquid nitrogen bath, and 300 µL of dimethyl sulfoxide (DMSO) were added dropwise. After 20 minutes, a solution containing 1 mmol of 2-(4-methoxyphenyl)ethanol in 10 mL of DCM and 5 mmol of triethylamine was added. The reaction mixture was stirred at room temperature for 6 hours. After completion, the mixture was washed with 10 mL of 1 mol L^{-1} hydrochloric acid solution, and the organic layer was concentrated under reduced pressure. The product was obtained without any further purification, and its identity and purity were confirmed by GC-MS analysis.[26]
Dark oil, 60% yield. MS (IE, 70 eV), *m/z* (%): 107 (10%), 150 (14%).

Synthesis of Hypofuran B (3-(5-(hydroxymethyl)furan-2-yl)-2-4−hydroxyphenyl)acrylaldehyde) (4)

A mixture of 1 mmol of compound (2), 1 mmol of 5-hydroxymethyl-2-furaldehyde, 30 mol% of pyrrolidine, and 1 mL of ethanol was transferred to a reaction tube and subjected to microwave irradiation at 150 W and 120 °C for 15 minutes.[27] The resulting compound was purified by column chromatography.
Brown oil, 66% yield, diastereoisomeric ratio (*E/Z*) 89:11. MS (IE, 70 eV), *m/z*(%): 213 (100%), 244 (26%).^{1}H NMR (400 MHz, Acetone-d6) δ 9.49 (s, 1H, H-1'), 7.13 (s, 1H, H-3'), 6.89 (d, J = 8.6 Hz, 2H, H-8.8'), 6.74 (d, J = 8.6 Hz, 2H, H-9.9'), 6.16 (d, J = 3.5 Hz, 1H, H-3), 6.06 (d, J = 3.5 Hz, 1H, H-4), 4.33 (s, 2H, H-6). ^{13}C NMR (101 MHz, Acetone-d6) δ 192.38(C-1'), 158.87(C-10), 157.54(C-5), 150.27(C-2), 138.50(C-2'), 135.46(C-3'), 130.58(C-8.8'), 124.55(C-1'), 116.71(C-4), 115.30(C-9.9'), 109.81(C-3), 56.50(C-6). HRMS (ESI) *m/z*, calcd. for C14H12O4 [M + Na+]: 267.0627, found: 267.0623.

Synthesis of 4-methoxyphenyl analogues of Hypofuran B (3,5-15)

4-methoxyphenyl derivatives of hypofuran B were synthesized by reacting (4-methoxyphenyl)acetaldehyde (2) with a series for furaldehydes. To this, 1 mmol of furfural derivative, 0.5 mmol of potassium hydroxide, 0.1 mmol of sodium dithionite ($Na_2S_2O_4$), and 2 mL of ethanol was transferred to a reaction vessel. The mixture was stirred magnetically and heated to 70 °C. Subsequently, a solution of 1 mmol of 2-(4-methoxyphenyl)acetaldehyde (2) in 2 mL of ethanol was added dropwise. After stirring at 70 °C for 1 hour, the reaction mixture was concentrated under reduced pressure, and the crude product was purified by silica gel column chromatography using mixtures of hexane and ethyl acetate as eluents

3-(5-(hydroxymethyl)furan-2-yl)-2-(4-methoxyphenyl)acrylaldehyde (3)

Dark oil, 33% yield, 93:7 (*E/Z*) diastereomeric ratio. MS (EI, 70 eV), *m/z* (%): 44 (39%), 128 (48%), 227 (100%), 258 (36%). ^{1}H NMR (400 MHz, $CDCl_3$) δ: 9.66 (s, 1H, H-1′), 7.23–7.16 (m, 3H, H-8, 8′, and 3′), 7.02–6.97 (m, 2H, H-9, 9′), 6.28 (s, 1H, H-4), 6.23 (s, 1H, H-3), 4.57 (s, 2H, H-6), 3.87 (s, 3H, H-11). ^{13}C NMR (101 MHz, $CDCl_3$) δ: 193.01 (C-1′), 159.85 (C-10), 157.25 (C-5), 150.62 (C-2), 138.29 (C-2′), 136.12 (C-3′), 130.59 (C-8, 8′), 125.35 (C-7), 117.31 (C-3), 114.28 (C-9, 9′), 110.61 (C-4), 57.61 (C-6), 55.41 (C-11). HRMS (ESI) *m/z*, calcd. for $C_{15}H_{14}O_4$ $[M+H]^+$: 259.0964, found: 259.0955.

2-(4-methoxyphenyl)-3-(5-phenylfuran-2-yl)acrylaldehyde (5)

Dark oil, 47% yield, diastereoisomeric ratio 88:22 *E/Z*. MS (IE, 70 eV), *m/z* (%): 77 (30%), 105 (100%), 144 (23%), 304 (35%). ^{1}H NMR (300 MHz, Chloroform-d) δ 9.70 (s, 1H, H-1'), 7.48 – 7.43 (m, 2H, H-7, 7'), 7.39 – 7.28 (m, 3H, H-8, 8' e 9), 7.25 (d, J = 8.4 Hz, 2H, H-11, 11'), 7.22 (s, 1H, H-3'), 7.02 (d, J = 8.7 Hz, 2H, H-12, 12'), 6.66 (d, J = 3.7 Hz, 1H, H-4), 6.52 (d, J = 3.7 Hz, 1H, H-3), 3.88 (s, 3H, H-14). ^{13}C NMR (75 MHz, Chloroform-d) δ 192.94 (C-1'), 159.82 (C-13), 156.98 (C-5), 150.26 (C-2), 137.58 (C-2'), 135.22 (C-3'), 130.66 (C-11, 11'), 129.58 (C-10), 128.89 (C-8, 8'), 128.76 (C-6), 125.89 (C-9), 124.50 (C-7, 7'), 119.75 (C-3), 114.21 (C-12, 12'), 108.14 (C-4), 55.48 (C-14). HRMS (ESI) *m/z*, calcd. for $C_{20}H_{15}NO_2O_3$ $[M+H]^+$: 305.1172, found: 305.1165.

2-(4-methoxyphenyl)-3-[5-(2-nitrophenyl)furan-2-yl]acrylaldehyde (6)

Orange solid, 58% yield, diastereoisomeric ratio (*E/Z*) 92:8. MS (IE, 70 eV), *m/z* (%): 132 (56%), 202 (100%), 319 (35%), 349 (22%). ^{1}H NMR (400 MHz, Chloroform -d) δ 9.70 (s, 1H, H-1'), 7.70 (d, J = 6.8 Hz, 1H, H-8), 7.52 (dd, J = 7.2, 3.2 Hz, 1H, H-11), 7.45 (d, J = 7.3 Hz, 2H, H-9, 10), 7.19 (s, 3H, H-13.13' e 3'), 7.04 – 6.98 (m, 2H, H-14,14'), 6.63 (s, 1H, H-4), 6.43 (s, 1H, H-3), 3.86 (s, 3H, H-16). ^{13}C NMR (101 MHz, Chloroform-d) δ 192.89 (C-1'), 159.95 (C-15), 151.67 (C-5), 150.74 (C-2), 147.53 (C-7), 139.17 (C-2'), 134.81 (C-3'), 132.17 (C-11), 130.51 (C-13.13'), 129.29 (C-9), 129.20 (C-10), 125.37 (C-12), 124.24 (C-8), 123.16 (C-6), 118.63 (C-3), 114.38 (C-14.14'), 112.68 (C-4), 55.46 (C-16). HRMS (ESI) *m/z*, calcd. for $C_{20}H_{15}NO_2O_3$ $[M+H]^+$: 350.1023, found: 350.1019.

3-[5-(4-bromophenyl)furan-2-yl]-2-(4-methoxyphenyl)acrylaldehyde (7)

Orange solid, 68% yield, diastereoisomeric ratio (*E/Z*) 88:12. MS (IE, 70 eV), *m/z* (%): 44 (100%), 183 (85%), 185 (82%). 382 (49%). 385 (50%). ^{1}H NMR (400 MHz. Chloroform-d) δ 9.72 (s. 1H. H-1'). 7.49 – 7.45 (m. 2H. H-8, 8'), 7.33 – 7.29 (m, 2H, H-7, 7'), 7.25 (dd, J = 7.1, 1.9 Hz, 2H, H-12, 12'), 7.22 (s, 1H, H-3'), 7.05 – 7.01 (m, 2H, H-13, 13'), 6.68 (s, 1H, H-4), 6.54 (s, 1H, H-3), 3.90 (s, 3H, H-15). ^{13}C NMR (101 MHz, Chloroform-d) δ 192.89 (C-1'), 159.87 (C-14), 155.78 (C-2), 150.54 (C-5), 137.95 (C-2'), 134.84 (C-3'), 132.09 (C-8.8'), 130.63 (C-12, 12'), 128.49 (C-6), 125.90 (C-7.7'), 122.74 (C-9), 119.68 (C-3), 114.19 (C-13.13'), 108.55 (C-4), 55.52 (C-15). HRMS (ESI) *m/z*, calcd. for $C_{20}H_{15}BrO_3$ [M + Na+]: 405.0096, found: 405.0092.

3-[5-(4-chlorophenyl)furan-2-yl]-2-(4-methoxyphenyl)acrylaldehyde (8)

Orange solid, 62% yield, diastereoisomeric ratio (*E/Z*) 88:12. MS (IE, 70 eV), *m/z* (%): 139 (100%), 141 (33%), 338 (31%), 340 (10%). ^{1}H NMR (400 MHz, Chloroform-d) δ 9.73 (s, 1H, H-1'), 7.40 – 7.35 (m, 2H, H-8, 8'), 7.35 – 7.30 (m, 2H, H-7, 7'), 7.28 – 7.24 (m, 1H, H-12, 12'), 7.22 (s, 1H, H-3'), 7.07 – 7.02 (m, 2H, H-13, 13'), 6.67 (s, 1H, H-4), 6.55 (s, 1H, H-3), 3.91 (s, 3H, H-15). ^{13}C NMR (101 MHz, Chloroform-d) δ 192.78 (C-1'), 159.76 (C-14), 155.66 (C-2), 150.38 (C-5), 137.79 (C-2'), 134.75 (C-3'), 134.42 (C-11), 130.53 (C-12, 12'), 129.04 (C-7, 7'), 127.97 (C-9), 125.71 (C-6), 125.57 (C-8, 8'), 119.58 (C-3), 114.07 (C-13, 13'), 108.34 (C-4), 55.40 (C-15). HRMS (ESI) *m/z*, calcd. for $C_{20}H_{15}ClO_3$ [M + Na+]: 361.0602, found: 361.0598.

3-[5-(3-chlorophenyl)furan-2-yl]-2-(4-methoxyphenyl)acrylaldehyde (9)

Yellow solid, 55% yield, diastereoisomeric ratio (*E/Z*). MS (IE, 70 eV), *m/z* (%): 44 (32%), 139 (100%), 141 (32%), 338 (20%), 340 (6%). ^{1}H NMR (400 MHz, Chloroform-d) δ 9.71 (s, 1H, H-1'), 7.34 (s, 1H, H-11), 7.32 (s, 1H, H-7), 7.26 – 7.21 (m, 4H, H-9, 8, 13, 13'), 7.19 (s, 1H, H-3'), 7.05 – 6.99 (m, 2H, H-14, 14'), 6.68 (s, 1H, H-3), 6.56 (s, 1H, H-4), 3.88 (s, 3H, H-16). ^{13}C NMR (101 MHz, Chloroform-d) δ 192.96 (C-1'), 159.92 (C-15), 155.28 (C-2), 150.70 (C-5), 138.12 (C-2'), 135.05 (C-3'), 134.57 (C-12), 131.22 (C-10), 130.62 (C-13, 13'), 130.14 (C-6), 128.57 (C-8), 125.66 (C-9), 124.45 (C-7), 122.47 (C-11), 119.69 (C-4), 114.16 (C-14, 14'), 108.93 (C-3), 55.43 (C-16). HRMS (ESI) *m/z*, calcd. for $C_{20}H_{15}ClO_3$ $[M-H]^{+}$: 339.0782, found: 339.0774.

3-[5-(2-chlorophenyl)furan-2-yl]-2-(4-methoxyphenyl)acrylaldehyde (10)

Orange solid, 79% yield, diastereoisomeric ratio (*E/Z*)96:4. MS (IE, 70 eV), *m/z* (%): 44 (100%), 139 (84%), 141 (29%), 338 (22%), 340 (7%). ^{1}H NMR (400 MHz, Chloroform-d) δ 9.71 (s, 1H, H-1'), 7.43 – 7.32 (m, 2H, H- 9, 11), 7.18-7.24 (m, 6H, H- 4, H-3', 8, 10, 13, 13'), 7.09 – 6.98 (m, 2H, H-14, 14'), 6.57 (s, 1H, H-3), 3.88 (s, 3H, H-16). ^{13}C NMR (101 MHz, Chloroform-d) δ 192.85 (C-1'), 159.77 (C-15), 152.96 (C-2), 149.89 (C-5), 138.25 (C-2'), 134.76 (C-3'), 130.83 (C-11), 130.61 (C-6), 130.47 (C-13, 13'), 129.09 (C-12), 128.27 (C-9), 127.90 (C-7), 126.88 (C-8), 125.75 (C-10), 119.37 (C-3), 114.14 (C-14, 14'), 113.72 (C-4), 55.37 (C-16). HRMS (ESI) *m/z*, calcd. for $C_{20}H_{15}ClO_3$ $[M-H]^{+}$: 339.0782, found: 339.0774.

2-(4-methoxyphenyl)-3-(5-phenylthiophen-2-yl)acrylaldehyde (11)

Orange solid, 40% yield, diastereoisomeric ratio (*E/Z*) 96:4. MS (IE, 70 eV), *m/z* (%): 44 (100%), 160 (21%), 320 (16%). ^{1}H NMR (400 MHz, Chloroform-d) δ 9.70 (s, 1H, H-1'), 7.53 (s, 1H, H-3'), 7.48 – 7.43 (m, 2H, H-7, 7'), 7.38 – 7.24 (m, 4H, H-4, 8, 8', 9), 7.23 – 7.15 (m, 3H, H-3, 11, 11'), 7.07 – 7.00 (m, 2H, H-12, 12'), 3.88 (s, 3H, H-14). ^{13}C NMR (101 MHz, Chloroform-d) δ 193.03 (C-1'), 160.19 (C-13), 150.67 (C-5), 142.33 (C-3'), 138.68 (C-2'), 137.52 (C-2), 135.56 (C-10), 133.46 (C-6), 131.01 (C-11, 11'), 128.99 (C-8, 8'), 128.60 (C-9), 126.10 (C-7, 7'), 124.76 (C-4), 123.33 (C-3), 114.75 (C-12, 12'), 55.36 (C-14). HRMS (ESI) *m/z*, calcd. for $C_{20}H_{16}O_2S$ $[M+H]^{+}$: 321.0943, found: 321.0935.

2-(4-methoxyphenyl)-3-(4-phenylthiophen-2-yl)acrylaldehyde (12)

Yellow solid, 40% yield, diastereoisomeric ratio (*E/Z*) 97:3. MS (IE, 70 eV), *m/z* (%): 44 (100%), 132 (28%), 160 (28%), 173 (31%), 320 (45%). ^{1}H NMR (400 MHz, Chloroform-d) δ 9.81 – 9.73

(m, 1H, H-1'), 7.64 (s, 1H, H-3'), 7.60 (s, 1H, H-5), 7.57 – 7.50 (m, 2H, H-7, 7'), 7.47 (d, J = 2.4 Hz, 1H, H-3), 7.42 (t, J = 7.4 Hz, 2H, H-8, 8'), 7.35 (d, J = 7.4 Hz, 1H, H-9), 7.20 (dd, J = 7.7, 2.3 Hz, 2H, H-11, 11'), 7.06 (dd, J = 7.8, 2.4 Hz, 2H, H-12, 12'), 3.93 – 3.78 (m, 3H, H-14). ^{13}C NMR (101 MHz, Chloroform-d) *δ* 193.05 (C-1'), 160.29 (C-13), 142.29 (C-2), 142.21 (C-3'), 139.55 (C-2'), 138.97 (C-4), 134.74 (C-6), 132.97 (C-10), 131.02 (C-5), 128.98 (C-11, 11'), 127.71 (C-8, 8'), 126.85 (C-3), 126.26 (C-3), 124.56 (C-7, 7'), 114.80 (C-12, 12'), 55.33 (C-14). HRMS (ESI) *m/z*, calcd. for $C_{20}H_{16}O_2S$ $[M+H]^+$: 321.0943, found: 321.0937.

3-(4,5-dimethylfuran-2-yl)-2-(4-methoxyphenyl)acrylaldehyde (13)
Orange oil, 20% yield, diastereoisomeric ratio (*E/Z*) 94:6. MS (IE, 70 eV), *m/z* (%): 43 (100%), 96 (40%), 132 (44%), 214 (42%), 256 (67%). ^{1}H NMR (400 MHz, Chloroform-d) *δ* 9.59 (s, 1H, H-1'), 7.19 – 7.14 (m, 2H, H-9, 9'), 7.11 (s, 1H, H-3'), 6.97 (d, J = 9.4 Hz, 2H, H-10, 10'), 6.07 (s, 1H, H-3), 3.86 (s, 3H, H-12), 2.18 (s, 3H, H-6), 1.84 (s, 3H, H-7). ^{13}C NMR (101 MHz, Chloroform-d) *δ* 192.89 (C-1'), 159.51 (C-11), 158.04 (C-5), 152.44 (C-2), 148.09 (C-8), 136.58 (C-3'), 136.17 (C-2'), 130.52 (C-9, 9'), 125.70 (C-4), 118.39 (C-3), 114.12 (C-10, 10'), 55.29 (C-12), 11.75 (C-6), 9.71 (C-7). HRMS (ESI) *m/z*, calcd. for $C_{16}H_{16}O_3$ $[M+H]^+$: 257.1172, found: 257.1163.

3-(5-ethylfuran-2-yl)-2-(4-methoxyphenyl)acrylaldehyde (14)
Orange oil, 40% yield, diastereoisomeric ratio (*E/Z*) 93:7. MS (IE, 70 eV), *m/z* (%): 44 (100%), 200 (26%), 227 (36%), 256 (39%). ^{1}H NMR (400 MHz, Chloroform-d) *δ* 9.63 (s, 1H, H-1'), 7.21 – 7.14 (m, 3H, H-3', 9, 9'), 7.00 – 6.95 (m, 2H, H-10, 10'), 6.23 (s, 1H, H-4), 6.00 (s, 1H, H-3), 3.85 (s, 3H, H-12), 2.60 (dd, J = 7.7, 2.8 Hz, 3H, H-6), 1.17 (ddd, J = 7.9, 5.6, 2.3 Hz, 3H, H-7). ^{13}C NMR (101 MHz, Chloroform-d) *δ* 193.01 (C-1'), 161.96 (C-11), 159.66 (C-5), 149.39 (C-2), 136.78 (C-2'), 136.48 (C-3'), 130.67 (C-9, 9'), 125.76 (C-8), 118.24 (C-4), 114.18 (C-10, 10'), 108.14 (C-3), 55.40 (C-12), 21.74 (C-6), 11.89 (C-7). HRMS (ESI) *m/z*, calcd. for $C_{16}H_{16}O_3$ $[M+H]^+$: 257.1172, found: 257.1164.

2-(4-methoxyphenyl)-3-(5-methylfuran-2-yl)acrylaldehyde (15)
Orange oil, 40% yield, diastereoisomeric ratio (*E/Z*) 92:8. MS (IE, 70 eV), *m/z* (%): 44(100%), 227(32%), 242 (41%). ^{1}H NMR (400 MHz, Chloroform-d) *δ* 9.62 (s, 1H, H-1'), 7.18 – 7.15 (m, 3H, H-3', 8, 8'), 7.03 – 6.94 (m, 2H, H-9, 9'), 6.18 (s, 1H, H-4), 5.99 (s, 1H, H-3), 3.84 (s, 1H, H-11), 2.27 (s, 1H, H-6). ^{13}C NMR (101 MHz, Chloroform-d) *δ* 192.87 (C-1'), 159.58 (C-10), 156.29 (C-5), 149.40 (C-2), 136.73 (C-2'), 136.43 (C-3'), 130.50 (C-8, 8'), 125.58 (C-7), 118.09 (C-4), 114.13 (C-9, 9'), 109.58 (C-3), 55.28 (C-11), 13.85 (C-6). HRMS (ESI) *m/z*, calcd. for $C_{15}H_{14}O_3$ [M]+: 243.1015, found: 243.1011.

Synthesis of Drynaran and Analogues (16-26)
The synthesis of drynaran (**16**) and its analogues (**17-26**), as well as their spectroscopic and spectrometric data, were reported in a previous study.[17] The compounds were obtained using the same methodology described above for the synthesis of the 4-methoxyphenyl analogues of hypofuran B.

Synthesis of Drynaran derivatives (27-28)

3-(5-(methoxymethyl)furan-2-yl)-2-phenylacrylaldehyde (27)
Drynaram (1 mmol), KOH (1 mmol), and methyl iodide (1.5 mmol) were added to the reaction flask under a nitrogen atmosphere. The reaction mixture was stirred at room temperature for 24 hours and then subjected to column chromatography.
Yellow oil, 83% yield, diastereoisomeric ratio (*E/Z*) 94:6. MS (EI, 70 eV), *m/z* (%): 44 (100%), 197 (85%), 242 (14%). ^{1}H NMR (400 MHz, Acetone-d$_6$) *δ* 9.72 (s, 1H, H-1'), 7.47–7.40 (m, 4H, H-3', 10, 10', and 11), 7.25–7.20 (m, 2H, H-9, 9'), 6.41 (dd, J = 3.6, 0.6 Hz, 1H, H-3), 6.22 (d, J = 3.5 Hz, 1H, H-34), 4.30 (s, 2H, H-6), 3.23 (s, 3H, H-7).^{13}C NMR (101 MHz, Acetone-d$_6$) *δ* 192.15 (C-1'), 155.70 (C-5), 150.60 (C-2), 138.92 (C-2'), 135.54 (C-3'), 133.99 (C-8), 129.26 (C-9, 9'), 128.38

(C-10, 10'), 128.07 (C-11), 117.19 (C-4), 111.89 (C-3), 65.65 (C-6), 57.27 (C-7). HRMS (ESI) *m/z*, calcd. for $C_{15}H_{14}O_3$ [M + Na]$^+$: 265.0835, found: 265.0829.

Synthesis of 6-(5-(Hydroxymethyl)furan-2-yl)-5-phenylhexa-3,5-dien-2-one (28)

Drynaram (1 mmol), KOH (1 mmol) and acetone (1 mL) were transferred to a reaction flask, and the mixture was stirred at room temperature for 1 hour. The acetone was then evaporated under reduced pressure, and the residue was subjected to column chromatography.

Yellow oil, 77% yield, diastereoisomeric ratio (*E/Z*) 94:6. MS (EI, 70 eV), *m/z* (%): 44 (100%), 207 (44%), 268 (57%). ^{1}H NMR (400 MHz, $CDCl_3$) *δ* 7.50–7.39 (m, 4H, H-4', 8, 8', and 10), 7.18–7.10 (m, 2H, H-9, 9'), 6.82 (s, 1H, H-6'), 6.10 (d, J = 3.4 Hz, 1H, H-3), 5.73 (d, J = 15.5 Hz, 1H, H-3'), 5.44 (d, J = 3.5 Hz, 1H, H-4), 4.48 (s, 2H, H-6), 2.24 (s, 3H, H-1'). ^{13}C NMR (101 MHz, $CDCl_3$) *δ* 198.49 (C-2'), 154.93 (C-5), 151.88 (C-2), 146.91 (C-4'), 137.94 (C-5'), 136.96 (C-7), 129.30 (C-9, 9'), 129.04 (C-3'), 128.92 (C-8, 8'), 128.20 (C-10), 126.89 (C-6'), 113.74 (C-4), 110.25 (C-3), 57.56 (C-6), 28.07 (C-1'). HRMS (ESI) *m/z*, calcd. for $C_{17}H_{16}O_3$ [M + Na]$^+$: 291.0991, found: 291.0981.

1.3. Crystal structure elucidation

Suitably shaped single crystals of **7**-**9** were selected and exposed to the X-ray beam on an XtaLAB Rigaku diffractometer (Synergy, Dualflex, HyPix). The crystals were kept at a steady T = 100.00(10) K during data collection. The structures were solved with the ShelXT 2018/2 (Sheldrick, 2018) structure solution program using the Intrinsic Phasing solution method using Olex2 (Dolomanov et al., *J. Appl. Cryst.*, (2009), **42**, 339-341) as the graphical interface. The model was refined with version 2019/2 of ShelXL 2019/2 (Sheldrick, *Acta Crystallogr. Sect. C,* (2015), 71, 3–8) using full-matrix least squares minimisation. MERCURY (Macrae et al., *J. Appl. Cryst.*, (2020), **53**, 226-235) was chosen to prepare the crystal structure projections. The entire X-ray diffraction datasets, loading all structure factors and RES refinement file, were deposited in CCDC under deposit code shown in Table S1.

Table 1. Crystal data and refinement statistics for the three compounds elucidated here.

	**7**	**8**	**9**
Formula	$C_{20}H_{15}BrO_3$	$C_{20}H_{15}ClO_3$	$C_{20}H_{15}ClO_3$
$D_{calc.}$/ g cm^{-3}	1.510	1.356	1.379
m/mm^{-1}	3.445	2.160	2.197
Formula Weight	383.23	338.77	338.77
T/K	100(2)	100(2)	100(2)
Crystal System	monoclinic	monoclinic	monoclinic
Space Group	$P2_1/n$	$P2_1/c$	$P2_1/n$
a/Å	9.52210(10)	10.3907(3)	10.34940(10)
b/Å	18.1787(2)	9.14470(10)	12.05200(10)
c/Å	10.31500(10)	22.1446(6)	13.27410(10)
a/°	90	90	90
b/°	109.2330(10)	127.954(4)	99.8400(10)
g/°	90	90	90
V/Å^3	1685.86(3)	1659.15(10)	1631.33(2)
Z	4	4	4
Z'	1	1	1
Wavelength/Å	1.54184	1.54184	1.54184
Radiation type	CuK$_a$	Cu K$_a$	CuK$_a$
Q_{min}/°	4.865	4.600	4.990
Q_{max}/°	79.271	79.315	79.353
Measured Refl.	18845	18987	18341
Independent Refl.	3650	3606	3526
Reflections with I > s2(I)	3445	3353	3238
R_{int}	0.0309	0.0355	0.0312
Parameters	218	218	218
Restraints	0	0	0
Largest Peak	0.334	0.245	0.247
Deepest Hole	-0.444	-0.294	-0.323
GooF	1.096	1.092	1.079
wR_2 (all data)	0.0777	0.0901	0.0865
wR_2 (I > s2(I))	0.0756	0.0880	0.0836
R_1 (all data)	0.0314	0.0371	0.0358
R_1 (I > s2(I))	0.0287	0.0343	0.0326
CCDC deposit number	2480202	2480201	2480203

1.4. Anti-*Trypanosoma cruzi* activity assay

he *in vitro* anti-*T. cruzi* activity was evaluated on L929 cells (mouse fibroblasts) infected with Tulahuen strain of the parasite expressing the *Escherichia coli* β-galactosidase as reporter gene according to the method described previously.[28-29] Briefly, four thousand L929 cells were added to each well of a 96-well microtiter plate. After an overnight incubation, a 10-fold excess of trypomastigotes were added to the cells, incubated for 2 h, then replaced with 200 µL of fresh medium and incubated for an additional 48 h to establish the infection. Then, medium was replaced, and test compounds were added at six different concentrations using serial dilutions, starting at 100 µg mL^{-1}. Compounds that produced 50% of *T. cruzi* growth inhibition in at least 30 µg mL^{-1} were selected to determine the IC_{50}. To this, cells were exposed to each synthesized compound at serial decreasing dilutions (100 to 1.56 µg mL^{-1}) and the plate was incubated for 96 h. Afterwards, 50 µl of 500 µM chlorophenol red beta-*D*-galactopyranoside (CPRG) in 0.5% Nonidet P40 was added to each well, and the plate incubated for 16 to 20 h, after which the absorbance at 570 nm was measured. Controls with uninfected cells, untreated infected cells,

infected cells treated with benznidazole at 3.8 μM (positive control) or 1% DMSO were used. The results were expressed as the percentage of *T. cruzi* growth inhibition in compound tested cells as compared to the infected cells and untreated cells. The IC_{50} values were calculated by linear interpolation. Quadruplicates were run on the same plate, and the experiments were repeated at least once.[30-31]

1.5. Anti- *P. falciparum* activity assay

Compounds were tested against the chloroquine-resistant *Plasmodium falciparum* W2 clone[22], cultured as previously described. [29-30] Briefly, the compounds were tested against the parasite erythrocytic asexual stages using a chloroquine-resistant and mefloquine-sensitive W2 clone cultured at 37°C. The activity was measured using the SYBR assay with the parasite suspension (0.5% parasitemia and 2% hematocrit) [34] with minor modifications [31]. The test compounds, in serial dilutions, were incubated in "U" bottom 96-wells plates. After 48 h at 37°C, the culture supernatant was removed and replaced by 100 μl of lysis buffer solution [Tris (20 mM; pH 7.5), EDTA (5 mM), saponin (0.008%; wt/vol), and Triton X 100 (0.08%; vol/vol)] followed by addition of 0.2 μl/ml SYBR Safe (Sigma-Aldrich, Carlsbad, CA, USA). The plate content was transferred to a flat bottom plate and incubated in the dark for 30 min. The plate was read in a fluorometer (Synergy H4 Hibrid Reader, Biotek) with excitation at 485 nm and 535 nm of emission. In all tests, the compound activities were expressed by the 50% inhibitory concentration of the parasite growth (IC_{50}) when compared to the drug-free controls and estimated using the curve-fitting software Origin 8.0 (OriginLab Corporation, Northampton, MA, USA). Compounds with IC_{50} greater than 20 μg/ml were classified as not active, as partially active when 10 μg/ml < IC_{50} < 20 μg/ml and less than 10 μg/ml were considered as active. Chloroquine was used as an anti-malarial reference drug in all tests performed.

1.6. Cytotoxicity assay

For the *in vitro* cytotoxic activity test and CC_{50} determination over the L929 cell line, 4.000 L929 cells in 200 μL of RPMI-1640 medium (pH 7.2-7.4) (Gibco BRL) plus 10% fetal bovine serum and 2 mM glutamine were added to each well of a 96-well microtiter plate that was incubated for three days at 37°C. The medium was then replaced, and the cells were exposed to compounds at increasing concentrations starting at IC_{50} value for *T. cruzi*. After 96 h of incubation with the compounds the alamarBlueTM was added and the absorbance at 570 and 600 nm was measured after 4-6 h. Controls with untreated and DMSO 1%-treated cells were run in parallel. The results were expressed as the percentage difference in the reduction between treated and untreated cells. The compound concentration that inhibits 50% of the L929 cell viability (CC_{50}) was determined by linear interpolation and the selectivity index (SI) was calculated by the ratio of CC_{50} L929 cells/ IC_{50} *T. cruzi* or *P. falciparum*. Quadruplicates were run on the same plate, and the experiments were repeated at least once.[29-30]

1.7. Drug-likeness parameters

Computational calculation was carried out to assess whether the investigated compounds can fulfil the features of candidate drugs, based on Lipinski's Rule of Five.[25- 26] Physicochemical parameters such as n-octanol/water partition coefficient (CLogP), number of hydrogen bond donors (HBD), number of hydrogen bond acceptors (HBA), molecular weight (MW), number of rotable bonds (nRotb), and total polar surface area (TPSA) were calculated using Swissadme software package (http://www.swissadme.ch/index.php#).

Table S2. In silico-calculated physicochemical parameters for the selected compounds, based on Lipinski's Rule of Five.

Compound	MW[a]	ClogP[b]	HBA[c]	ROB[d]	HBD[e]	TPSA[f]	Number of rule violations
	≤500	≤4.15	≤10	≤9	≤5	≤130	
**4**	258.27	2.26	4	5	1	5967	0
**5**	304.35	3.05	3	5	0	39.44	0
**6**	349.34	2.70	5	5	0	85.26	0
**7**	383.24	3.55	3	5	0	39.44	0
**8**	338.79	3.45	3	5	0	39.44	0
**9**	338.79	3.5	3	5	0	39.44	0
**10**	338.79	3.34	3	5	0	39.44	0
**11**	320.41	3.42	2	5	0	54.54	0
**12**	320.41	3.18	2	5	0	54.54	0
**13**	256.30	2.92	3	4	0	39.44	0
**14**	256.30	2.87	3	3	0	39.44	0
**15**	242.27	2.73	3	3	0	39.44	0
**16**	244.24	1.82	4	4	2	70.67	0
**17**	242.27	2.54	3	5	0	39.44	0
**18**	268.31	2.54	3	5	1	50.44	0
**19**	228.24	2.19	3	4	1	50.44	0
**20**	353.21	3.28	2	4	0	30.21	0
**21**	308.76	3.12	2	4	0	30.21	0
**22**	308.76	3.16	2	4	0	30.21	0
**23**	308.76	3.06	2	4	0	30.21	0
**24**	290.38	2.98	1	4	0	45.31	0
**25**	290.38	3.02	1	4	0	79.03	0
**26**	319.31	2.41	4	5	0	54.54	0
**27**	226.27	2.54	2	3	0	30.21	0
**28**	212.24	2.43	2	3	0	39.44	0
**29**	226.27	2.62	2	4	0	30.21	0

[a] Molecular weight. [b] partition coefficient prediction. [c] number of hydrogen bonding acceptors. [d] number of rotatable bonds. [e] number of hydrogen bonding donor groups. [f] topological polar surface.

2. Mass and NMR Spectra of Compounds 3-15, 27 and 28.

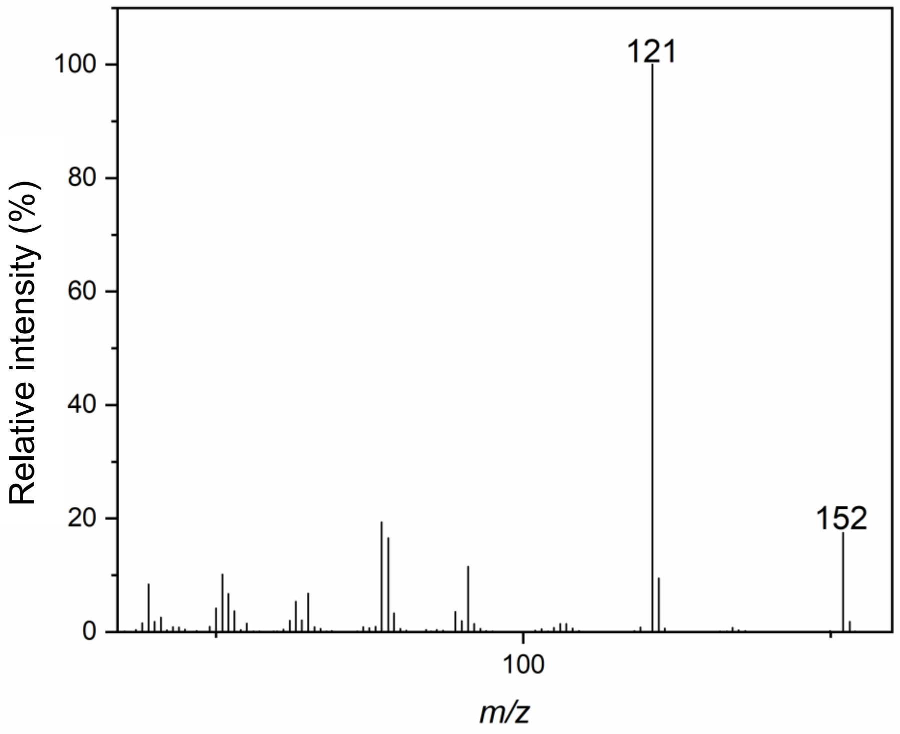

Figure S1: Mass Spectrum(IE, 70 eV) of compound **1.**

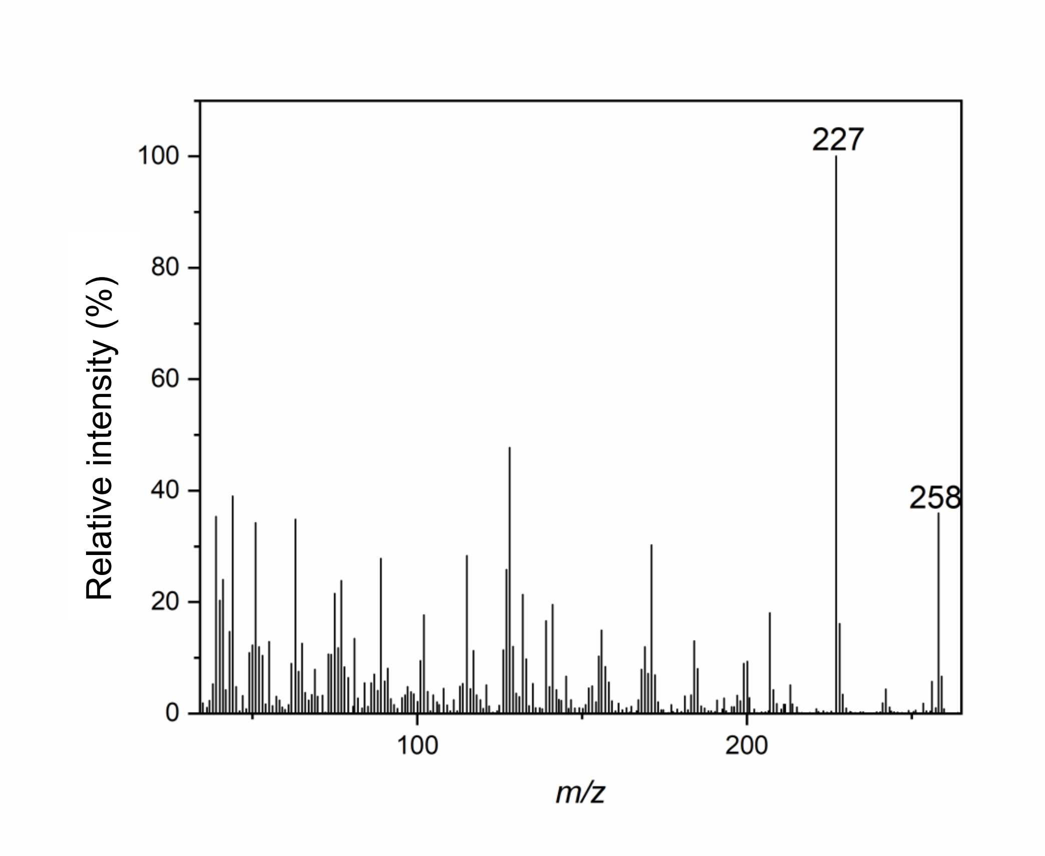

Figure S2: Mass Spectrum(IE, 70 eV) of compound **3**.

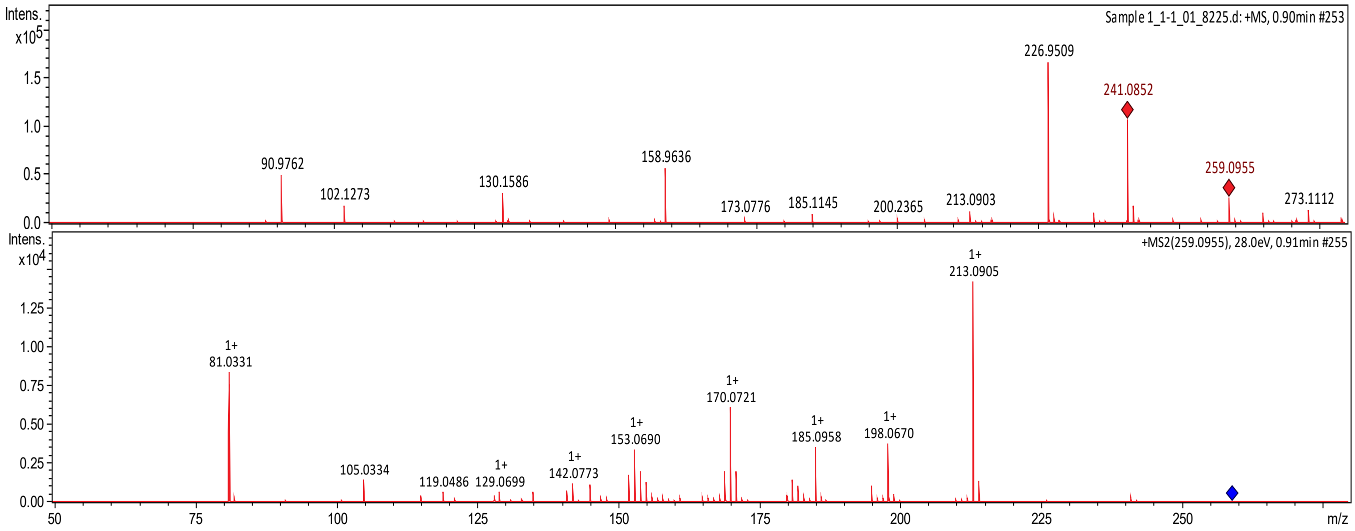

Figure S3: High-resolution mass spectrum (HRMS, ESI) of compound **3**.

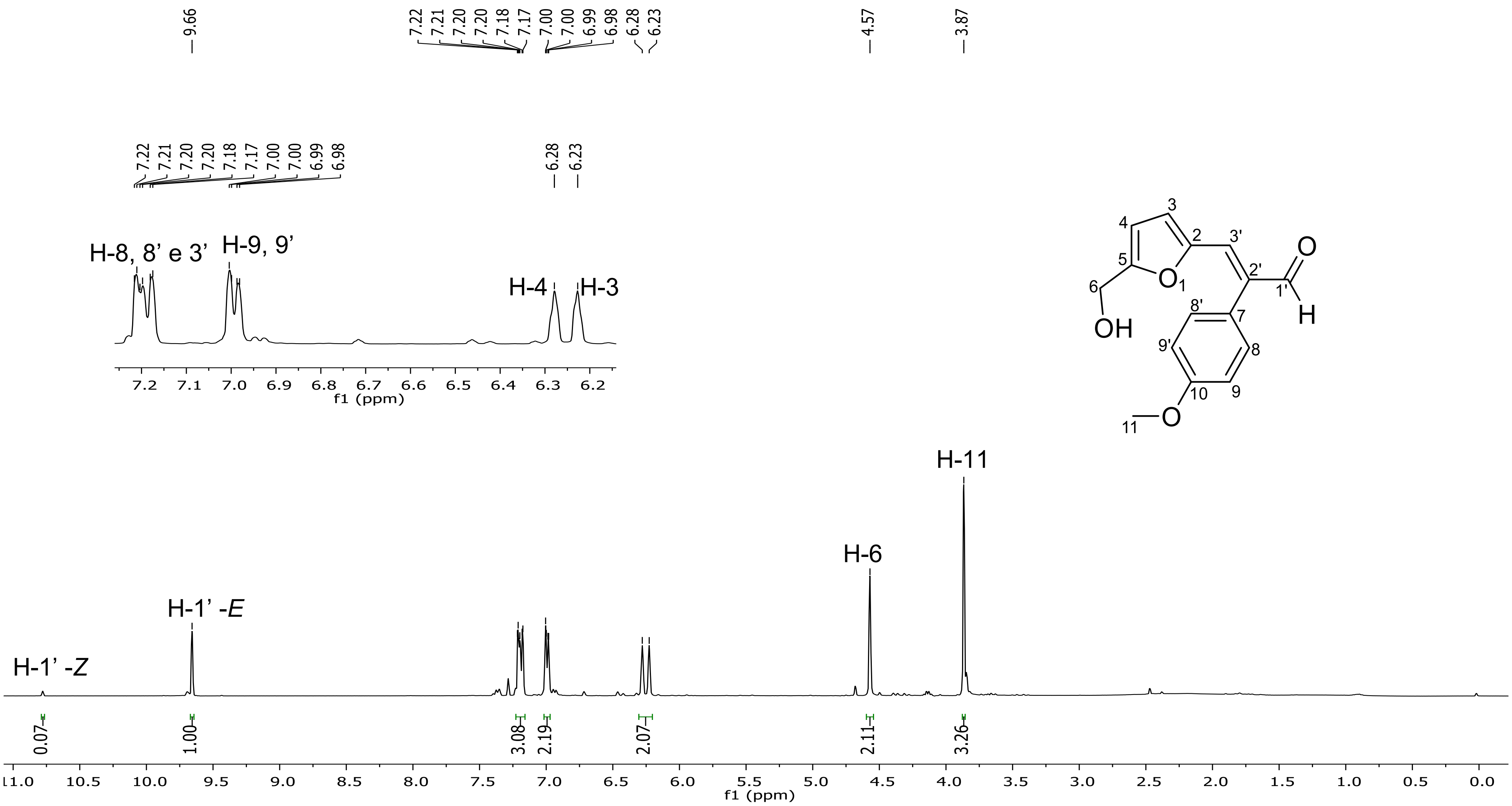

Figure S4: ^{1}H NMR (300 MHz, $CDCl_3$) of compound **3**.

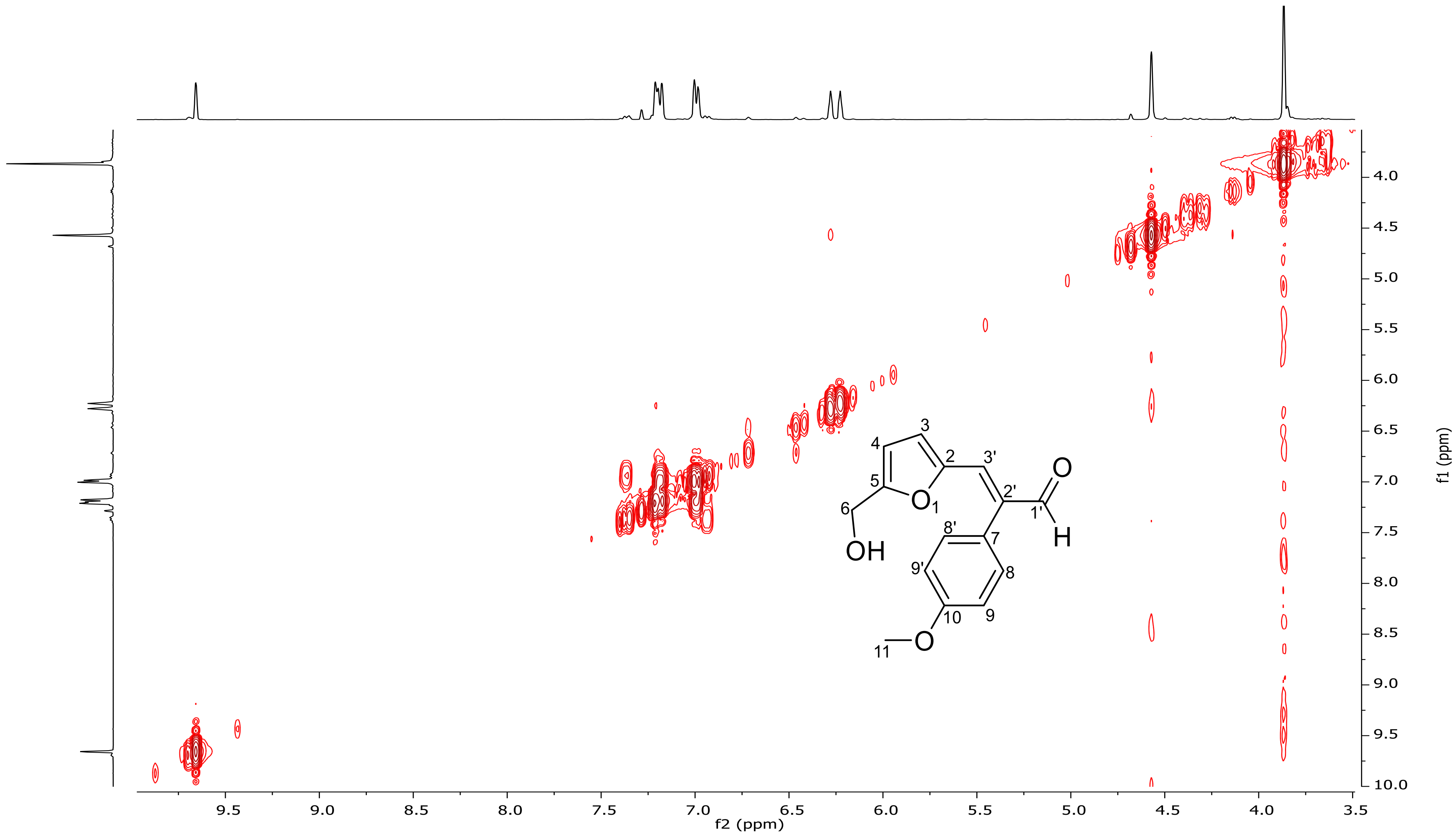

Figure S5: $^{1}H,^{1}H$-COSY (400 MHz, CDCl3) of compound **3**.

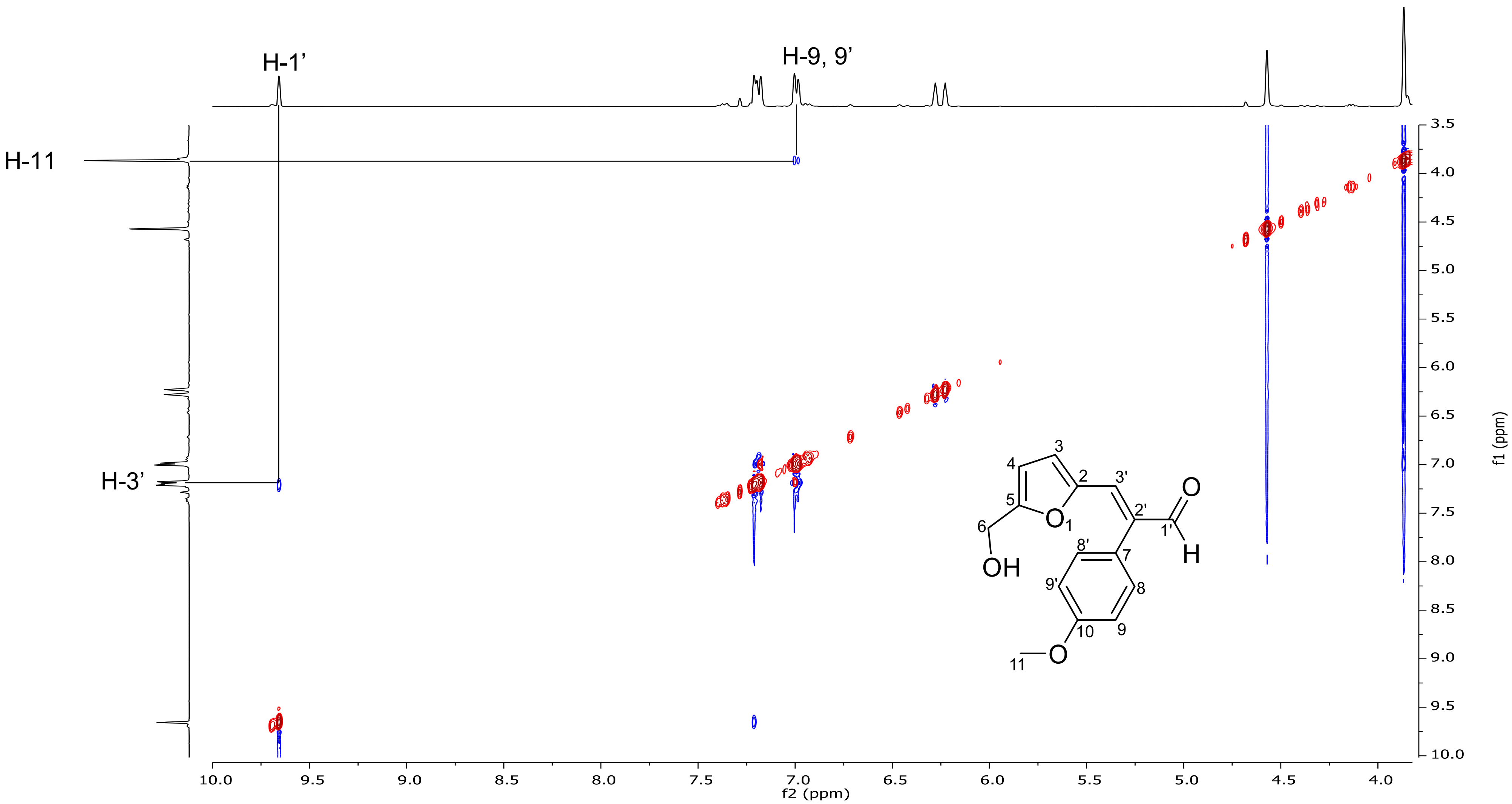

Figure S6: $^{1}H,^{1}H$-NOESY (400 MHz, $CDCl_3$) of compound **3**.

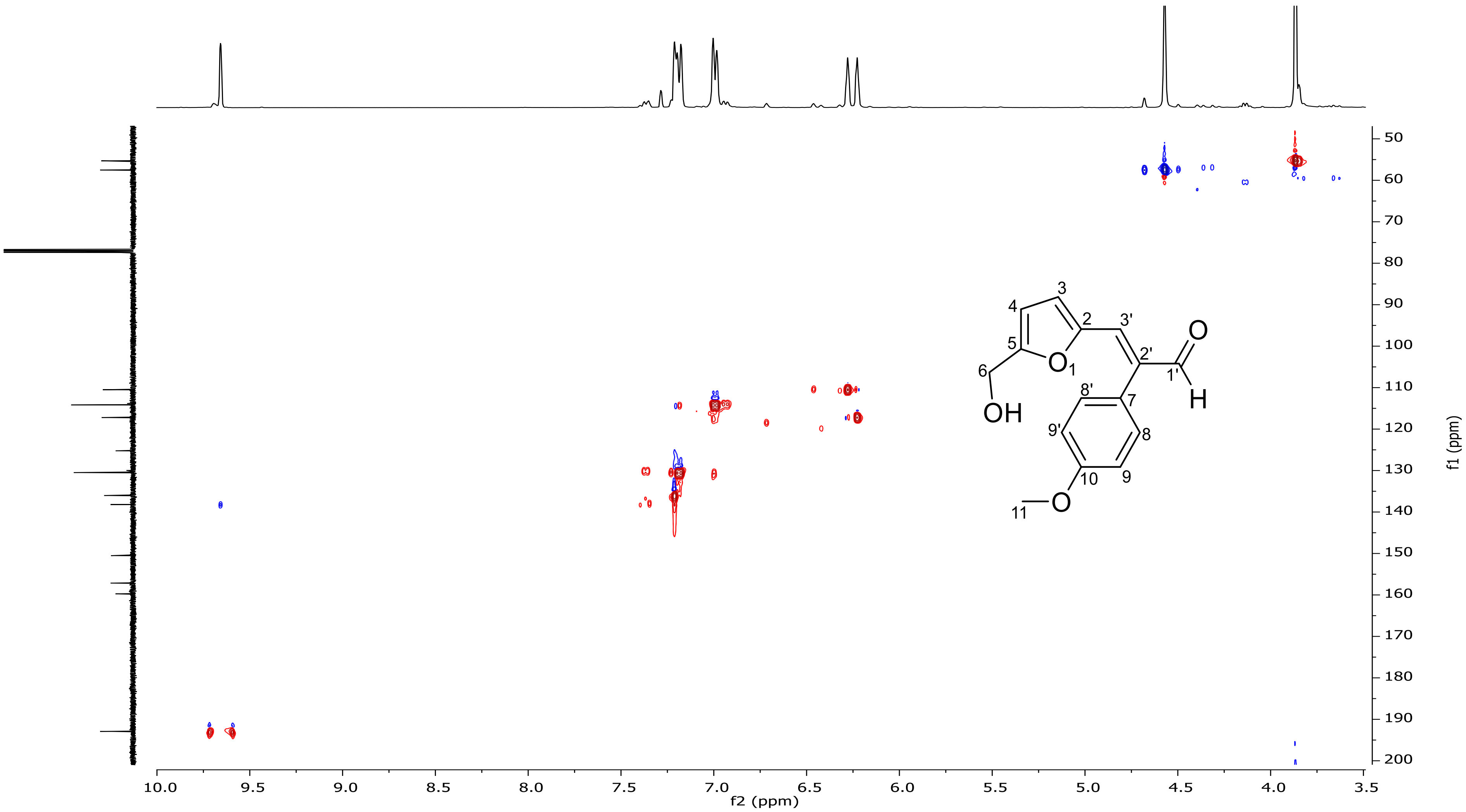

Figure S7: ^{1}H,^{13}C-HSQC (400, 101 MHz, CDCl3) of compound **3**.

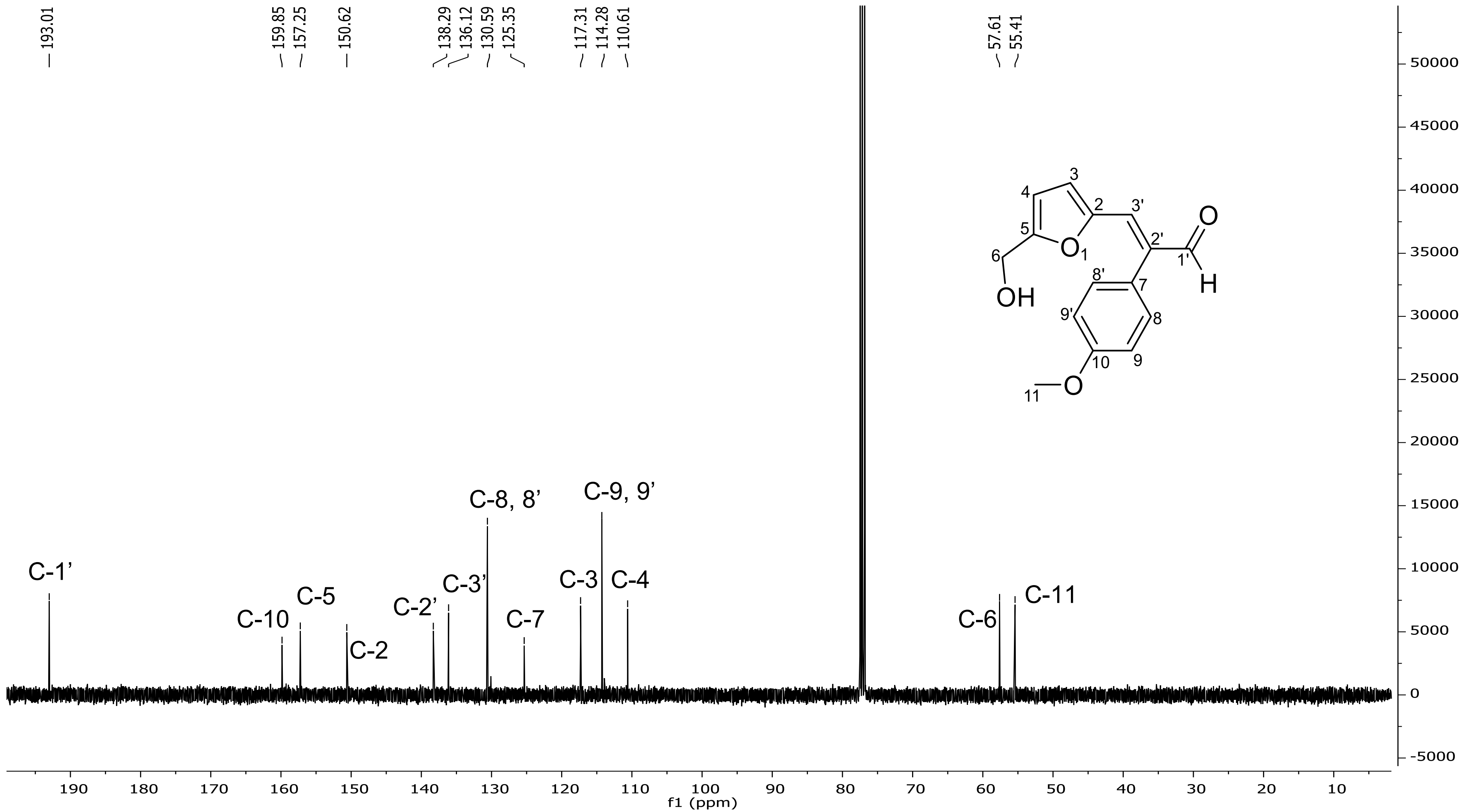

Figure S8: ^{13}C NMR (101 MHz, $CDCl_3$) of compound **3.**

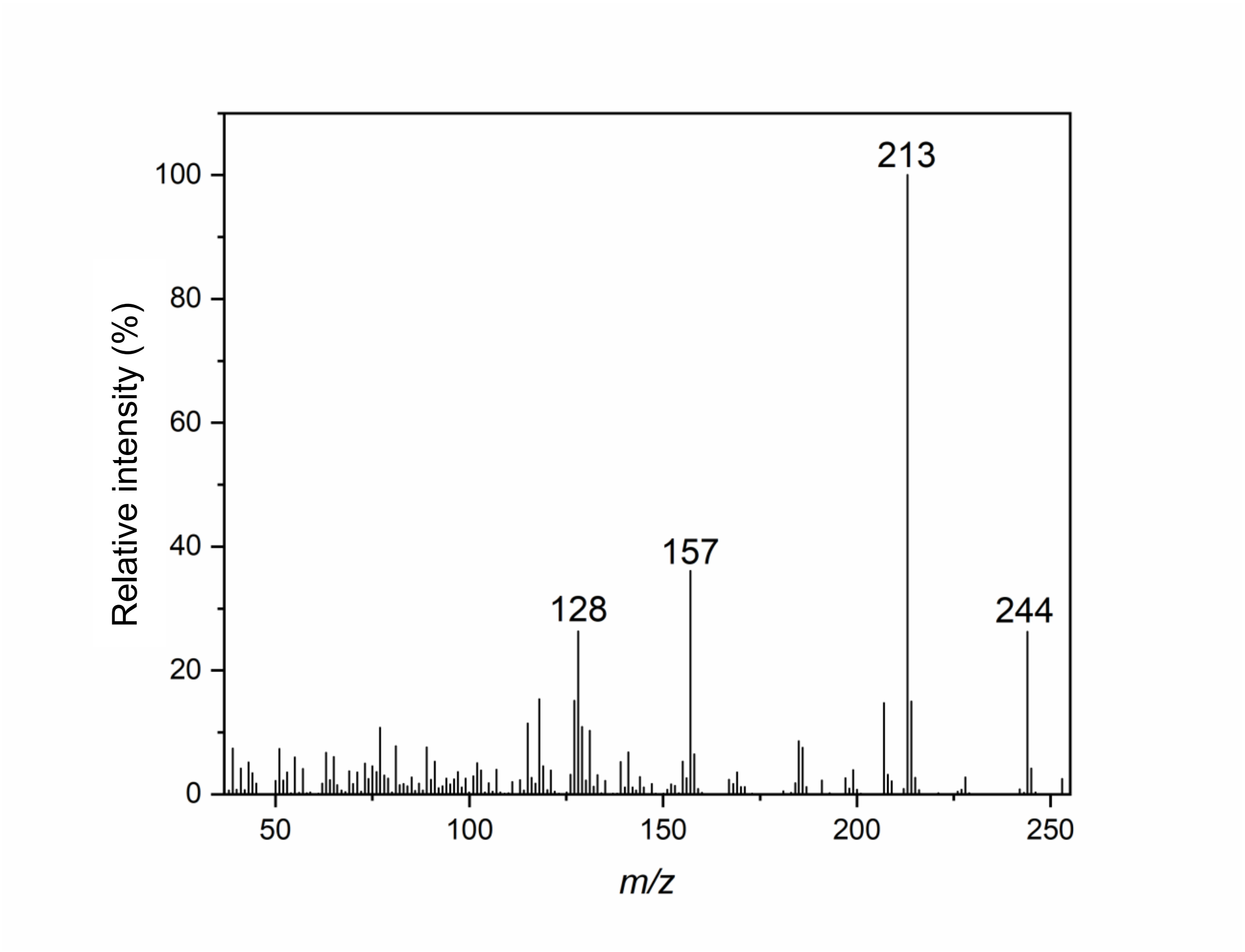

Figure S9: Espectro de Massas (IE, 70 eV) of compound **4**.

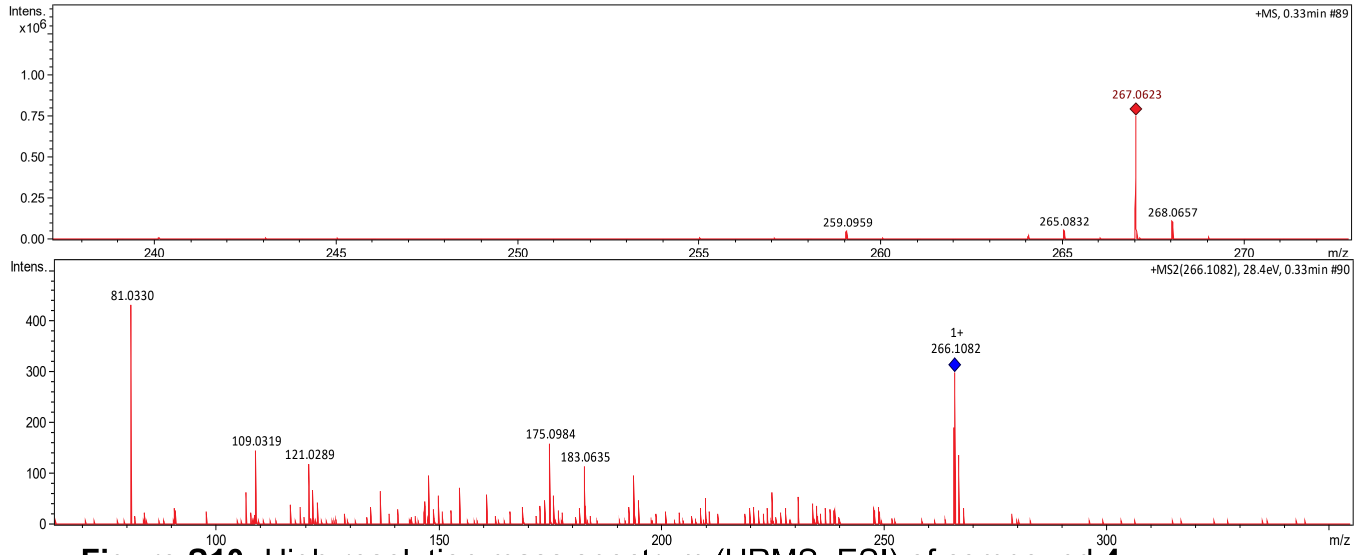

Figure S10: High-resolution mass spectrum (HRMS, ESI) of compound **4**

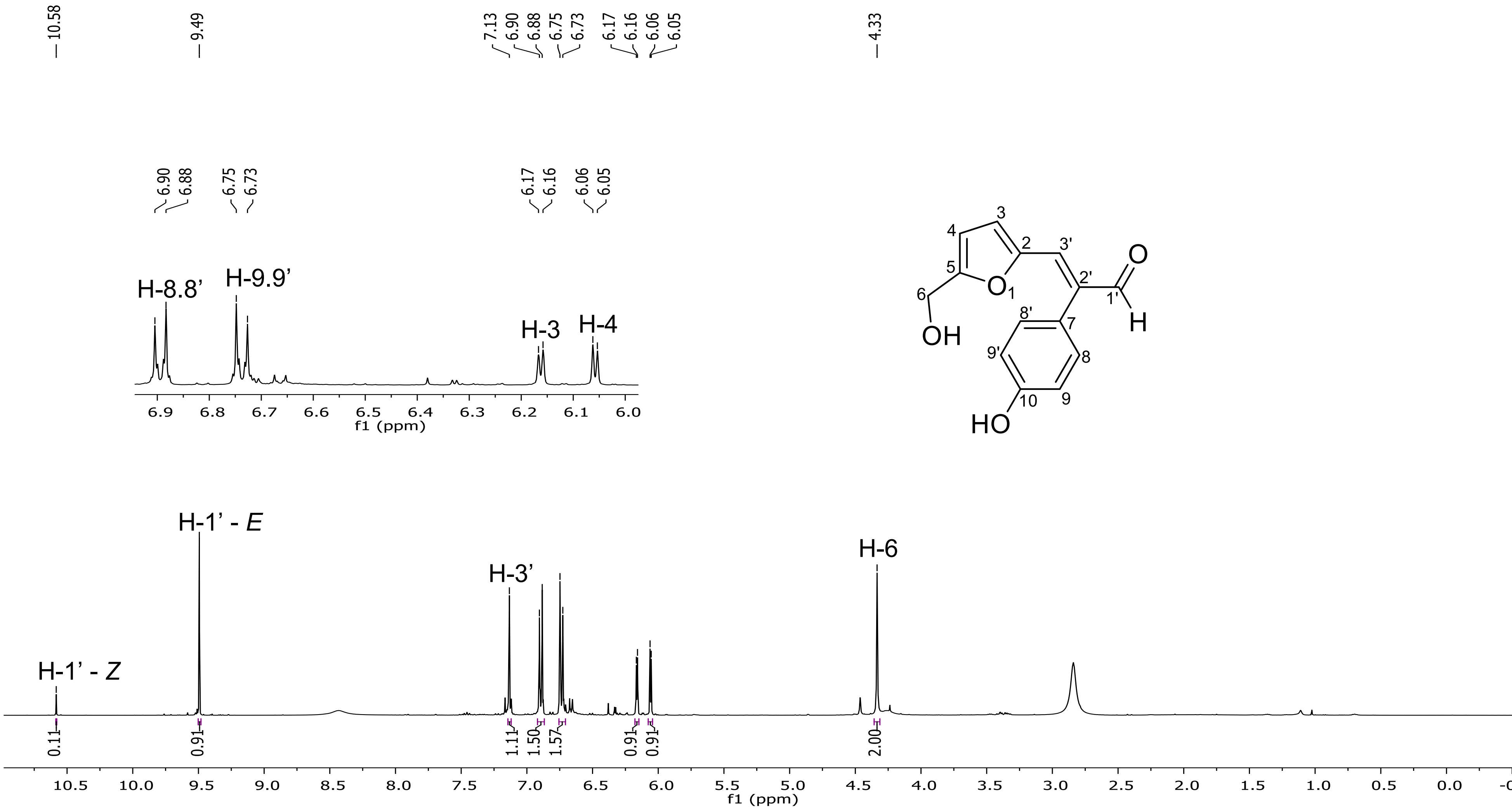

Figure S11: ^{1}H NMR (300 MHz, $CDCl_3$) of compound **4**.

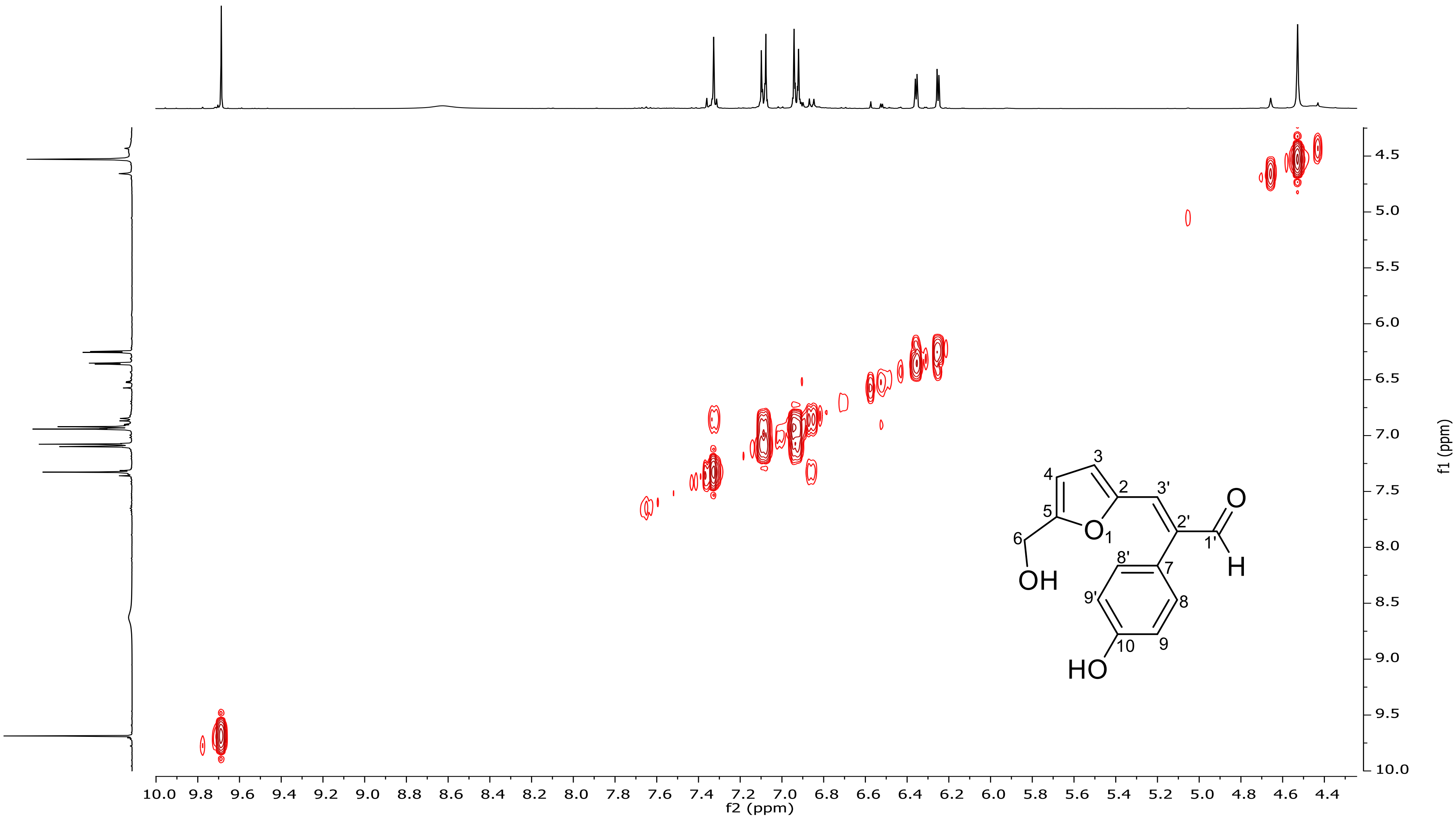

Figure S12: $^{1}H,^{1}H$-COSY (400 MHz, CDCl3) of compound **4**.

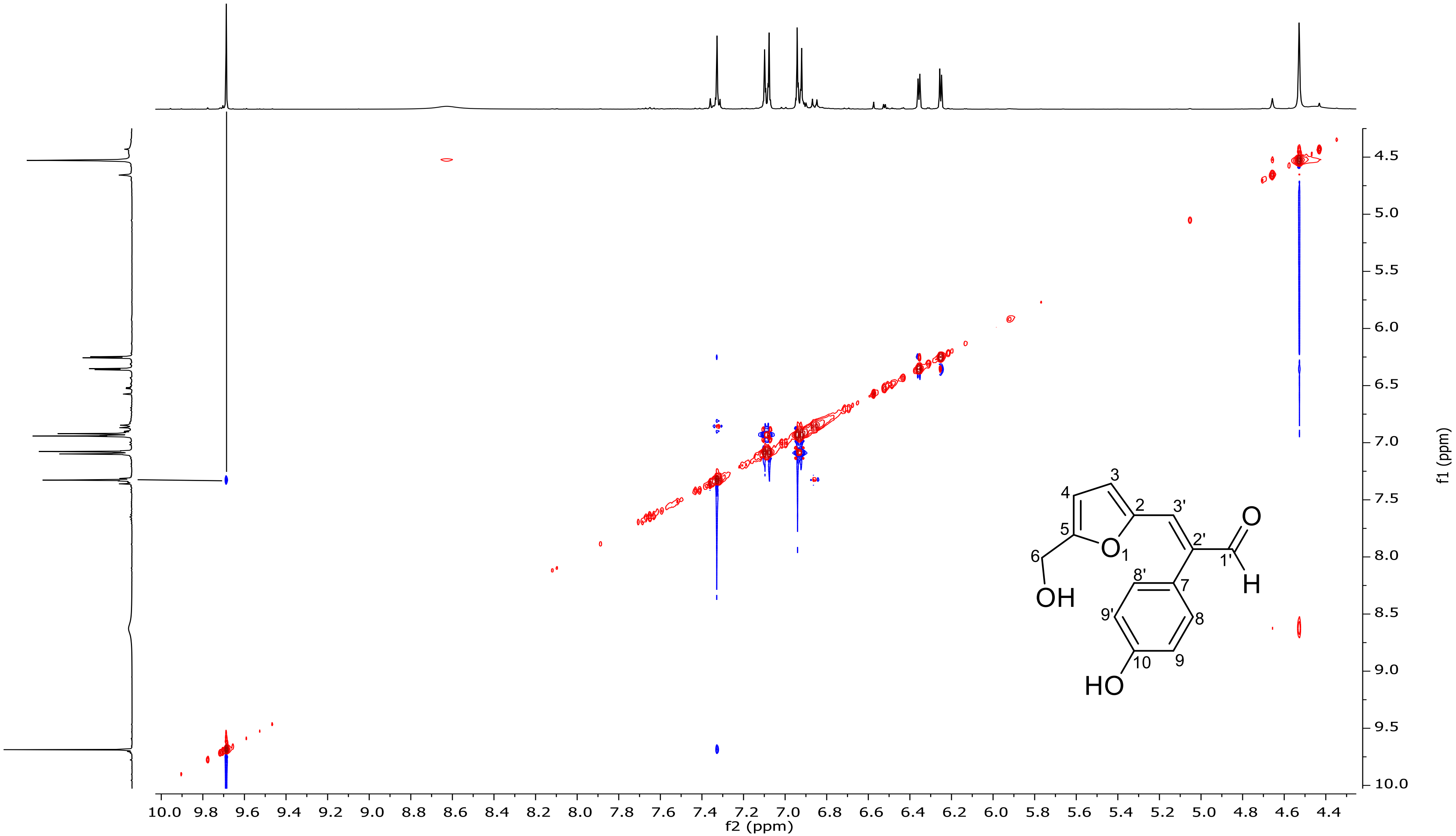

Figure S13: $^{1}H,^{1}H$-NOESY (400 MHz, $CDCl_3$) of compound **4**.

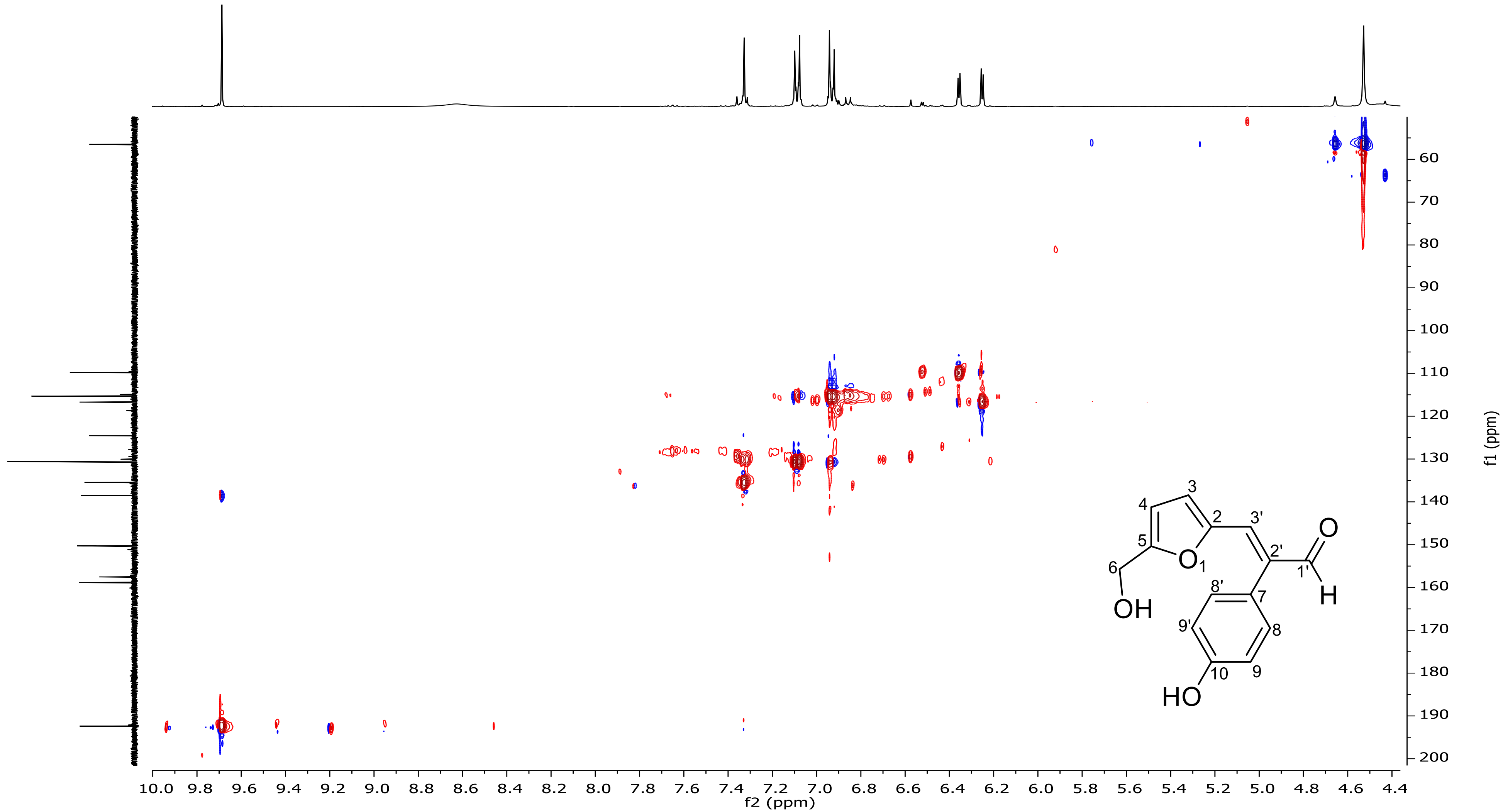

Figure S14: $^{1}H,^{13}C$-HSQC (400, 101 MHz, CDCl3) of compound **4**.

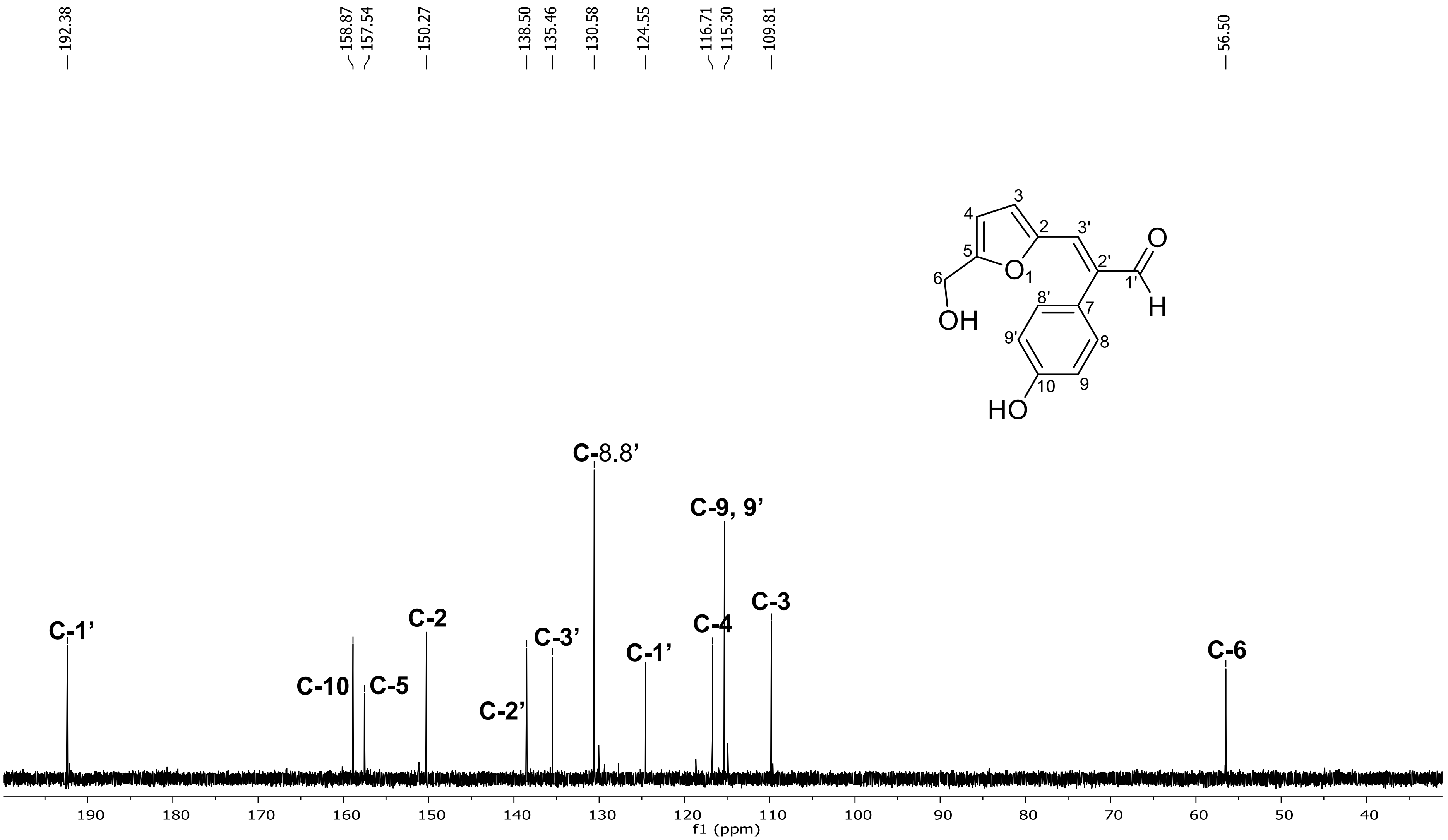

Figure S15: ^{13}C NMR (101 MHz, $CDCl_3$) of compound **4**.

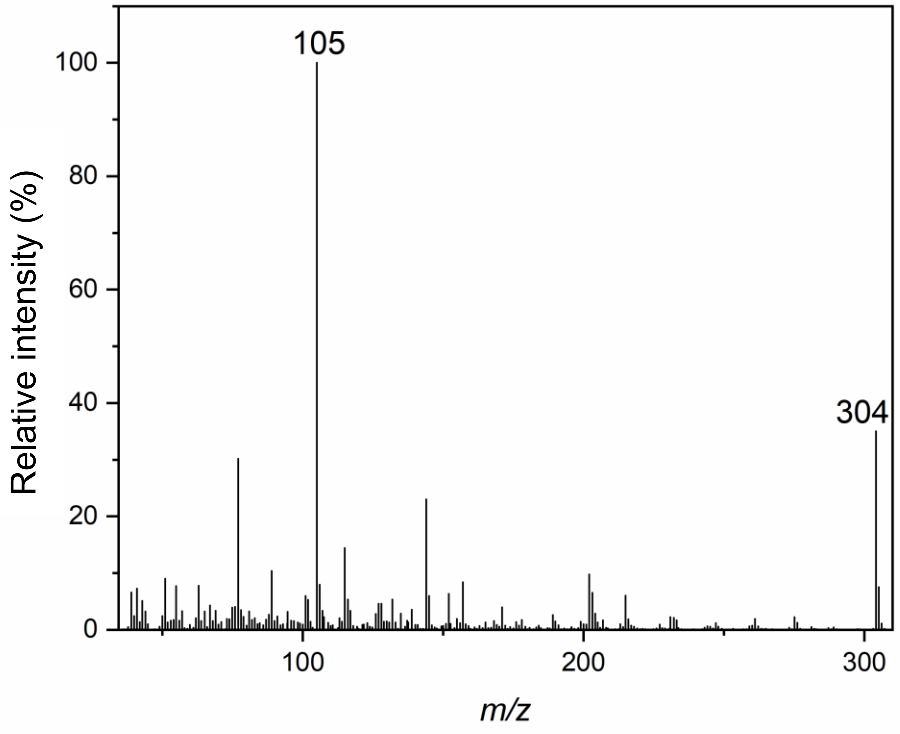

Figure S15: Espectro de Massas (IE, 70 eV) of compound **5.**

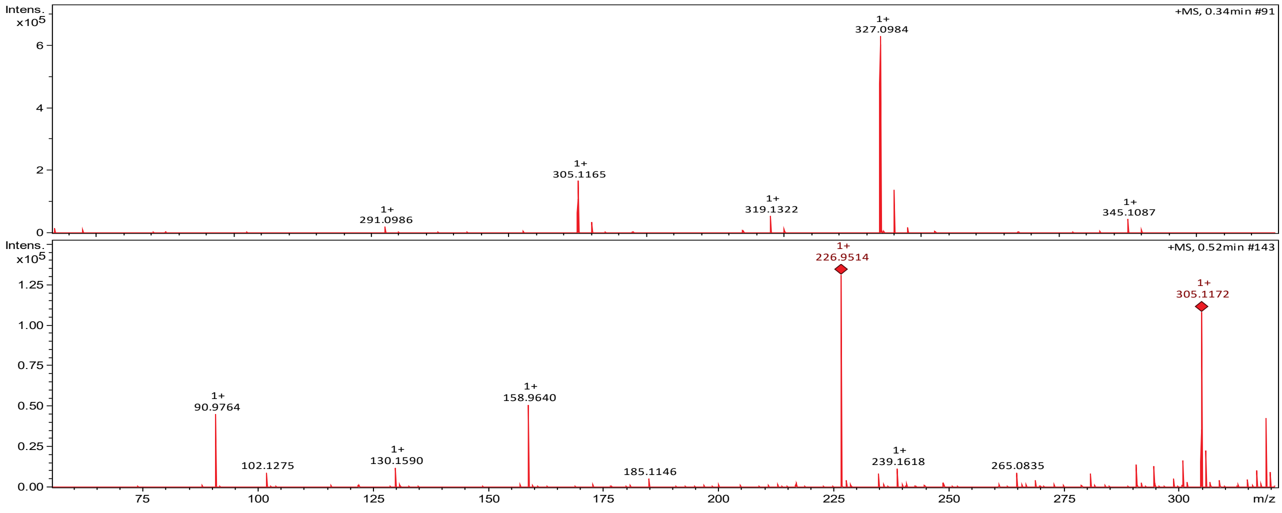

Figure S17: High-resolution mass spectrum (HRMS, ESI) of compound **5.**

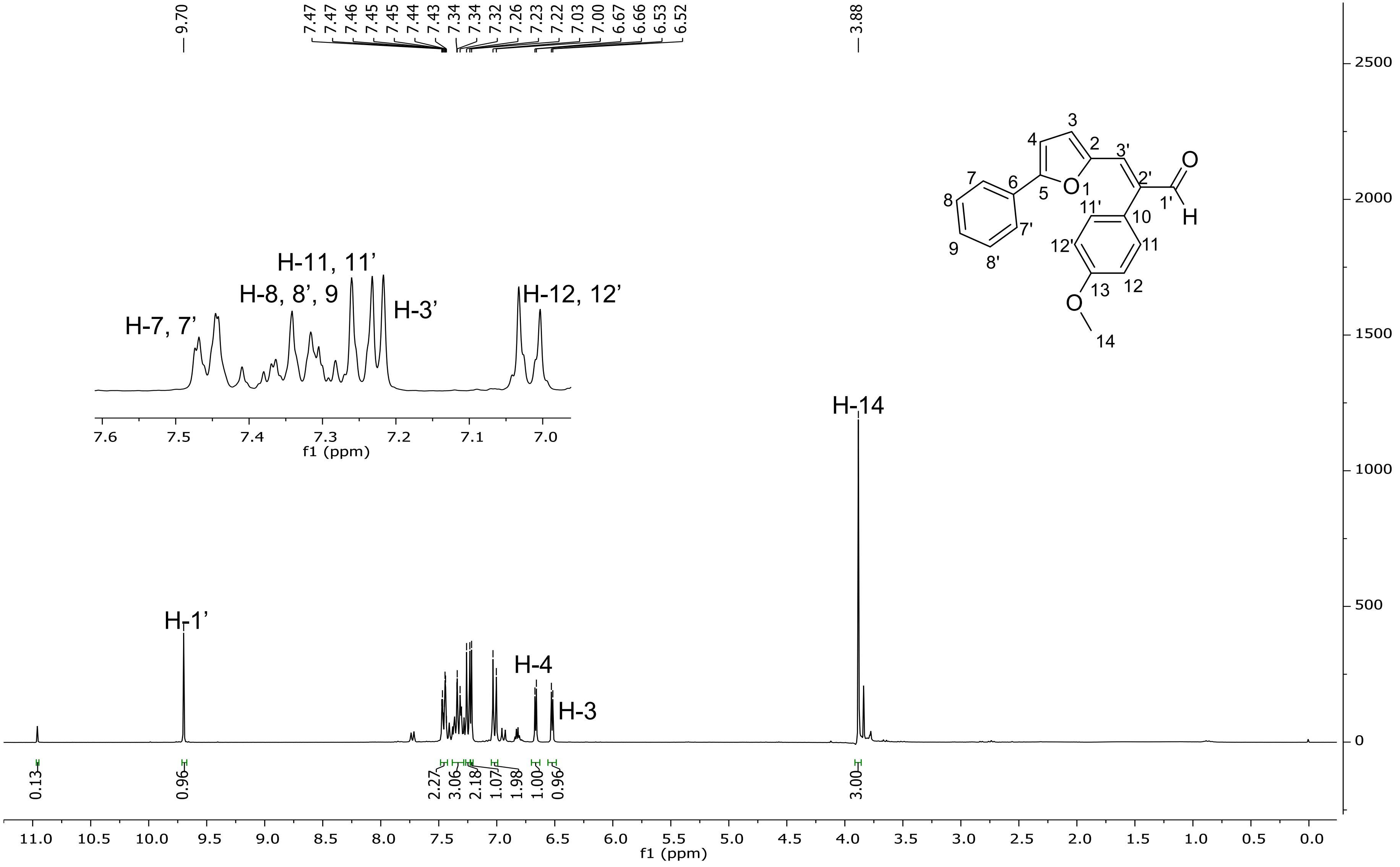

Figure S18: ^{1}H NMR (300 MHz, $CDCl_3$) of compound **5**.

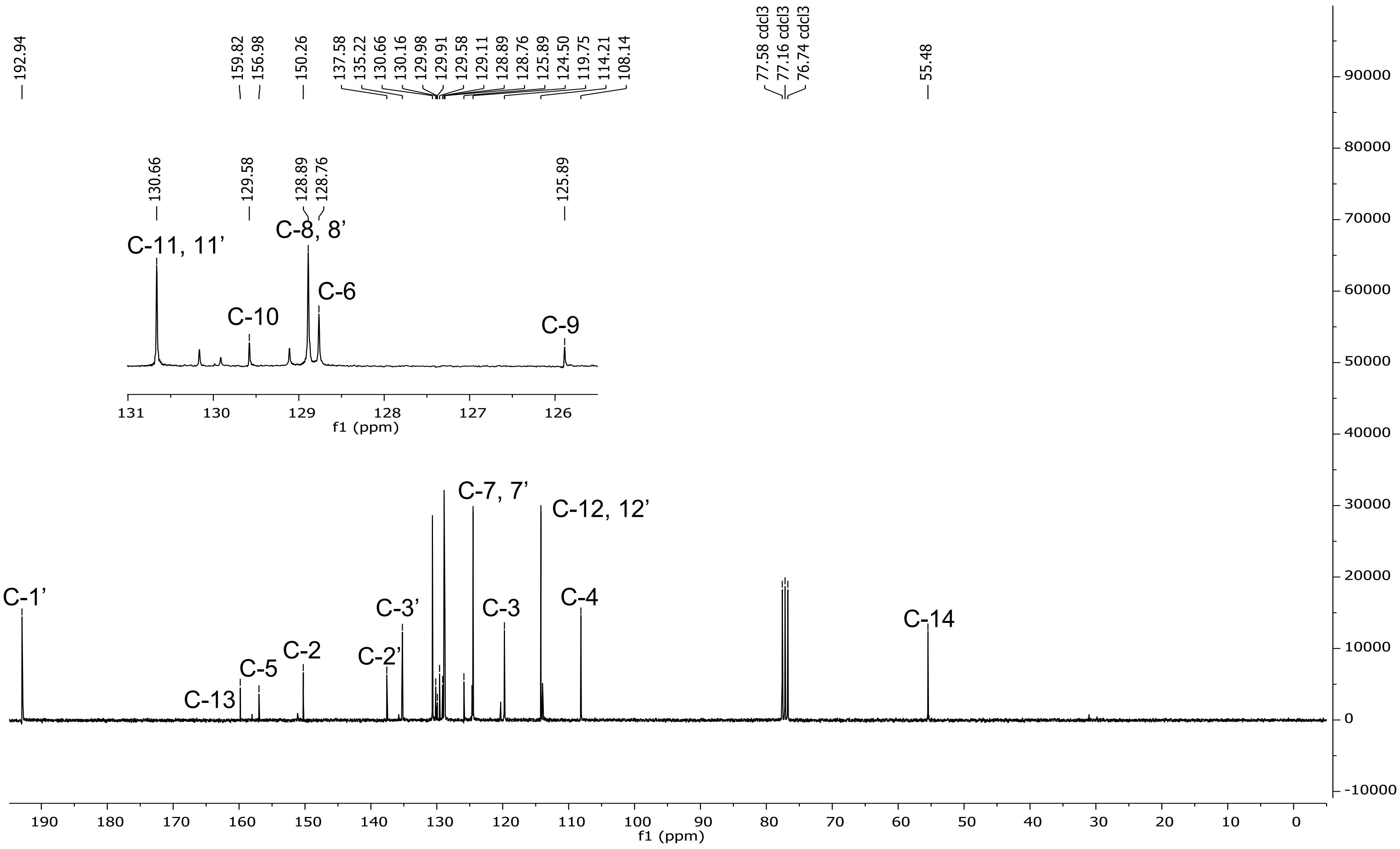

Figure S19: ^{13}C NMR (101 MHz, $CDCl_3$) of compound **5**.

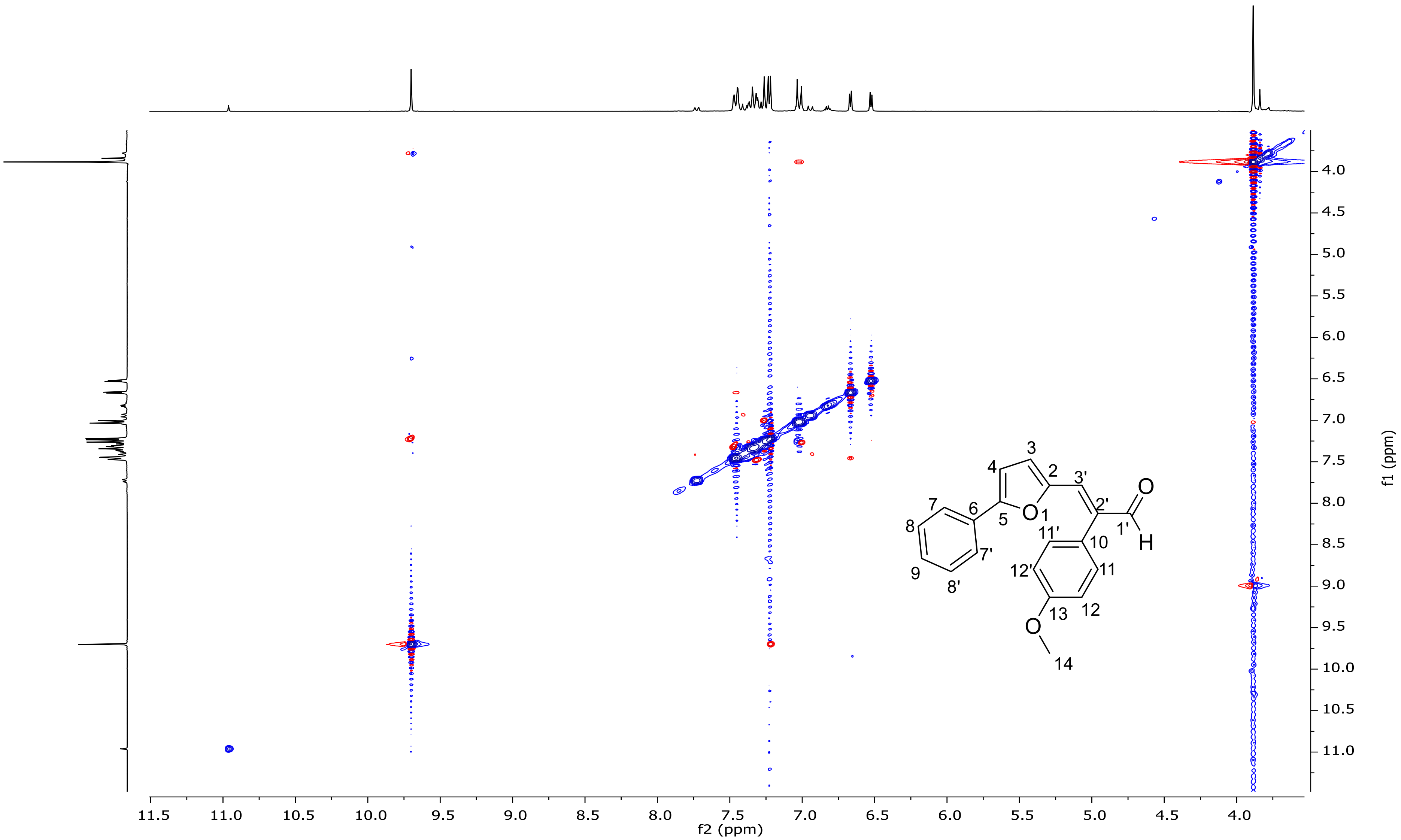

Figure S20: ^1H,^1H-NOESY (400 MHz, $CDCl_3$) of compound **5**.

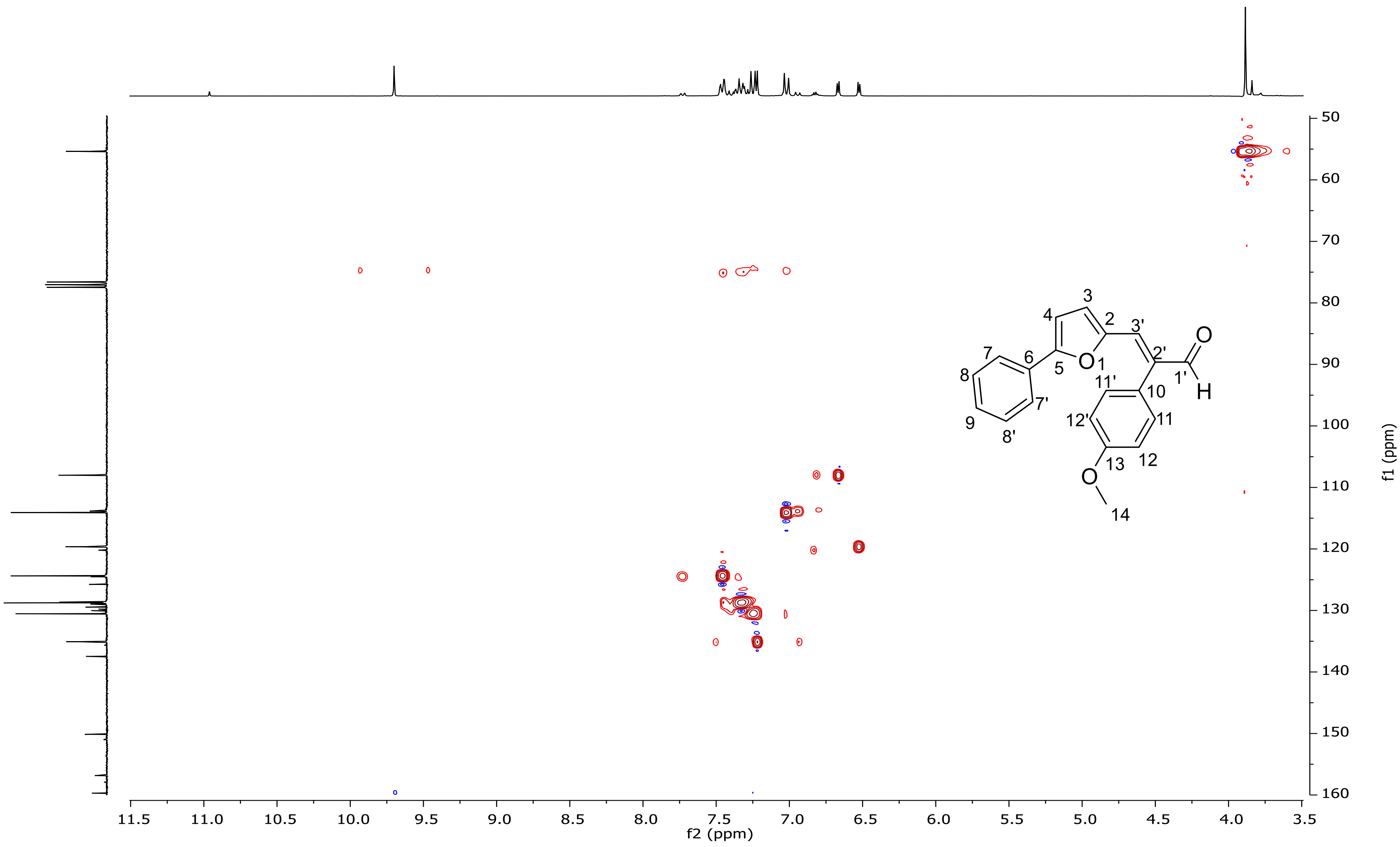

Figure S21: $^{1}H,^{13}C$-HSQC (400, 101 MHz, $CDCl_3$) of compound **5.**

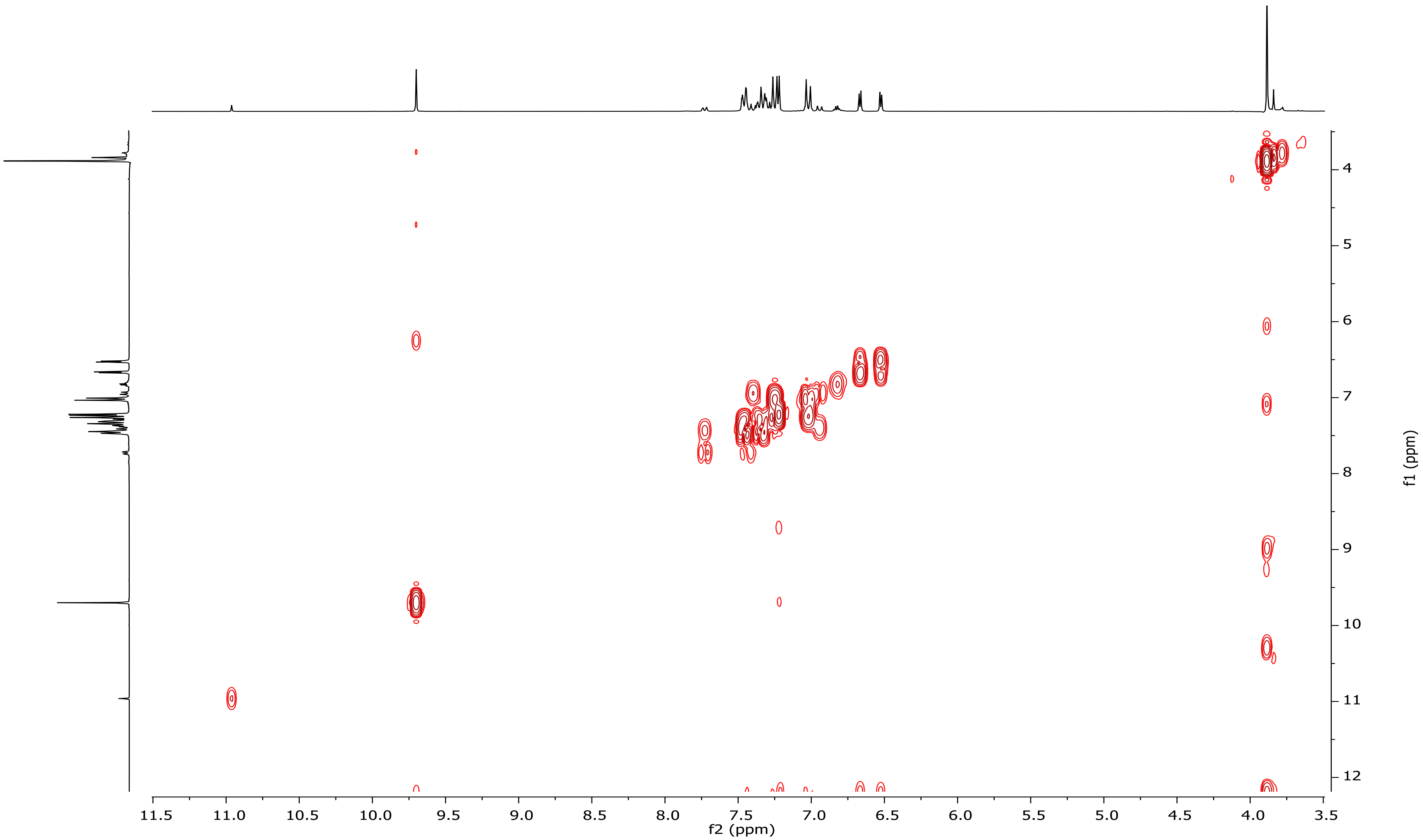

Figure S22: $^{1}H,^{1}H$-COSY (400 MHz, $CDCl_3$) of compound **5**.

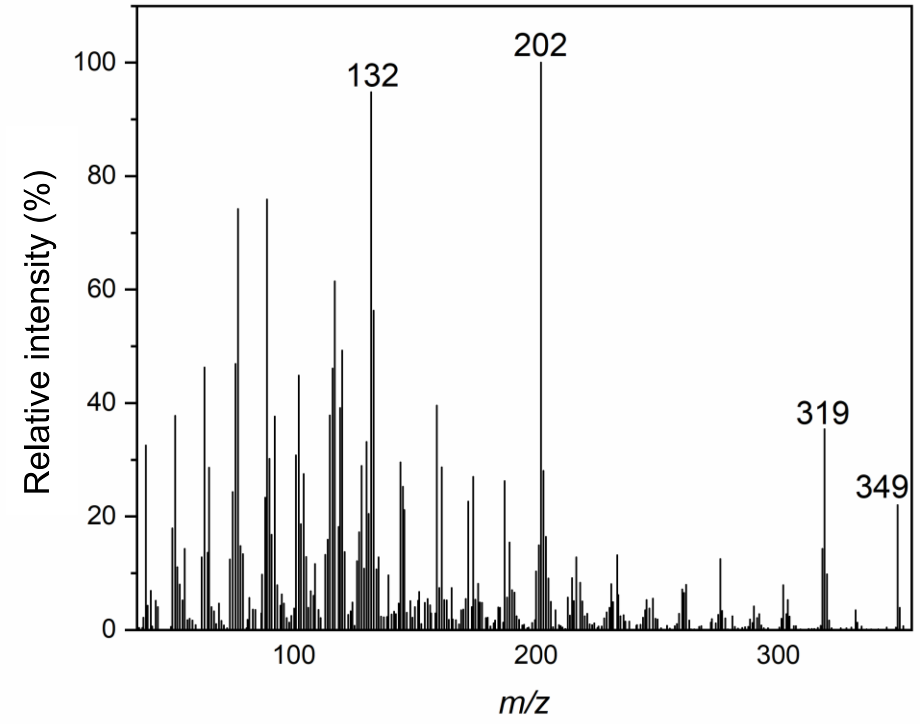

Figure S23: Mass Spectrum(IE, 70 eV) of compound **6**.

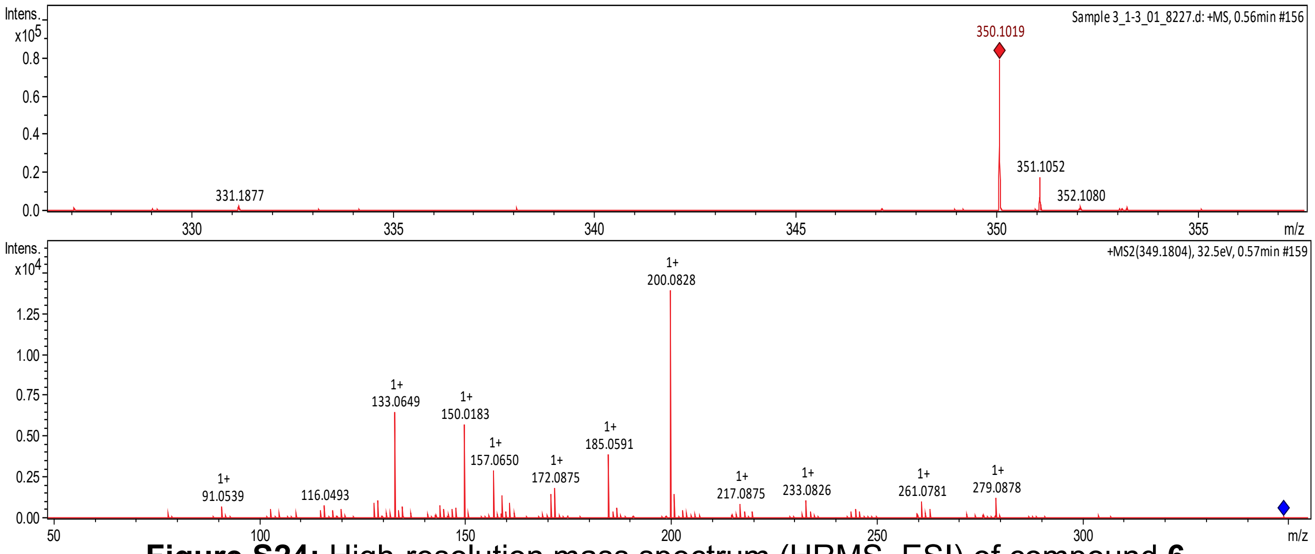

Figure S24: High-resolution mass spectrum (HRMS, ESI) of compound **6.**

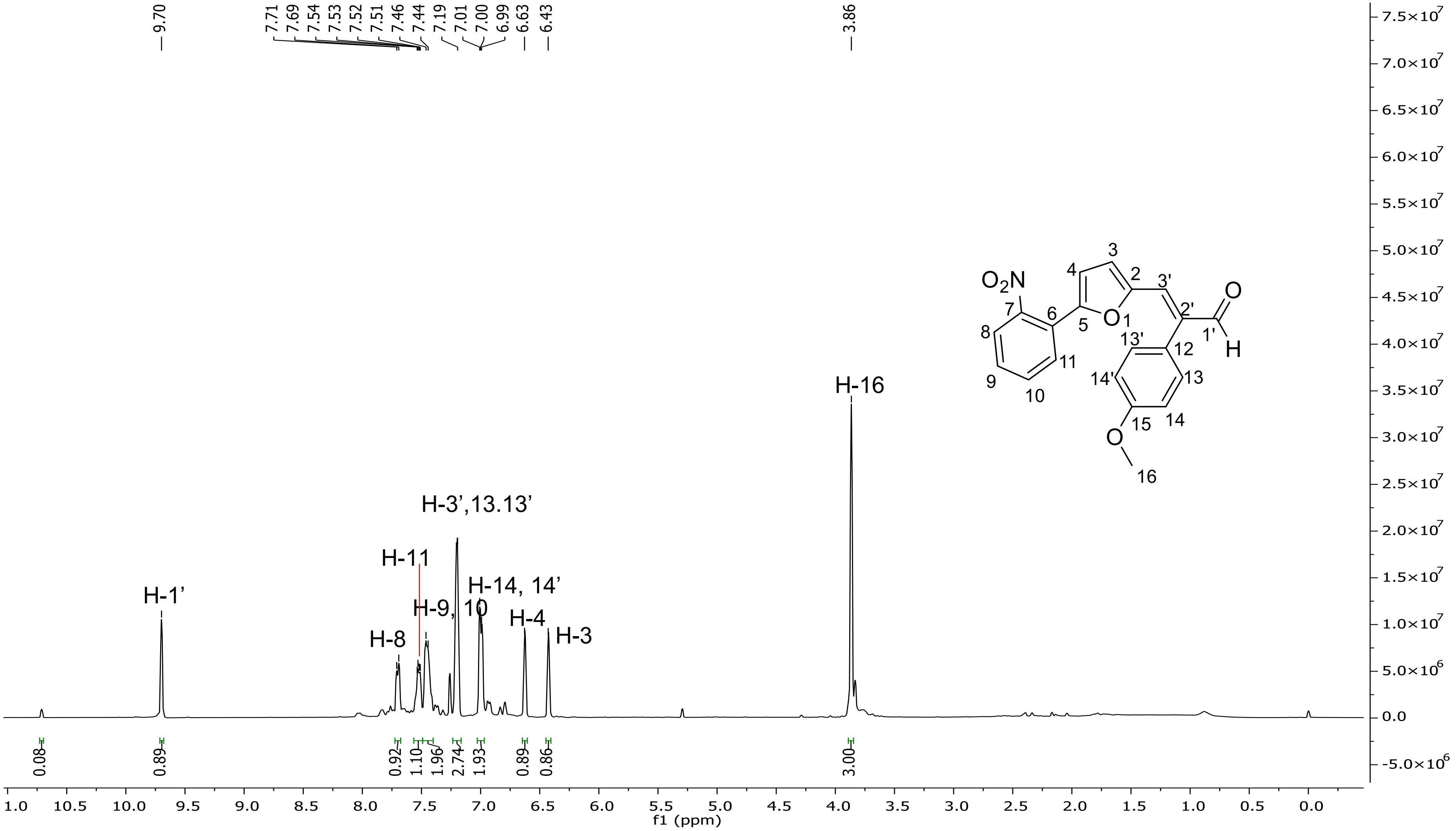

Figure S25: ^{1}H NMR (300 MHz, $CDCl_3$) of compound **6**.

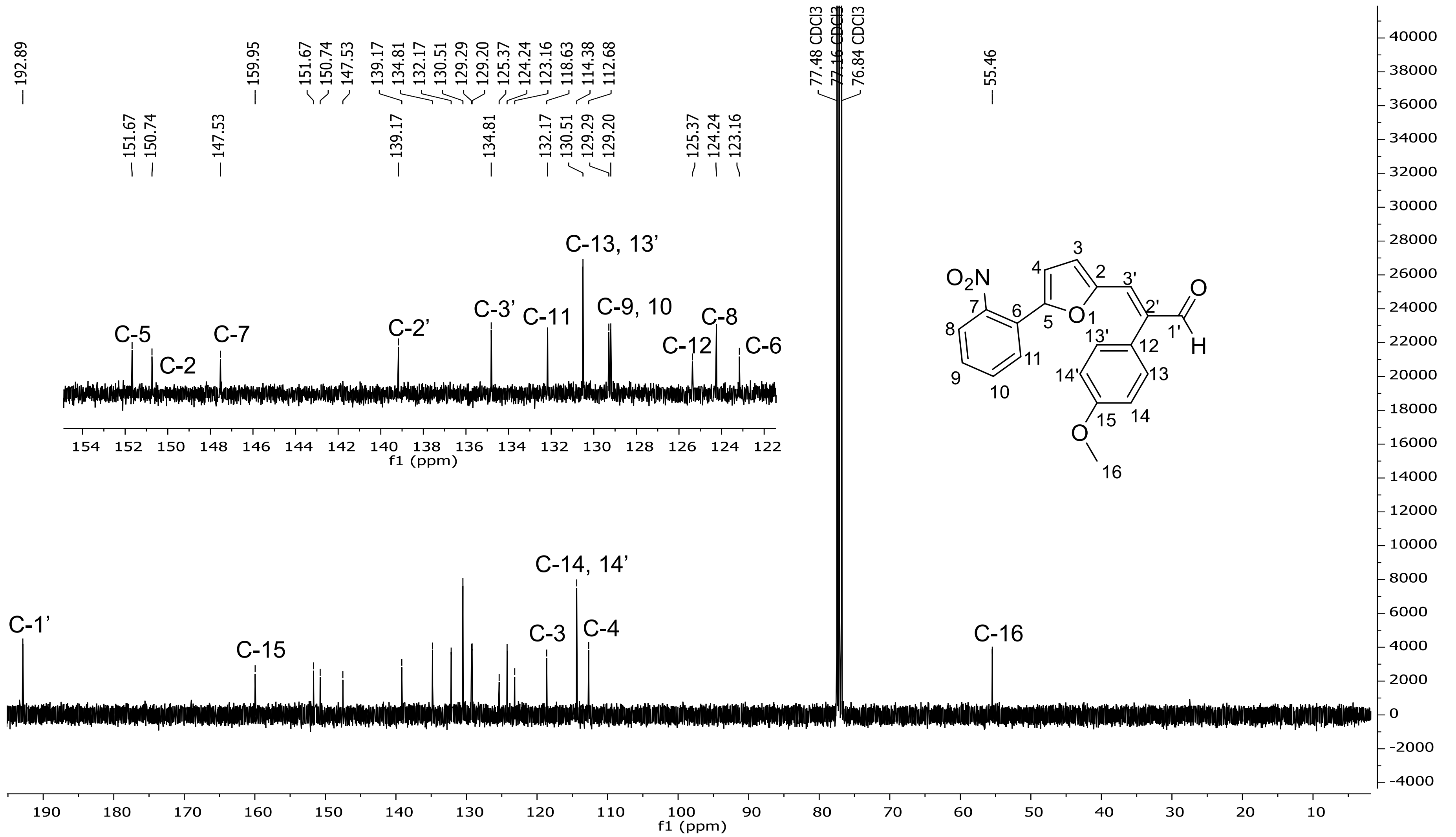

Figure S26: ^{13}C NMR (101 MHz, $CDCl_3$) of compound **6**.

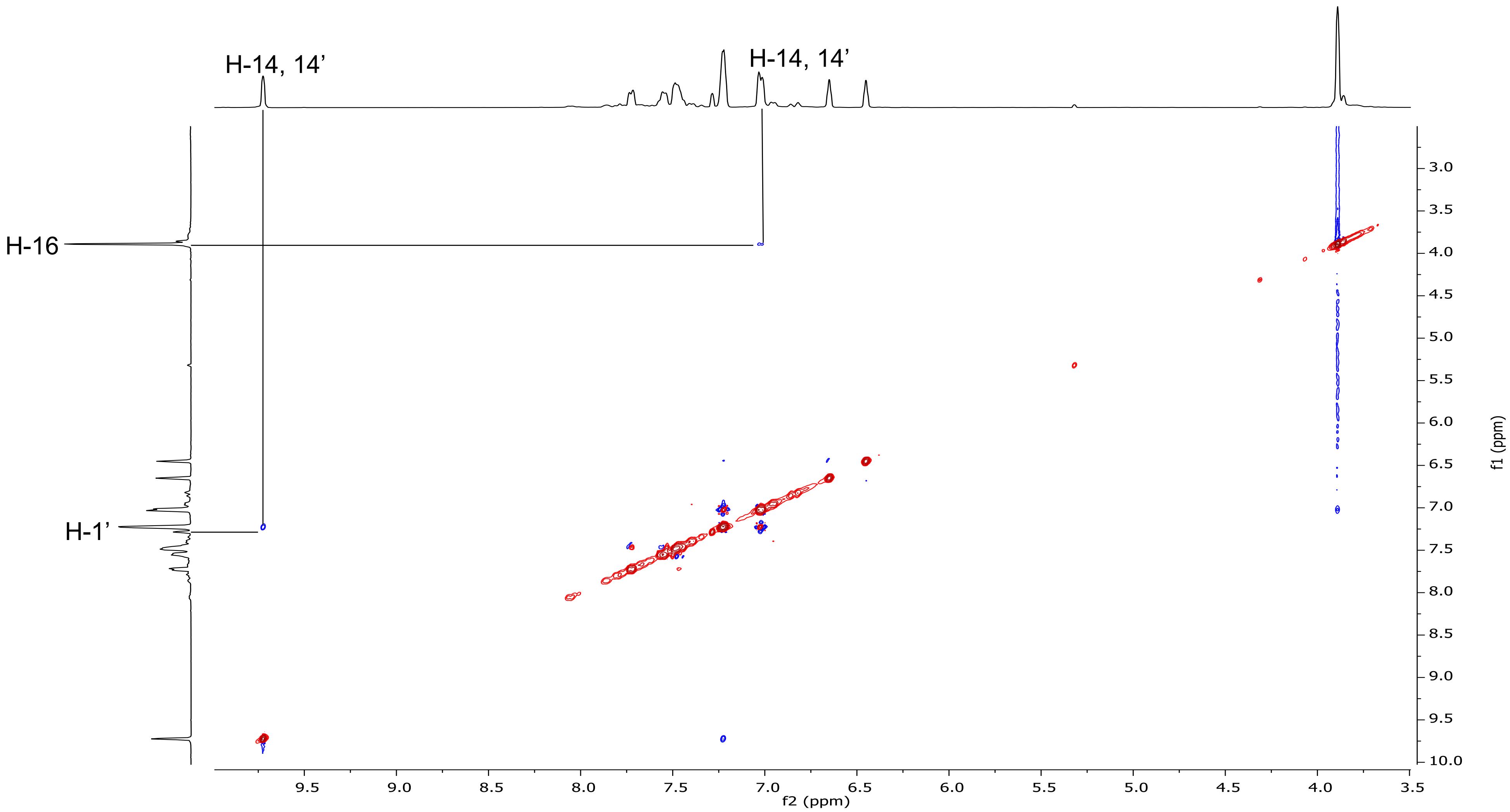

Figure S27: $^{1}H,^{1}H$-NOESY (400 MHz, $CDCl_3$) of compound **6**.

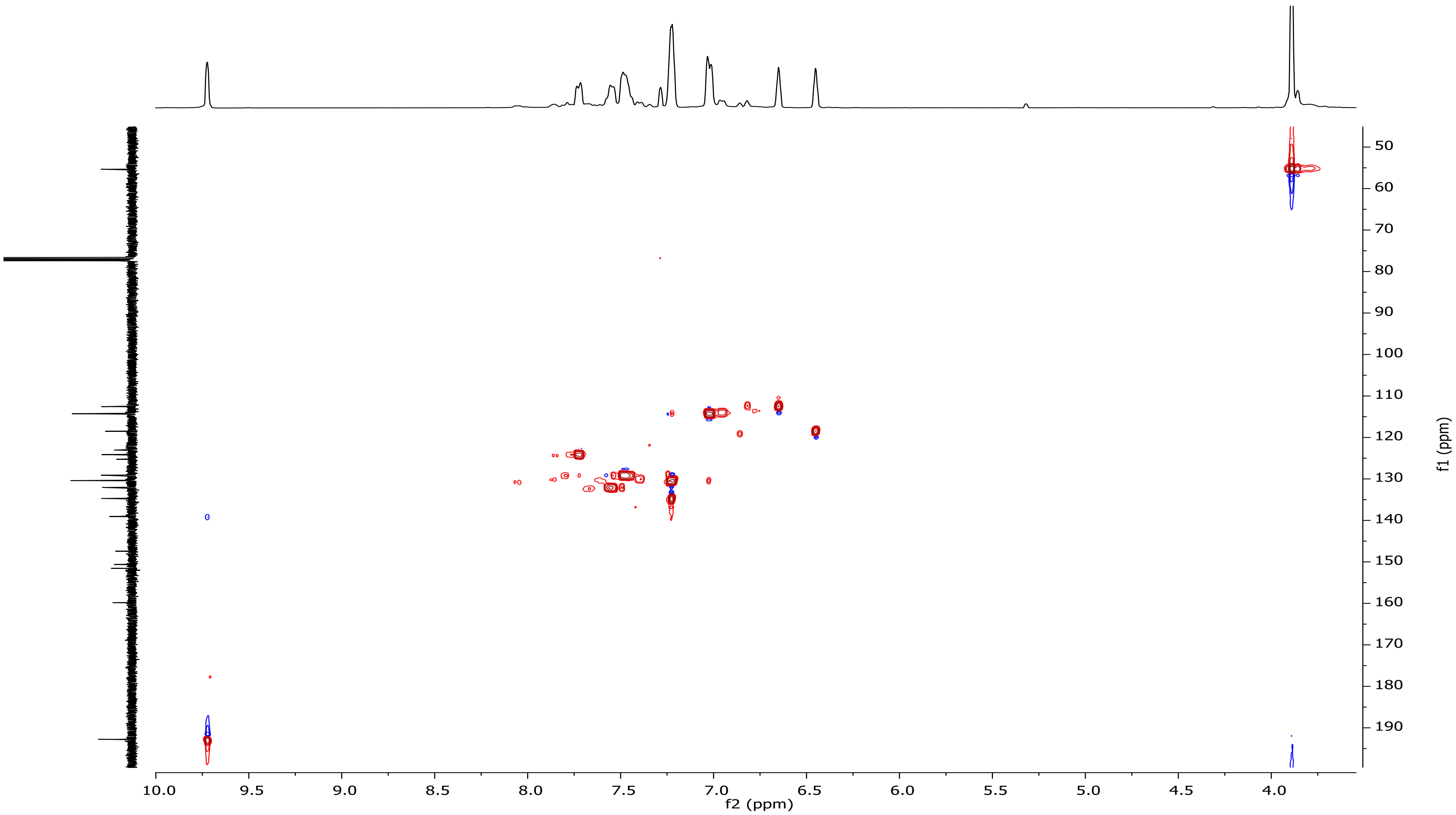

Figure S28: $^{1}H,^{13}C$-HSQC (400, 101 MHz, $CDCl_3$) of compound **6**.

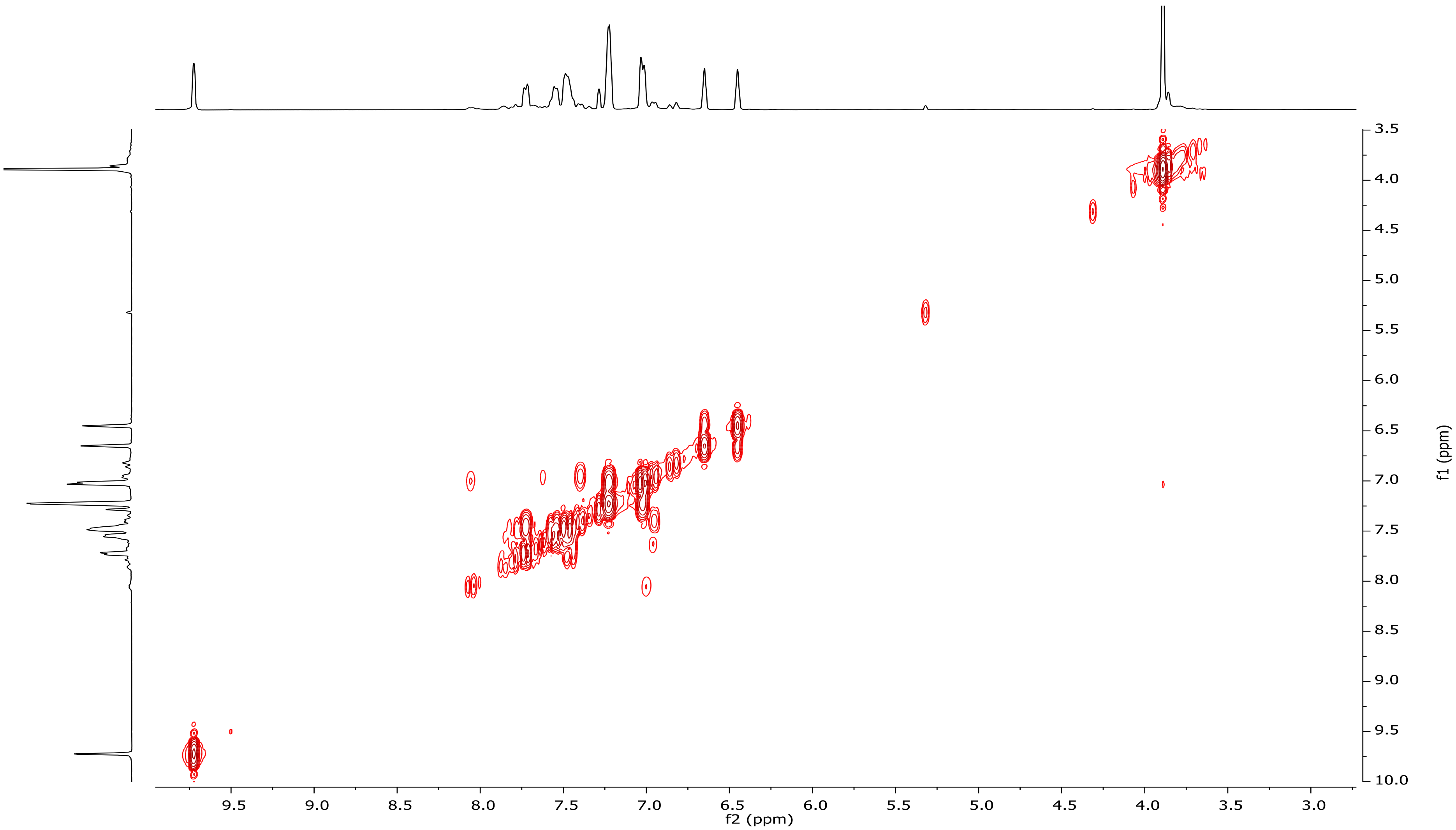

Figure S29: $^{1}H,^{1}H$-COSY (400 MHz, $CDCl_3$) of compound **6**.

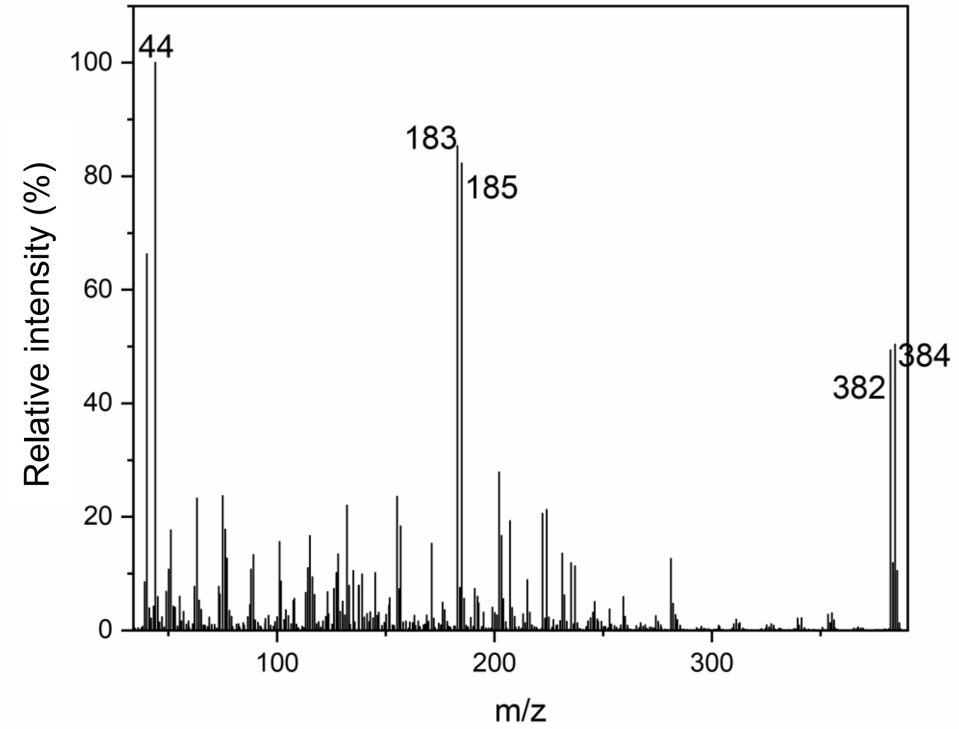

Figure S30: Mass Spectrum(IE, 70 eV) of compound **7**.

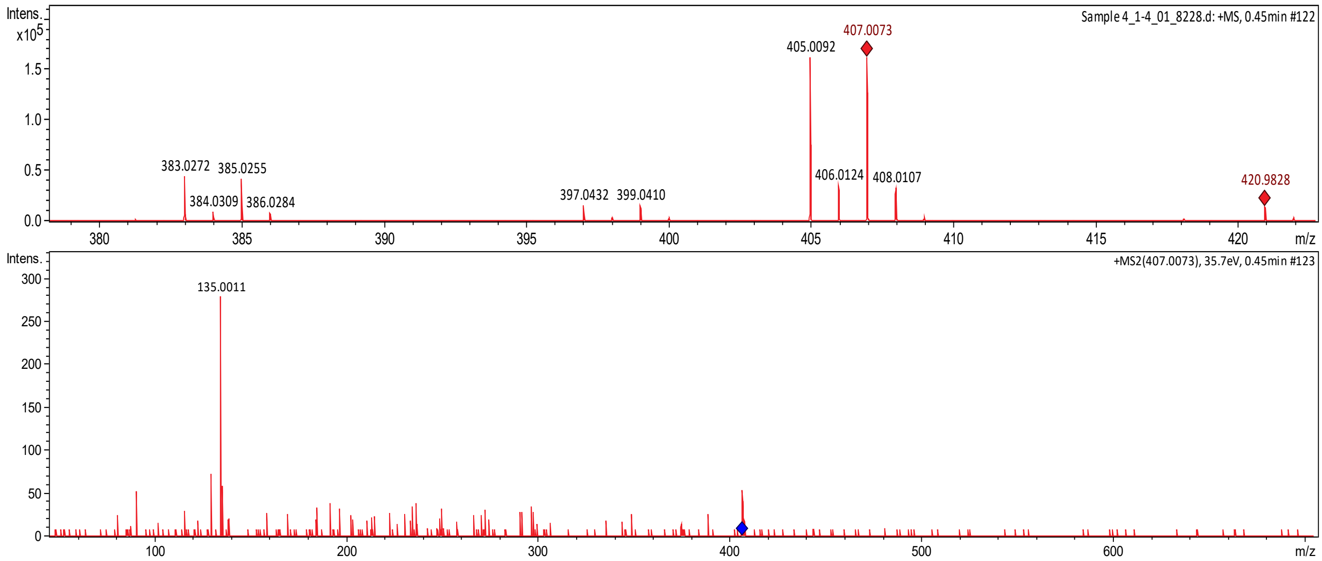

Figure S31: High-resolution mass spectrum (HRMS, ESI) of compound **7.**

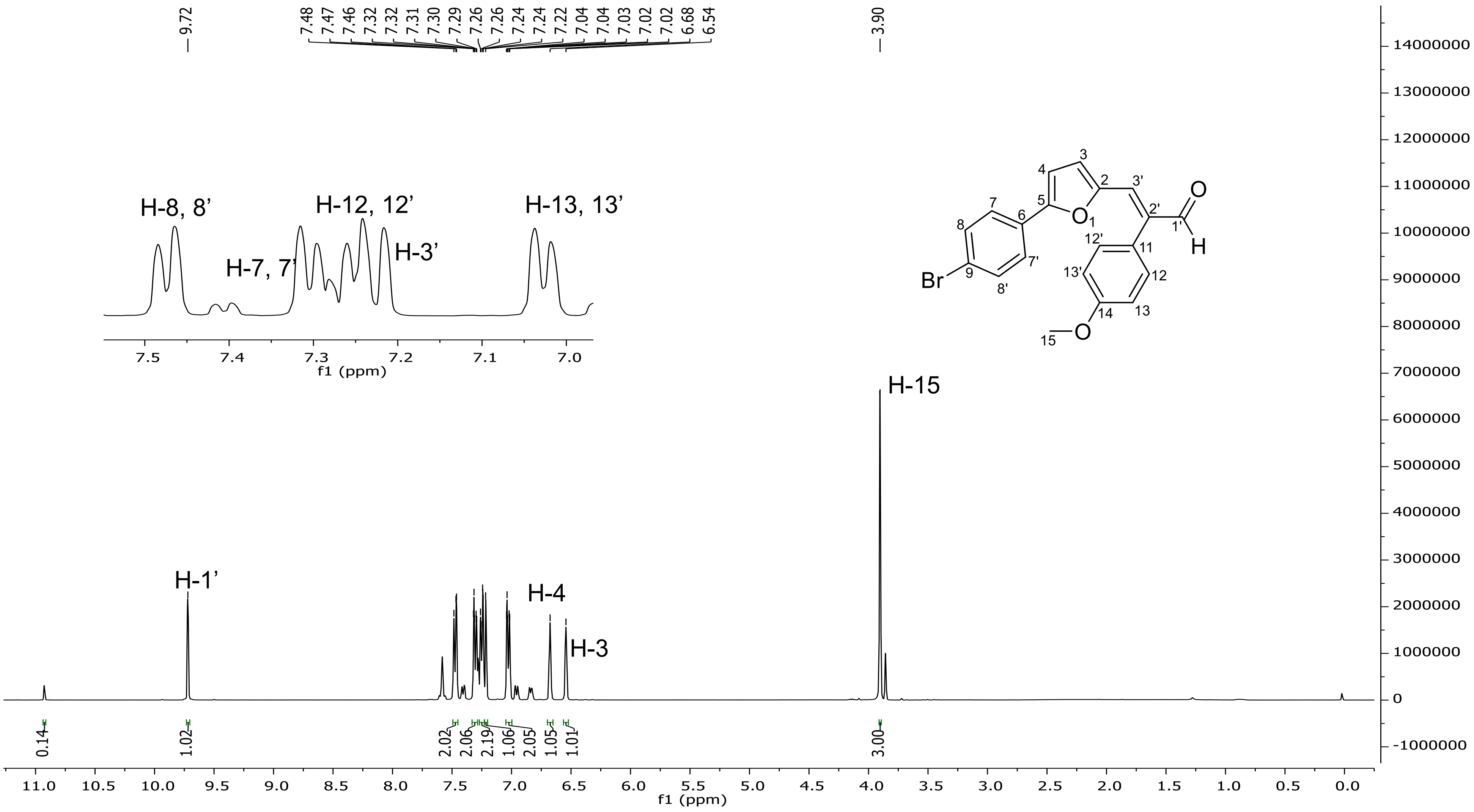

Figure S32: ^{1}H NMR (300 MHz, $CDCl_3$) of compound **7**.

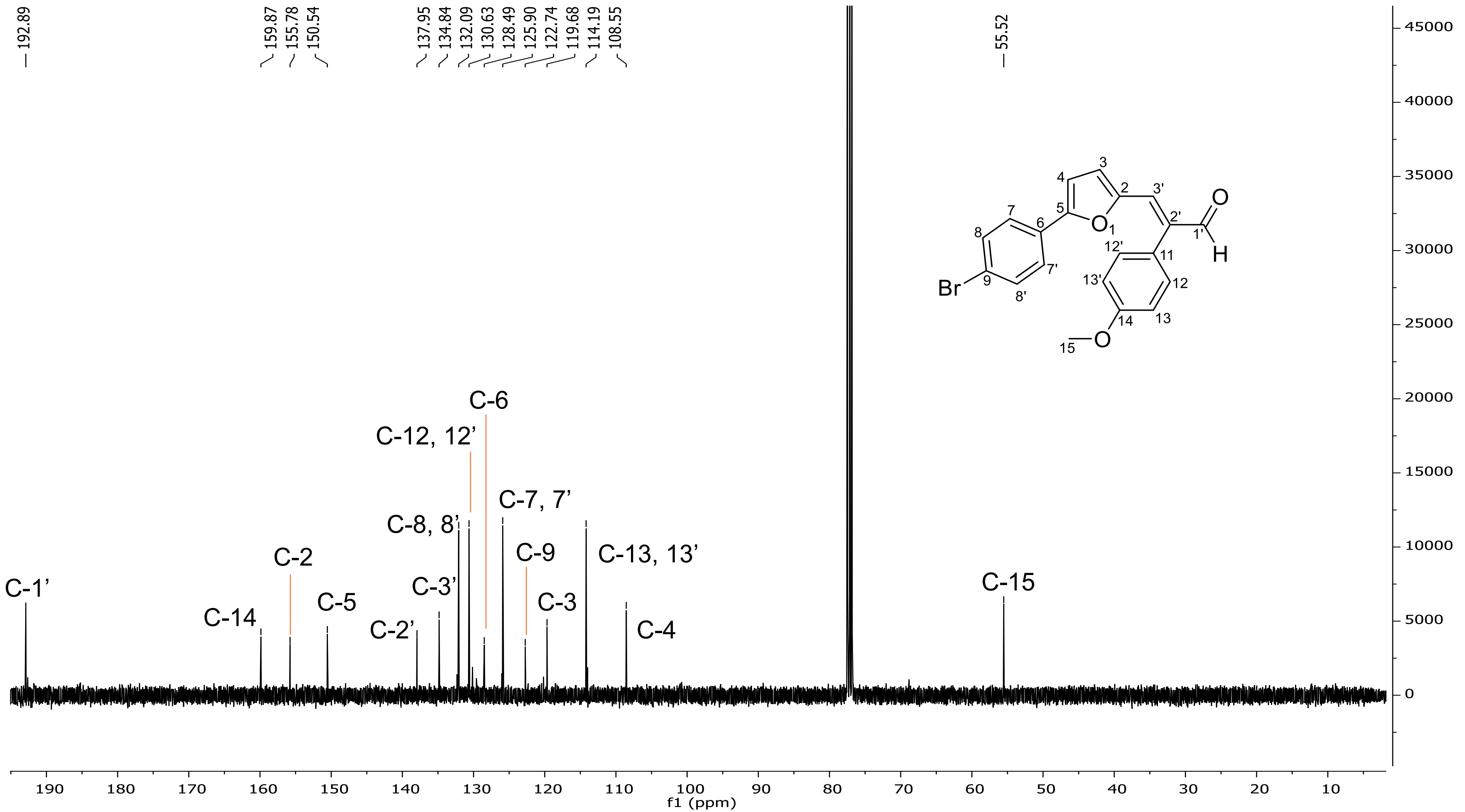

Figure S33: ^{13}C NMR (101 MHz, $CDCl_3$) of compound **7**.

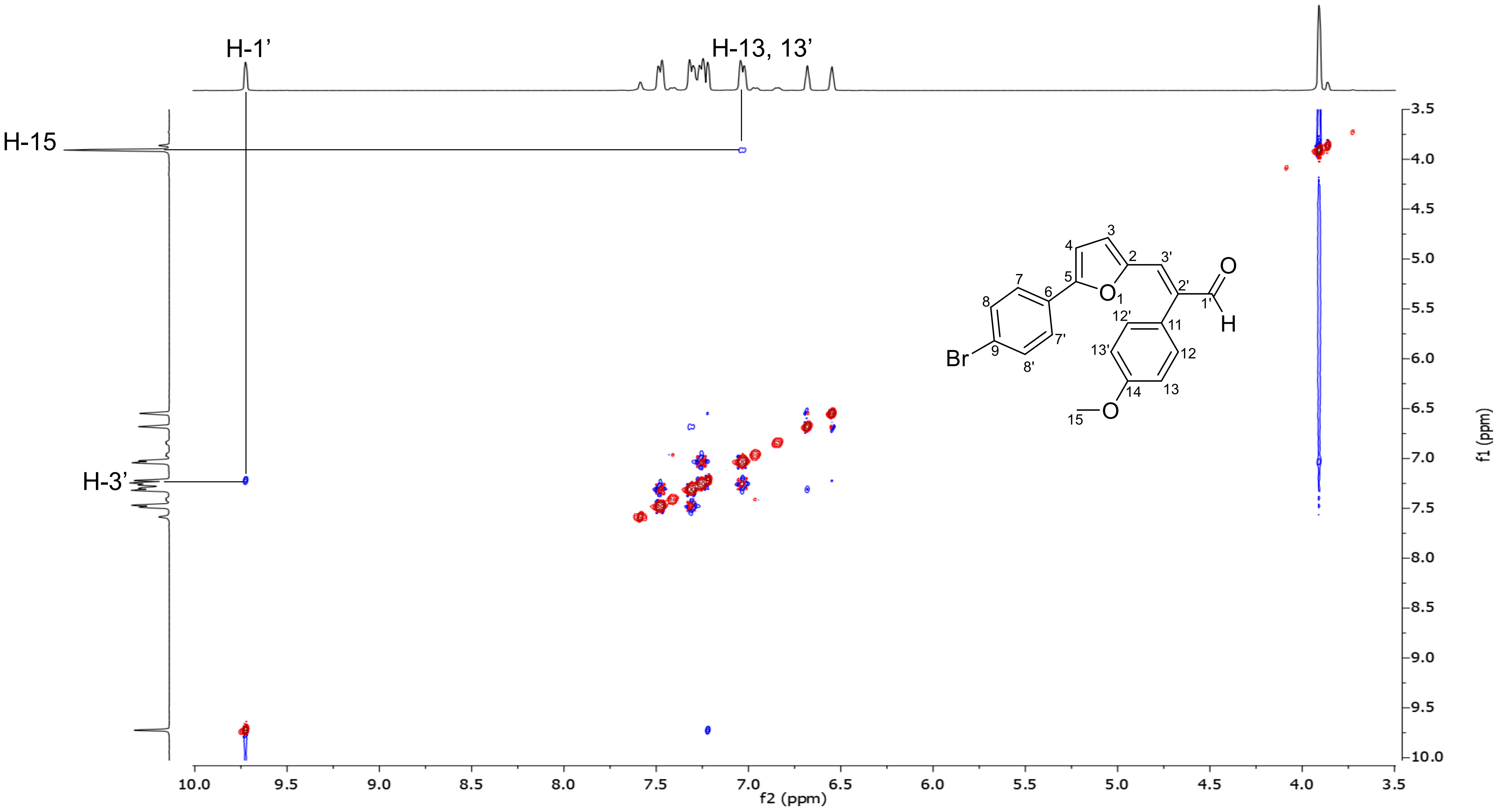

Figure S34: $^{1}H,^{1}H$-NOESY (400 MHz, $CDCl_3$) of compound **7**.

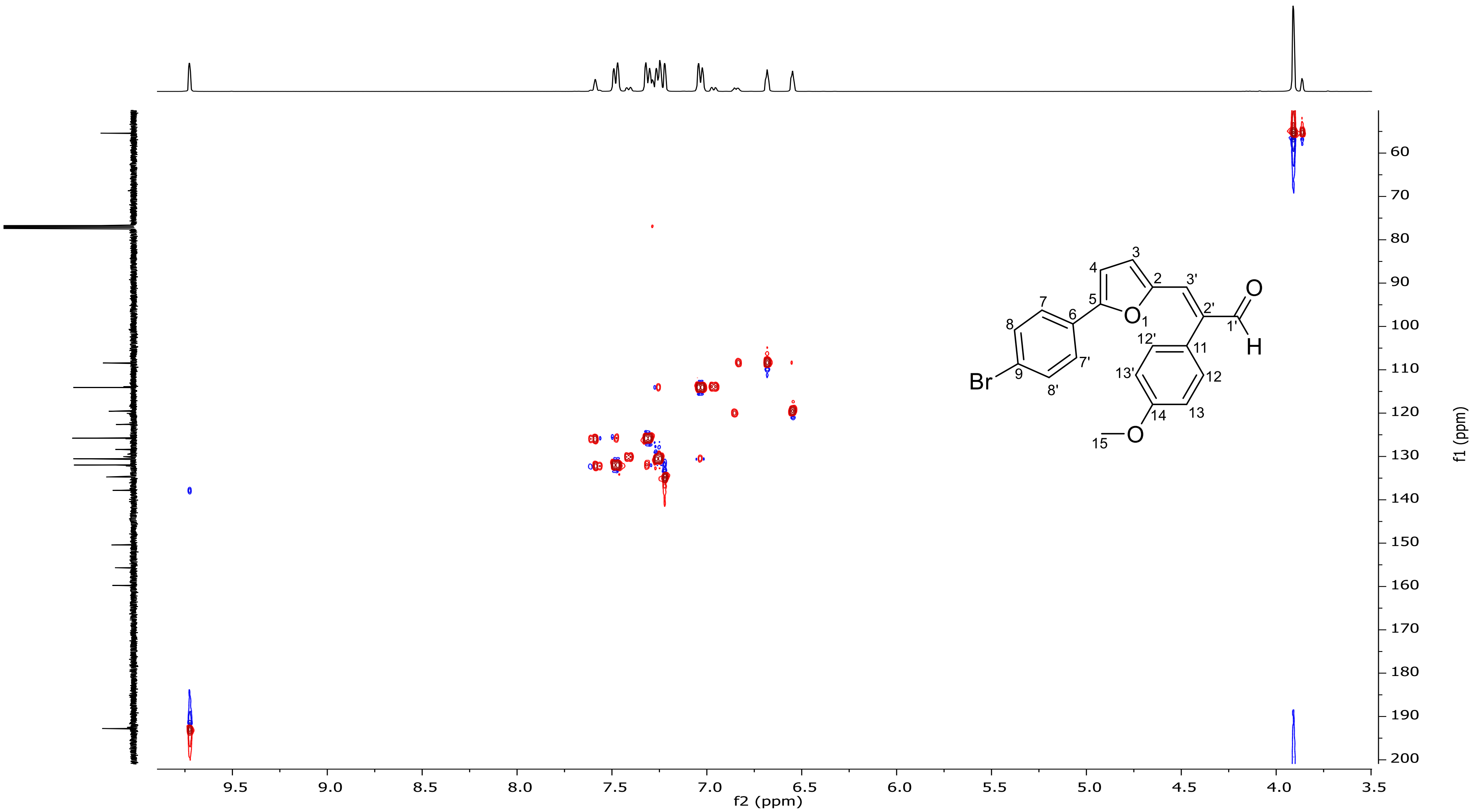

Figure S35: $^{1}H,^{13}C$-HSQC (400, 101 MHz, $CDCl_3$) of compound **7**.

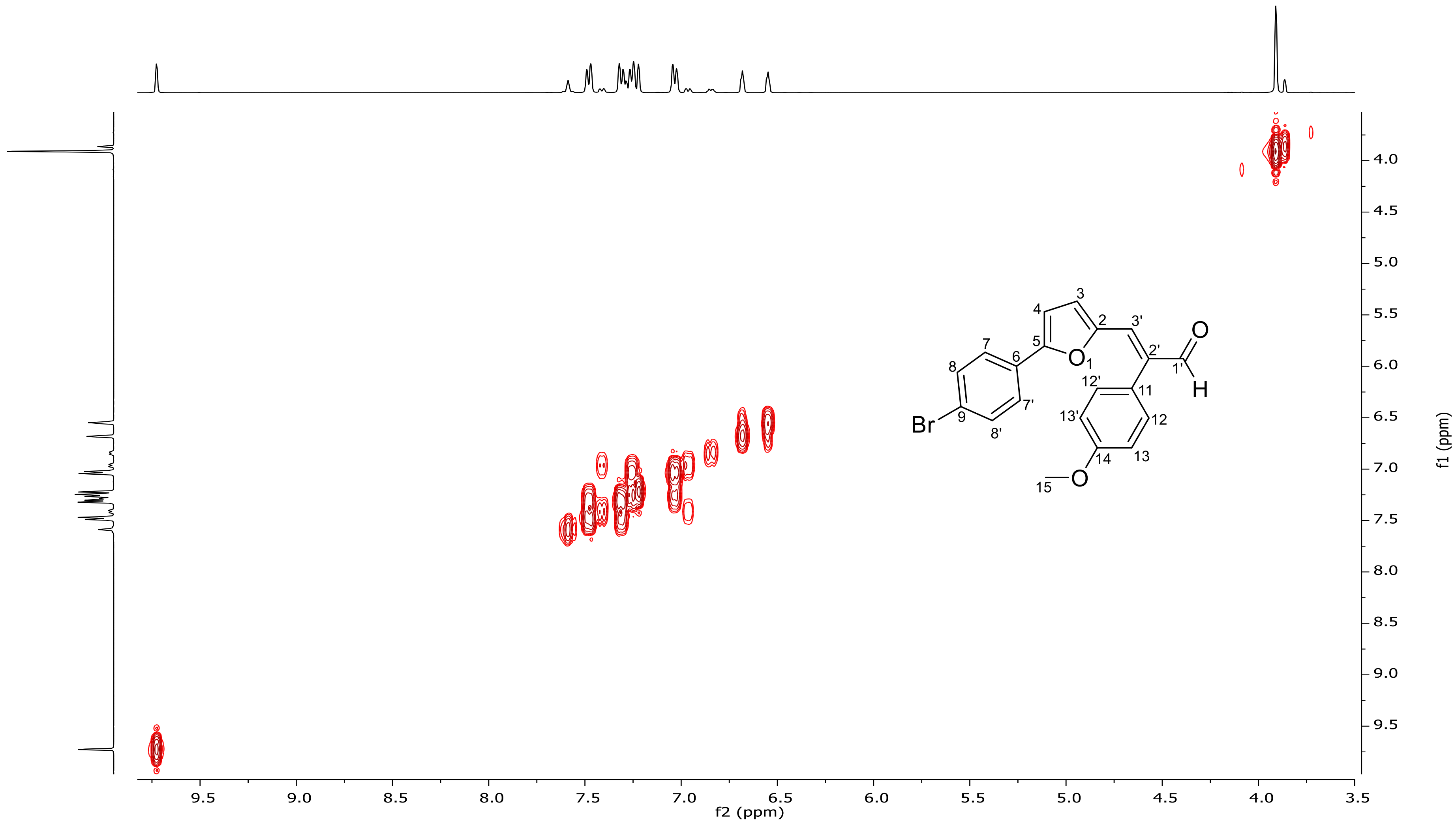

Figure S36: $^{1}H,^{1}H$-COSY (400 MHz, $CDCl_3$) of compound **7**.

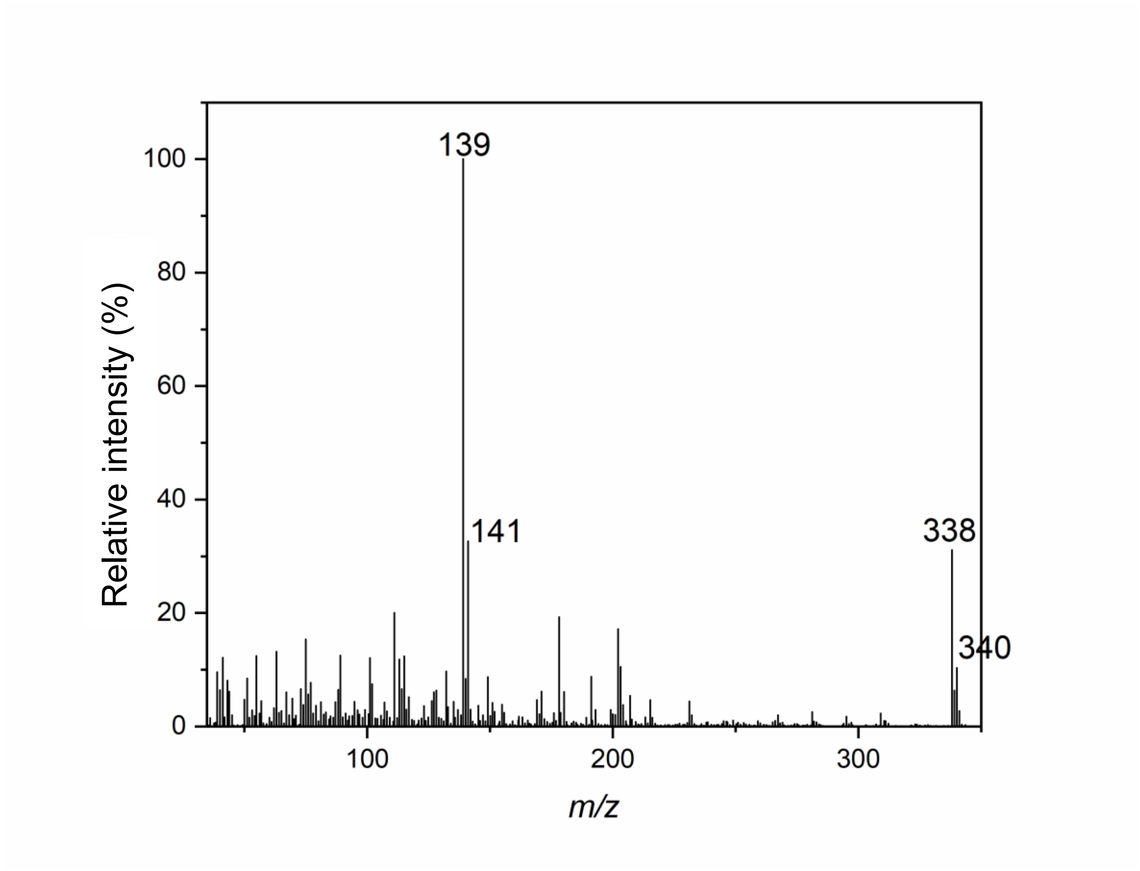

Figure S37: Mass Spectrum(IE, 70 eV) of compound **8**.

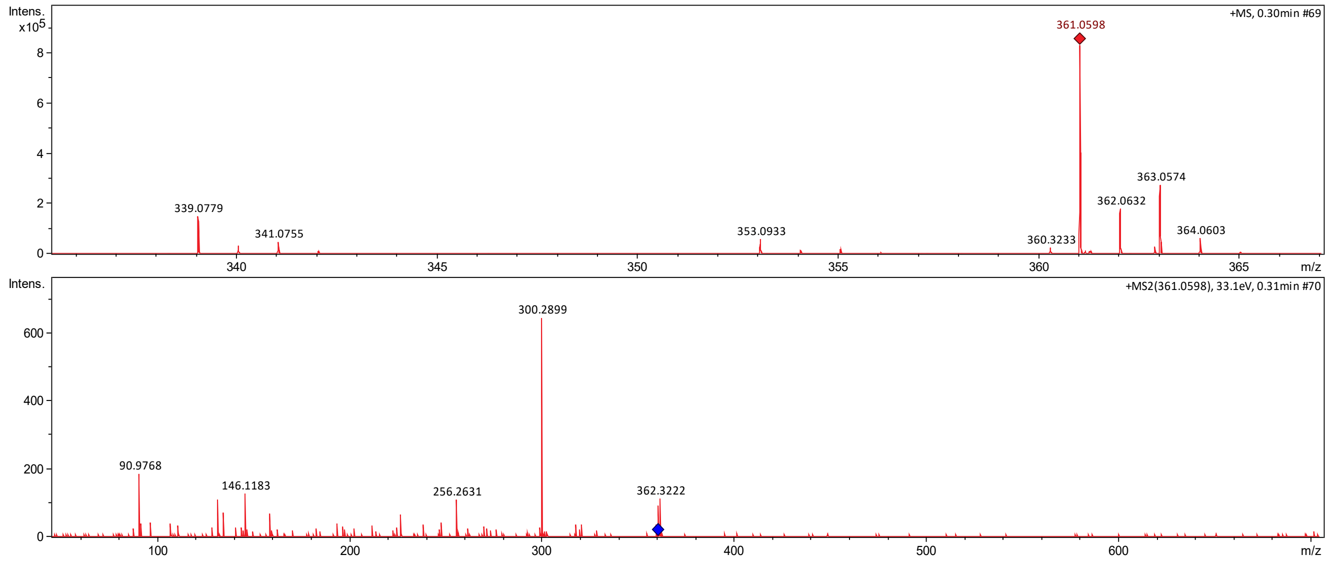

Figure S38: High-resolution mass spectrum (HRMS, ESI) of compound **8**.

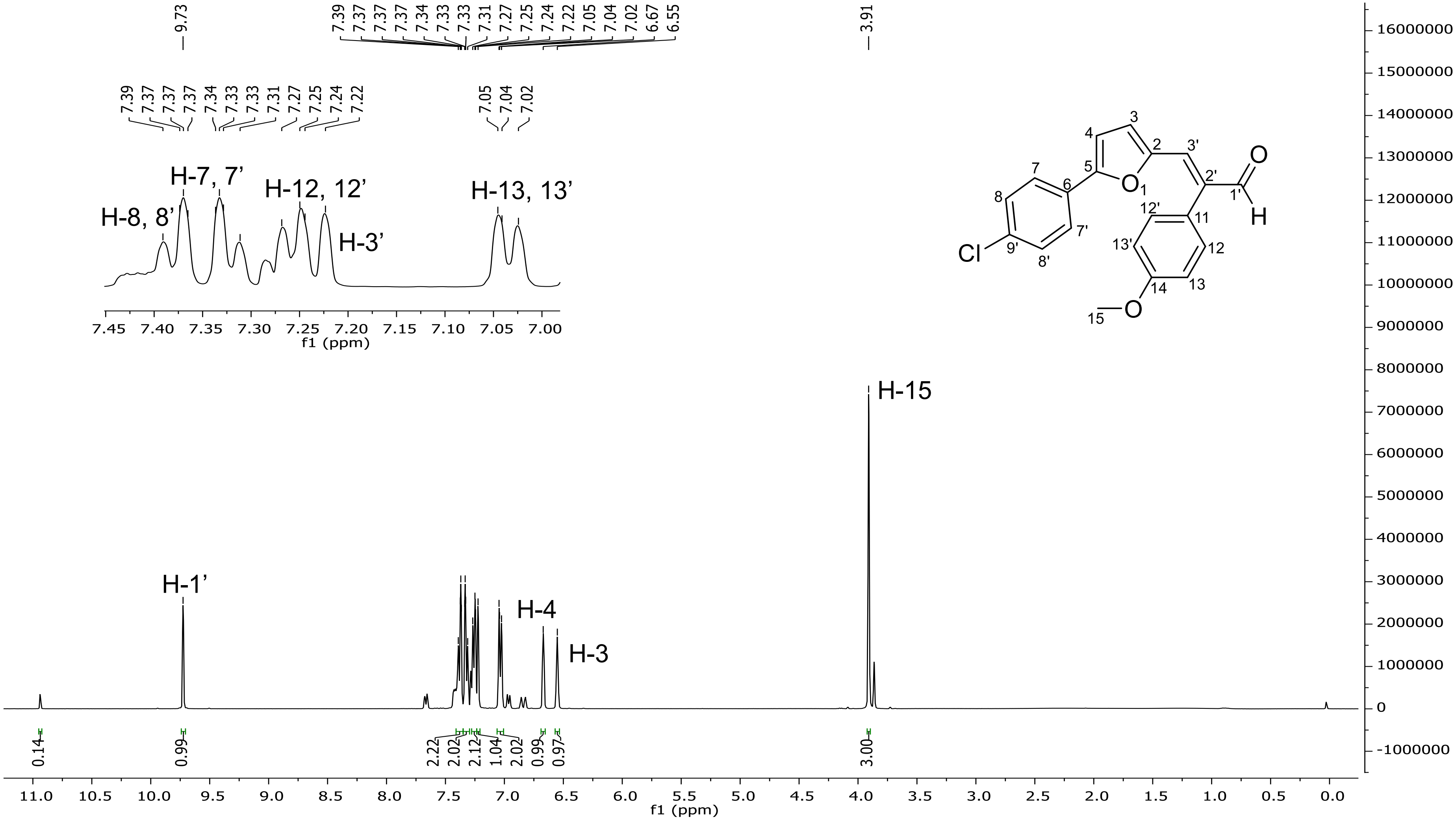

Figure S39: ^{1}H NMR (300 MHz, $CDCl_3$) of compound **8**.

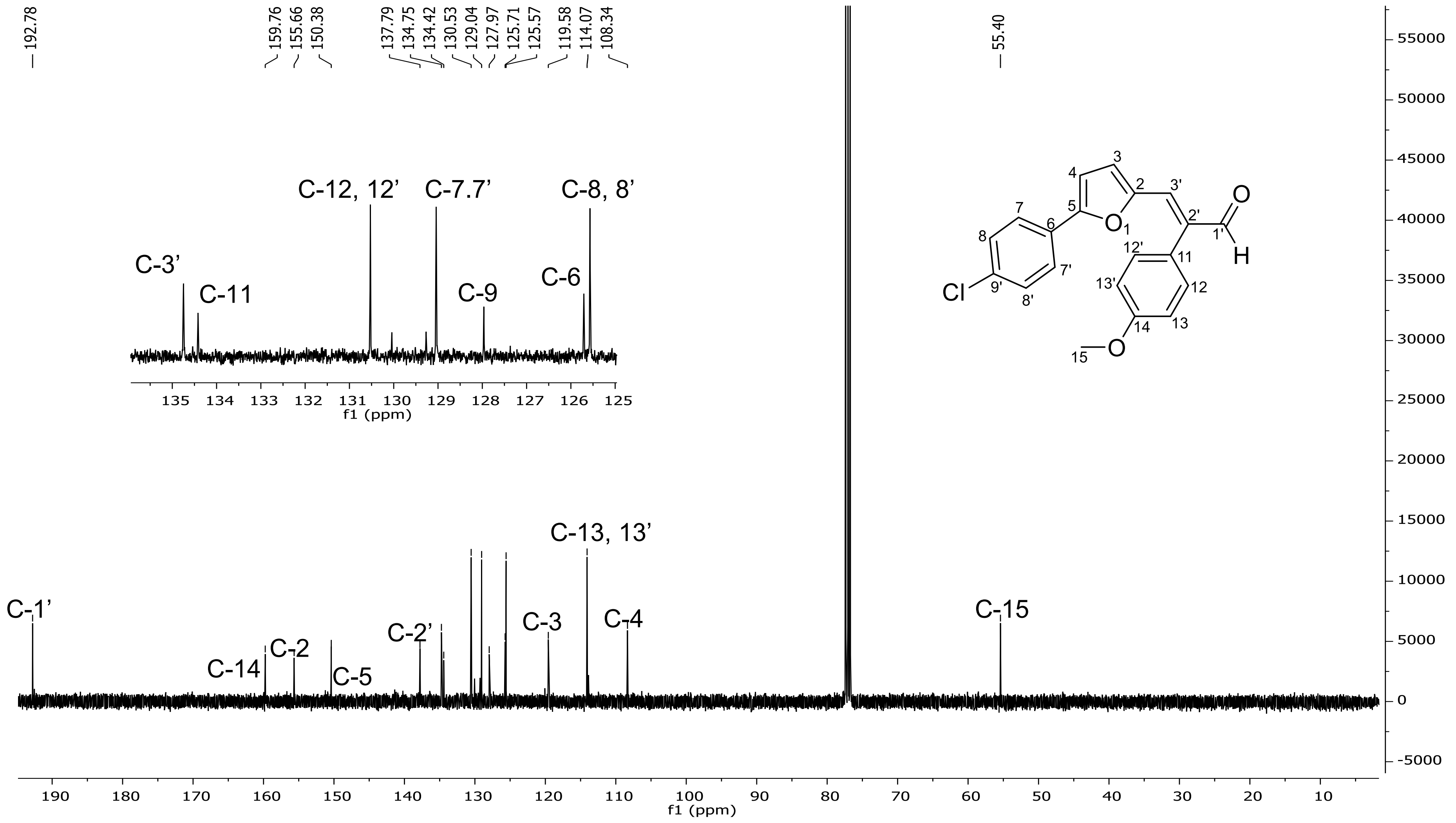

Figure S40: ^{13}C NMR (101 MHz, $CDCl_3$) of compound **8**.

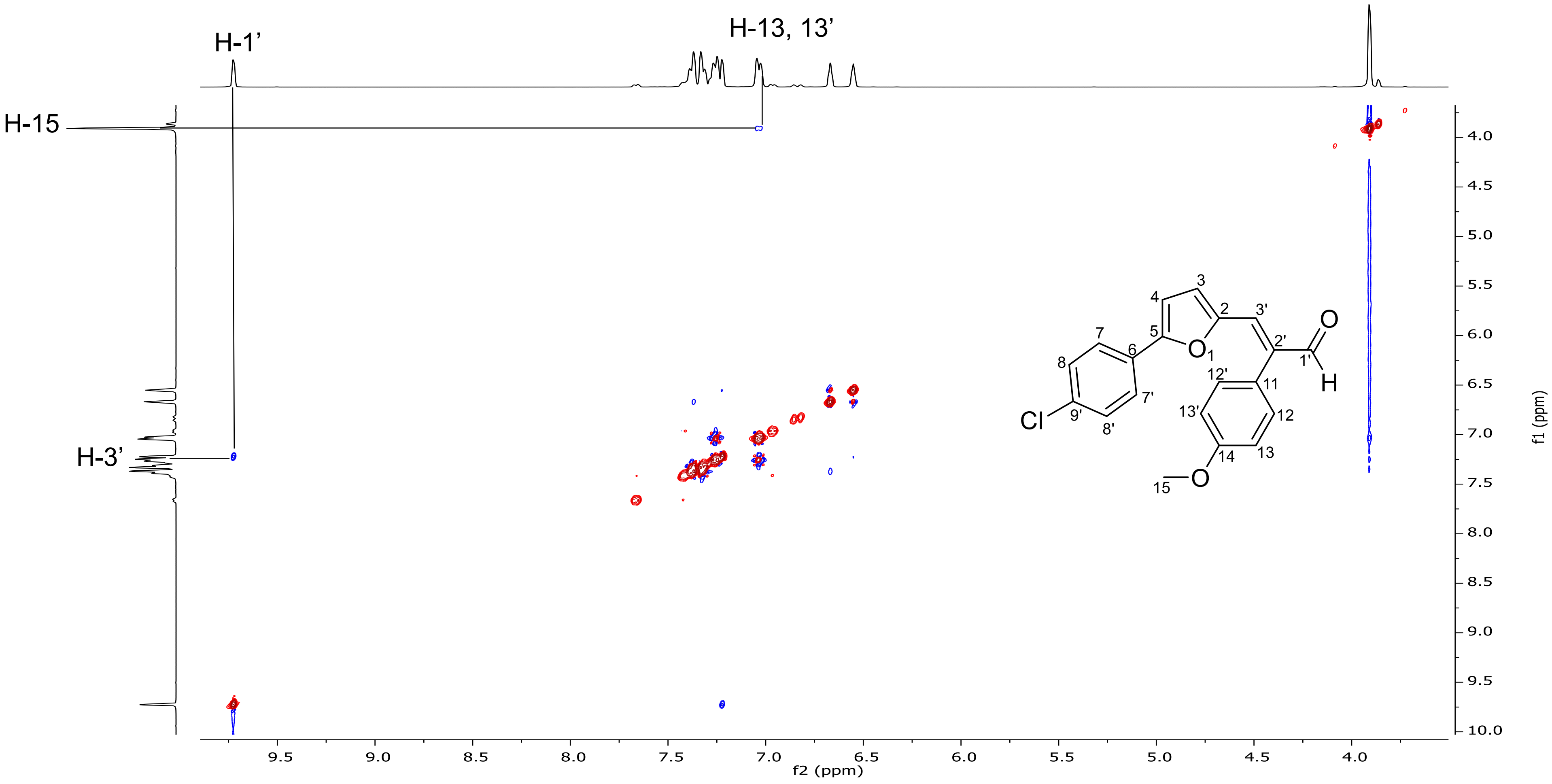

Figure S41: ^{1}H,^{1}H-NOESY (400 MHz, $CDCl_3$) of compound **8**.

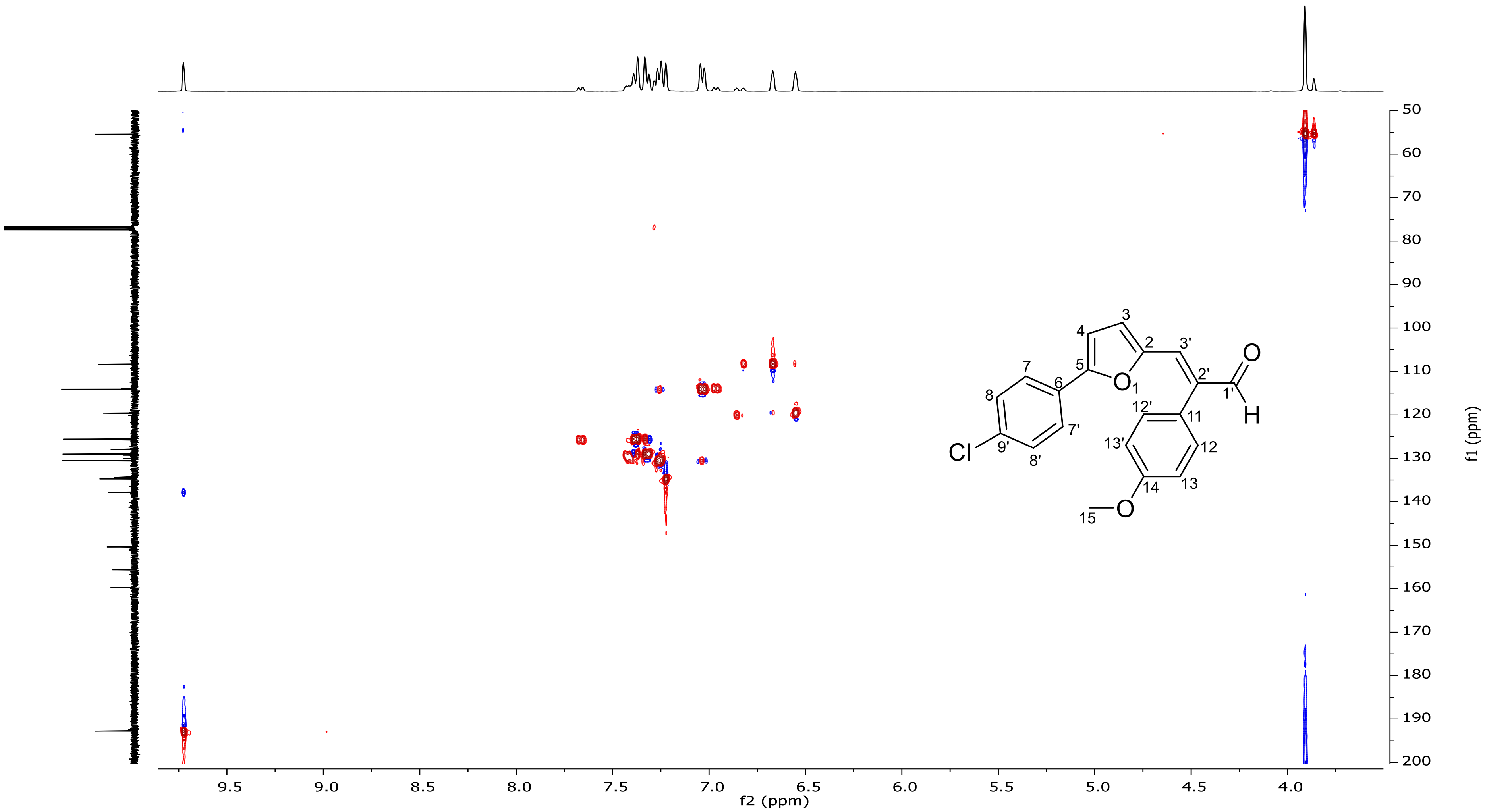

Figure S42: $^{1}H,^{13}C$-HSQC (400, 101 MHz, $CDCl_3$) of compound **8**.

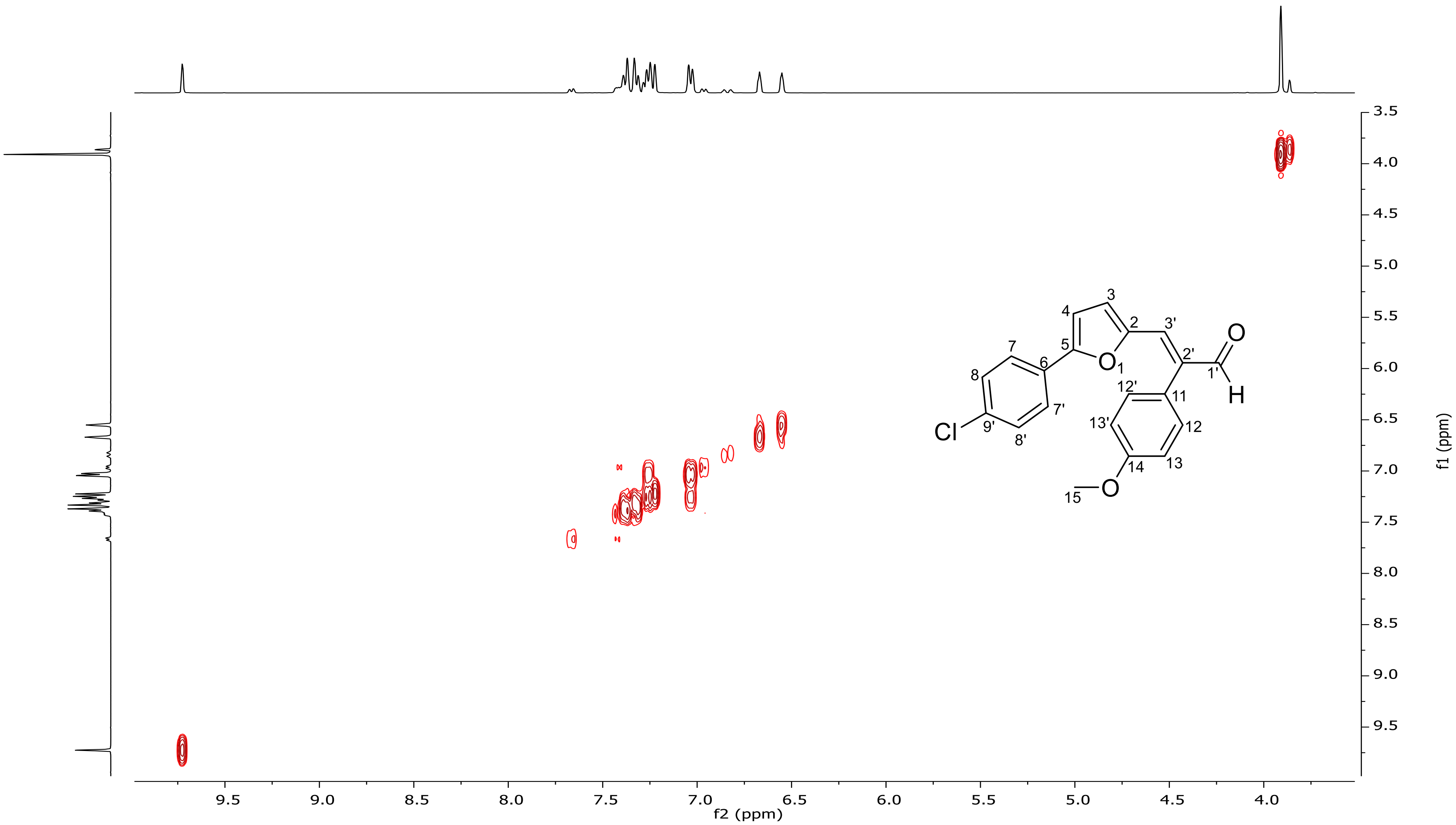

Figure S43: ^{1}H,^{1}H-COSY (400 MHz, $CDCl_3$) of compound **8**.

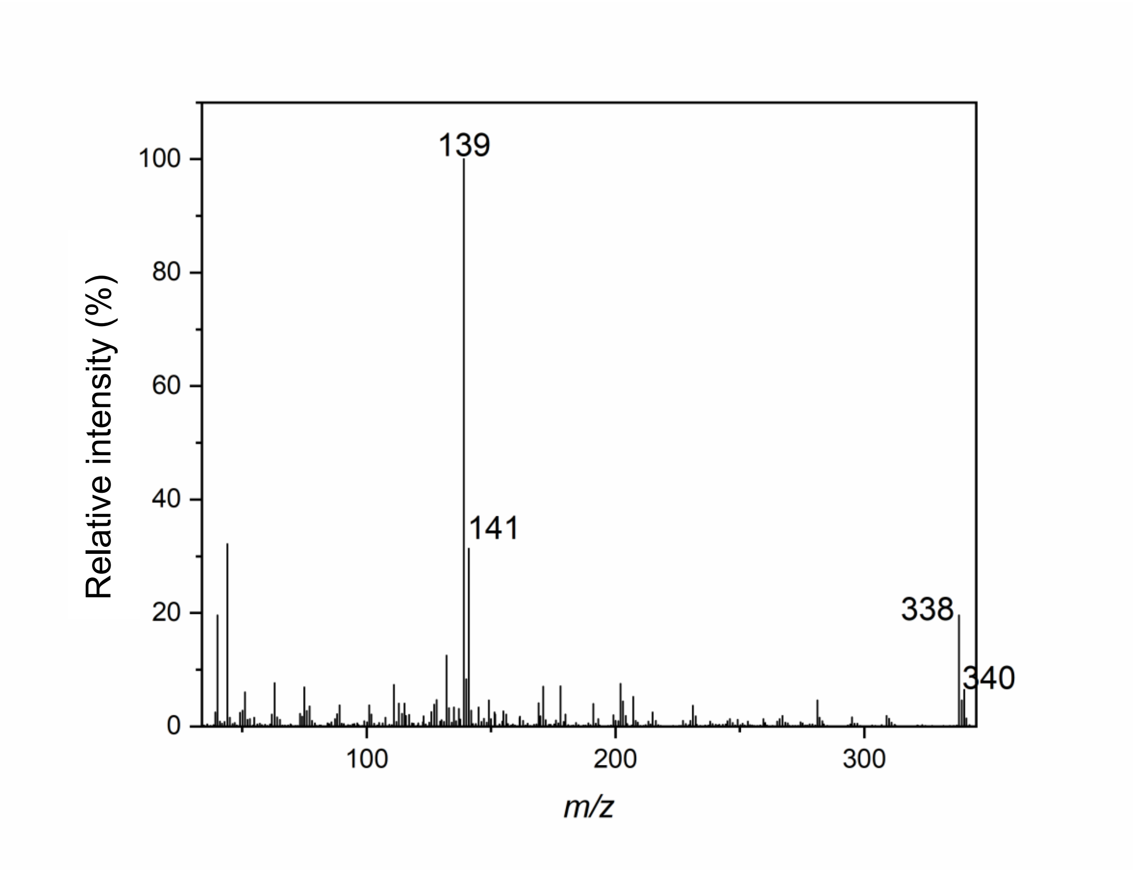

Figure S44: Mass Spectrum(IE, 70 eV) of compound **9**.

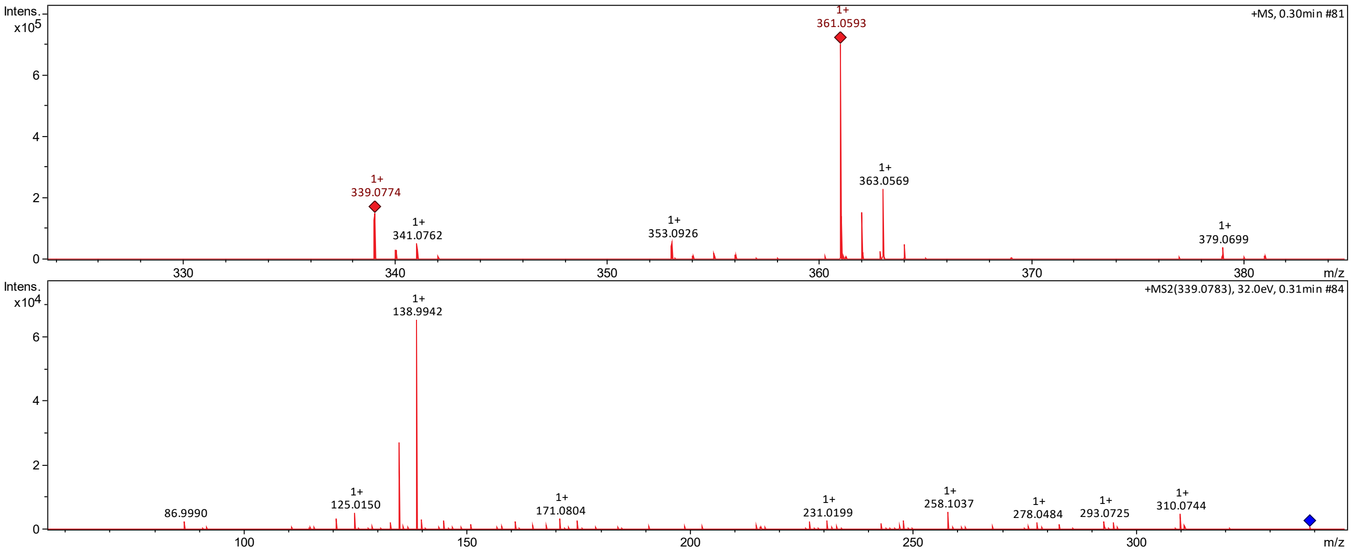

Figure S45: High-resolution mass spectrum (HRMS, ESI) of compound **9**.

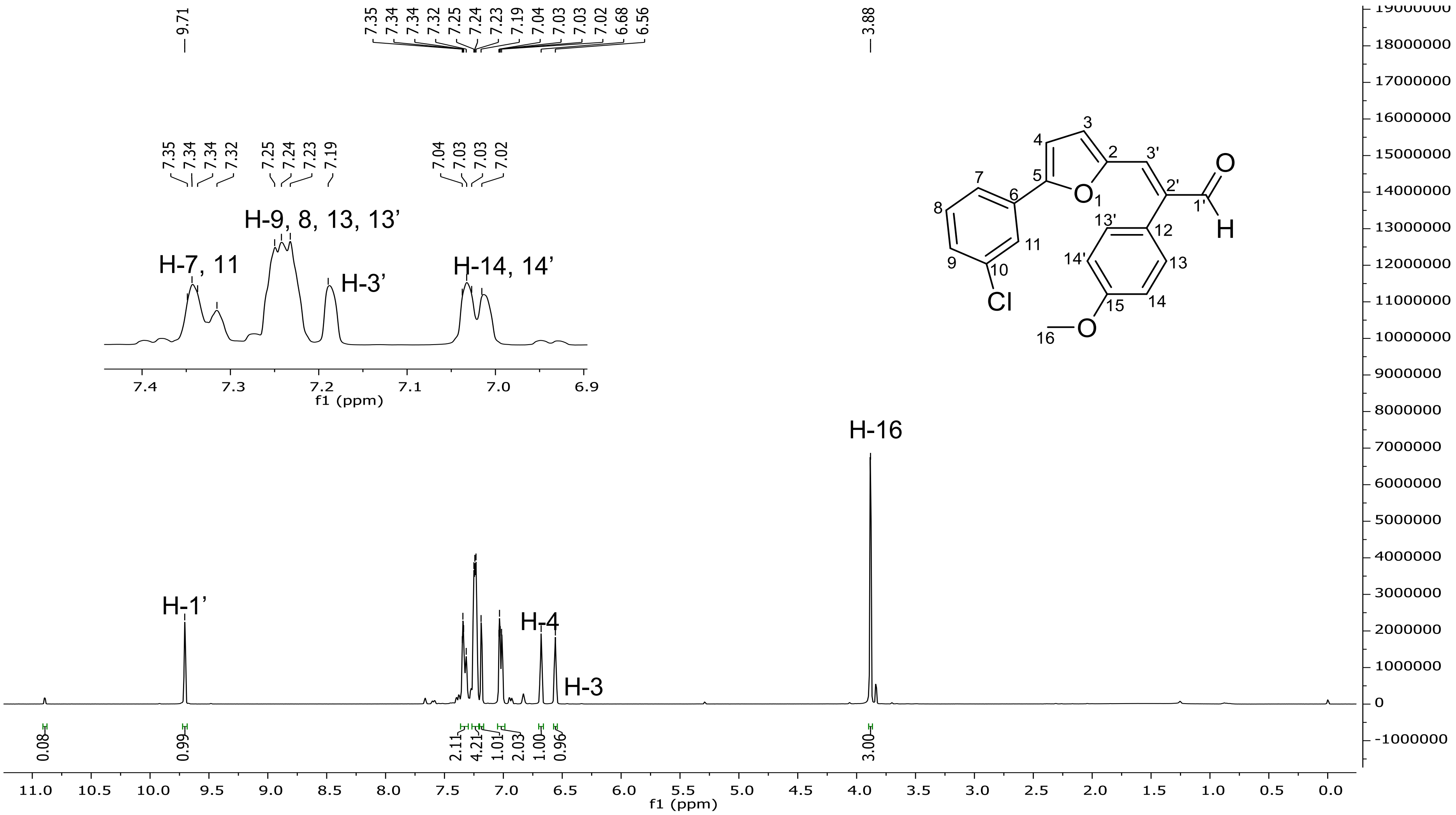

Figure S46: ^{1}H NMR (300 MHz, $CDCl_3$) of compound **9**.

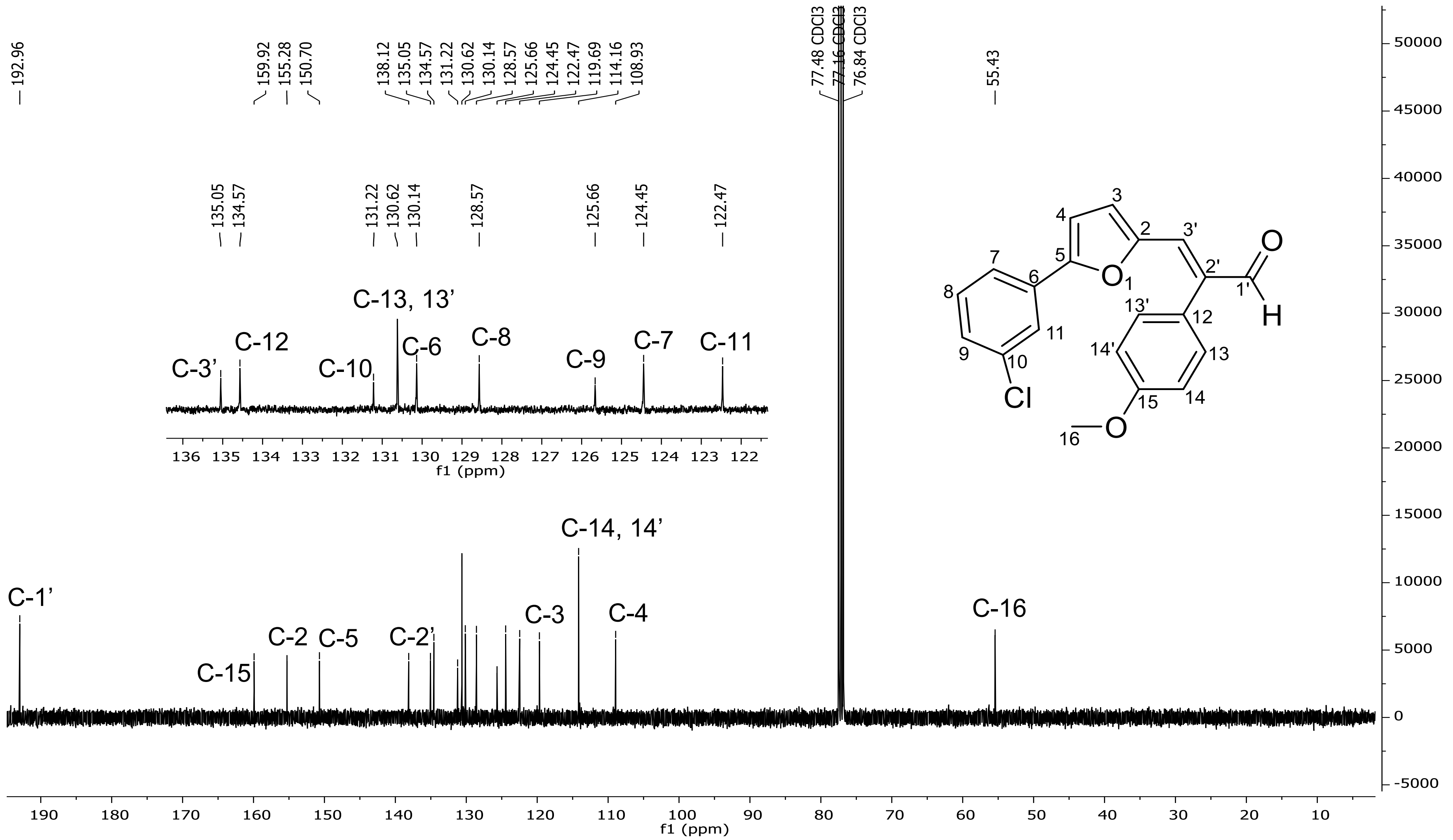

Figure S47: ^{13}C NMR (101 MHz, $CDCl_3$) of compound **9**

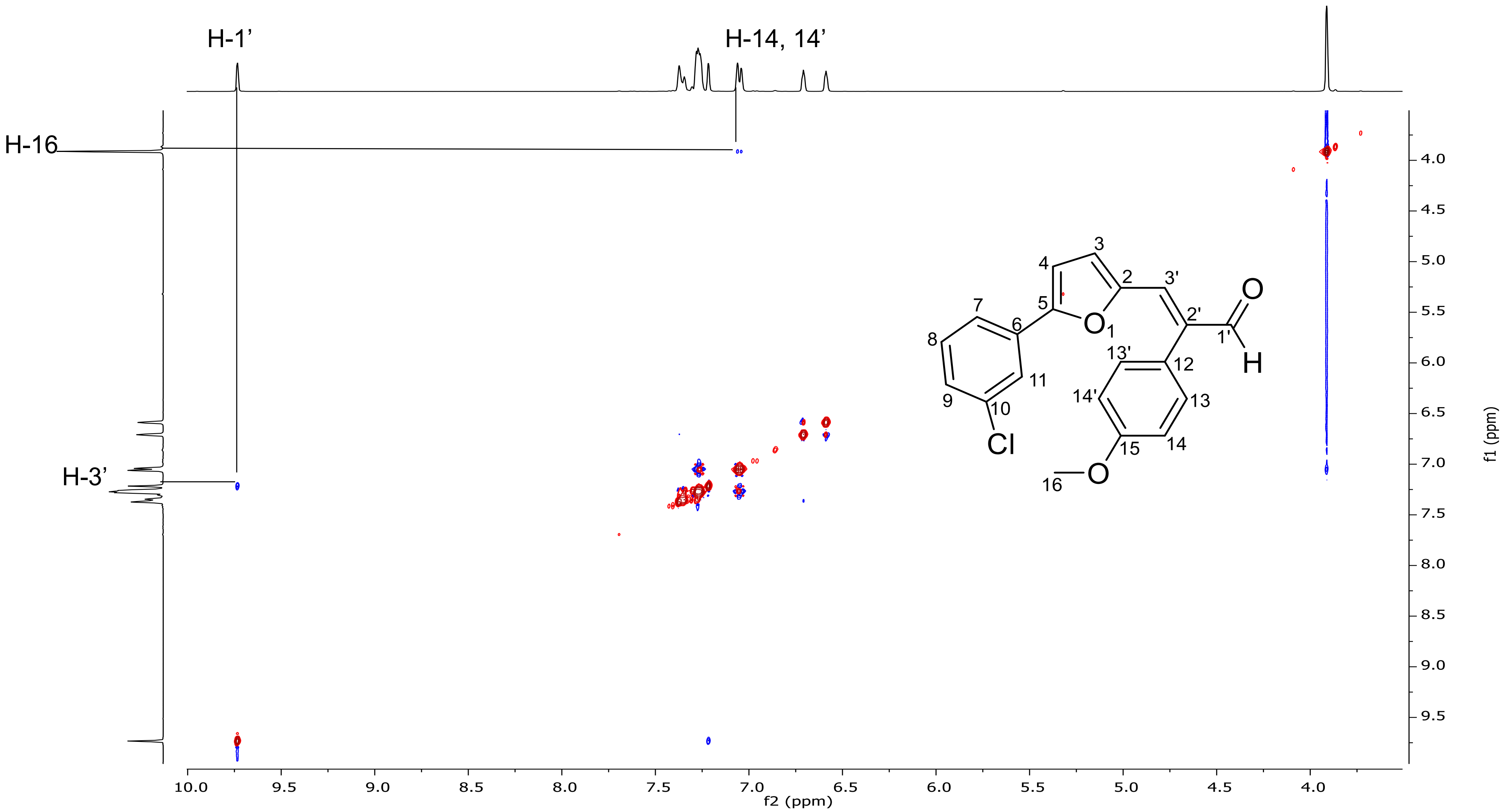

Figure S48: $^{1}H,^{1}H$-NOESY (400 MHz, $CDCl_3$) of compound **9**.

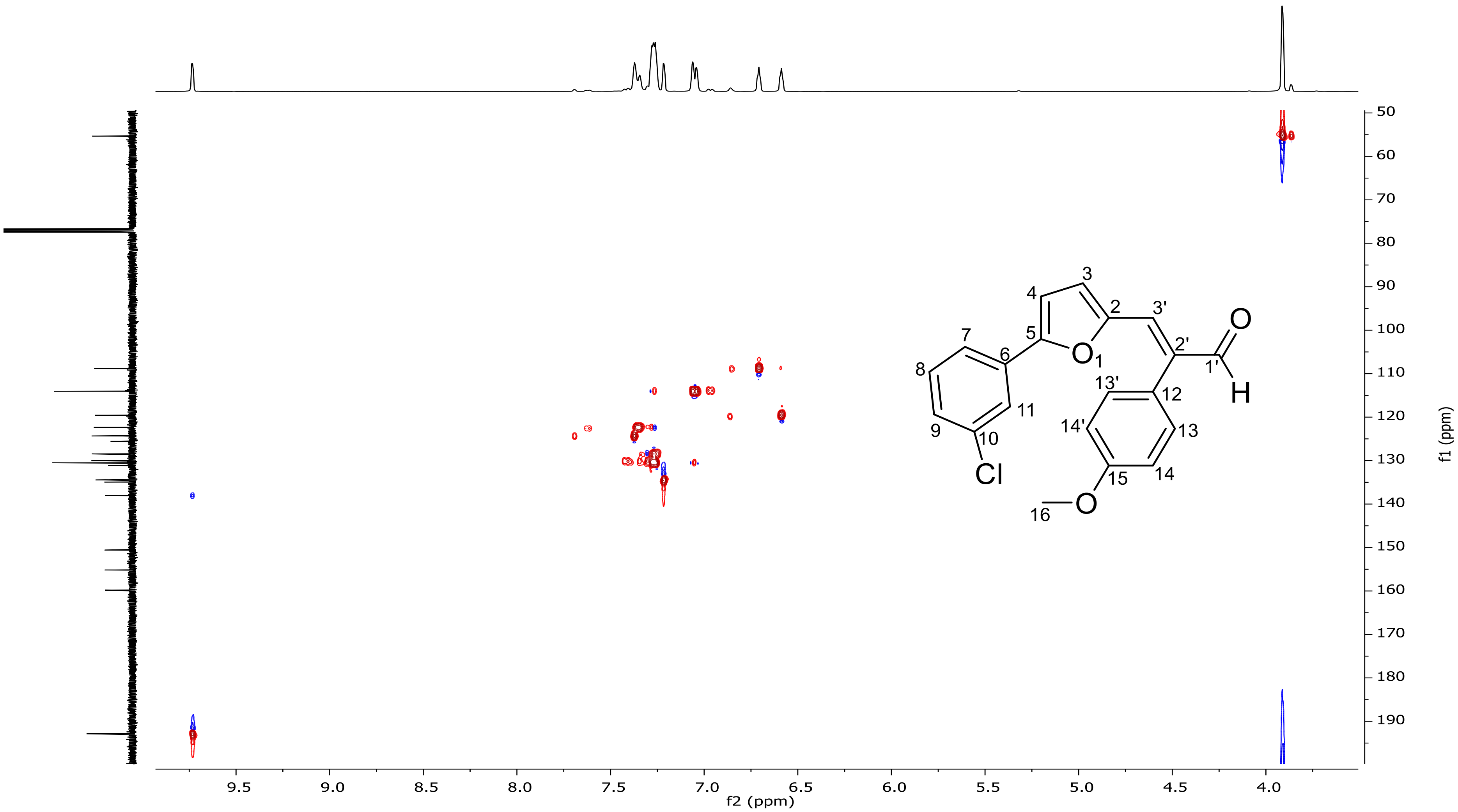

Figure S49: ^{1}H,^{13}C-HSQC (400, 101 MHz, $CDCl_3$) of compound **9**.

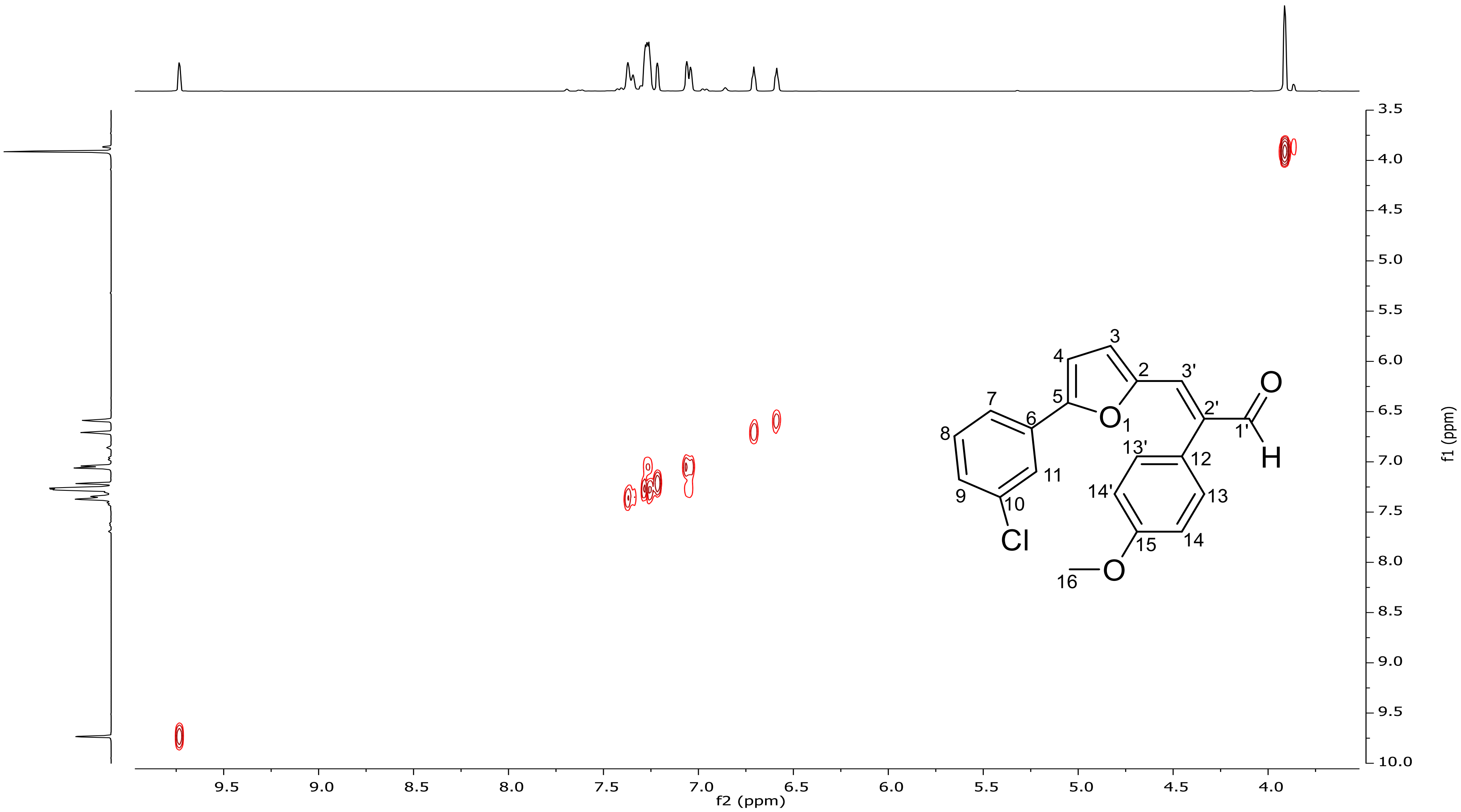

Figure S50: $^1H,^1H$-COSY (400 MHz, $CDCl_3$) of compound **9**.

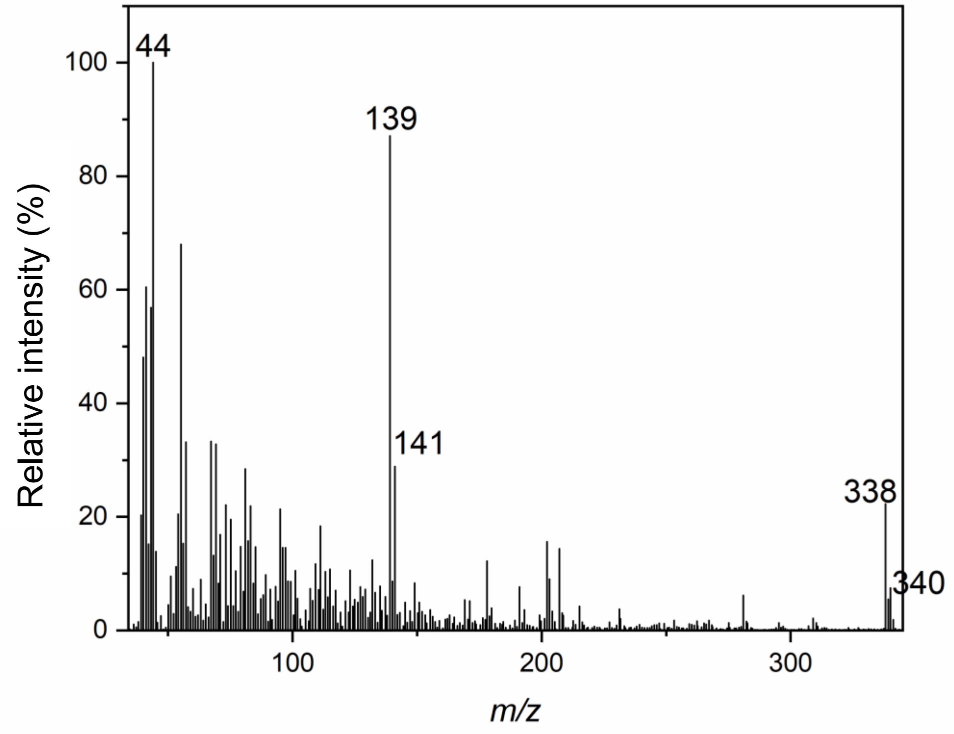

Figure S51: Mass Spectrum(IE, 70 eV) of compound **10**.

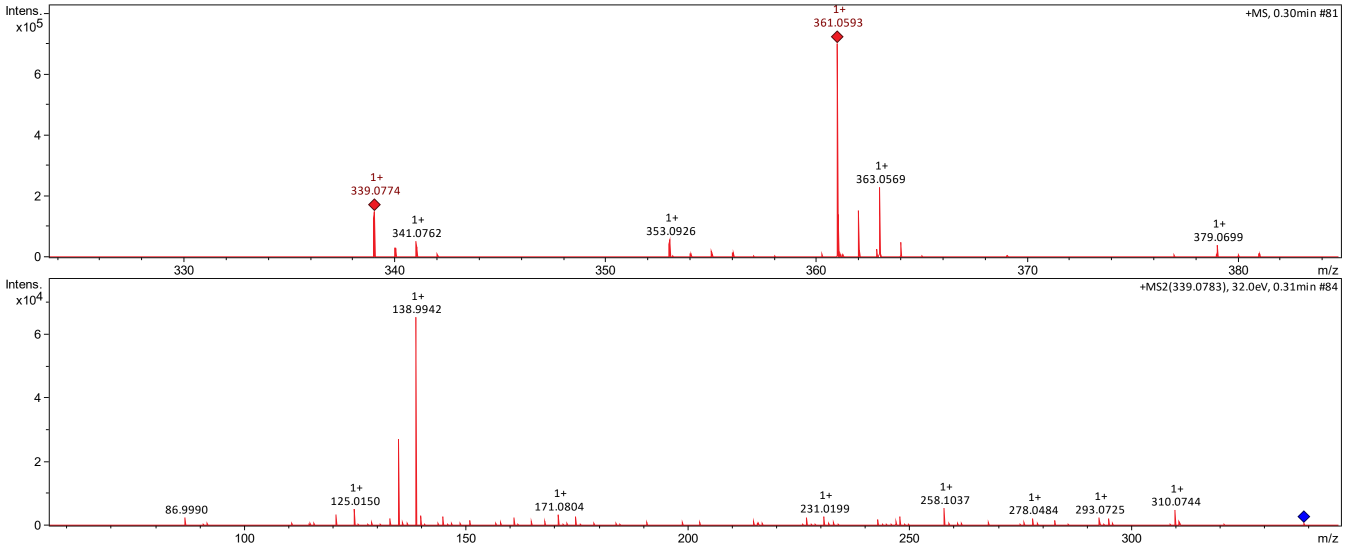

Figure S52: High-resolution mass spectrum (HRMS, ESI) of compound **10.**

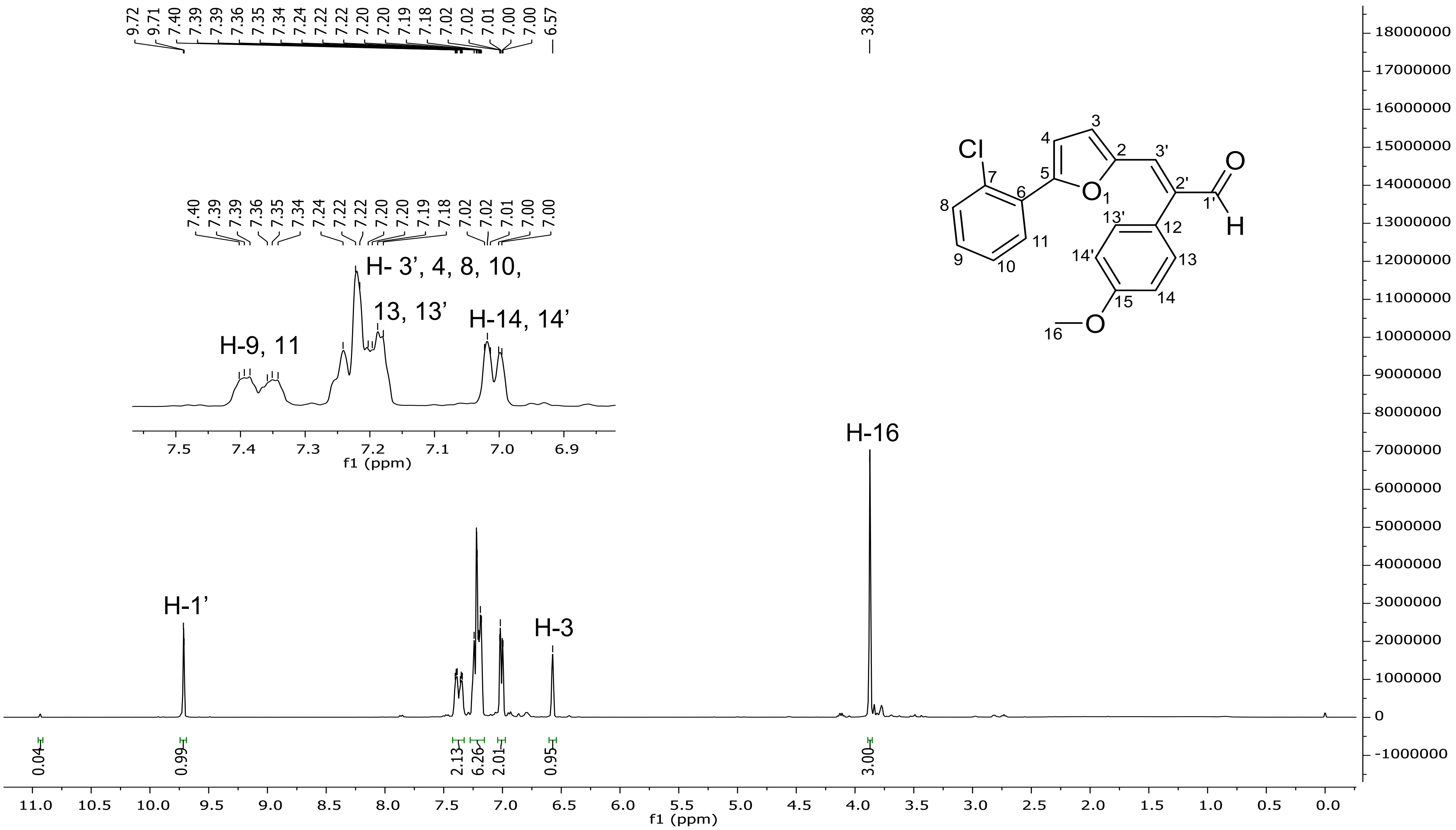

Figure S53: ^{1}H NMR (300 MHz, $CDCl_3$) of compound **10**.

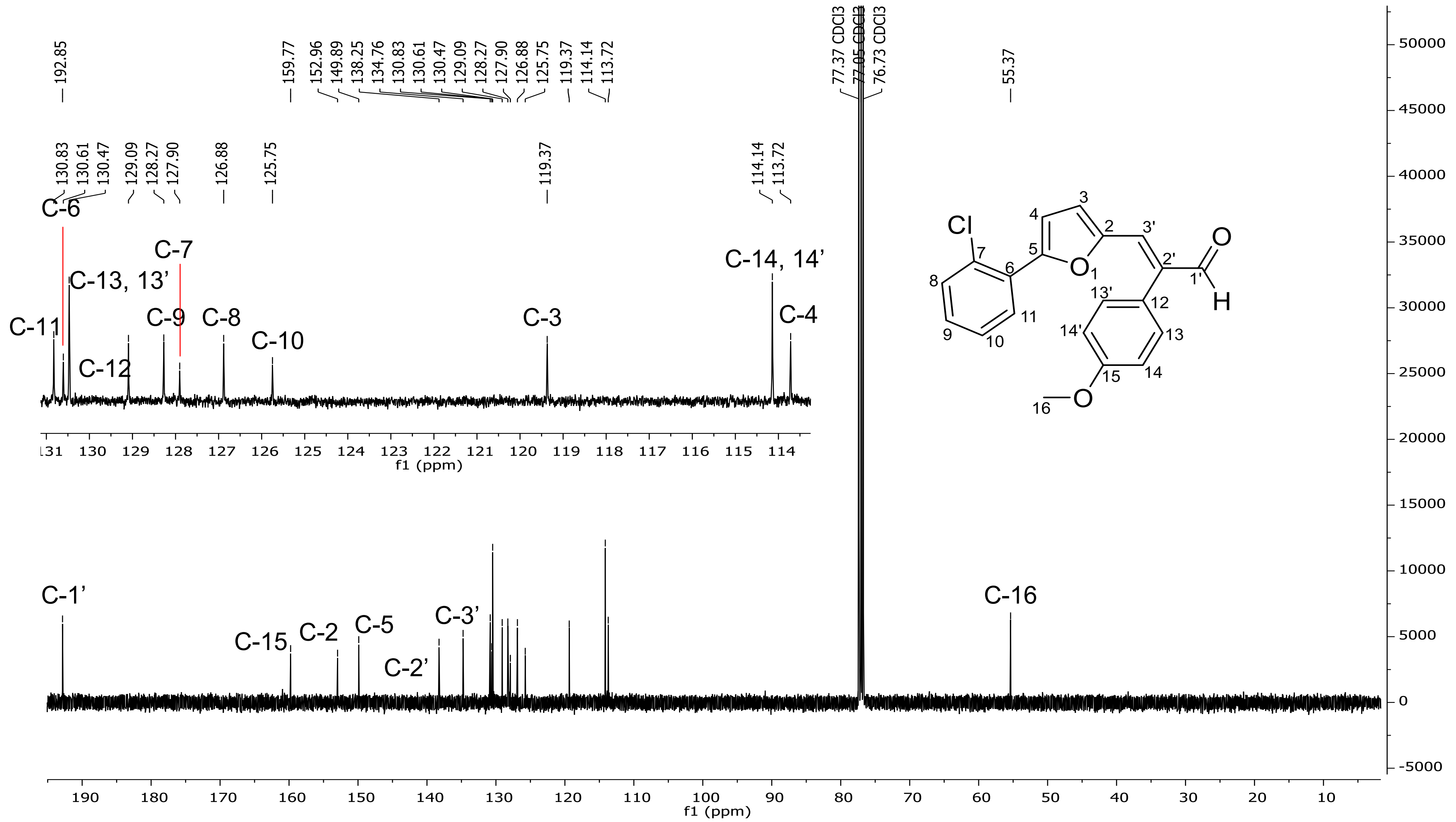

Figure S54: ^{13}C NMR (101 MHz, $CDCl_3$) of compound **10**.

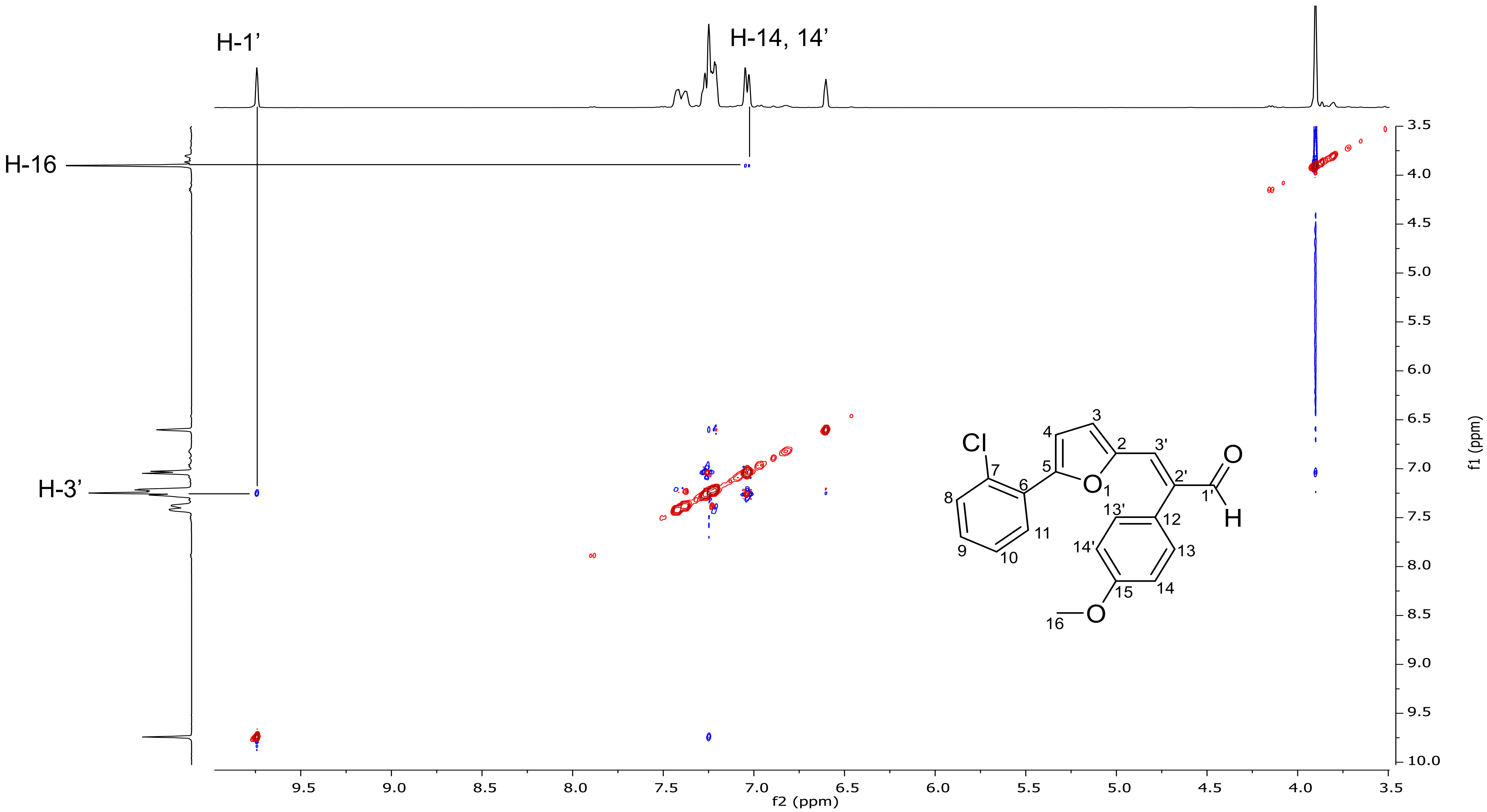

Figure S55: ^{1}H,^{1}H-NOESY (400 MHz, $CDCl_3$) of compound **10**.

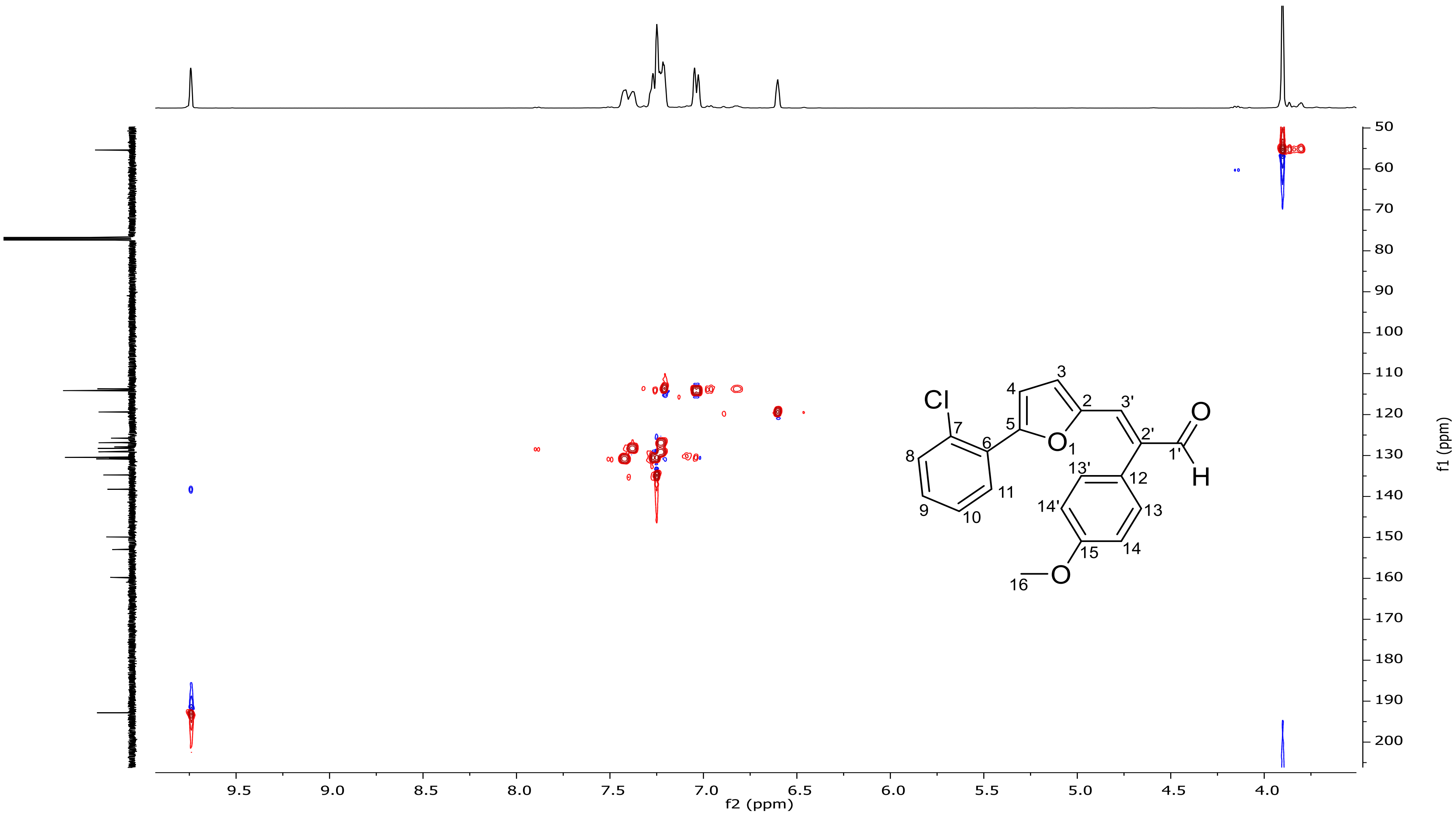

Figure S56: $^{1}H,^{13}C$-HSQC (400, 101 MHz, $CDCl_3$) of compound **10**.

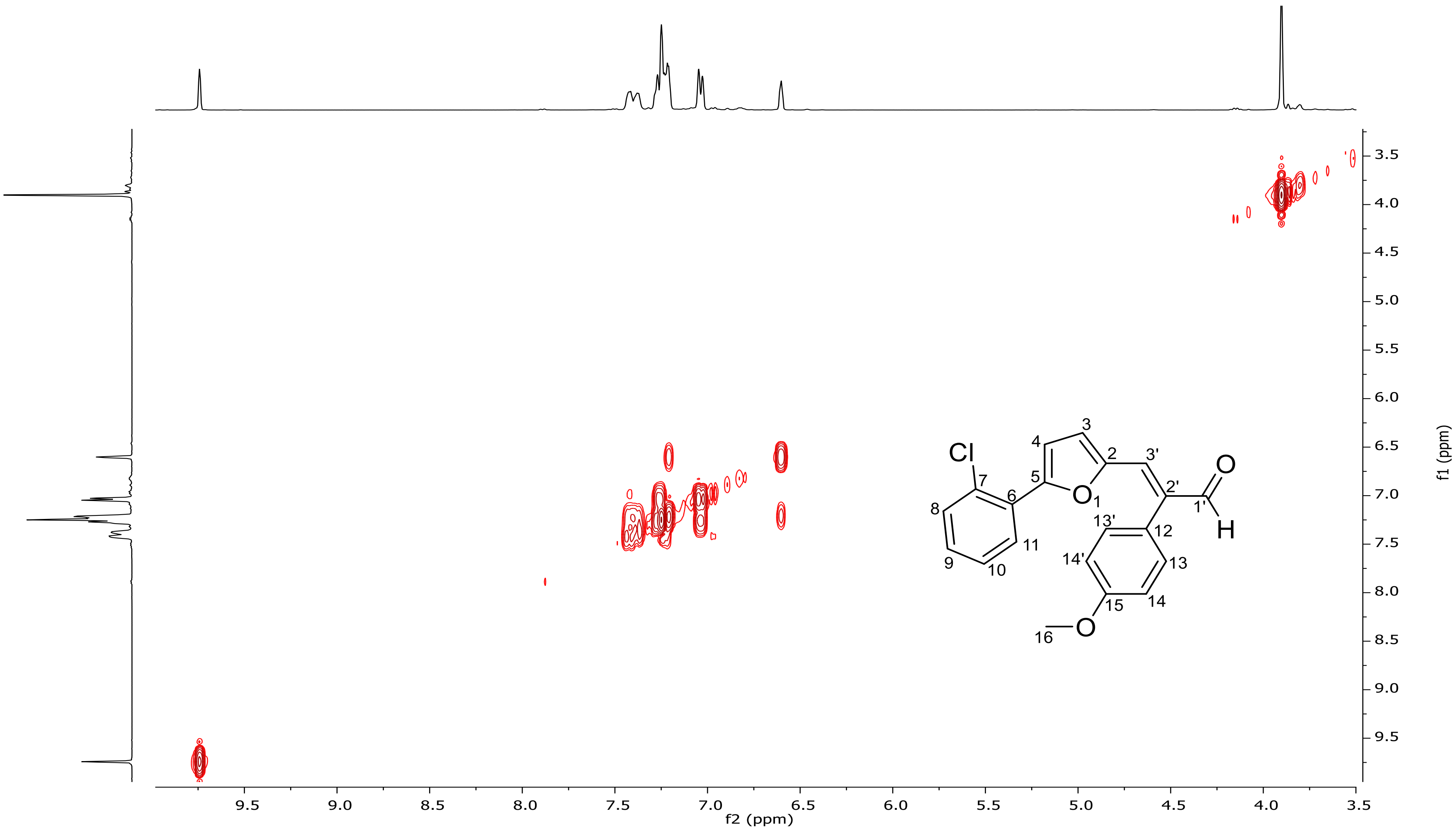

Figure S57: ^{1}H,^{1}H-COSY (400 MHz, $CDCl_3$) of compound **10**.

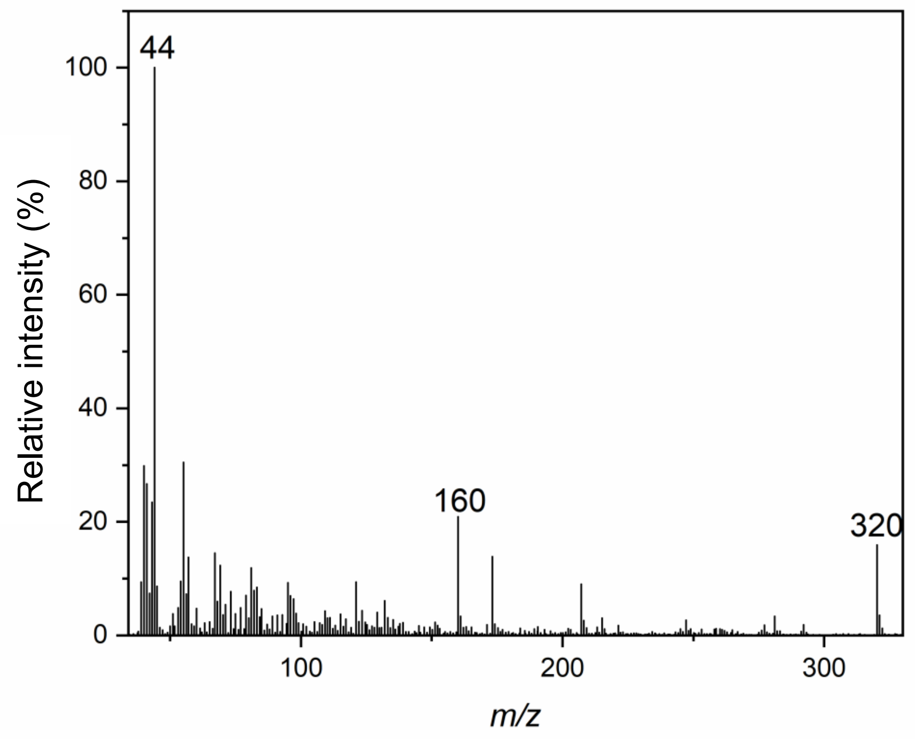

Figure S58: Mass Spectrum(IE, 70 eV) of compound **11**.

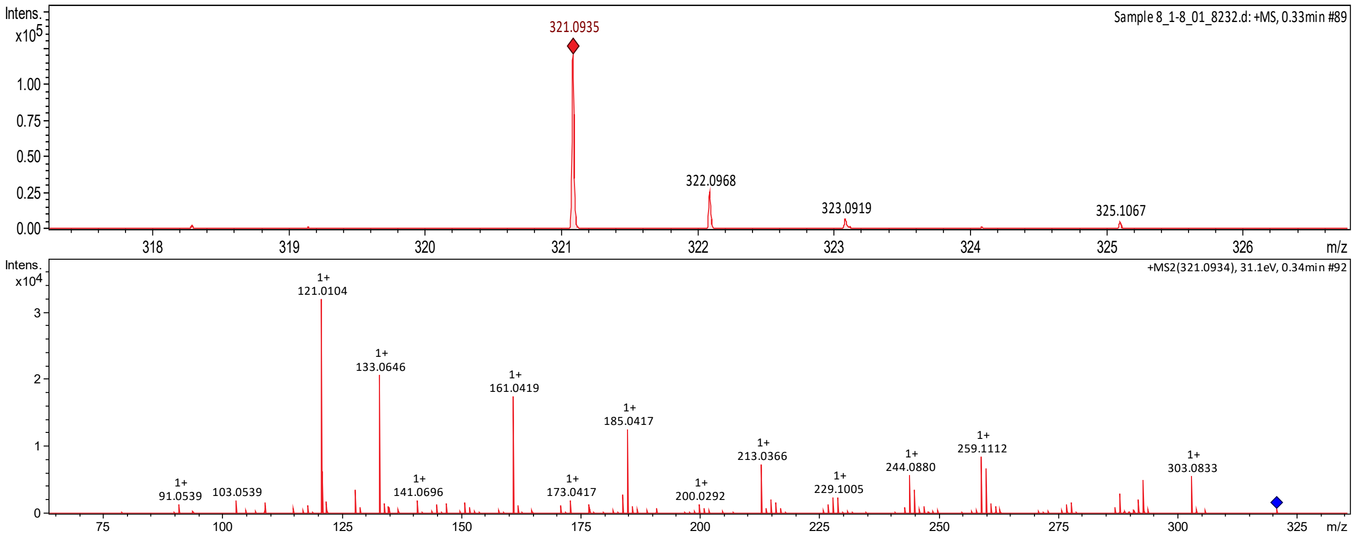

Figure S59: High-resolution mass spectrum (HRMS, ESI) of compound **11.**

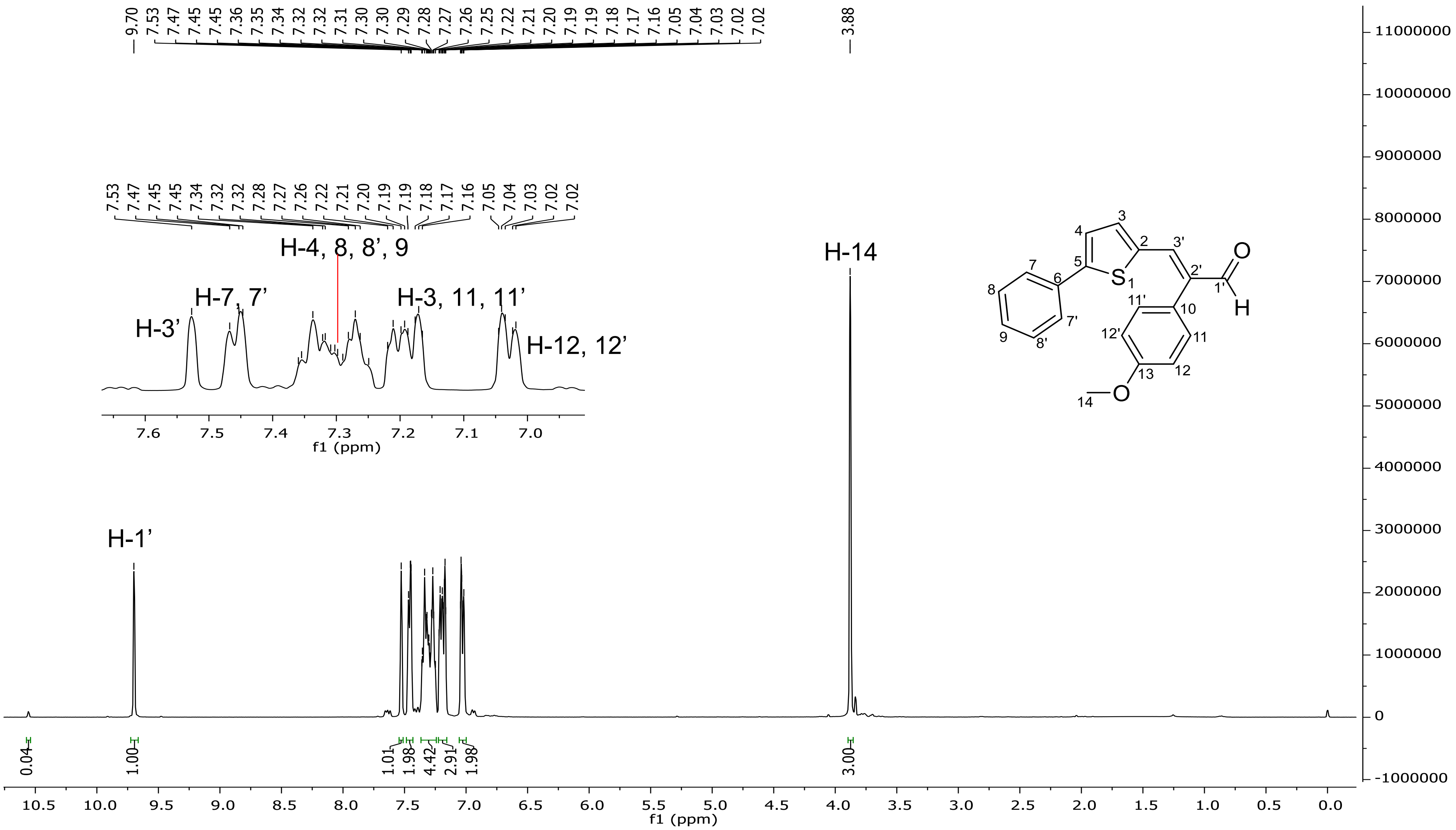

Figure S60: ^{1}H NMR (300 MHz, $CDCl_3$) of compound **11**.

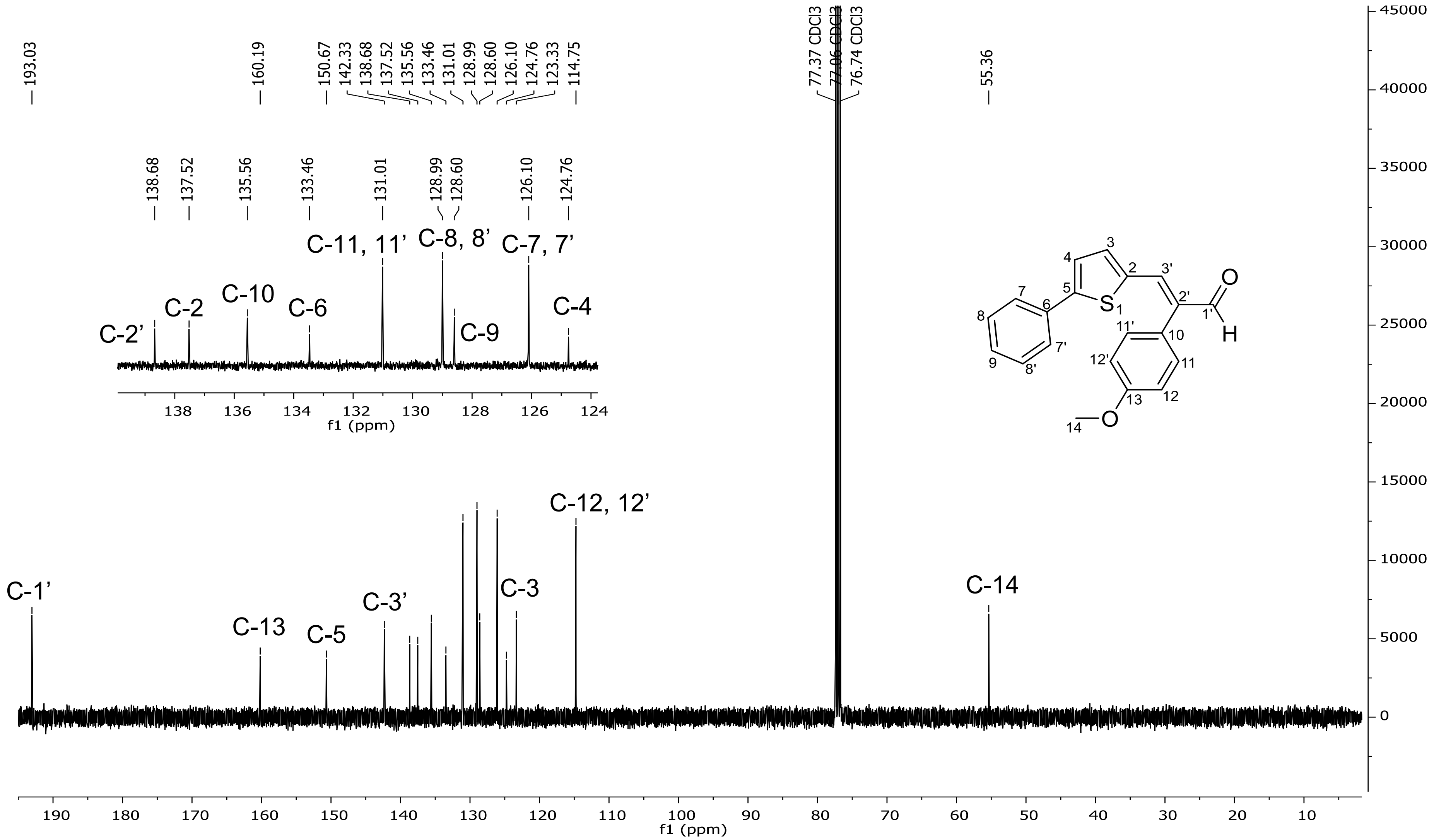

Figure S61: ^{13}C NMR (101 MHz, $CDCl_3$) of compound **11**.

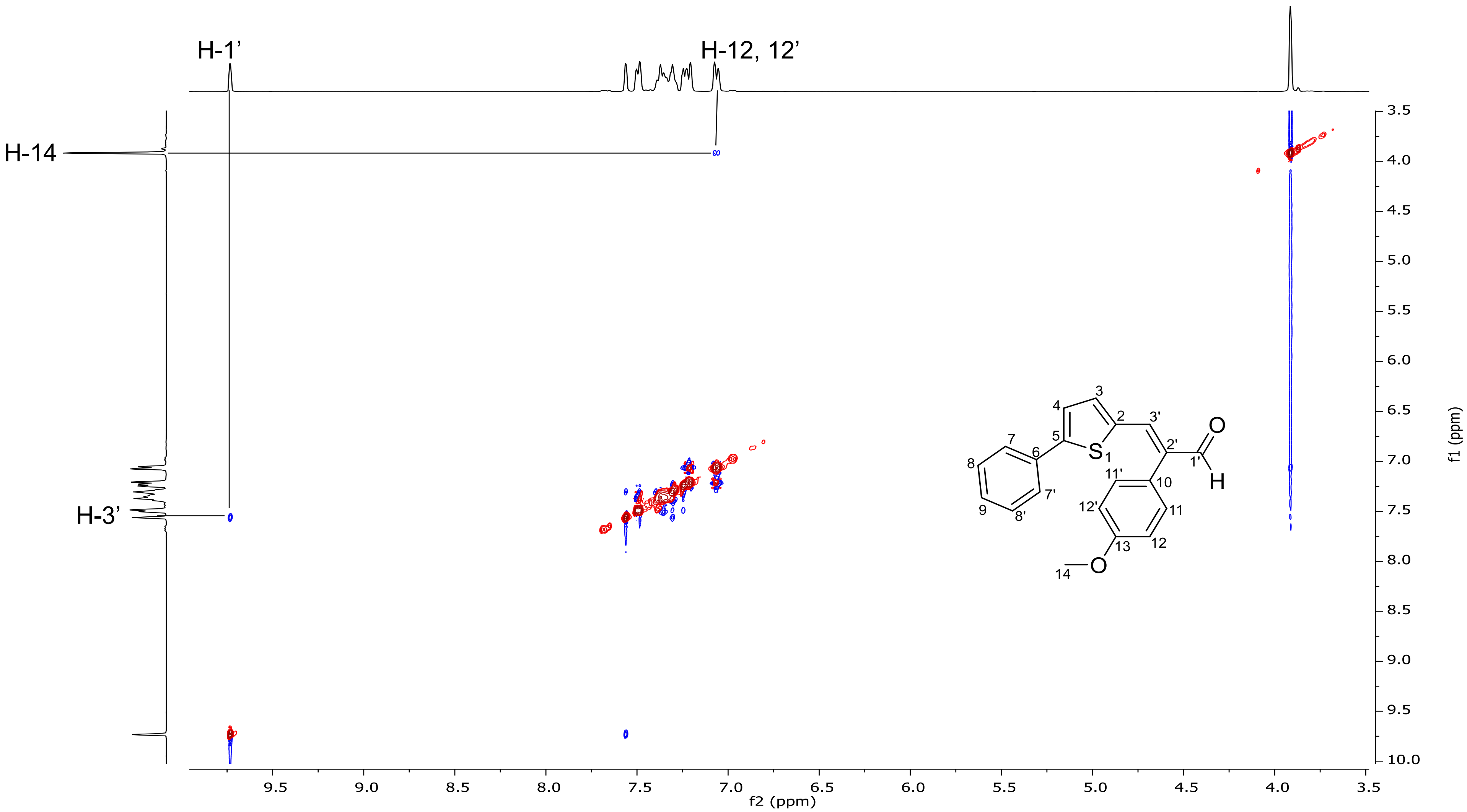

Figure S62: ^{1}H,^{1}H-NOESY (400 MHz, $CDCl_3$) of compound **11**.

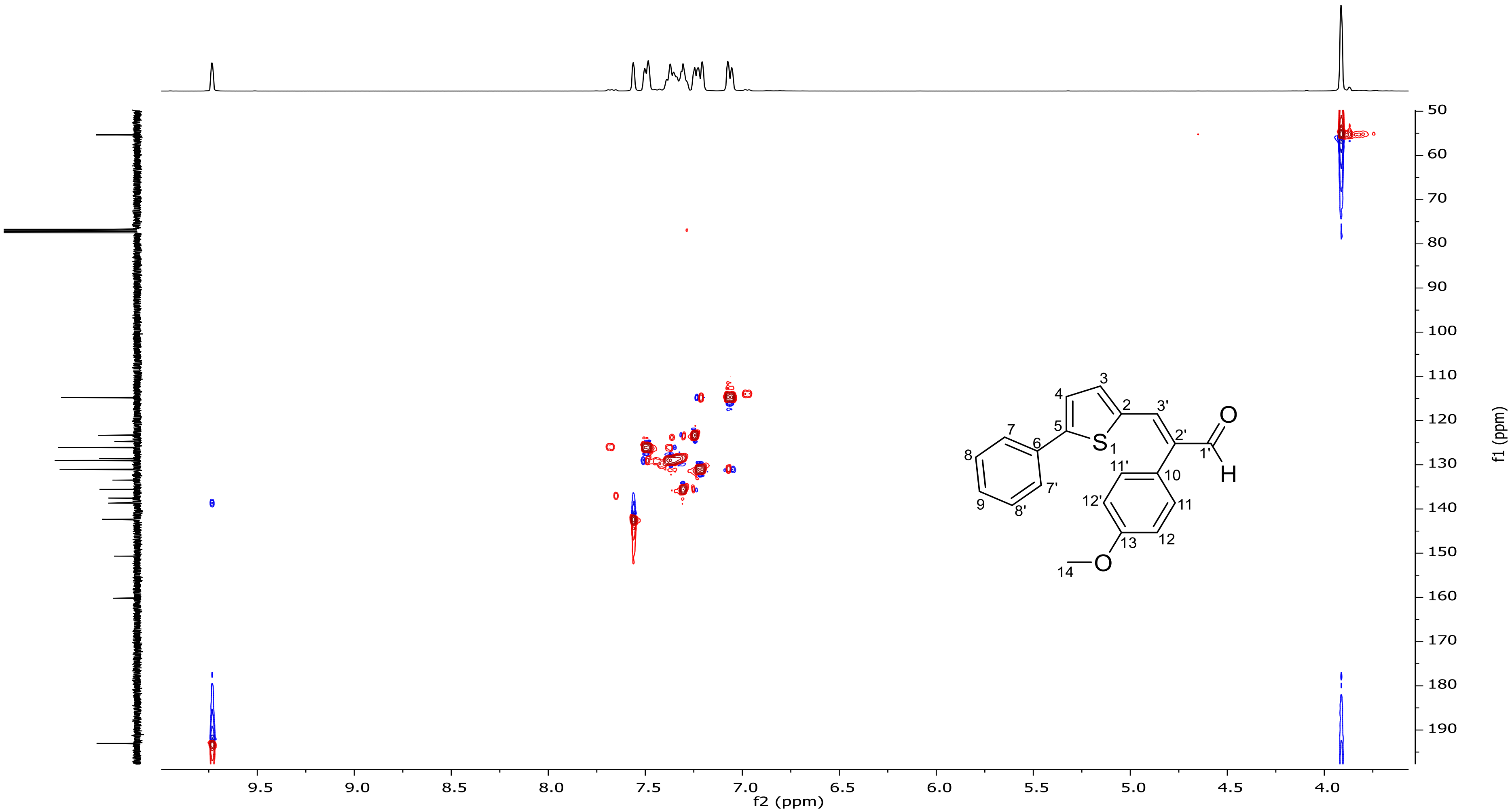

Figure S63: $^{1}H,^{13}C$-HSQC (400, 101 MHz, $CDCl_3$) of compound **11**.

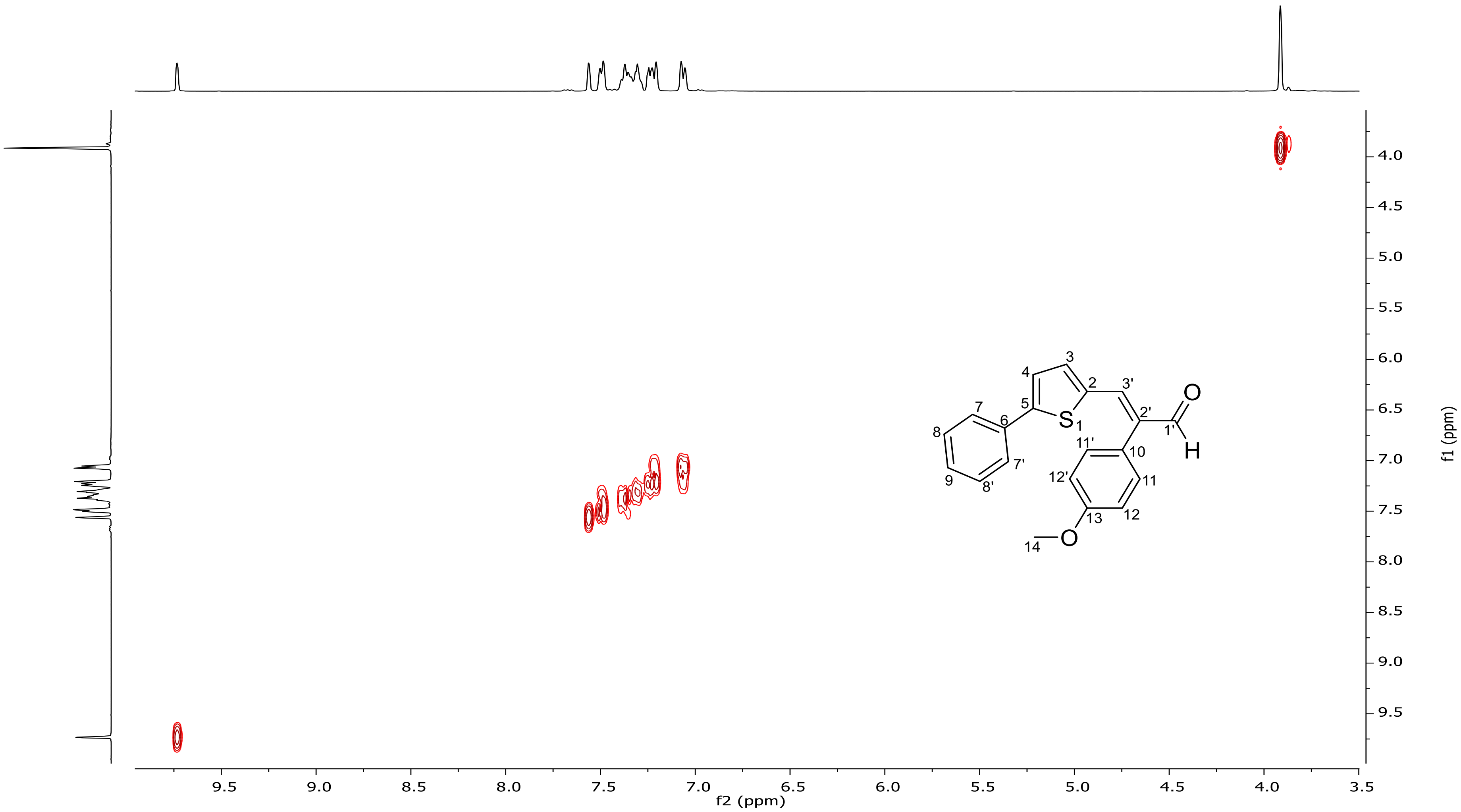

Figure S64: $^{1}H,^{1}H$-COSY (400 MHz, $CDCl_3$) of compound **11**

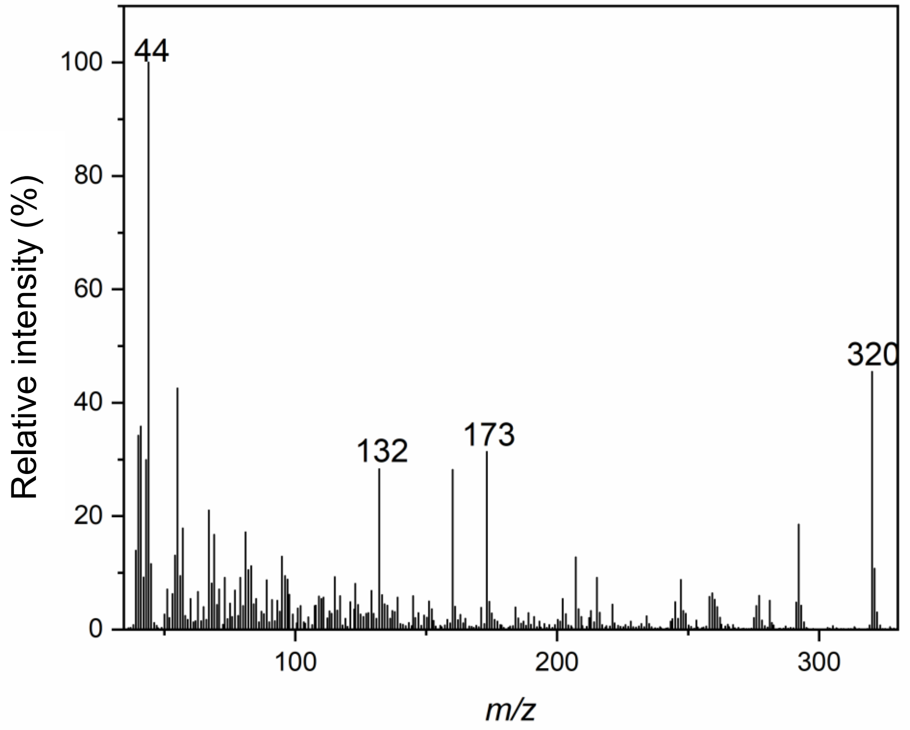

Figure S65: Mass Spectrum(IE, 70 eV) of compound **12**.

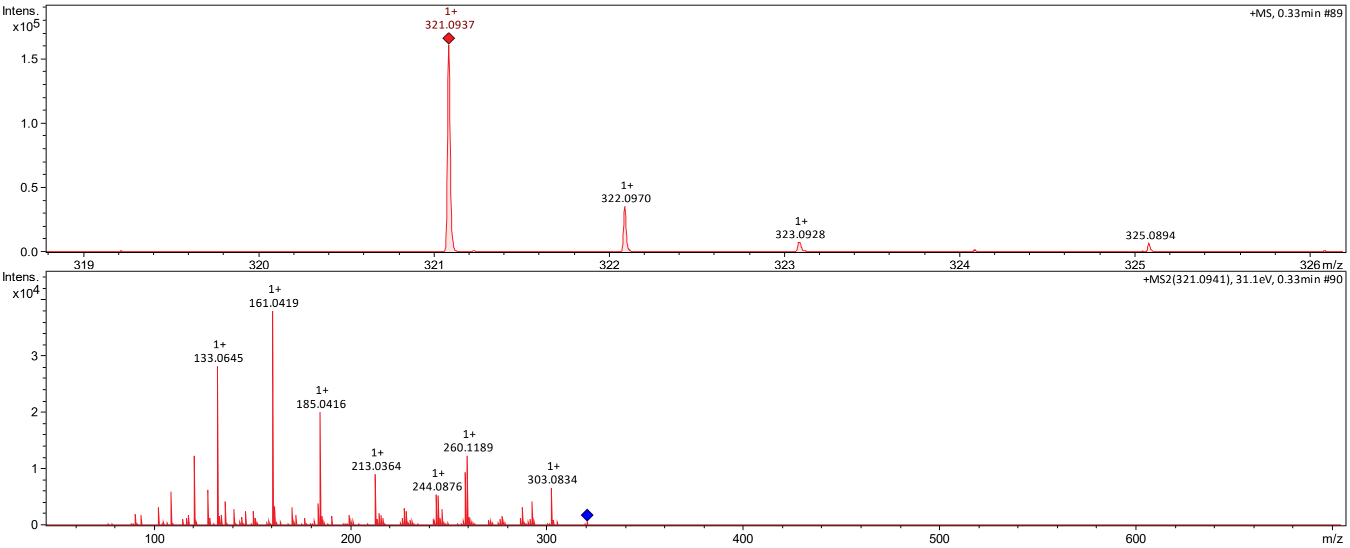

Figure S66: High-resolution mass spectrum (HRMS, ESI) of compound **12.**

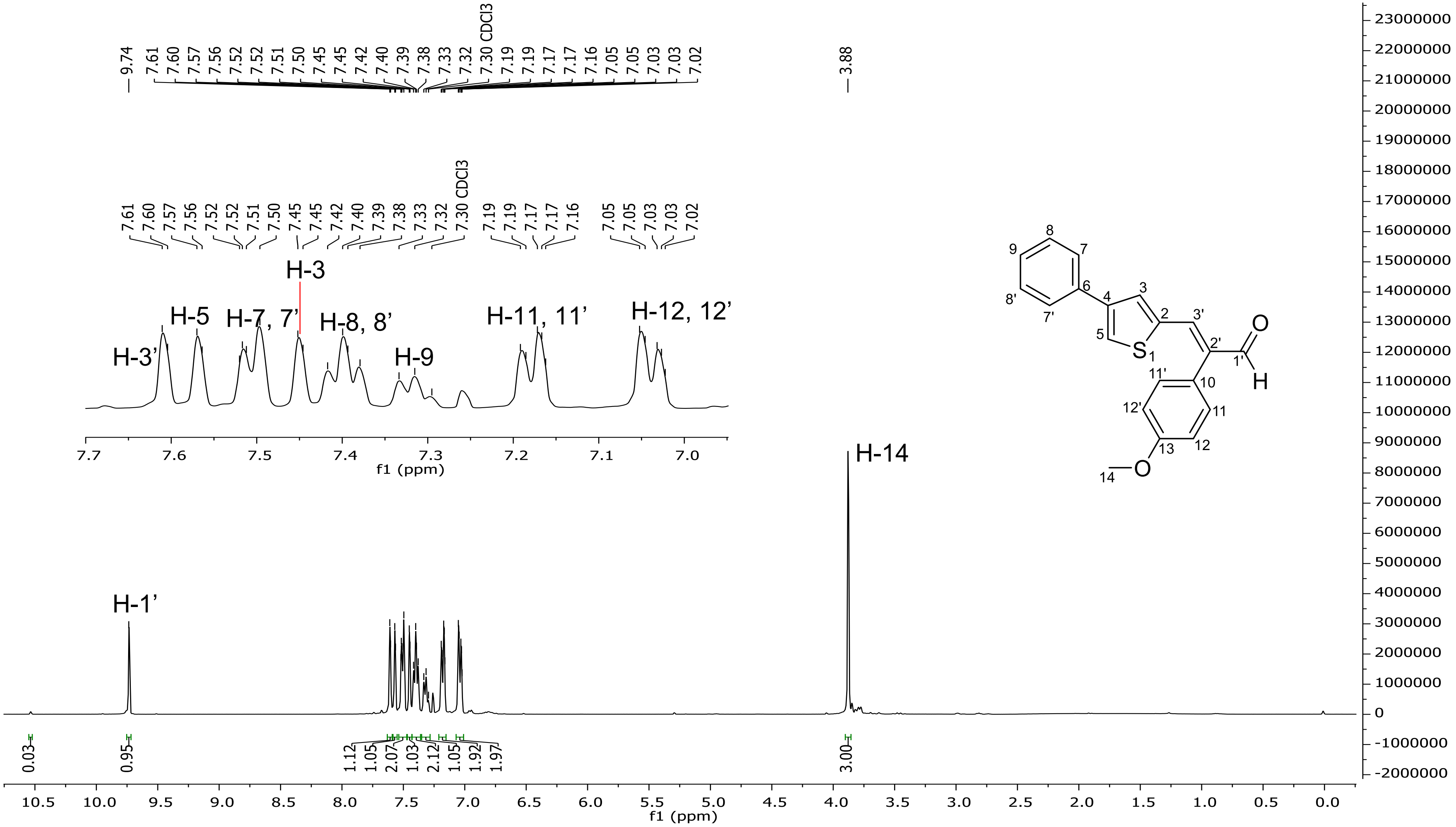

Figure S67: ^{1}H NMR (300 MHz, $CDCl_3$) of compound **12**.

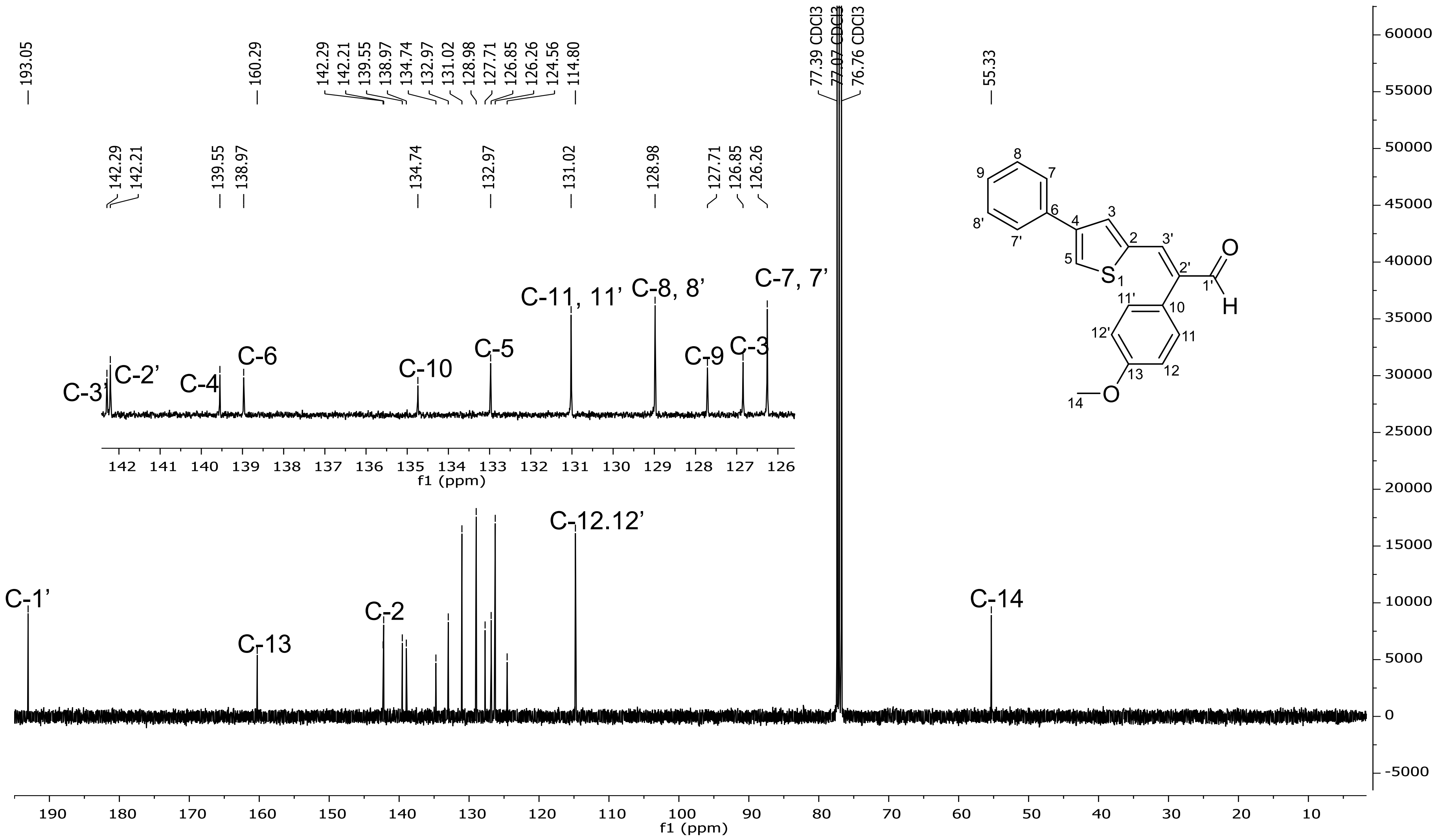

Figure S68: ^{13}C NMR (101 MHz, $CDCl_3$) of compound **12**

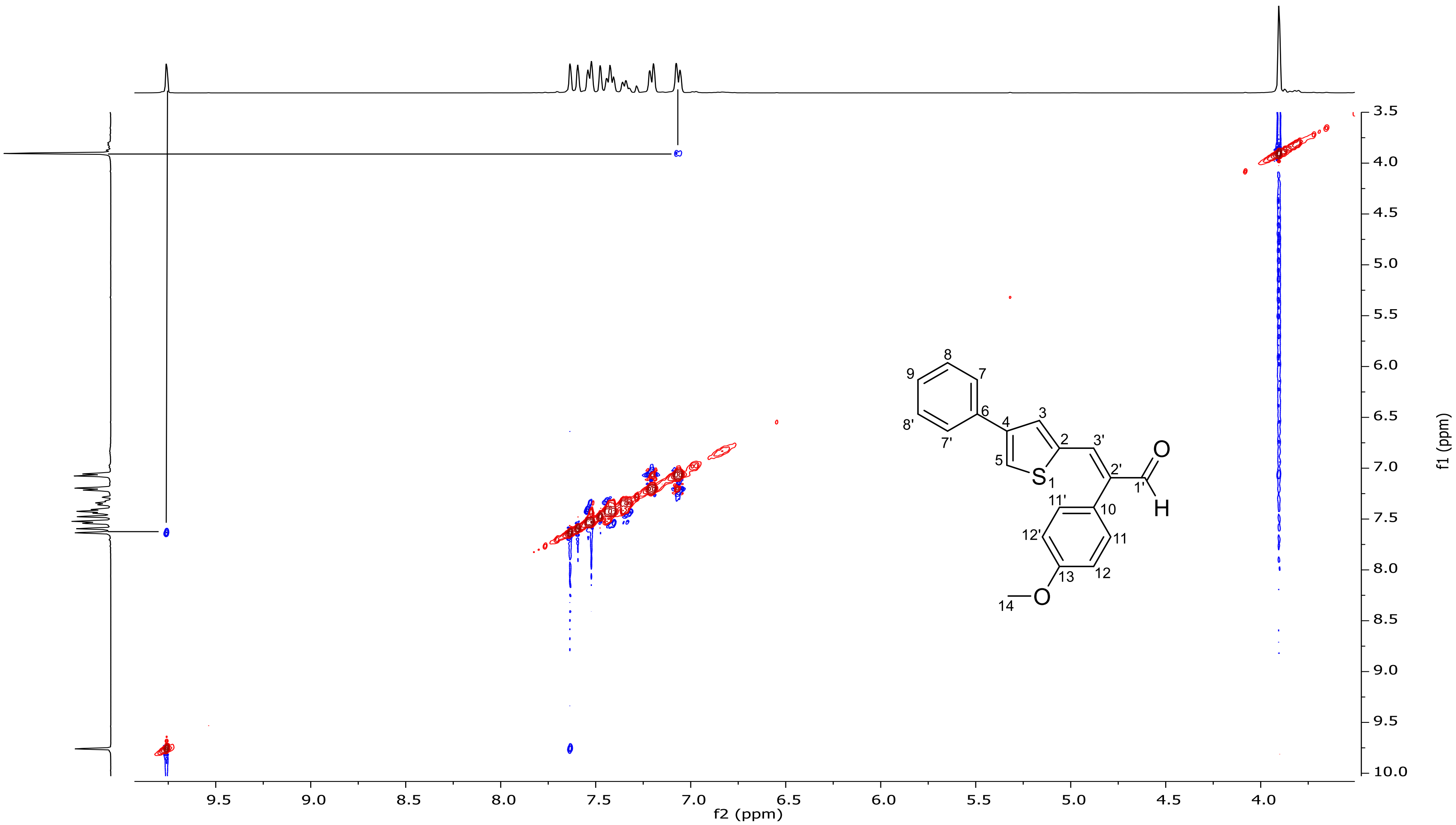

Figure S69: ^{1}H,^{1}H-NOESY (400 MHz, $CDCl_3$) of compound **12**.

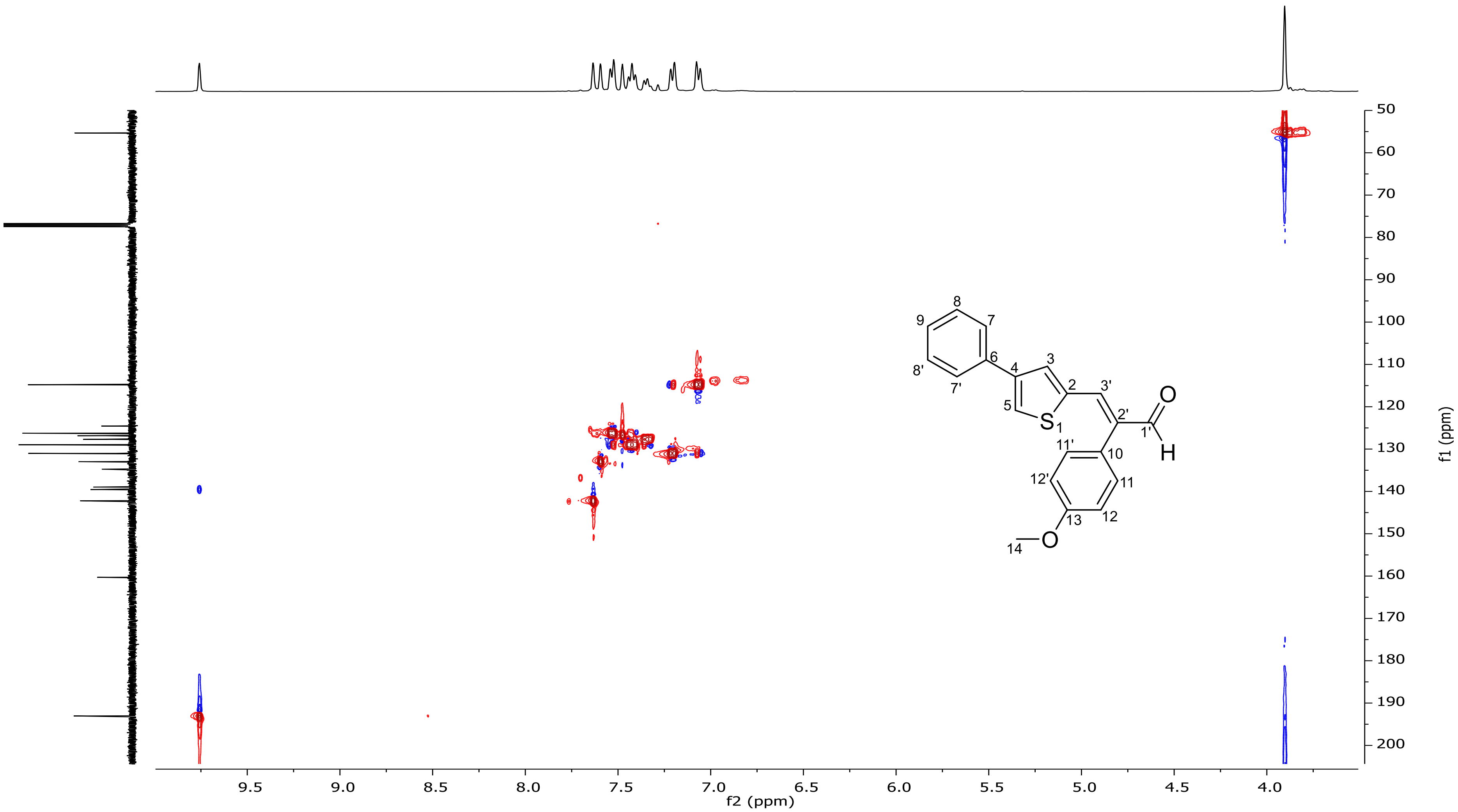

Figure S70: $^{1}H,^{13}C$-HSQC (400, 101 MHz, $CDCl_3$) of compound **12**.

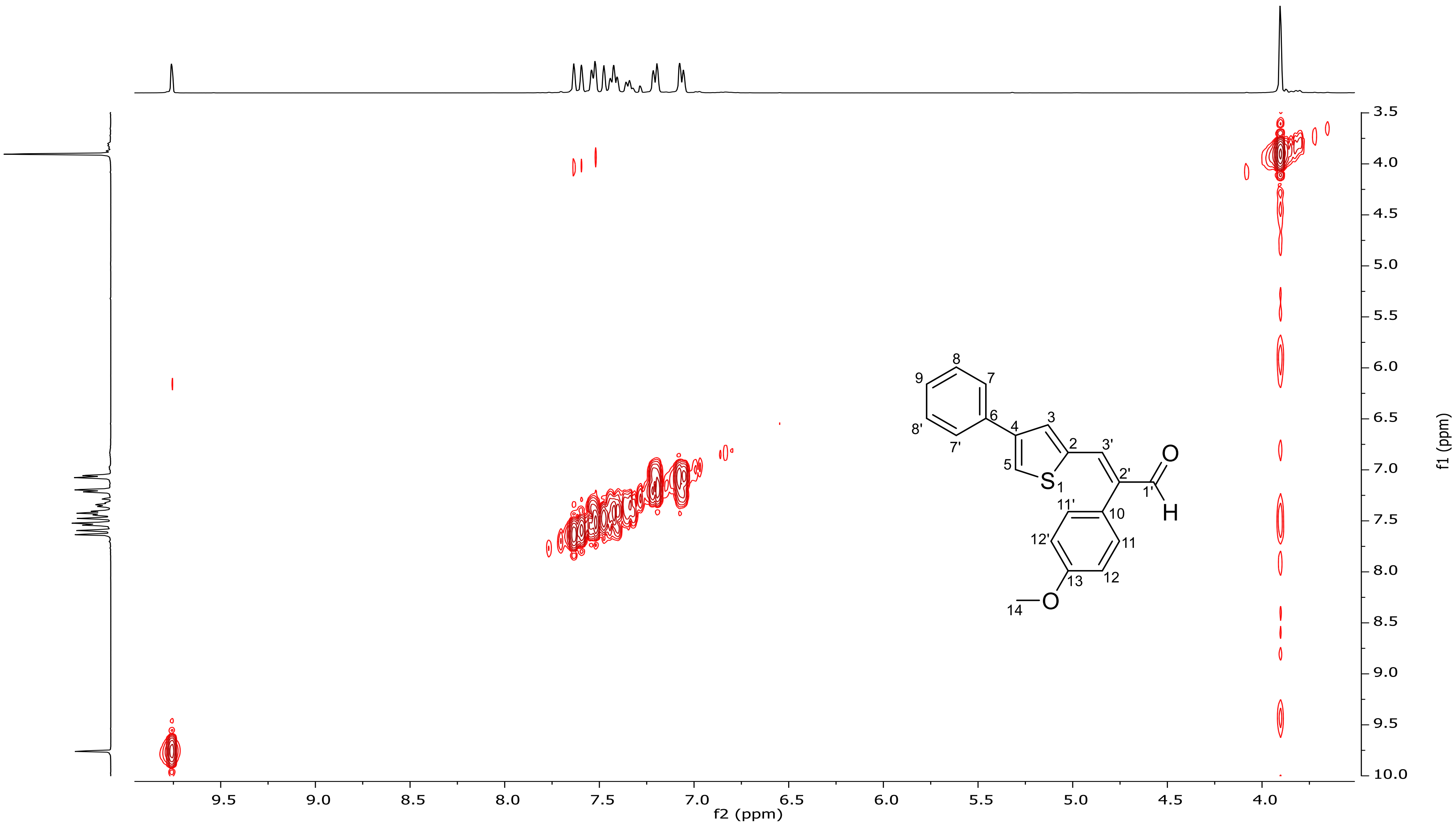

Figure S71: ^{1}H,^{1}H-COSY (400 MHz, $CDCl_3$) of compound **12**

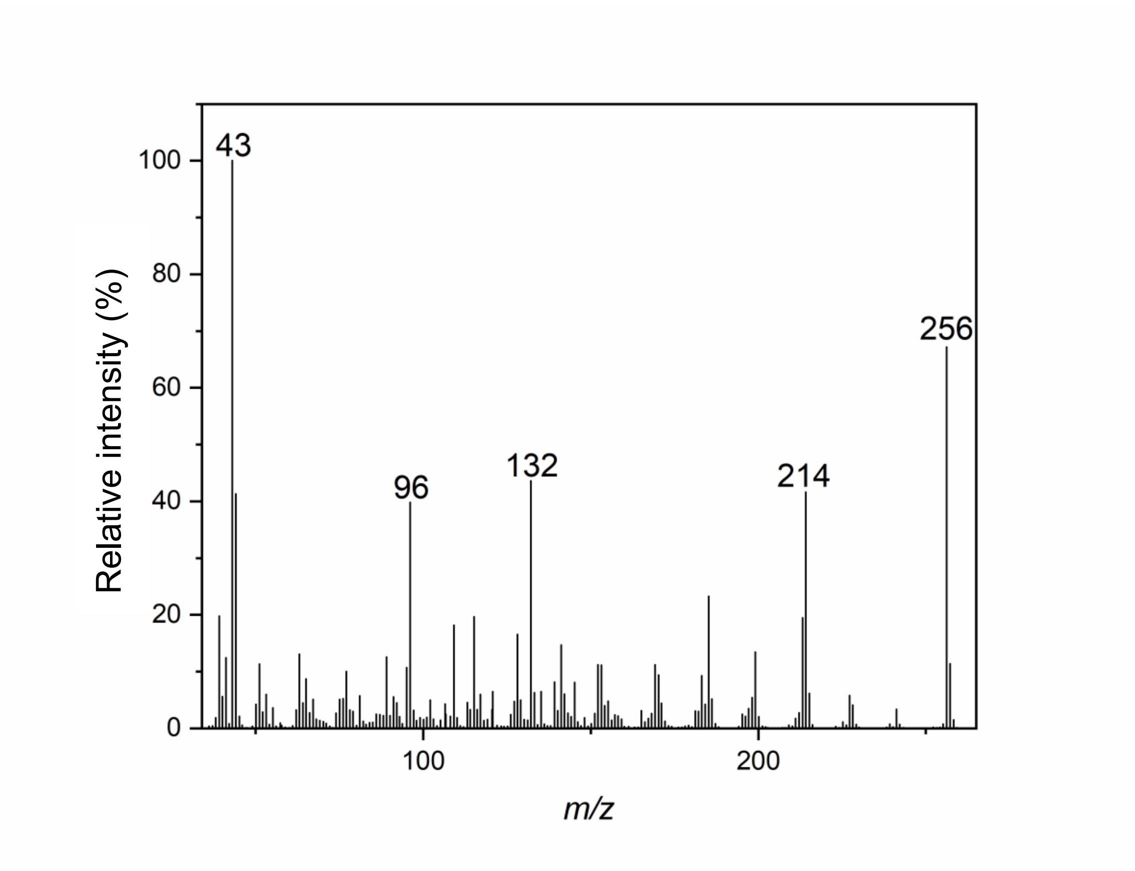

Figure S72: Mass Spectrum(IE, 70 eV) of compound **13**.

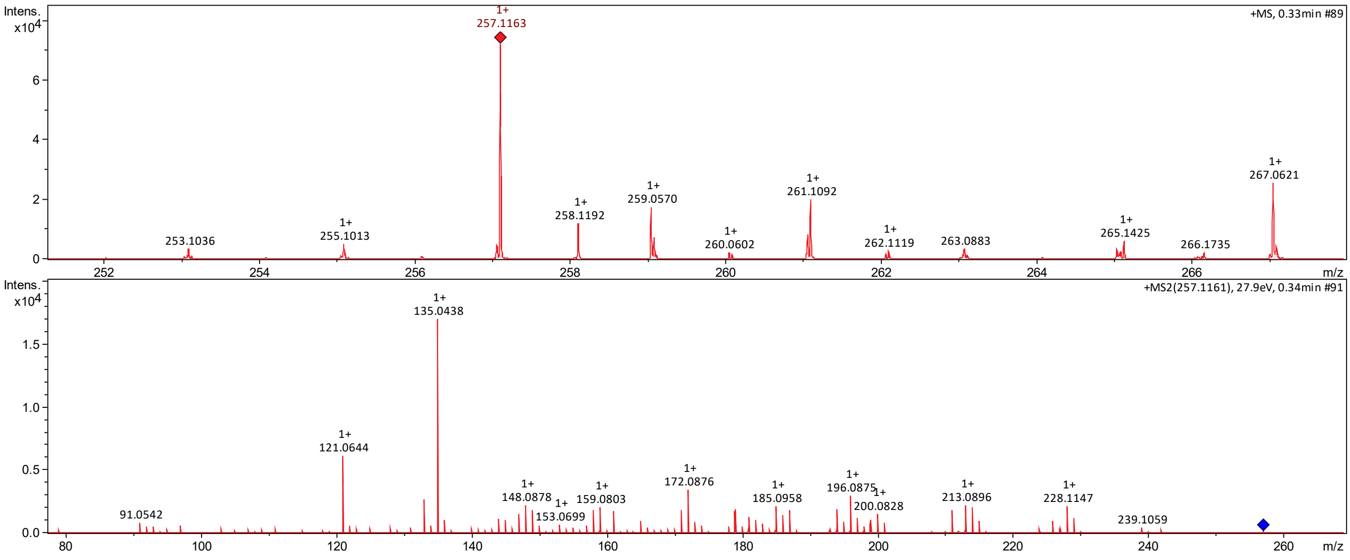

Figure S73: High-resolution mass spectrum (HRMS, ESI) of compound **13.**

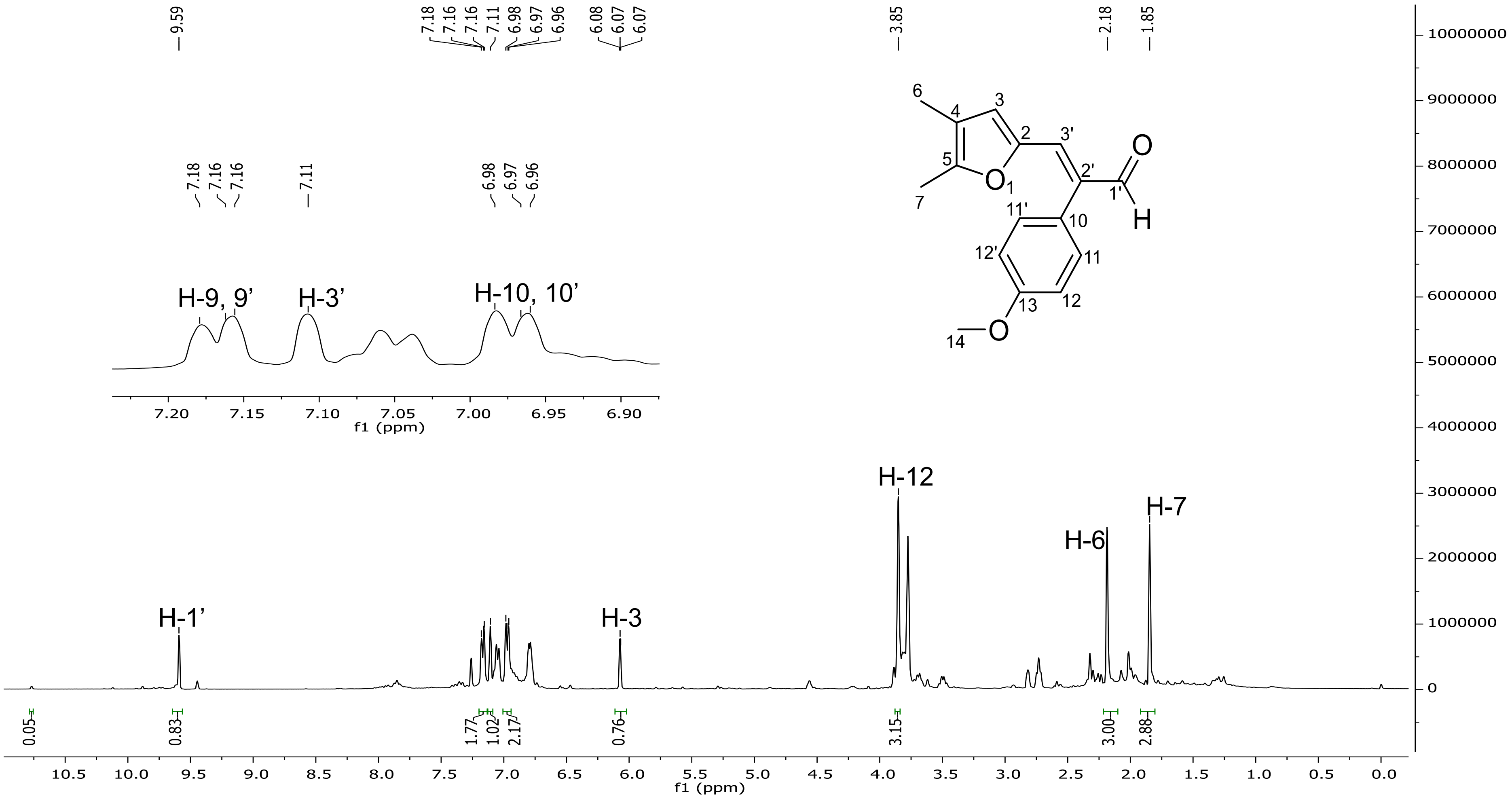

Figure S74: ^{1}H NMR (300 MHz, $CDCl_3$) of compound **13**.

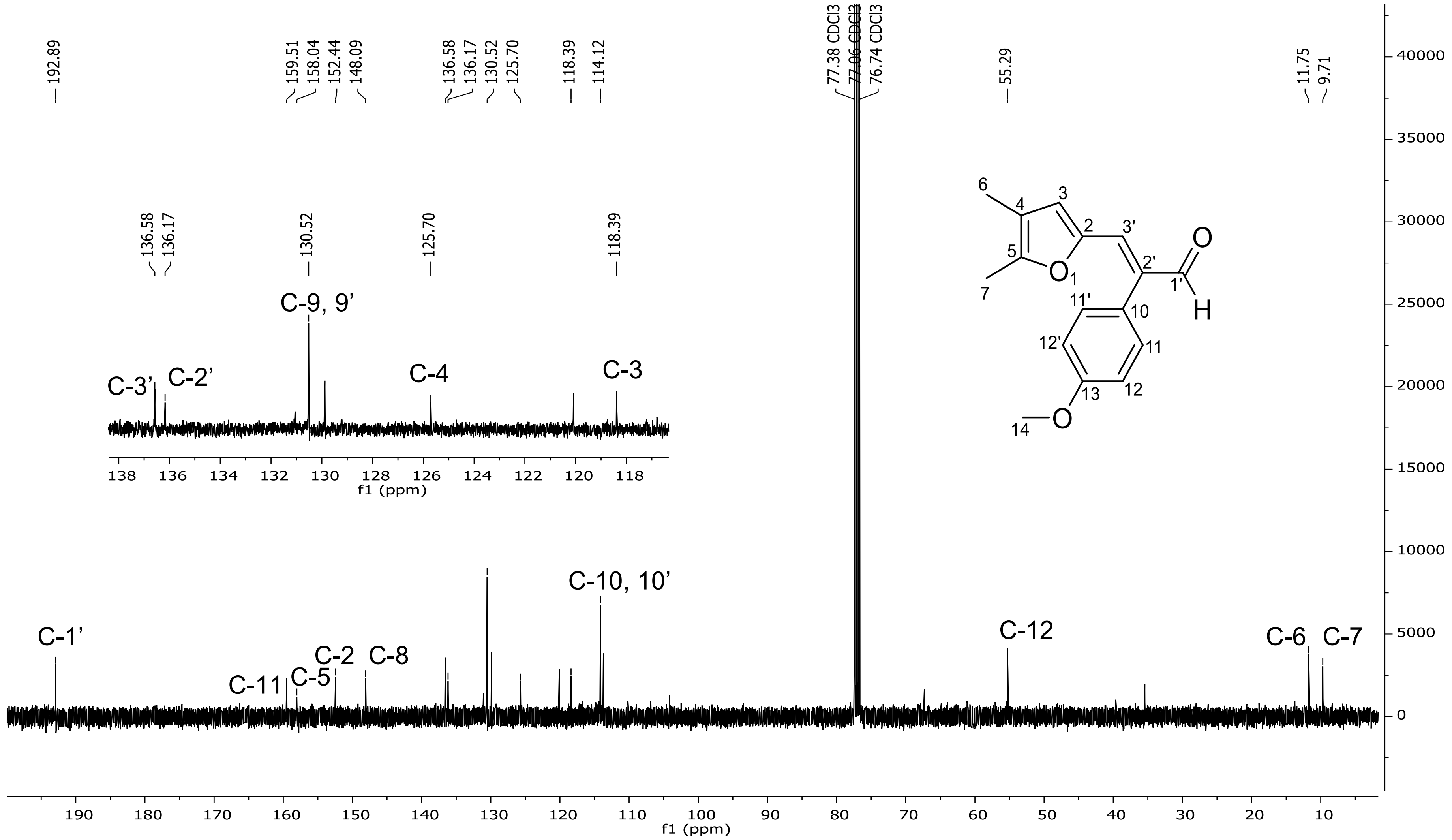

Figure S75: ^{13}C NMR (101 MHz, $CDCl_3$) of compound **13**.

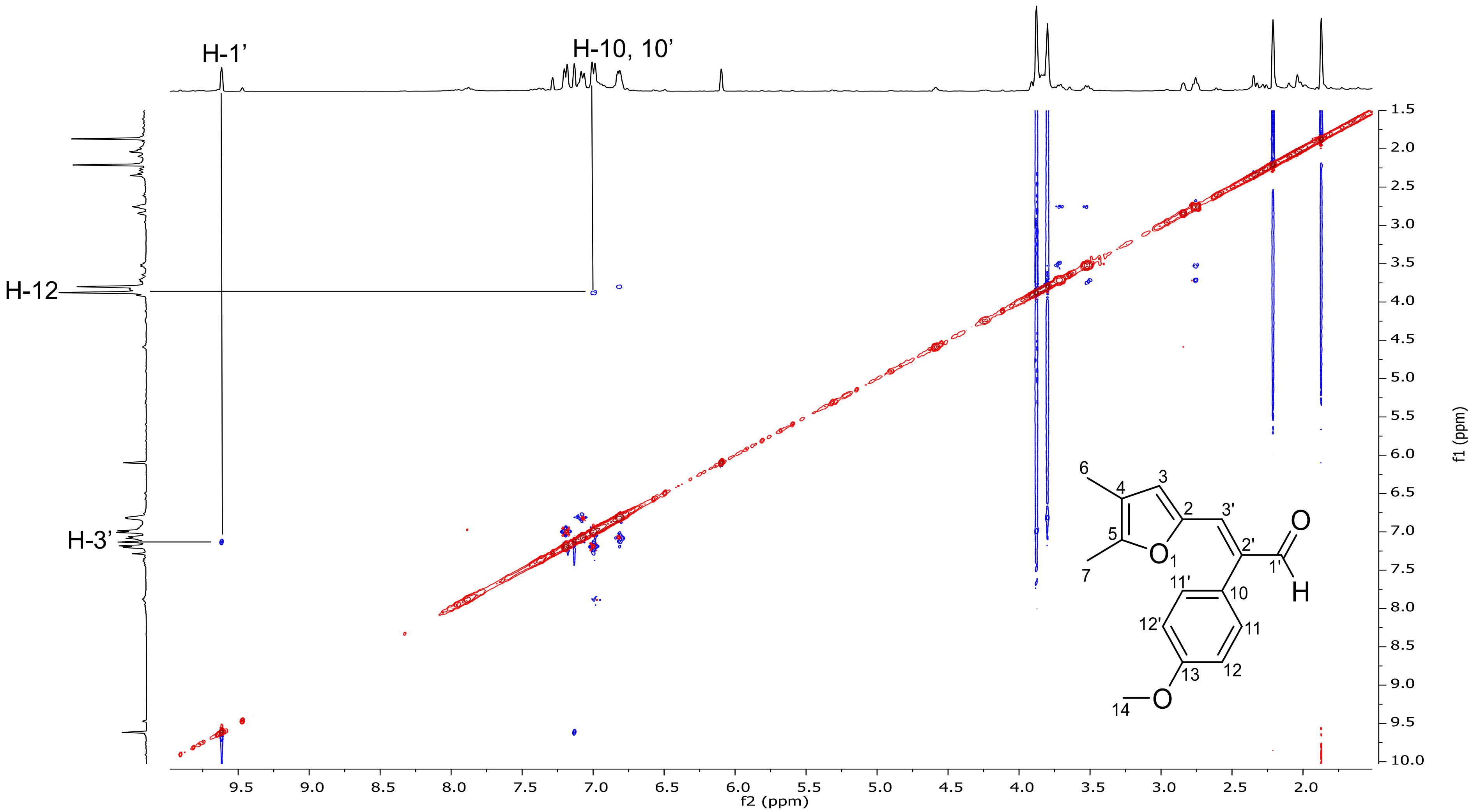

Figure S76: $^{1}H,^{1}H$-NOESY (400 MHz, $CDCl_3$) of compound **13**.

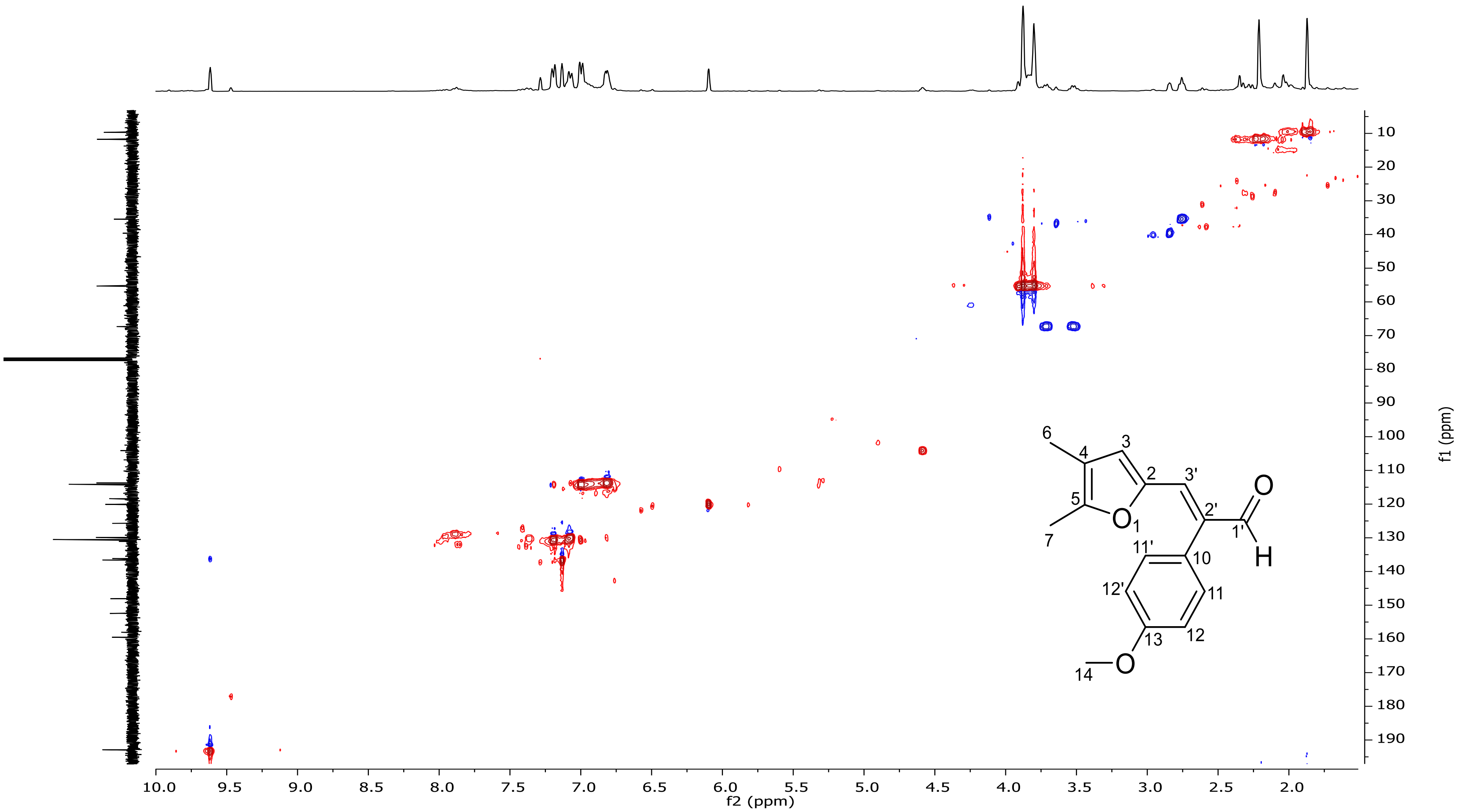

Figure S77: $^{1}H,^{13}C$-HSQC (400, 101 MHz, $CDCl_3$) of compound **13**.

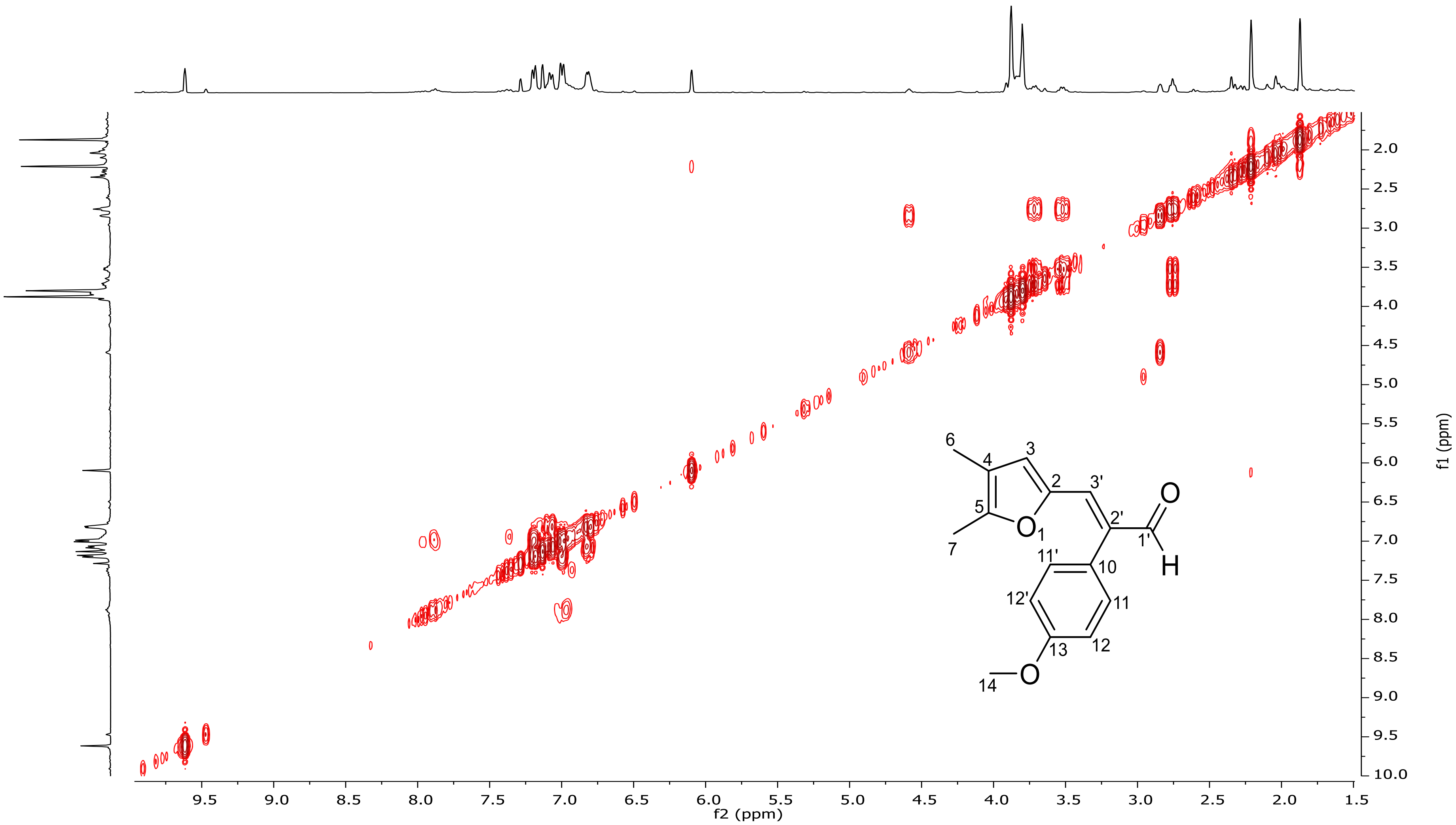

Figure S78: ${}^{1}H,{}^{1}H$-COSY (400 MHz, $CDCl_3$) of compound **13**.

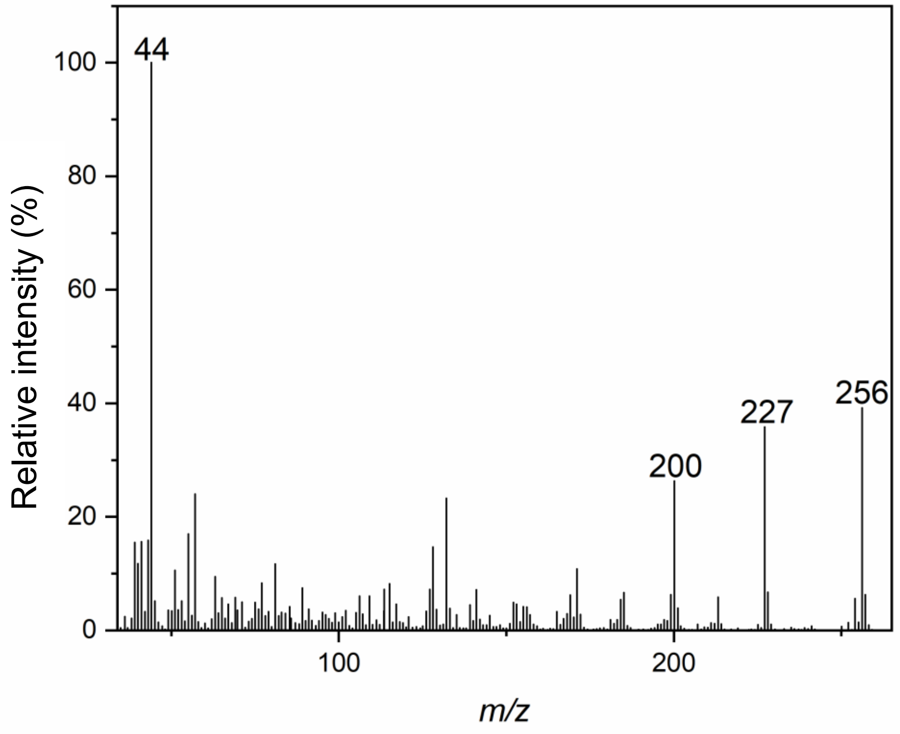

Figure S79: Mass Spectrum(IE, 70 eV) of compound **14**.

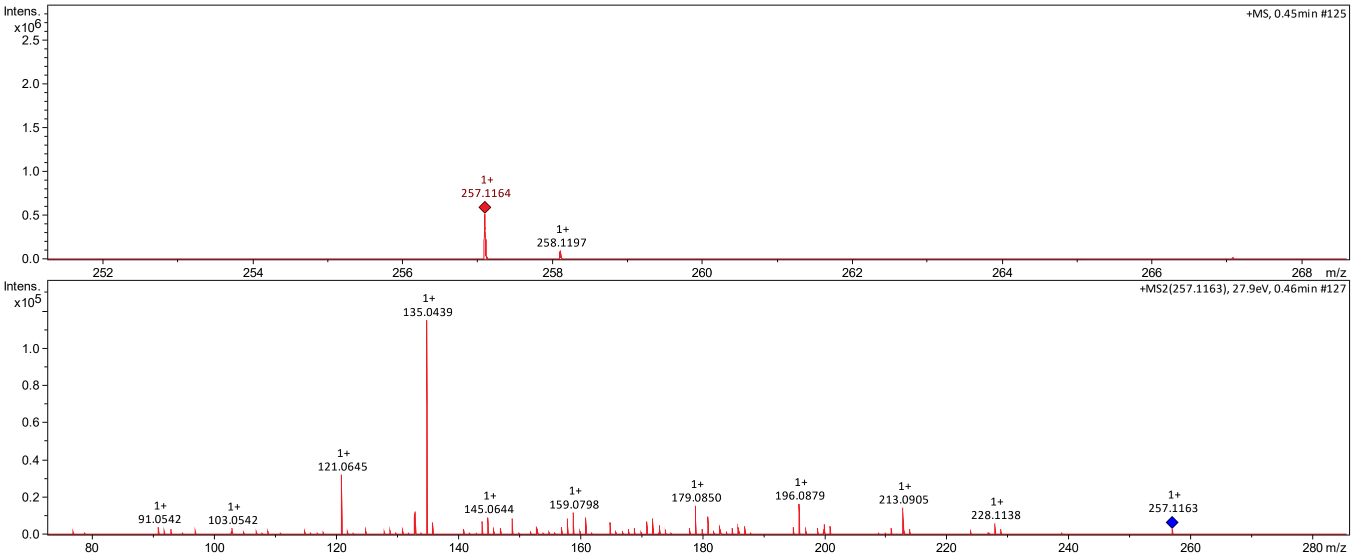

Figure S80: High-resolution mass spectrum (HRMS, ESI) of compound **14.**

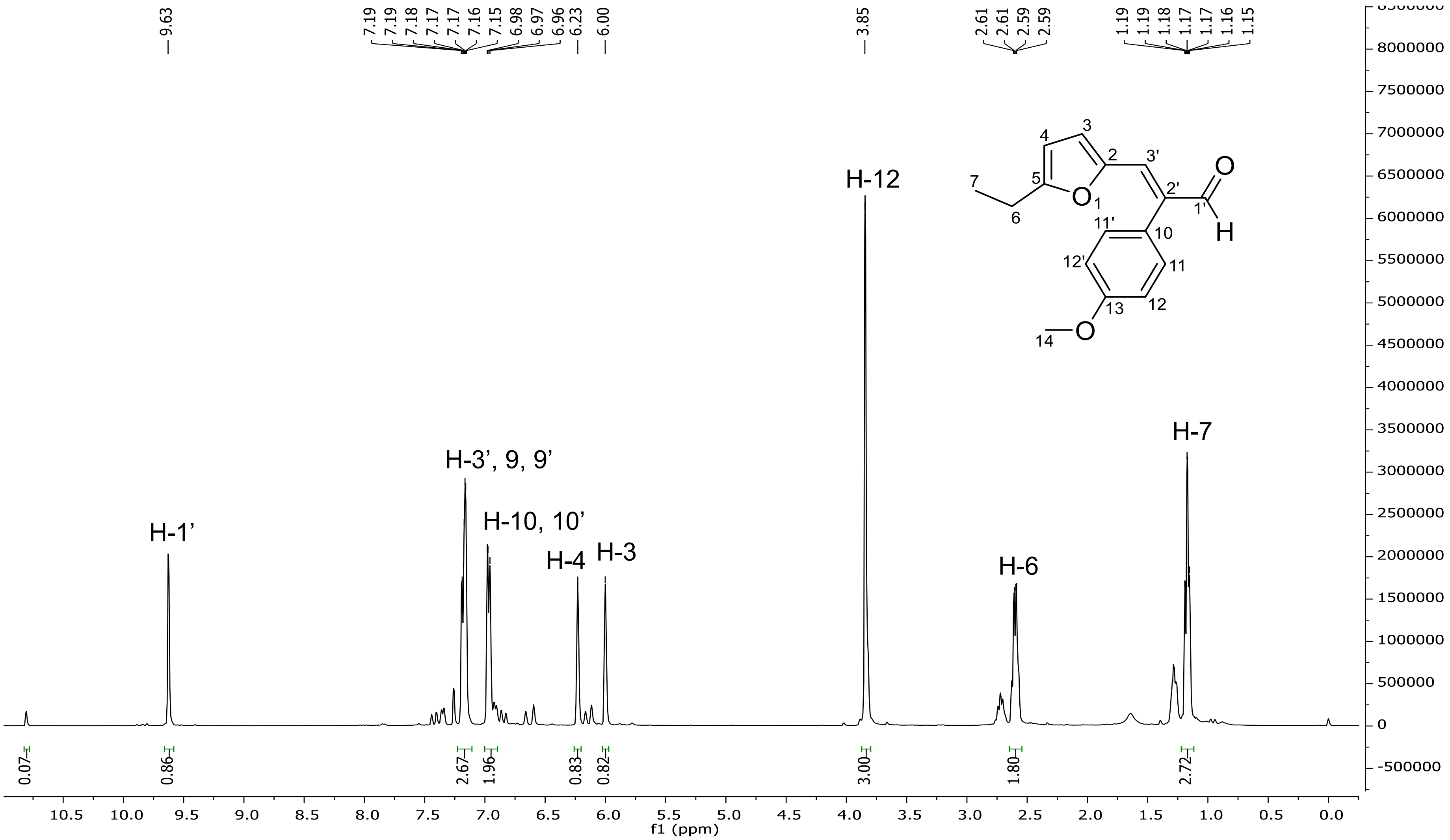

Figure S81: ^{1}H NMR (300 MHz, $CDCl_3$) of compound **14**.

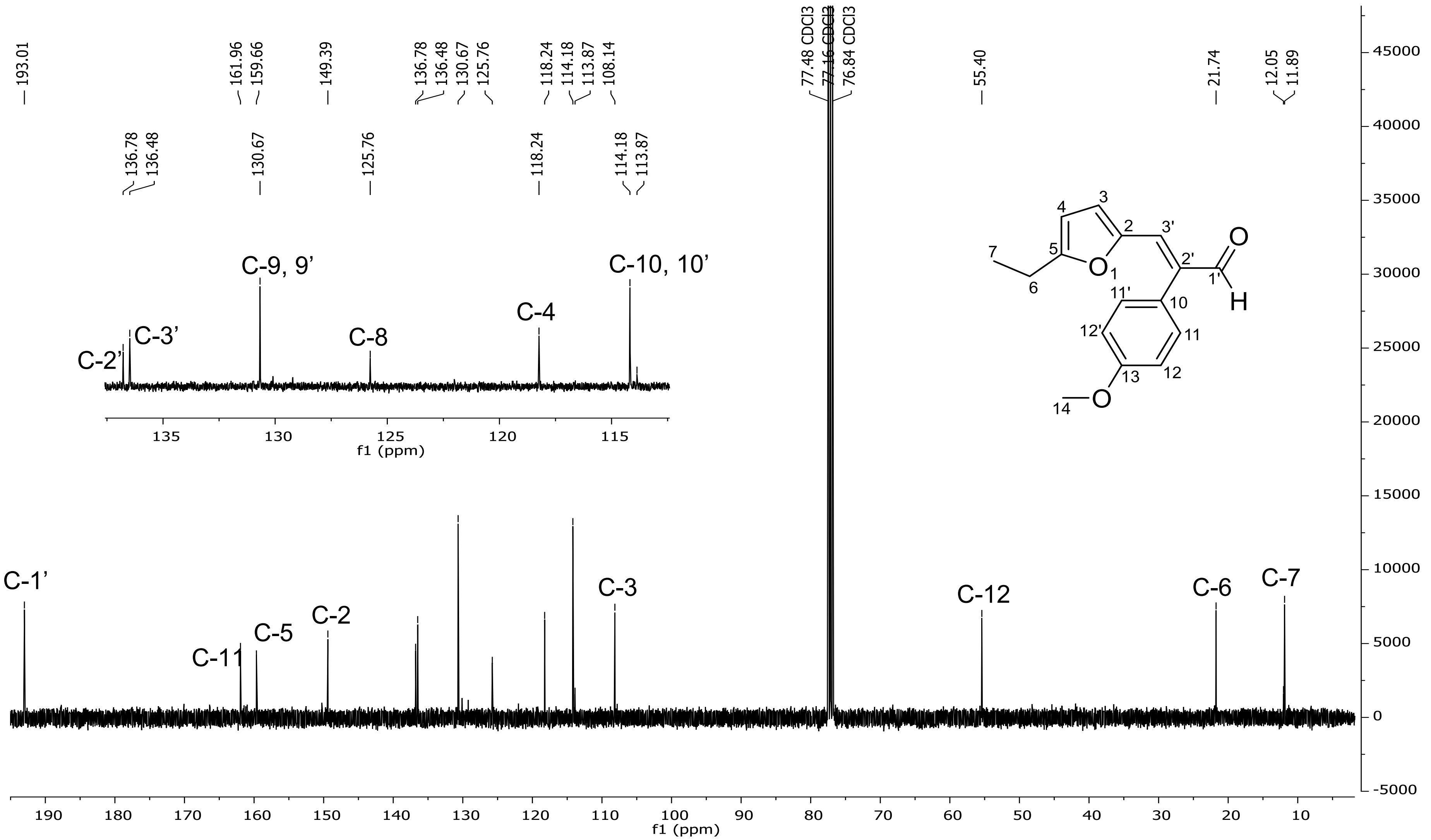

Figure S82: ^{13}C NMR (101 MHz, $CDCl_3$) of compound **14**.

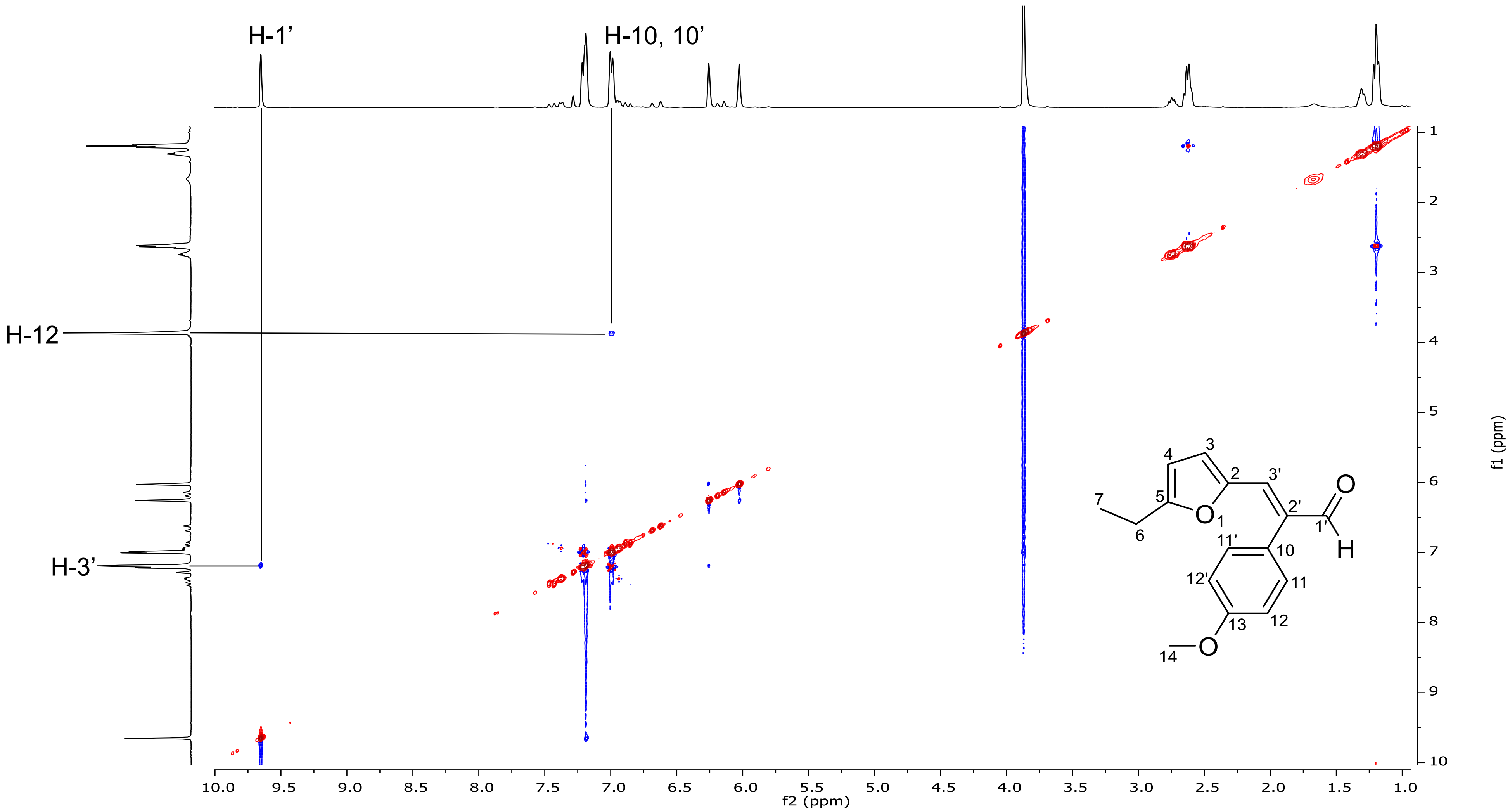

Figure S83: $^{1}H,^{1}H$-NOESY (400 MHz, $CDCl_3$) of compound **14**.

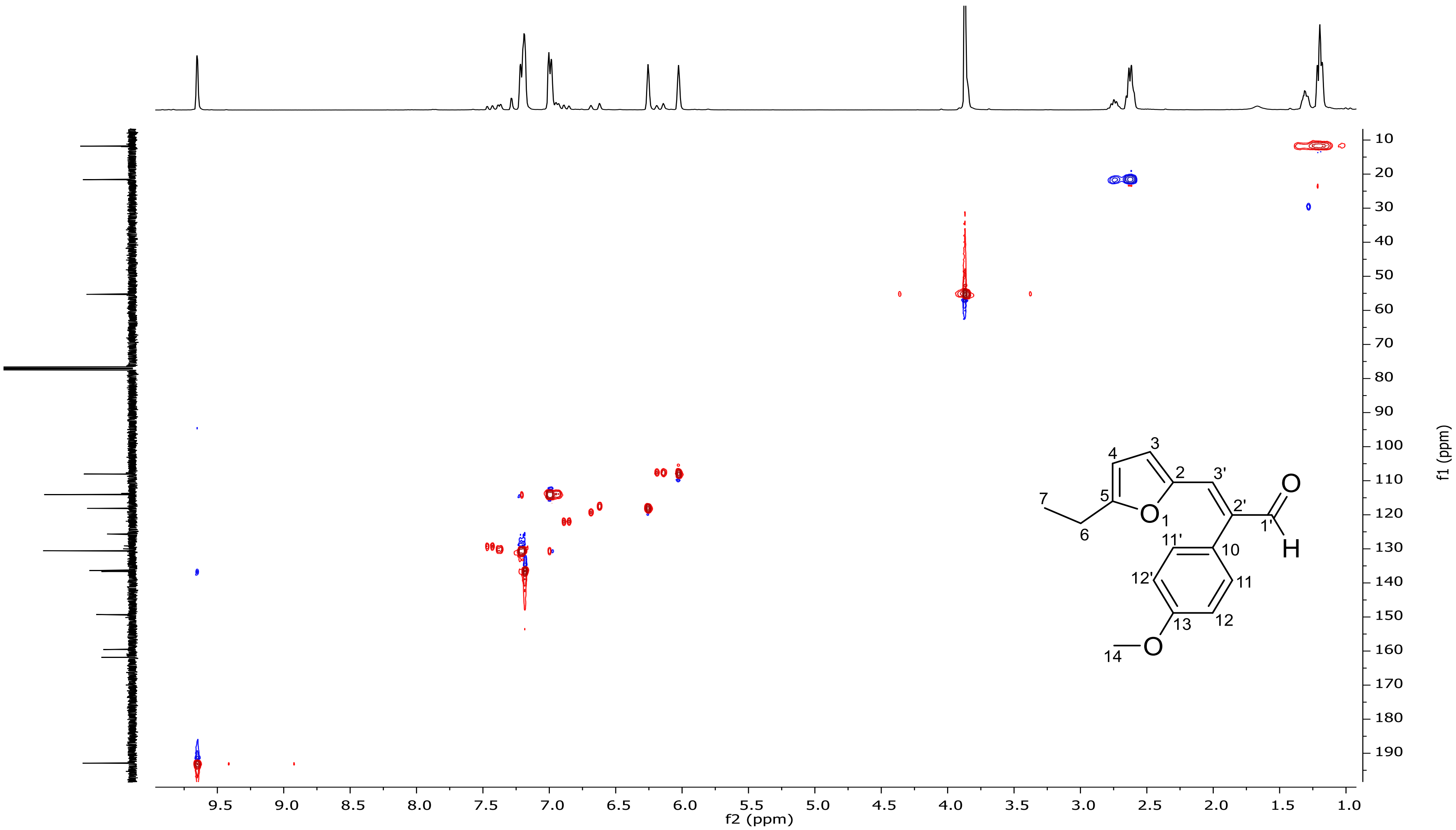

Figure S84: $^{1}H,^{13}C$-HSQC (400, 101 MHz, $CDCl_3$) of compound **14**.

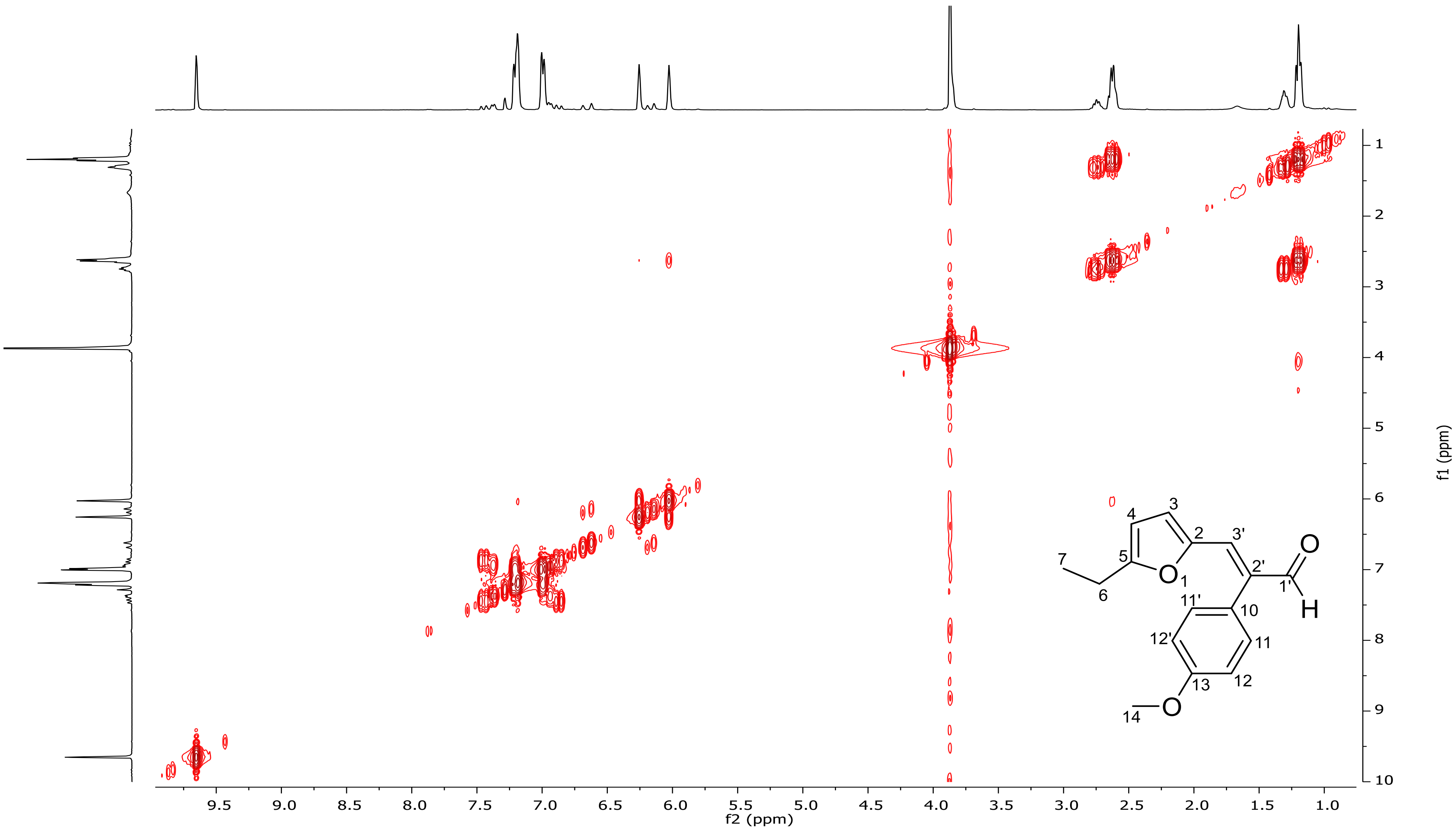

Figure S85: $^1H,^1H$-COSY (400 MHz, $CDCl_3$) of compound **14.**

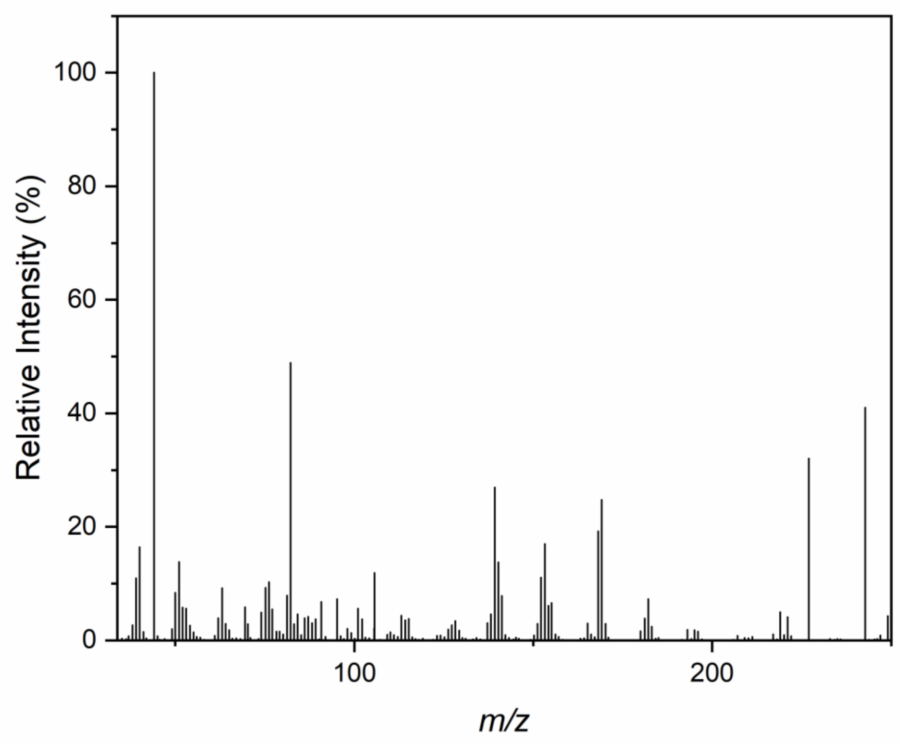

Figure S86: Mass Spectrum(IE, 70 eV) of compound **15.**

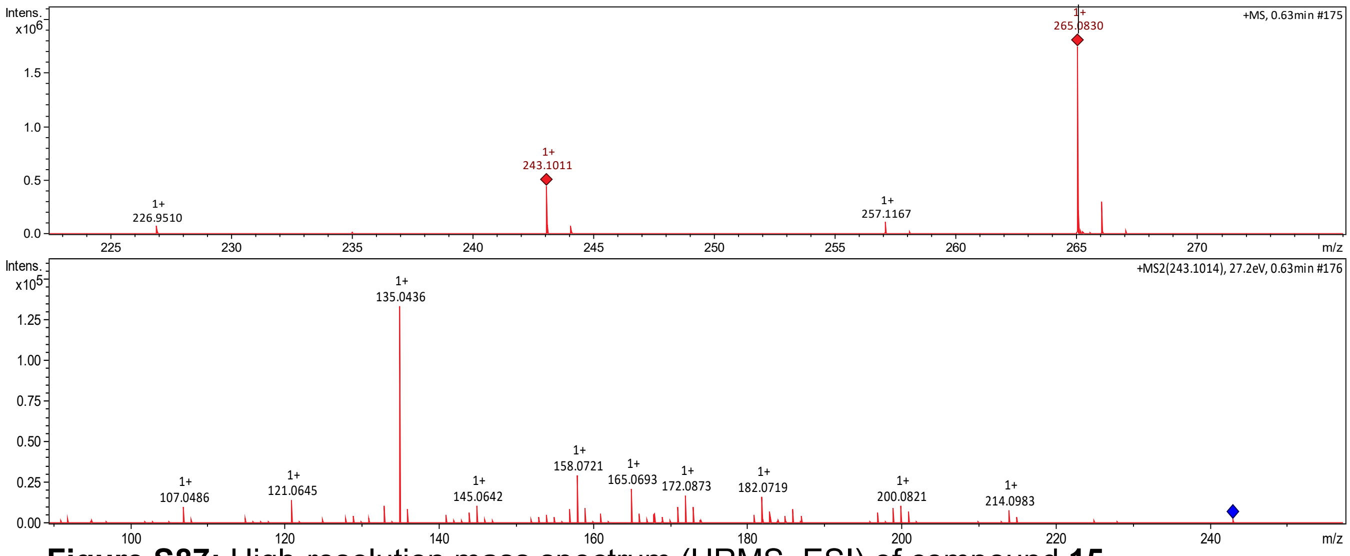

Figure S87: High-resolution mass spectrum (HRMS, ESI) of compound **15.**

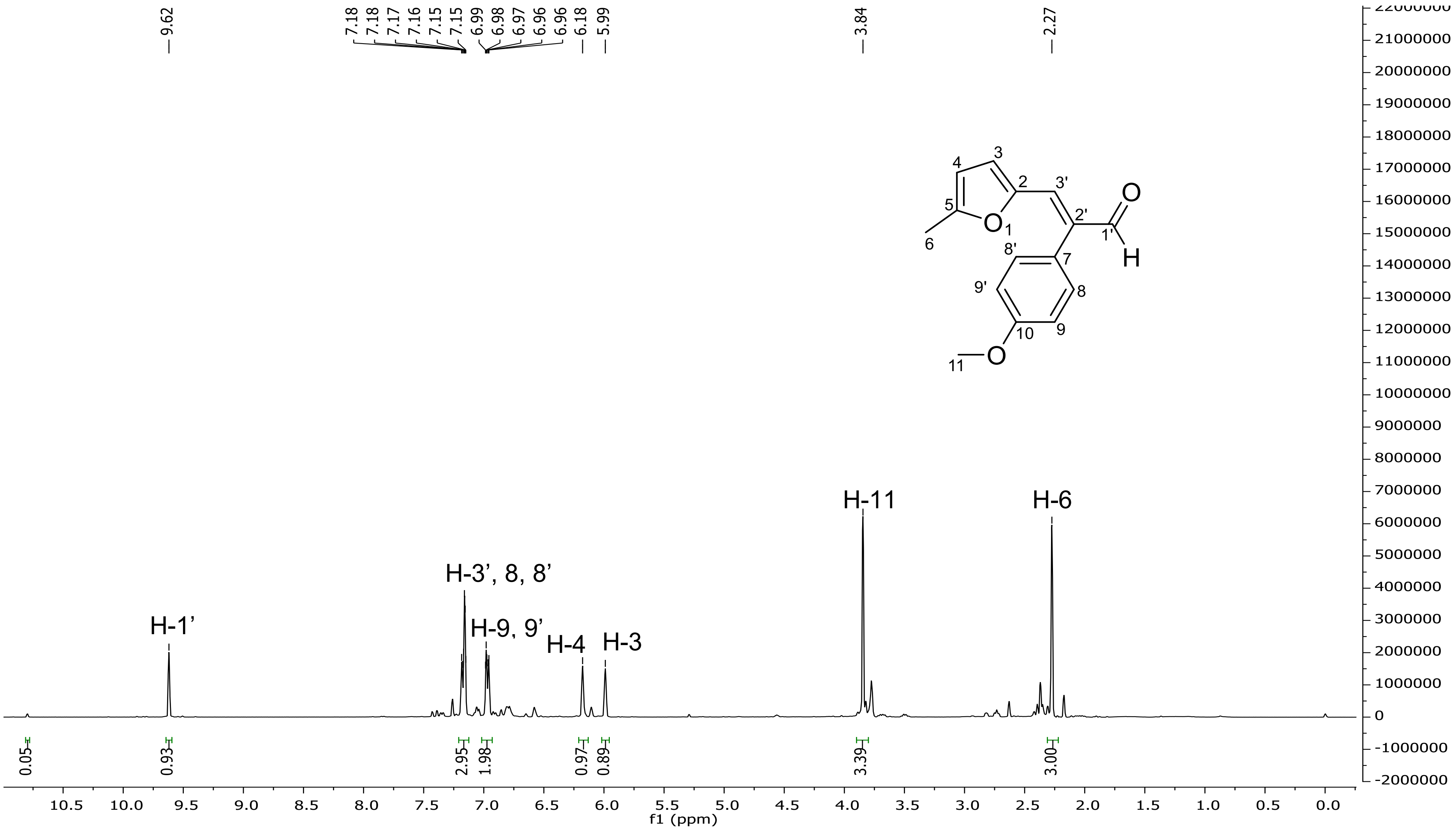

Figure S88: ^{1}H NMR (300 MHz, $CDCl_3$) of compound **15**.

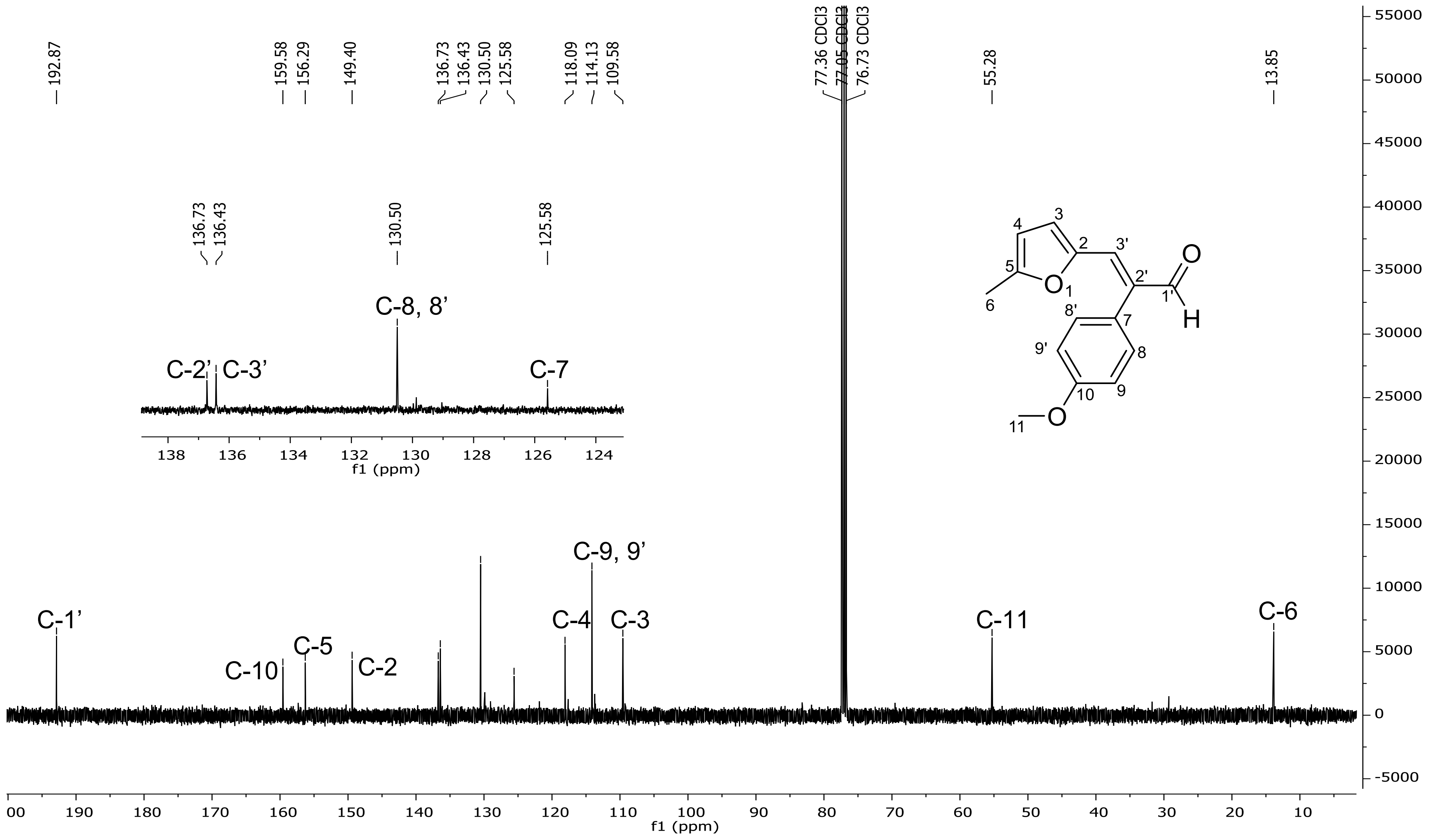

Figure S89: ^{13}C NMR (101 MHz, $CDCl_3$) of compound **15**.

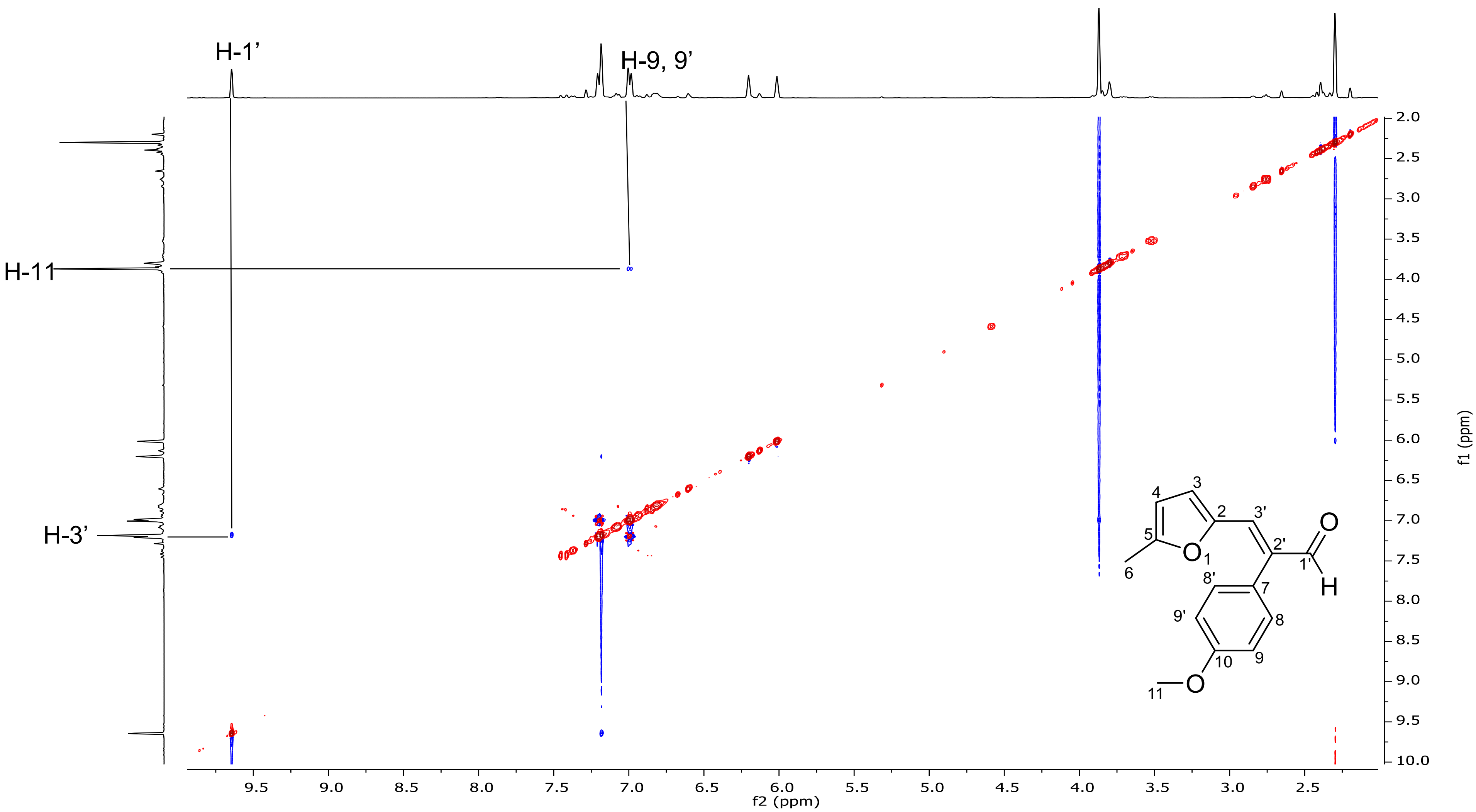

Figure S90: $^{1}H,^{1}H$-NOESY (400 MHz, $CDCl_3$) of compound **15**.

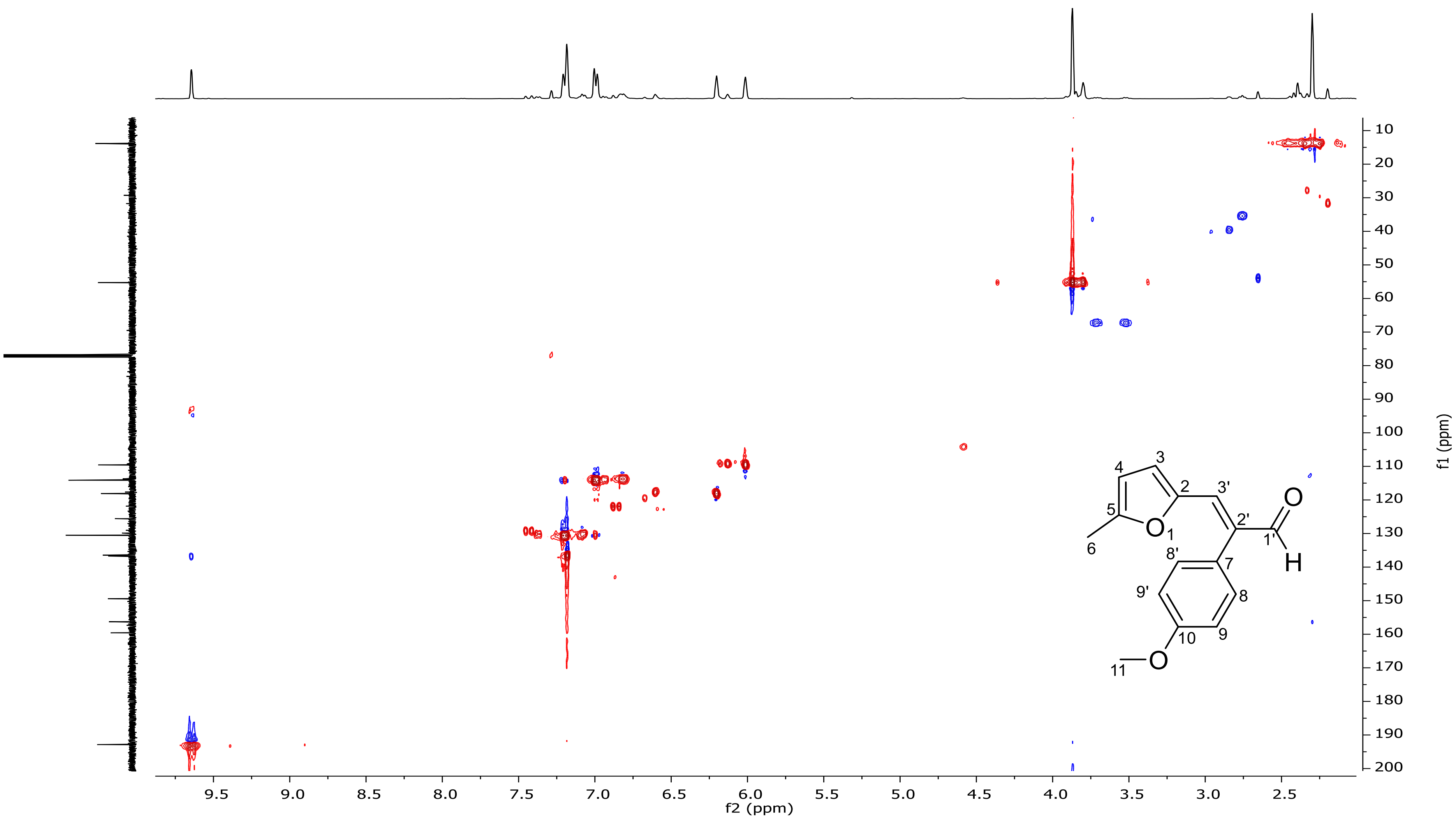

Figure S91: ^{1}H,^{13}C-HSQC (400, 101 MHz, $CDCl_3$) of compound **15**.

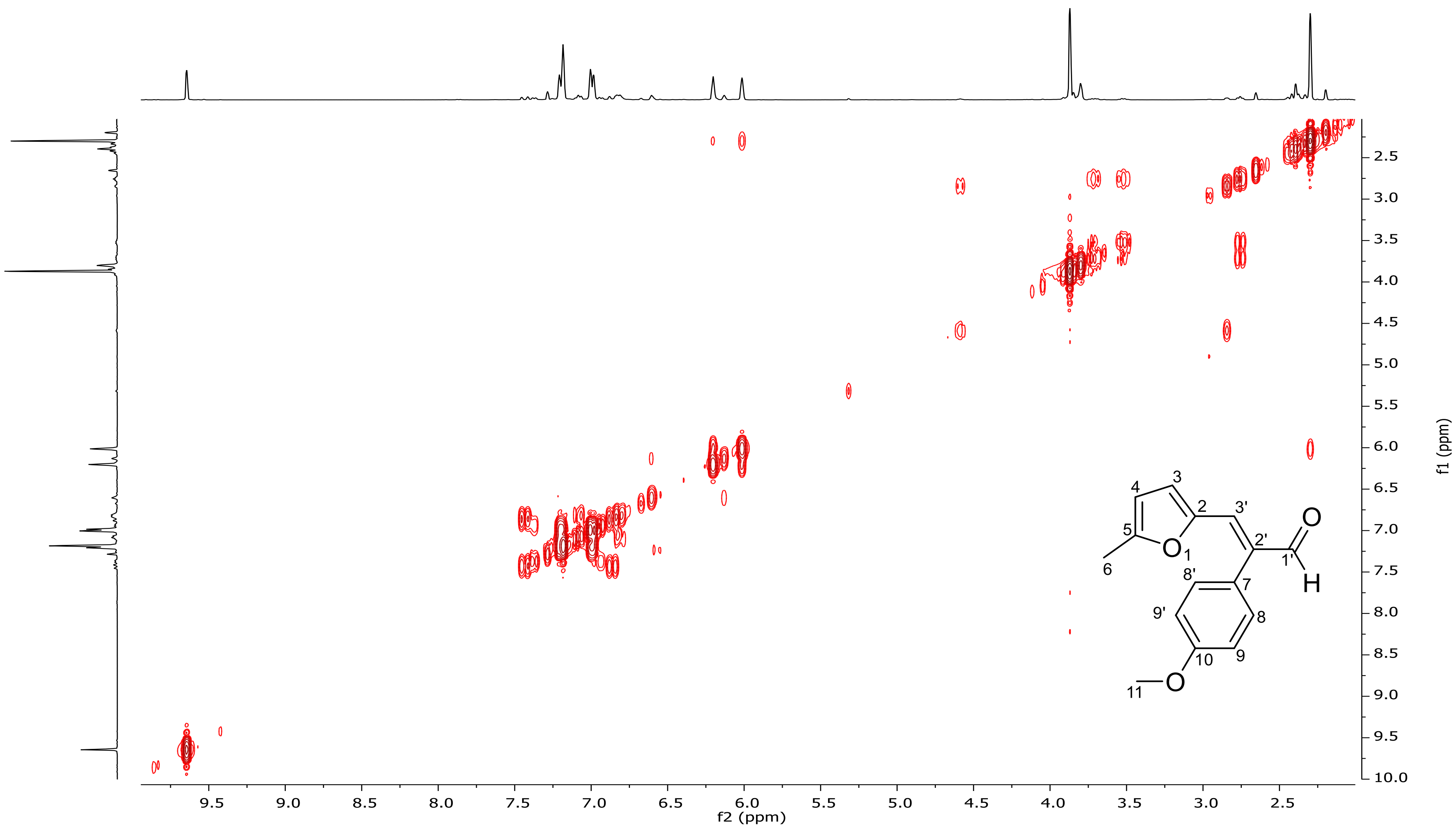

Figure S92: $^1H,^1H$-COSY (400 MHz, $CDCl_3$) of compound **15**.

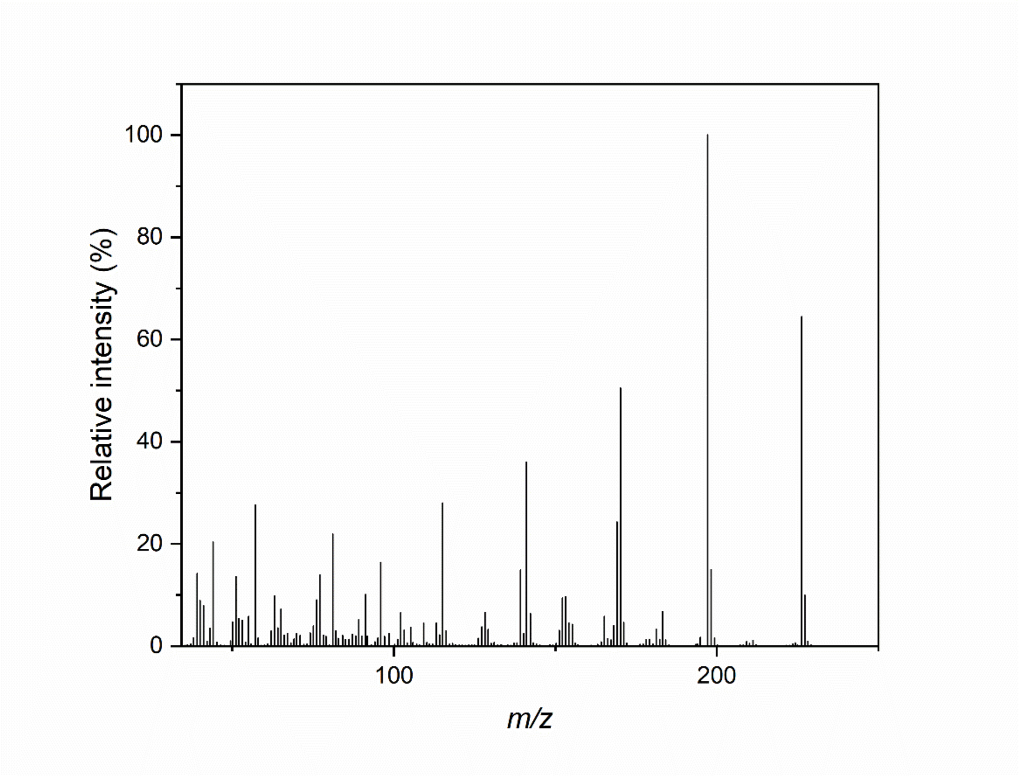

Figure S93: Mass Spectrum(IE, 70 eV) of compound **27**.

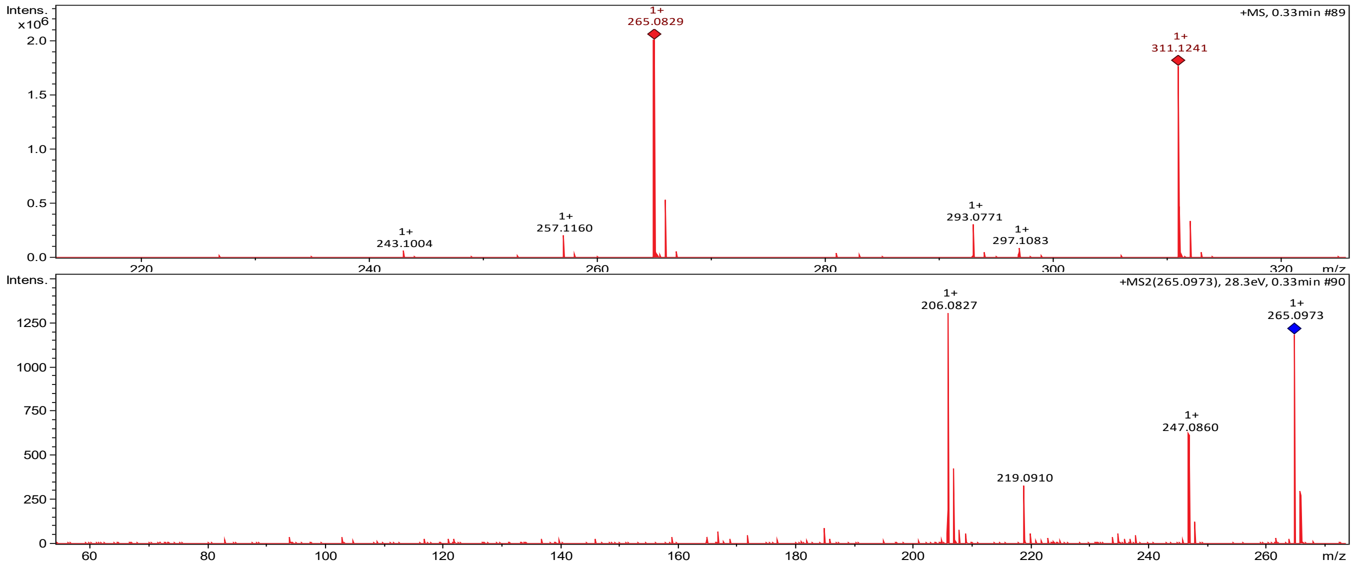

Figure S94: High-resolution mass spectrum (HRMS, ESI) of compound **27.**

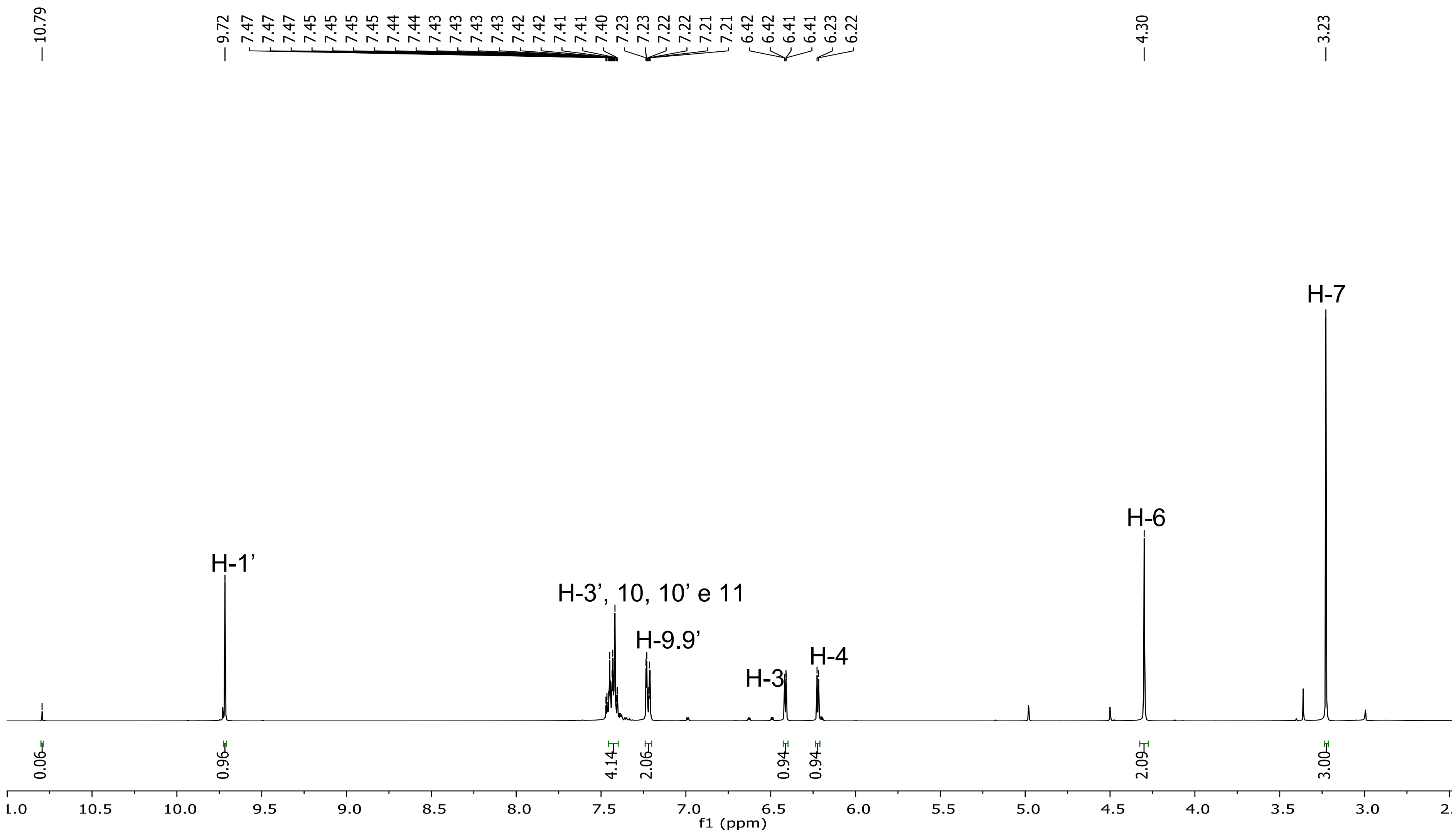

Figure S95: ^{1}H NMR (300 MHz, $CDCl_3$) of compound **27**.

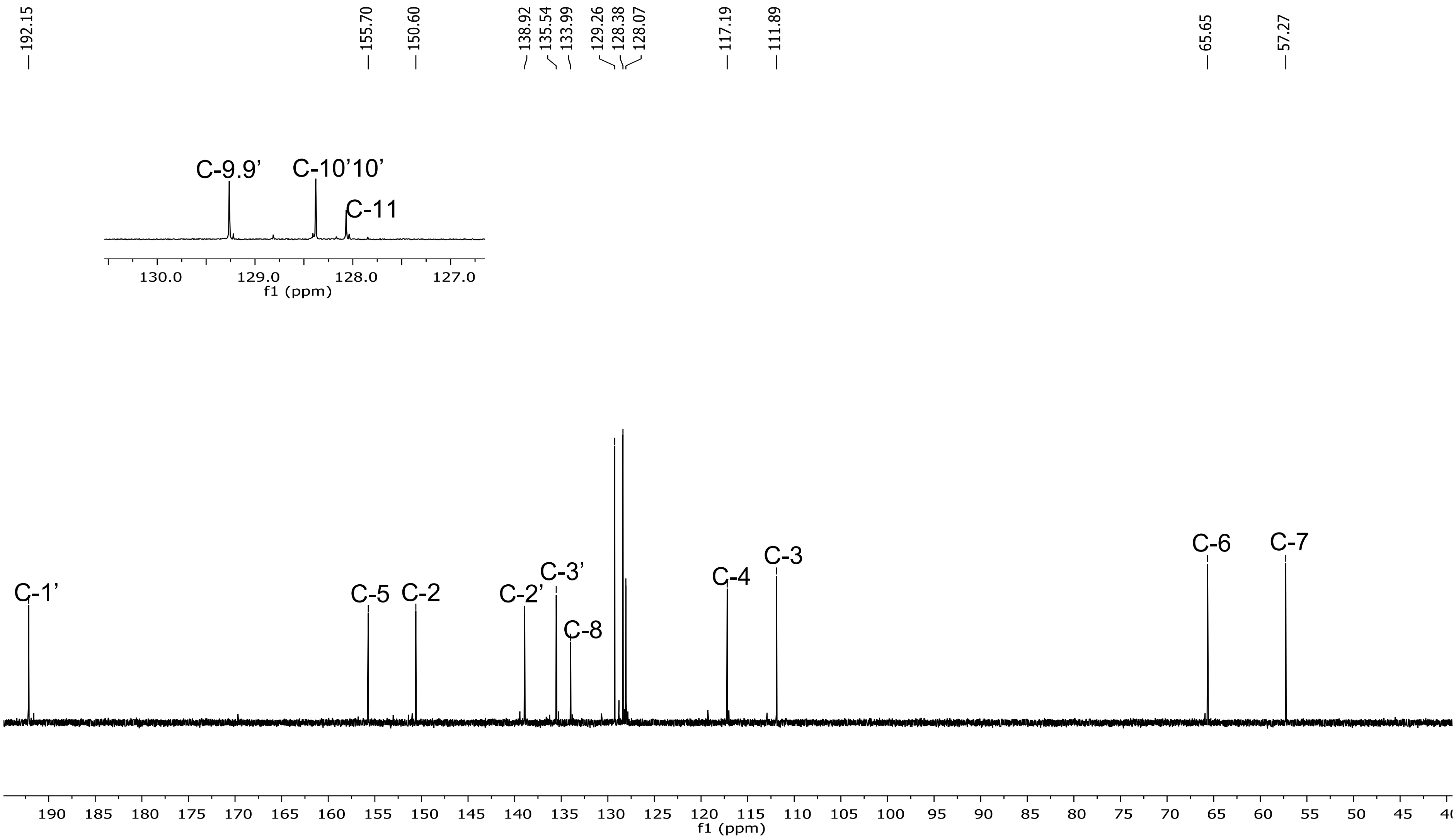

Figure S96: ^{13}C NMR (101 MHz, $CDCl_3$) of compound **27**.

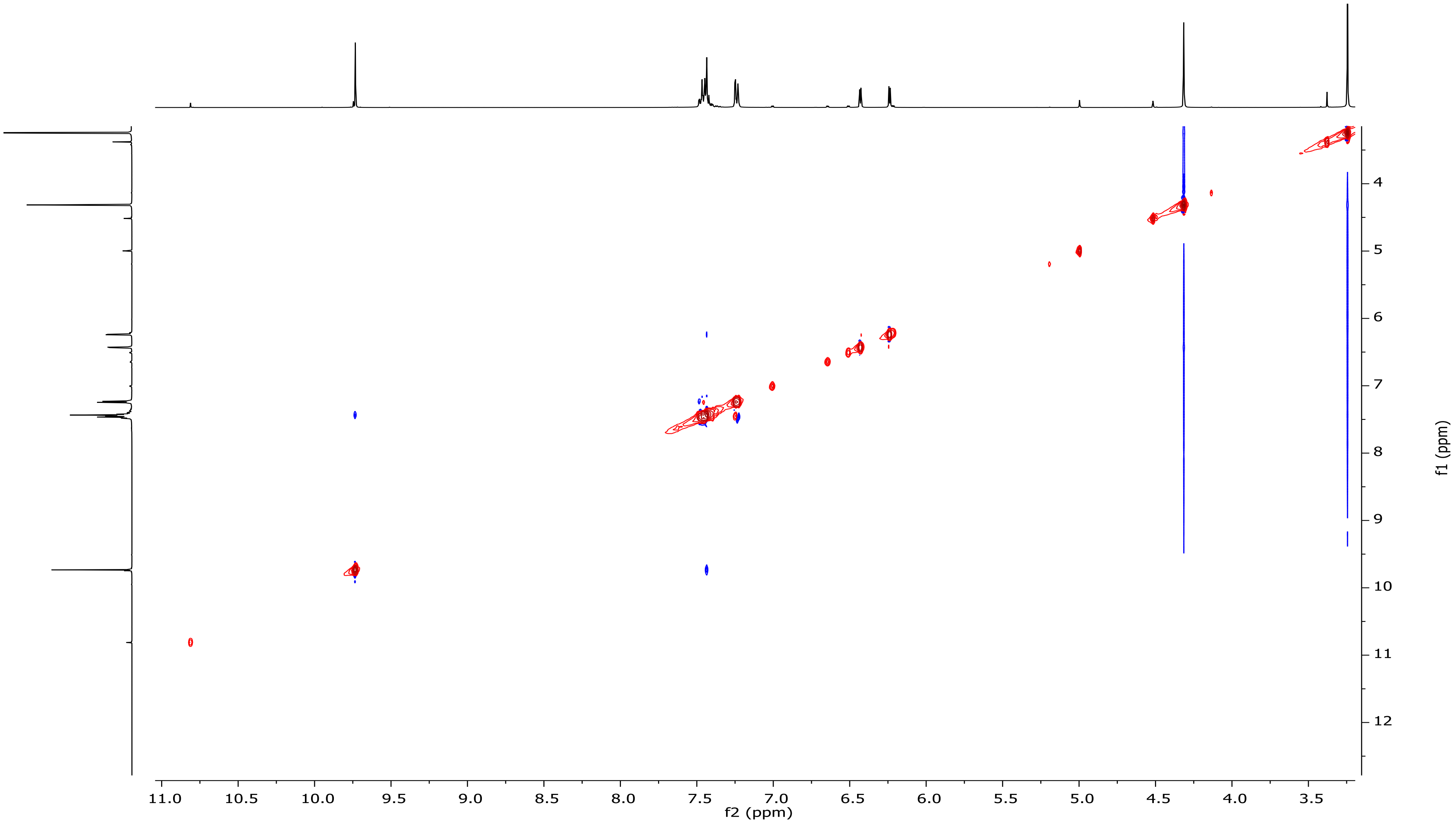

Figure S97: ^{1}H,^{1}H-NOESY (400 MHz, $CDCl_3$) of compound **27**.

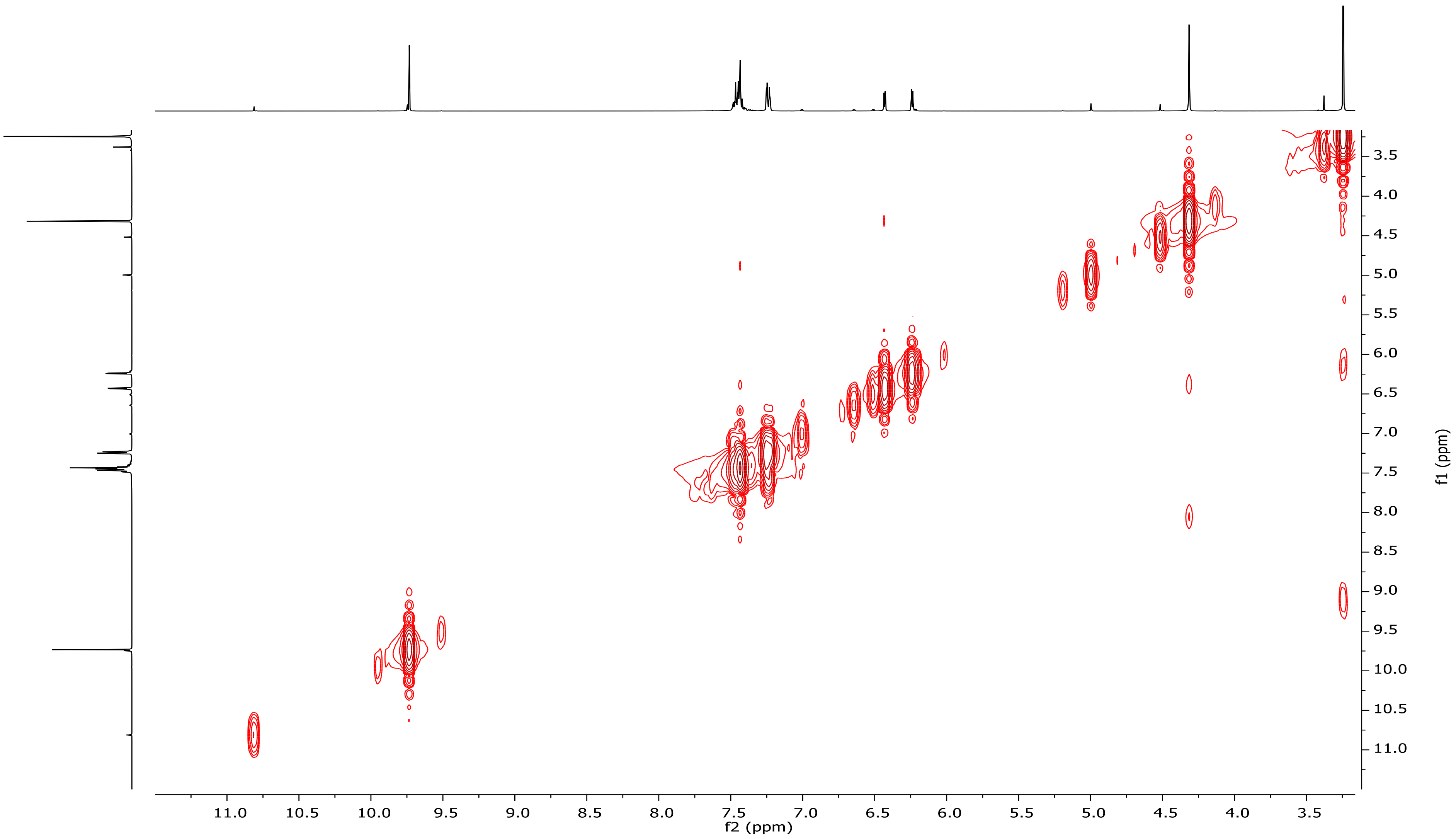

Figure S98: $^{1}H,^{1}H$-COSY (400 MHz, $CDCl_3$) of compound **27**.

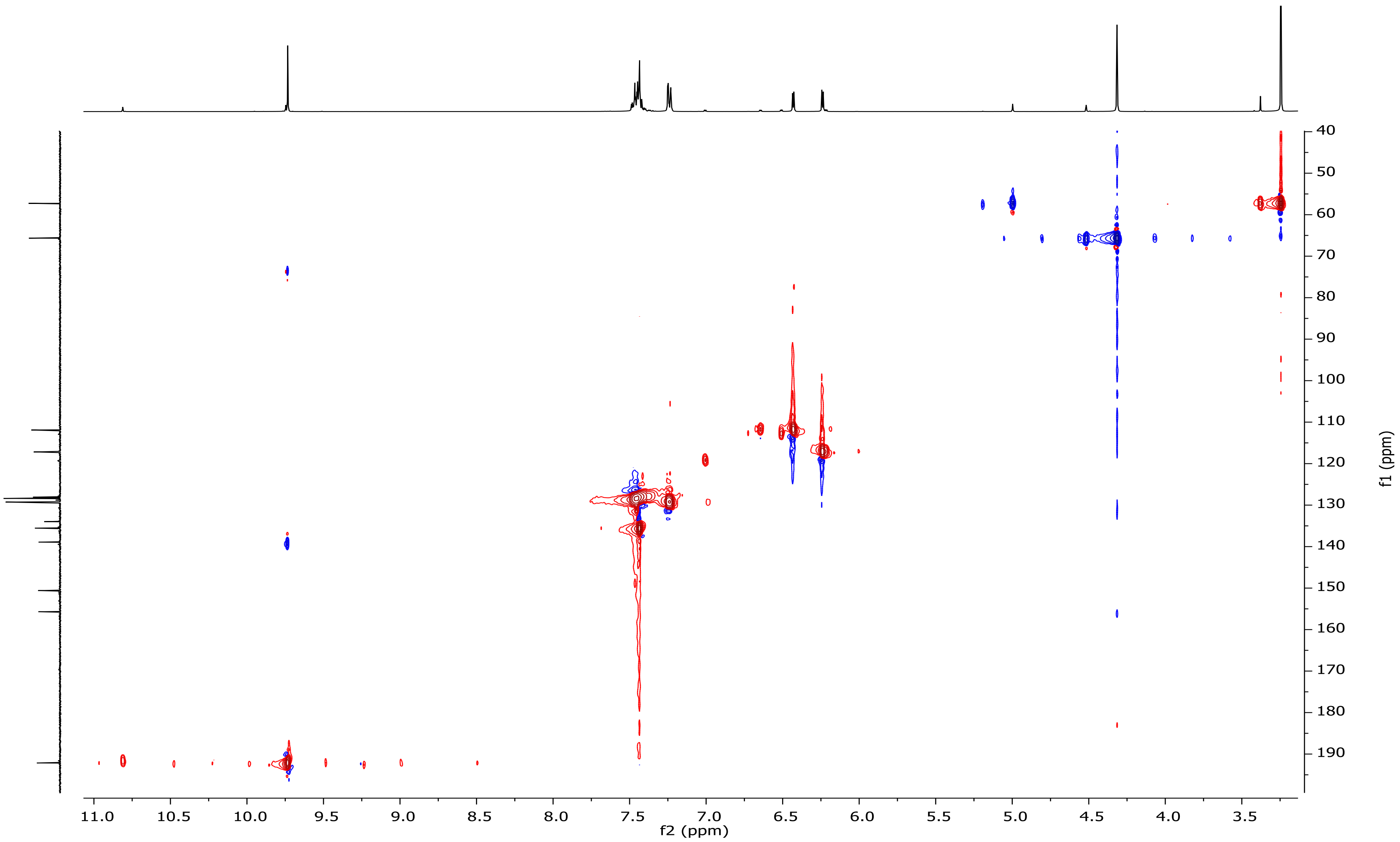

Figure S99: ^{1}H,^{13}C-HSQC (400, 101 MHz, $CDCl_3$) of compound **27**.

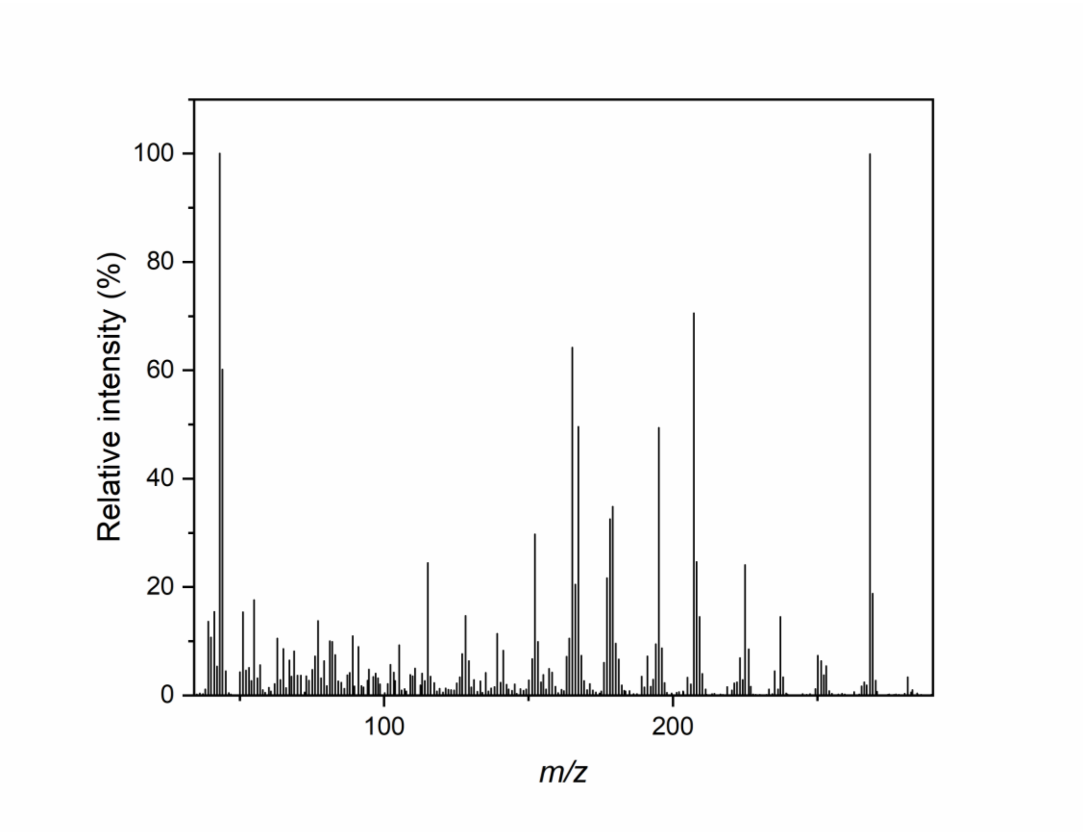

Figure S100: Mass Spectrum(IE, 70 eV) of compound **28**.

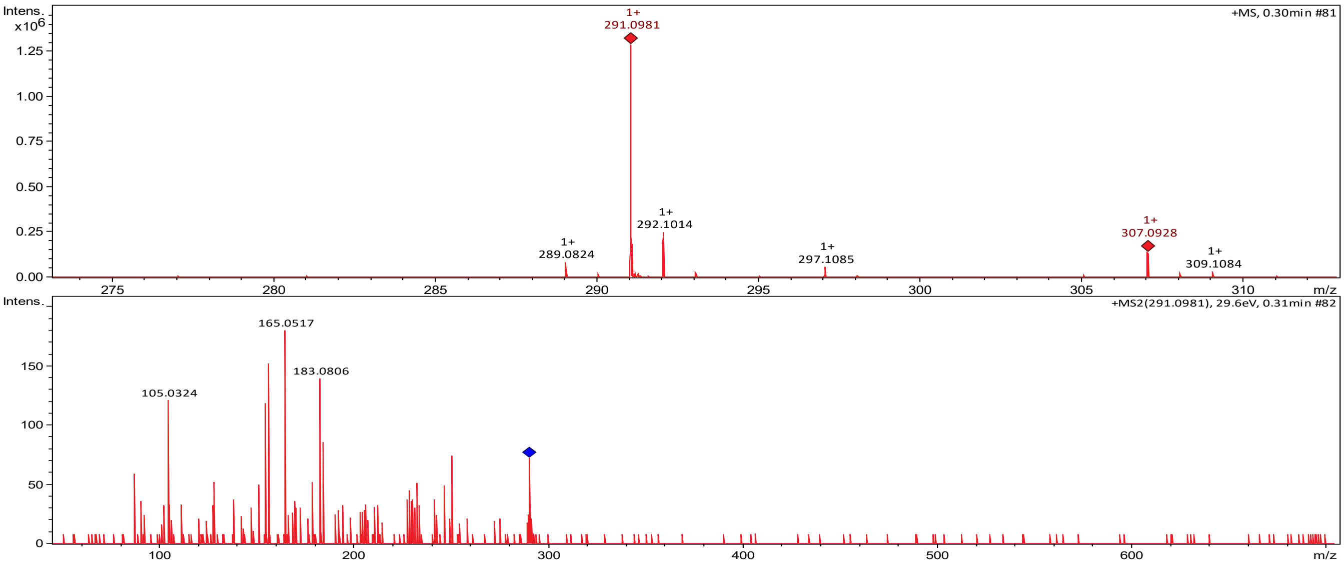

Figure S101: High-resolution mass spectrum (HRMS, ESI) of compound **28.**

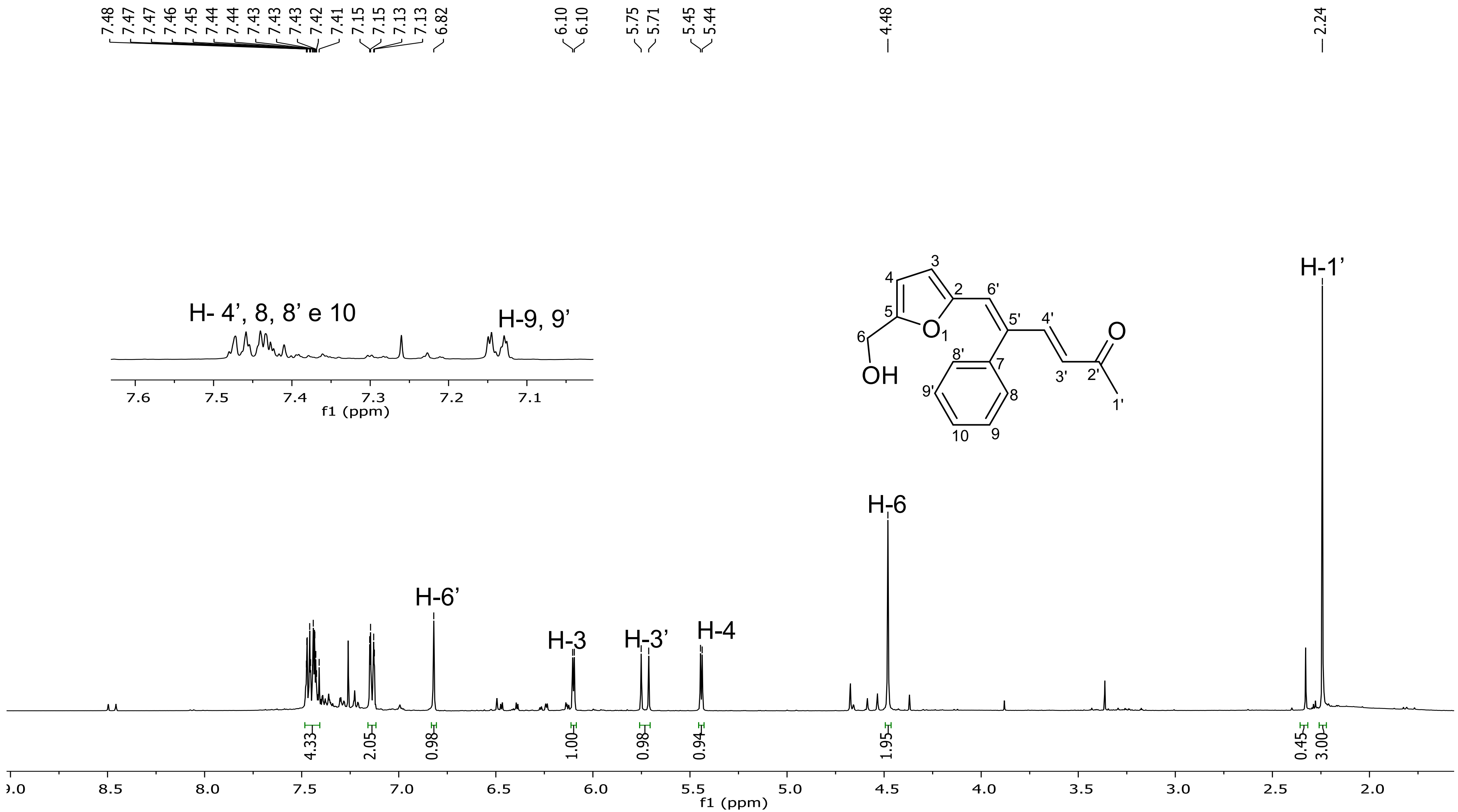

Figure S102: ^{1}H NMR (300 MHz, $CDCl_3$) of compound **28**.

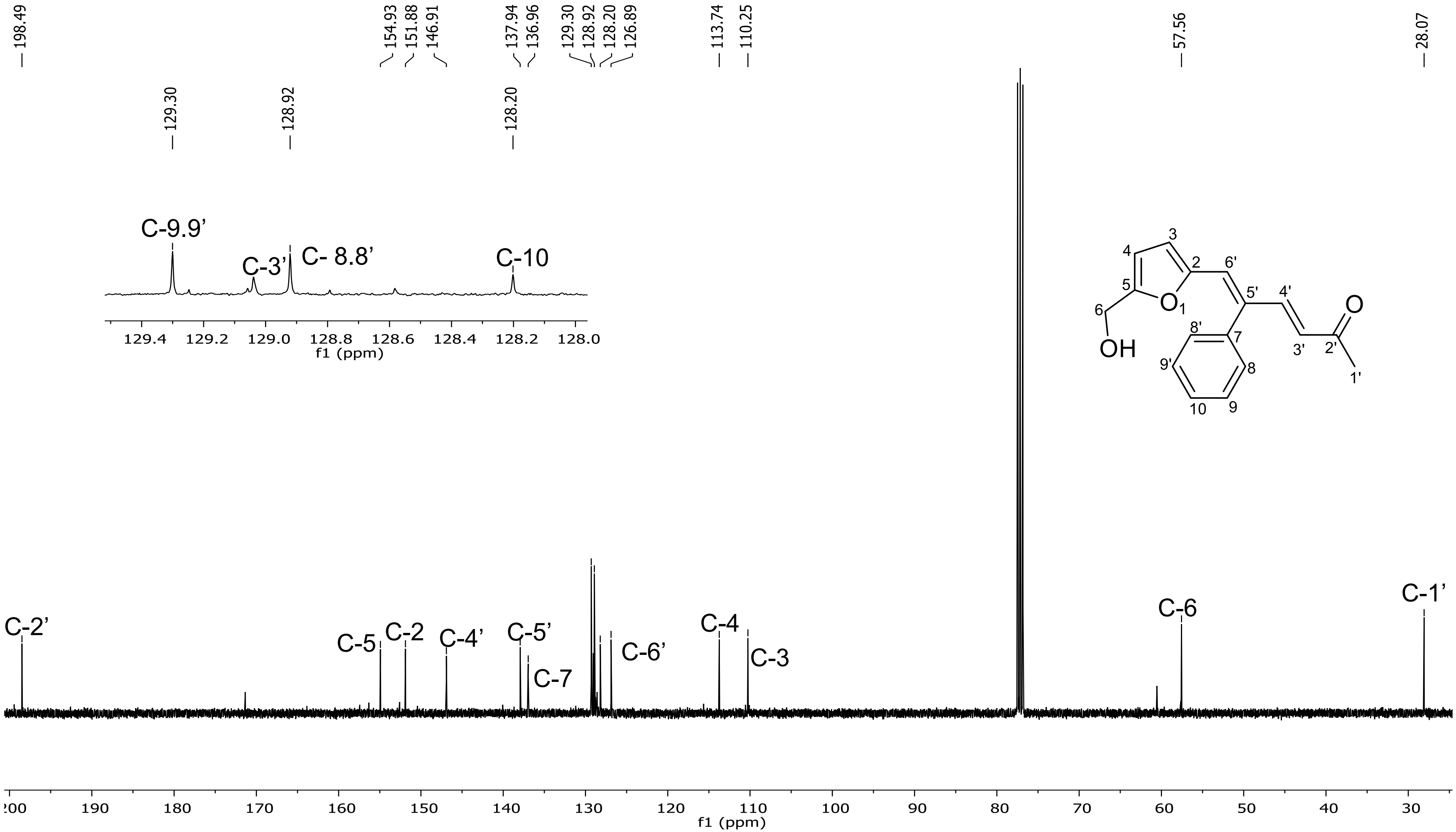

Figure S103: ^{13}C NMR (101 MHz, $CDCl_3$) of compound **28**.

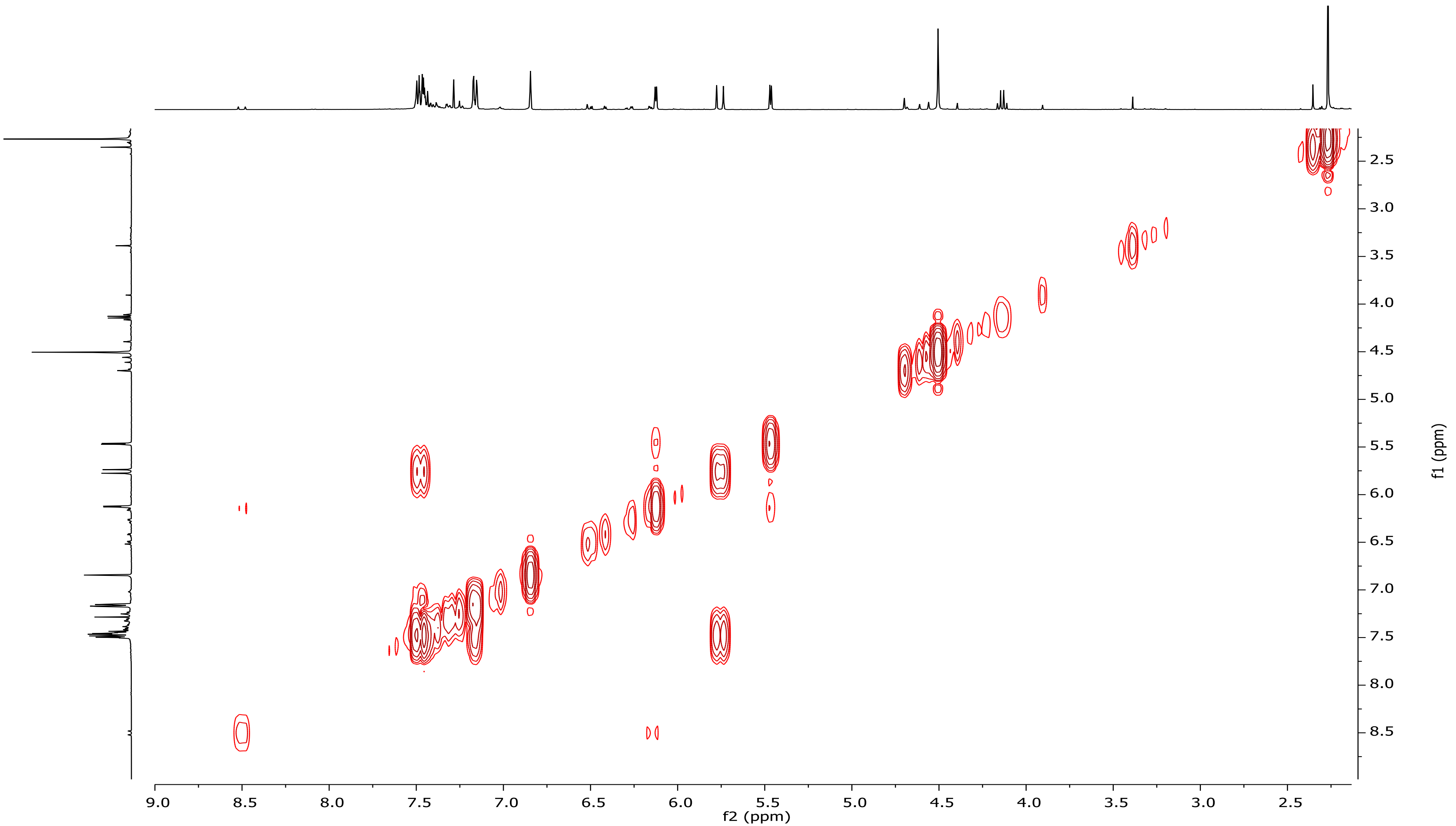

Figure S104: ^{1}H,^{1}H-COSY (400 MHz, $CDCl_3$) of compound **28**.

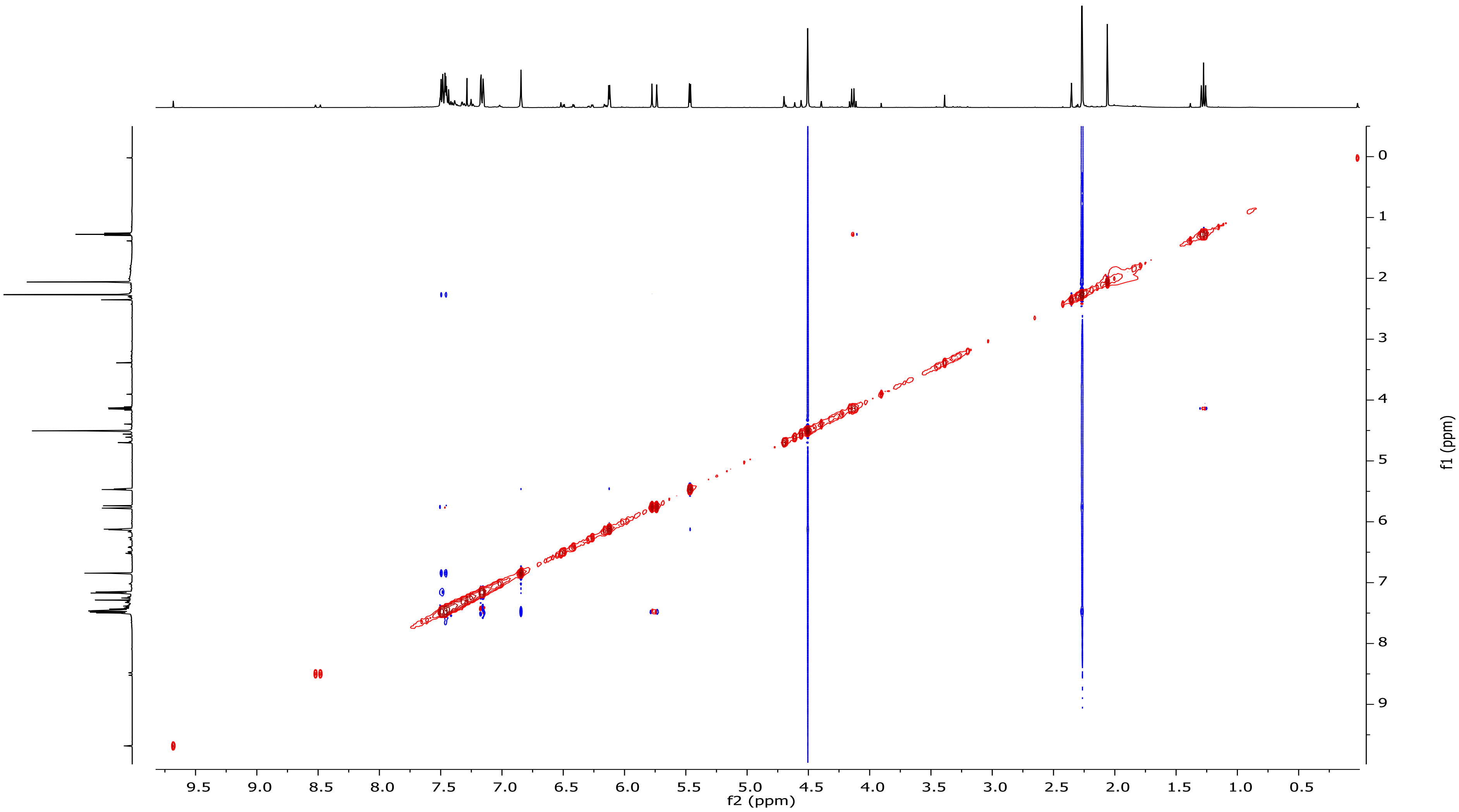

Figure S105: ^{1}H,^{13}C-HSQC (400, 101 MHz, $CDCl_3$) of compound **28**.

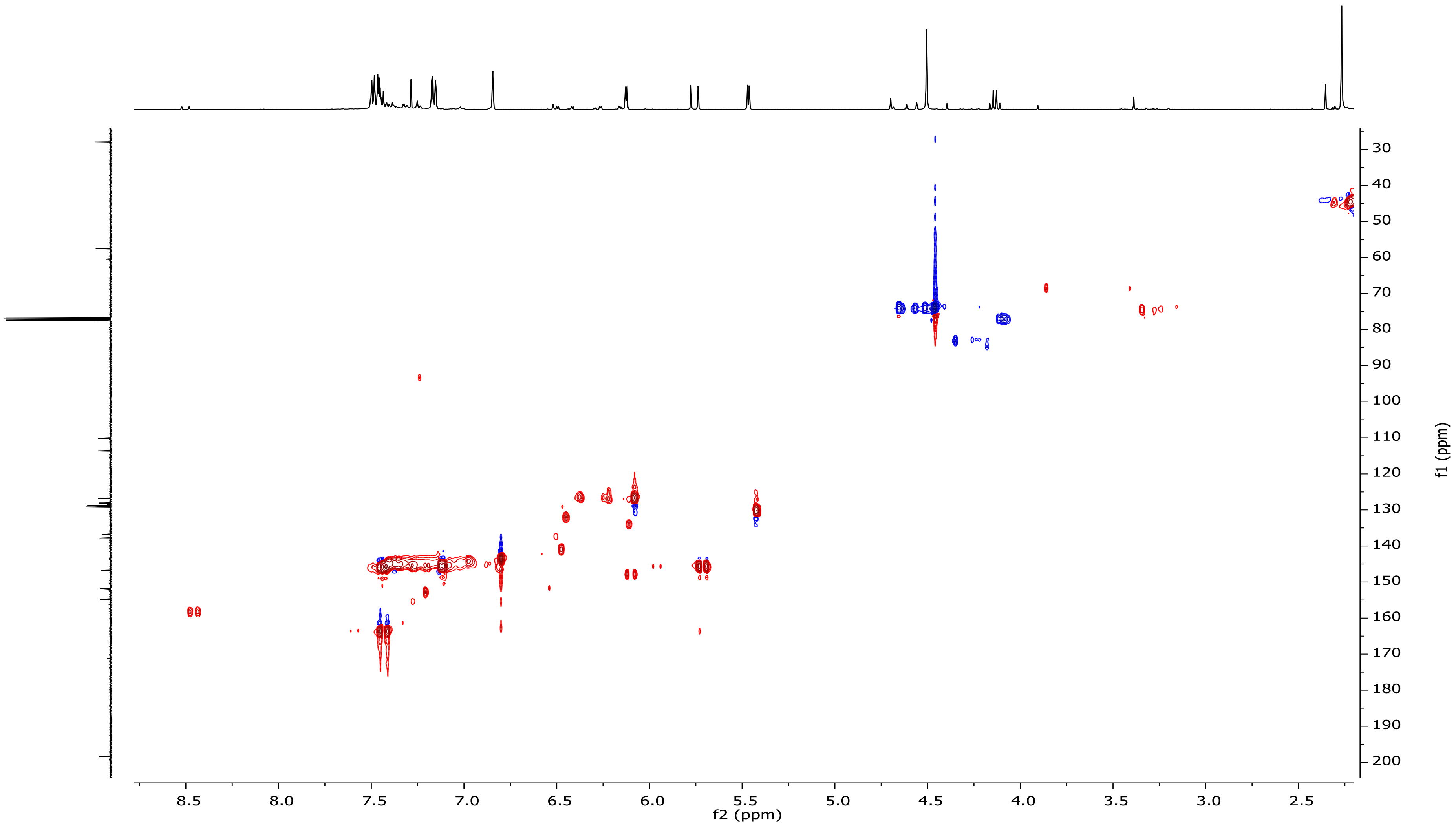

Figure S106: $^{1}H,^{1}H$-NOESY (400 MHz, $CDCl_3$) of compound **28**.
